# Deep learning analysis of long COVID and vaccine impact in low- and middle-income countries (LMICs): development of a risk calculator in a multicentric study

**DOI:** 10.3389/fpubh.2025.1416273

**Published:** 2025-06-26

**Authors:** Ahmed Shaheen, Nour Shaheen, Long COVID Collaboration Study Group in the LMICs, Sheikh Shoib, Fahimeh Saeed, Mudathiru Buhari, Vishal Bharmauria, Oliver Flouty

**Affiliations:** ^1^Alexandria Faculty of Medicine, Alexandria, Egypt; ^2^Psychosis Research Centre, University of Social Welfare and Rehabilitation Sciences, Tehran, Iran; ^3^Department of Health Services, Srinagar, India; ^4^Division of Infectious Disease, University of South Florida, Tampa, FL, United States; ^5^The Tampa Human Neurophysiology Lab and Department of Neurosurgery, Brain and Spine, University of South Florida, Morsani College of Medicine, Tampa, FL, United States; ^6^Center for Vision Research and Center for Integrative and Applied Neuroscience, York University, Toronto, ON, Canada

**Keywords:** long COVID, post-acute sequelae, chronic fatigue syndrome, depression, COVID-19, LMICs, vaccination, hospitalization

## Abstract

**Background:**

Coronavirus disease 2019 (COVID-19), caused by severe acute respiratory syndrome coronavirus 2 (SARS-CoV-2), is a global pandemic affecting millions worldwide. This study aims to bridge the knowledge gap between acute and chronic symptoms, vaccination impact, and associated factors in patients across different low- and middle-income countries (LMICs).

**Materials and methods:**

The study included 2,445 participants aged 18 years and older, testing positive for COVID-19. Data collection involved screening for medical histories, testing records, symptomatology, and persistent symptoms. Validated instruments, including the DePaul Symptom Questionnaire (DSQ-2) and the Patient Health Questionnaire-9 (PHQ-9), were used. We applied a self-supervised and unsupervised deep neural network to extract features from the questionnaire. Gradient boosted machines (GBM) model was used to build a risk calculator for chronic fatigue syndrome (CFS), depression, and prolonged COVID-19 symptoms.

**Results:**

Out of the study cohort, 68.1% of the patients had symptoms lasting longer than 2 weeks. The most frequent symptoms were loss of smell (46.8%), dry cough (40.1%), loss of taste (37.8%), headaches (37.2%), and sore throat (28.9%). The patients also reported high rates of depression (47.7%), chronic fatigue (6.5%), and infection after vaccination (23.7%). Factors associated with CFS included sex, age, and smoking. Vaccinated individuals demonstrated lower odds of experiencing prolonged COVID-19 symptoms, CFS, and depression. The predictive models achieved a high area under the curve (AUC) scores of 0.87, 0.82, and 0.74, respectively.

**Conclusion:**

The findings underscore the significant burden of long-term symptoms such as chronic fatigue and depression, affecting a considerable proportion of individuals post-infection. Moreover, the study reveals promising insights into the potential benefits of vaccination in mitigating the risk of prolonged COVID-19 symptoms, CFS, and depression. Overall, this research contributes valuable knowledge towards comprehensive management and prevention efforts amidst the ongoing global pandemic.

**Clinical trial registration:**

Clinical trials.gov, NCT05059184.

## 1 Introduction

Long COVID, also called ‘post-acute sequelae of COVID-19’, is a complex condition involving a diverse array of often severe symptoms that persist beyond the initial phase of infection with the severe acute respiratory syndrome coronavirus (SARS-CoV-2) (1, 2). It is estimated that globally, a minimum of 65 million individuals are affected by long COVID, considering a conservative estimate of 10% of individuals who have been infected with SARS-CoV-2, which is based on over 651 million documented COVID-19 cases worldwide (3). The incidence of long COVID varies, with estimates indicating it affects approximately 10–30% of individuals who were not hospitalized for their initial COVID-19 infection and 50–70% of those who were hospitalized (1, 2), and 10–12% of individuals who were vaccinated against COVID-19 (4–6). Long COVID is observed across a broad range of age groups and levels of disease severity during the acute phase. Most long COVID cases occur in patients who did not require hospitalization during their initial mild acute illness, overall reflecting the broader demographic distribution of COVID-19 cases (6, 7).

Limited research has examined the burden of long COVID in low- and middle-income countries (LMICs), with the International Severe Acute Respiratory and Emerging Infection Consortium characterization protocol being a notable exception. Among 14,112 recovered COVID-19 patients from 20 countries across four continents, 5,565 (39.4%) were from nine LMICs (8).

In the LMICs, there is a dearth of comprehensive research regarding long COVID syndrome prevalence and risk factors. Nevertheless, existing studies have investigated the extended health consequences faced by survivors of severe acute respiratory syndrome (SARS) and LMICs respiratory syndrome (MERS), two prior coronavirus outbreaks that share similarities with COVID-19 in terms of clinical manifestations, transmission modes, and potential complications (7). Aldhawyan et al. investigated the prevalence and risk factors of long COVID in the Eastern Province of Saudi Arabia, analyzing data from 1,355 recovered patients. Findings highlight the influence of sociodemographic and clinical factors on long COVID symptoms, emphasizing the need for targeted management strategies (9).

The limited characterization of COVID-19 in LMICs may obscure the true extent of long COVID, which remains largely unmeasured. Many LMICs lack the necessary research and surveillance infrastructure to accurately assess its prevalence and impact. Investigating long COVID in these regions is further complicated by weak referral systems and limited capacity for patient follow-up. Additionally, acute COVID-19 cases may be underdiagnosed, especially in LMICs, due to inadequate testing and underreporting of SARS-CoV-2 infections (10).

Given these challenges, we conducted this multicentric collaborative study to comprehensively evaluate the acute and chronic symptoms associated with COVID-19. We also investigated potential comorbidities, particularly focusing on depression, after COVID-19 vaccination, especially in the LMICs where data remain limited. The secondary objective is to develop and validate a machine learning risk calculator for patients in LMICs.

## 2 Methods

This study is designed to explore long COVID symptoms in individuals who tested positive for COVID-19. A total of 2,445 participants from Egypt, India, Pakistan, Syria, and Yemen were included. Data were collected through structured interviews, incorporating validated questionnaires that assessed demographics, medical history, COVID-19 testing, symptoms, and treatments. The methodology employed various statistical and machine learning techniques, including principal component analysis (PCA), clustering, and deep neural networks, to analyze the data and predict outcomes such as fatigue, depression, and symptom duration. We adhered to ethical standards and utilized secure data storage for analysis.

### 2.1 Study design, setting and participants

The current investigation constitutes a cross-sectional involving structured screening interviews, mirroring those conducted on individuals who have experienced symptoms for a duration ranging from 1 week to less than 6 months (11). The study was registered at clinicaltrials.gov under the NCT number: NCT05059184. The sampling method was based on a convenience sampling approach, targeting individuals aged 18 years and older who had tested positive for COVID-19 through either diagnostic or antibody tests. Participants were recruited from five countries—Egypt, India, Pakistan, Syria, and Yemen—between September 2021 and September 2022. We targeted individuals aged 18 years and above who had tested positive for COVID-19, either through diagnostic or antibody tests and had experienced symptoms. The Centers for Disease Control and Prevention (CDC) indicate that long COVID can be identified as early as 4 weeks after the initial infection (12).

### 2.2 Variables and data sources

Participants were screened using validated measurement instruments to collect data on their medical history, COVID-19 testing, symptomatology, treatments, and vaccination. The research involved a 155-item questionnaire covering a variety of factors:

- Demographics and baseline characteristics (15 items)
- COVID-19 testing (4 items)
- COVID-19 experience (6 items)
- Hospitalization (3 items)
- Treatments (2 items)
- Vaccination (5 items)
- DePaul Symptom Questionnaire (DSQ) for chronic fatigue symptoms (54 items)
- Patient Health Questionnaire (PHQ-9) for depression (9 items)
- Other COVID-19 symptoms (57 items).

The DSQ-2 is designed to assess ME/CFS symptoms, encompassing fatigue, post-exertional malaise, sleep disturbances, pain, neurological/cognitive impairments, as well as autonomic, neuroendocrine, and immune symptoms. It employed a 14-question short version with Likert-type scales for frequency and severity ratings (13, 14). The Patient Health Questionnaire (PHQ-9), comprised nine questions about mood and feelings experienced over the preceding 2 weeks, with responses rated on a 4-point scale. Cumulative scores were utilized to categorize levels of depression (15). Other symptoms were identified through a comprehensive literature review (16–18).

### 2.3 Study conduct

Local health authorities systematically gathered data from confirmed COVID-19 cases. Various groups within the same institution were allowed to engage, with the inclusion of new collaborators. Data collection transpired via direct physician-patient interviews at outpatient clinics, facilitated by written informed consent. Stringent measures were instituted to uphold patient confidentiality and data integrity. Collected data, sourced from both local and national entities, was channeled to a secure platform overseen by the central research team, utilizing Microsoft Forms. The subsequent secure storage of data for analysis adhered rigorously to ethical standards and the Declarations of Helsinki.

The study was conducted following the Declaration of Helsinki to safeguard participant and patient rights (19). The Institutional Review Board (IRB) diligently adhered to both national and local standards throughout the approval process. Subsequently, comprehensive briefings detailing project results and findings were provided to actively engaged governmental and academic entities. Furthermore, IRB approvals were secured from the Ethics Committees associated with collaborating centers responsible for data collection.

### 2.4 Statistical methods

#### 2.4.1 Data cleaning and variable preparation

We built an R package for data preparation and variables wrangling for this project “Shaheen. Questionnaire” which can be found at: https://github.com/doctor-shaheen/Shaheen.Questionnaire.

The original data frame contained 173 variables divided into sections as follows demographic and pre-COVID-19 disease history, COVID-19 infection and treatments, COVID-19 vaccination, chronic fatigue history, DSQ questionnaire, and PHQ-9 questionnaire.

Multichoice questions were converted into dummy variables, and we excluded variables that were frequent in less than 10% of the population due to the high rate of missing data and imbalance issues that could be introduced into the statistical models. There were 500 remaining variables. The remaining data had a missing rate of less than 0.01%.

The missing data were explored for the cause of any type of missing, and data that were missing due to a known cause (the patients did not experience the symptoms, or the criteria did not apply to them) were solved. The remaining variables with unknown missing data were imputed by the mode. We used the IOM Clinical Case Definition algorithm to make a scoring system for chronic fatigue questionnaires (20). The IOM Clinical Case Definition is a widely used diagnostic framework for CFS, also known as myalgic encephalomyelitis (ME/CFS) (20). Symptom duration was categorized after removing outliers > 40 days because it still had non-gaussian distribution (Supplementary Figures S1, S2).

#### 2.4.2 Data preprocessing

Highly correlated variables (>0.99), and zero variance variables were excluded. Categorical variables were converted into dummy variables, then we preprocessed the data using three methods: centering, scaling, and Yeo-Johnson transformation. These methods are used to standardize the data and make it more symmetric and normal-like (21). We used resampling methods for the model including fatigue as an outcome to overcome the imbalanced representation of the variables (22).

### 2.5 Machine learning

#### 2.5.1 Clustering and principal component analysis (PCA)

We performed density-based clustering on the 50 outcome variables using the HDBSCAN (Hierarchical Density-Based Spatial Clustering of Applications with Noise) algorithm, which can identify clusters with different densities and shapes. We employed an R package that provides an implementation of the HDBSCAN algorithm. We optimized the “minPts” parameter, which determines the minimum cluster size, by running 200 iterations and selecting the value that yielded the highest sum of stability scores. The stability scores quantify how sensitive each cluster is to variations in the parameter (23).

We also applied PCA analysis to obtain a low-dimensional representation of the outcome variables that preserve most of the data variability. PCA is a linear transformation method that computes orthogonal directions (principal components) that account for the most variation in the data. We determined the number of principal components to retain using the elbow method, which is a heuristic technique that locates a point of inflexion in the plot of cumulative variance explained versus several components. We used the principal components for subsequent analysis (24).

#### 2.5.2 Deep neural networks

We constructed a deep-learning auto-encoder network using the TensorFlow framework (25). The model has two components an encoder and a decoder. The encoder receives an input layer with 54 features and applies three dense layers with 50, 2, and 1 neuron respectively, each followed by a dropout layer with a rate of 0.1. The first two dense layers use the “relu” activation function, while the last one does not use any activation function. The encoder produces a single value that corresponds to the latent representation of the input, which we extracted and used as a dependent variable representing the outcomes. We then fitted a linear regression model to examine the relationship between the predictors and the 50-variable outcomes using this latent variable. The decoder takes the output of the encoder and applies three dense layers with 50, 2, and 54 neurons respectively, each followed by a dropout layer with a rate of 0.1. The first two dense layers use the “relu” activation function, while the last one does not use any activation function. The decoder generates a vector of 54 values that corresponds to the reconstructed input.

In addition to the auto-encoder network, we used another method to extract embedding features from the high-dimensional fifty-four variable outcomes. We used self-supervised contrastive representation learning, which is a technique that learns to distinguish between similar and dissimilar data points based on their features. We used the PyTorch-SCARF (Self-Supervised Contrastive Learning using Random Feature Corruption) algorithm, which is an implementation of the model architecture described by Bahri et al. (26). The SCARF model, like the auto-encoder, can learn hidden features from the data and can remove irrelevant and unnecessary information, resulting in a lower-dimensional representation of the data. The SCARF model works by randomly corrupting some of the features of the data and then learning to align the original and corrupted representations using a contrastive loss function. We trained the SCARF model to extract a two-dimensional representation of the fifty-four variable outcomes. The resulting embeddings were further analyzed and used as a dependent variable to build another linear regression model with independent variables (26).

#### 2.5.3 Predicting outcomes (depression, fatigue, and symptoms duration)

We used the “h2o” package to build three machine-learning models to predict depression, chronic fatigue, and symptom duration. We used the AUTOML method, which automatically selects and compares multiple algorithms based on their performance (27). We found that tree-based models, such as random forests and gradient boosting machines, performed well with our data, so we further fine-tuned their hyperparameters. We used cross-validation with 5 folds to train the models on 80% of the data and then tested them on the remaining 20% of the data. To evaluate and diagnose the models, we used several metrics, such as the area under the curve (AUC), receiver operator curve (ROC), Brier score, accuracy, sensitivity, specificity, and confusion matrix. These metrics measure how well the models can classify the outcomes and how often they make errors. (For all model training and hyperparameters, see Supplementary files).

#### 2.5.4 Interpretation of models

To interpret the models and understand the effects of the predictor variables on the outcome variable, we used two methods: Shapely Additive Explanations (SHAP) importance and variable importance plots. SHAP importance is a method that measures the contribution of each variable to the model prediction by computing the Shapley values, which are based on game theory. Shapley values represent the average marginal contribution of a variable across all possible subsets of variables. SHAP importance plots show the mean absolute Shapley values for each variable, ranked from highest to lowest. The higher the SHAP value is, the more important the variable for model prediction (28).

Variable importance plots are another method that measures the importance of each variable by calculating the decrease in model performance when a variable is randomly permuted. This means that the variable is replaced by noise, which breaks its relationship with the outcome variable. Variable importance plots show the decrease in model performance for each variable, ranked from the highest to the lowest. The higher the decrease is, the more important the variable for model prediction (27).

#### 2.5.5 Statistical analysis

We used mean, standard deviation, frequency, and percentages to describe and summarize the variables. We used chi-square, t-test and ANOVA, correlation, unadjusted logistic, linear regression, and standardized mean difference (SMD) for univariate analysis. We also built adjusted linear regression and logistic regression models for multivariate analysis. Backward stepwise selection was used to select variables that were included in the adjusted multivariate models. The dependent variables for the models were as follows: chronic fatigue score, depression score and symptoms duration, auto-encoder bottleneck layer, and embedding.

We assessed the model fit and calibration using the Akaike information criterion (AIC), the C-statistic, and the Hosmer-Lemeshow (H&L) test. The AIC measures the trade-off between the complexity and the goodness of fit of the model, with lower values indicating a better fit. The C-statistic measures the ability of the model to correctly classify the outcome, with values ranging from 0.5 (no discrimination) to 1.0 (perfect discrimination). The H&L test compares the observed and expected frequencies of the outcome in subgroups of the data, with a non-significant *p*-value indicating a good calibration.

All statistical analysis and model building were done using R version 4.3.1 and Python 3.11.4.

The model training was done using the M1 Apple Silicon CPU (29).

## 3 Results

### 3.1 Demographics

**Figure 1:**
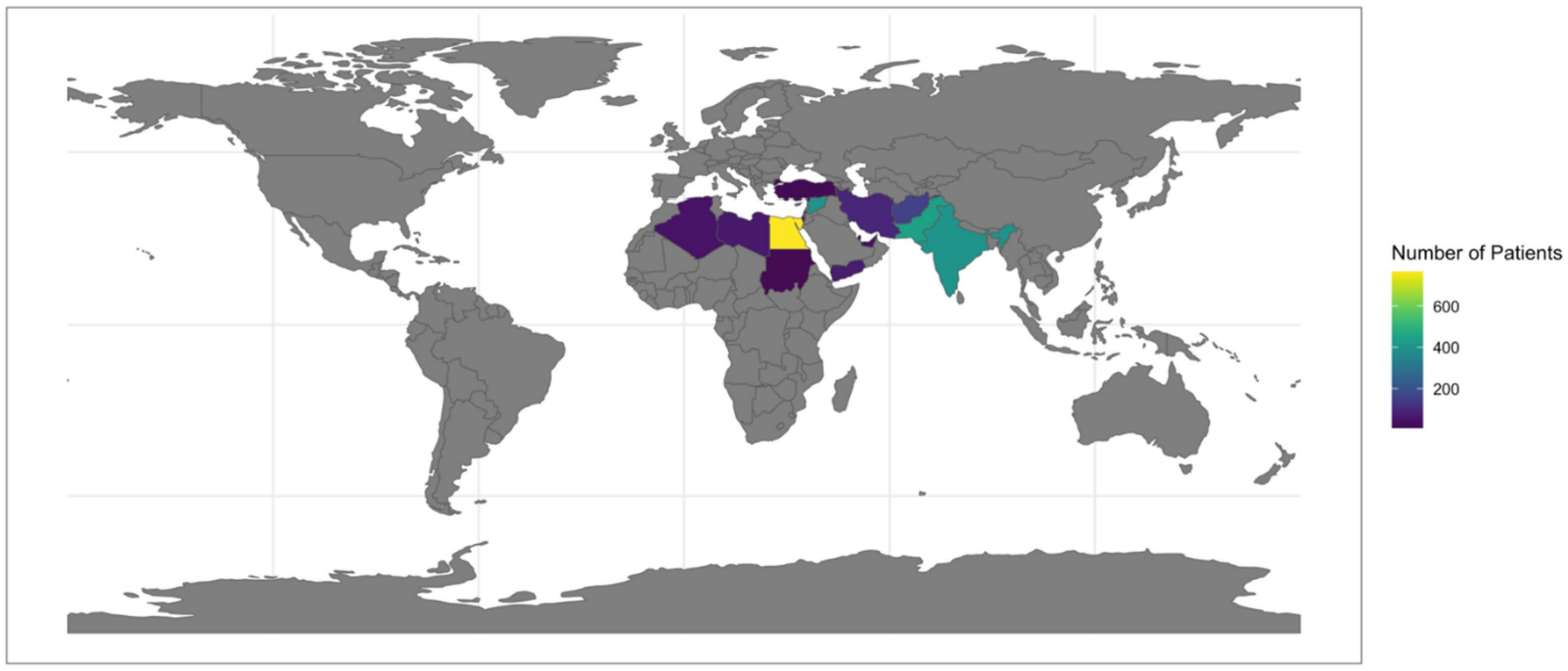
Geographic distribution of patients by country. Color gradient interpretation: yellow-green: highest number of patients (>600). Teal-blue: moderate number of patients (200–600). Dark purple: lowest number of patients (<200). Geographic representation: highest patient representation: Egypt, Pakistan, Syria, India. Moderate representation: Afghanistan, Iran, Libya, Yemen. Lower representation: Algeria, West Bank and Gaza, UAE, Turkey, Qatar, Sudan.

We included 2,445 patients, 43.5% of whom were male (1,063). Participants were from Egypt (29.98%), Pakistan (17.47%), India (15.68%), Syria (15.75%), and other countries (21.11%). The largest age group was 40–49 years (53.6%), followed by 50–59 (12.3%), 18–29 (10.5%), ≥60 (10.0%), and 30–39 (9.8%). Most were non-smokers (79.7%), while 9.7% were occasional and 10.5% regular smokers. The average BMI was 25.43 (±5.06), and 35.7% were healthcare professionals. (Table 1; Figure 1).

**Table 1:** Shows the demographic, pre-COVID-19 medical history and patient country distribution.

Variable	*N* (%)
Total Participants	2,445
BMI [mean (SD)]	25.42 (5.07)
Age group	(%)
18–29	267 (10.9)
30–39	244 (10.0)
40–49	1,369 (56.0)
50–59	312 (12.8)
>60	253 (10.3)
Sex = Male (%)	1,063 (43.5)
Healthcare professional = Yes (%)	912 (37.3)
Smoking	(%)
Never	2039 (83.4)
Occasionally	248 (10.1)
Regularly	158 (6.5)
Country	%
Afghanistan	158 (6.1)
Algeria	48 (1.8)
Egypt	767 (29.9)
India	401 (15.6)
Iran	92 (3.5)
Libya	59 (2.3)
Pakistan	448 (17.4)
Qatar	10 (0.39)
Sudan	9 (0.35)
Syria	403 (15.7)
Turkey	10 (0.39)
United Arab Emirates	44 (1.7)
vWest Bank and Gaza	42 (1.6)
Yemen	67 (2.6)

### 3.2 Pre-infection medical history

Pre-existing conditions were grouped into gastrointestinal, haematological, respiratory, neurological, allergies, and other categories. Common conditions included vertigo (15.7%), mould infections (12.8%), environmental allergies (12.8%), anaemia (9.9%), insomnia (9.9%), hypertension (9.7%), and mental health diagnoses (9.6%) (Supplementary Table S1; Supplementary File).

### 3.3 Vaccination, hospitalization, and treatments

Most of the vaccinated patients had received 2 shots (57.6%), while 23.4% were unvaccinated. The number of vaccinations was highly correlated (Pearson correlation r = 0.79) with the vaccination status variable (Supplementary Table S2).

Of the total sample, 304 patients (12.4%) were hospitalized, and 252 (10.3%) required oxygen support. Most (75.8%) received a COVID-19 vaccine, with AstraZeneca (23.0%), Sinovac (21.5%), Sinopharm (9.9%), Pfizer (10.5%), and Moderna/others (10.1%) being the most common. Common treatments included paracetamol (35.6%), aspirin (29.0%), azithromycin (36.9%), steroids (20.3%), and ibuprofen/naproxen (18.8%). Other medications included antihistamines (14.6% type 1, 9.5% type 2), antioxidants (7.9%), and omega-3 (8.8%) (Supplementary Table S2).

### 3.4 Symptoms of COVID-19 infection

**Figure 2:**
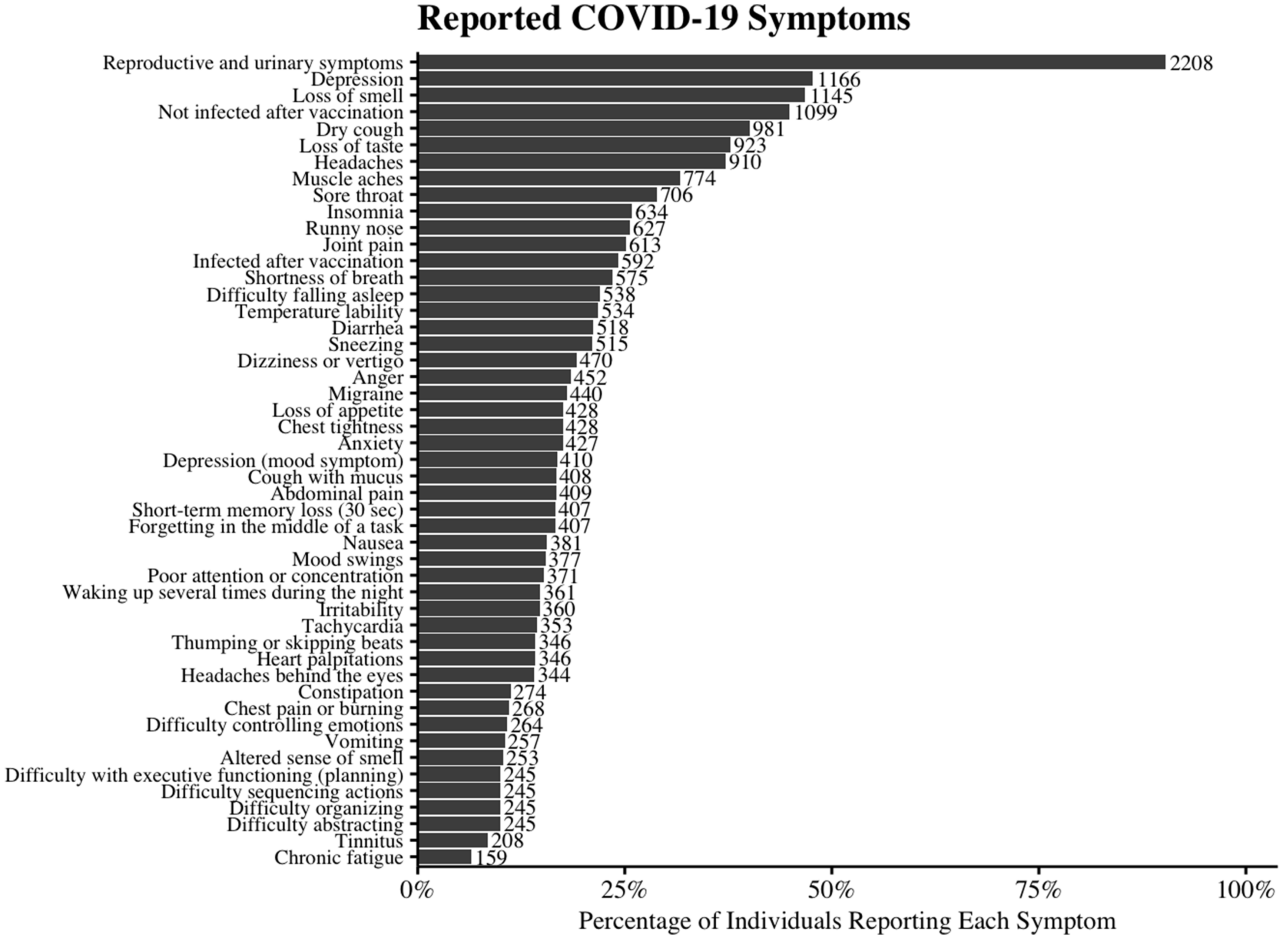
Reported Symptoms of COVID-19 Infection. The horizontal bar chart illustrates the prevalence of various reported symptoms among individuals with COVID-19. Each bar represents the percentage of participants who reported experiencing the given symptom. The number beside the bar indicates the number of cases representing that percentage.

The most common symptoms were loss of smell (46.8%), dry cough (40.1%), loss of taste (37.8%), headaches (37.2%), and sore throat (28.9%), lasting an average of 13.63 ± 17.50 days. Depression (47.7%), chronic fatigue (6.5%), and post-vaccination infection (24.2%) were also reported. Over 10% experienced migraine, tinnitus, dizziness, memory loss, brain fog, mood changes, and various systemic symptoms (Supplementary Table S3; Figure 2).

### 3.5 Female health

1,322 women were included with COVID-19, 80.2% of whom had regular menstrual cycles, and 2.0% were pregnant. Prolonged symptoms (>2 weeks) occurred in 68.1, and 7.6% reported chronic fatigue (Supplementary Table S3). Regular cycles were significantly linked to prolonged symptoms (*p* < 0.001, SMD 0.368), while pregnancy had no impact. Multivariate analysis confirmed regular cycles as an independent risk factor (OR 1.50, *p* = 0.017) (Table 2, Supplementary Tables S5, S6).

**Table 2:** Logistic regression model for symptoms duration in female group only; AIC = 1459.4, C-statistic = 0.697, H&L = Chi-sq (8) 8.61 (*p* = 0.376).

Dependent: symptoms duration		<2 weeks	>2 weeks	OR (univariable)	OR (multivariable)
Menstruation	No	113 (45.6)	135 (54.4)	-	-
	Yes	282 (28.1)	721 (71.9)	2.14 (1.61–2.85, *p* < 0.001)	1.50 (1.07–2.09, *p* = 0.017)
Before COVID vertigo dizziness	No	306 (29.3)	738 (70.7)	-	-
	Yes	116 (41.7)	162 (58.3)	0.58 (0.44–0.76, *p* < 0.001)	0.61 (0.45–0.82, *p* = 0.001)
Hospitalization	No	343 (28.8)	846 (71.2)	-	-
	Yes	79 (59.4)	54 (40.6)	0.28 (0.19–0.40, *p* < 0.001)	0.32 (0.20–0.51, *p* < 0.001)
COVID 19 vaccination	No	136 (43.5)	177 (56.5)	-	-
	Yes	286 (28.3)	723 (71.7)	1.94 (1.49–2.52, *p* < 0.001)	1.44 (1.07–1.93, *p* = 0.016)^**^
Pre-existing conditions anaemia	No	345 (30.8)	775 (69.2)	-	-
	Yes	77 (38.1)	125 (61.9)	0.72 (0.53–0.99, *p* = 0.041)	0.67 (0.47–0.95, *p* = 0.025)
Pre-existing conditions migraine	No	383 (32.4)	800 (67.6)	-	-
	Yes	39 (28.1)	100 (71.9)	1.23 (0.84–1.83, *p* = 0.302)	1.37 (0.89–2.14, *p* = 0.156)
Pre-existing conditions irritable bowel syndrome IBS	No	369 (30.6)	838 (69.4)	-	-
	Yes	53 (46.1)	62 (53.9)	0.52 (0.35–0.76, *p* = 0.001)	0.60 (0.39–0.92, *p* = 0.018)
BMI	Mean (SD)	26.4 (6.0)	24.7 (5.2)	0.95 (0.93–0.97, *p* < 0.001)	0.98 (0.95–1.00, *p* = 0.053)
Aspirin	No	294 (30.6)	668 (69.4)	-	-
	Yes	128 (35.6)	232 (64.4)	0.80 (0.62–1.03, *p* = 0.083)	0.69 (0.45–1.05, *p* = 0.082)
Paracetamol	No	262 (31.1)	580 (68.9)	-	-
	Yes	160 (33.3)	320 (66.7)	0.90 (0.71–1.15, *p* = 0.406)	0.86 (0.66–1.13, *p* = 0.270)
Ibuprofen	No	353 (32.3)	740 (67.7)	-	-
	Yes	69 (30.1)	160 (69.9)	1.11 (0.81–1.51, *p* = 0.523)	1.66 (1.00–2.75, *p* = 0.049)
Antioxidants	No	393 (32.3)	823 (67.7)	-	-
	Yes	29 (27.4)	77 (72.6)	1.27 (0.82–2.00, *p* = 0.294)	1.69 (1.02–2.89, *p* = 0.048)
Anti-type two histamine	No	394 (32.9)	802 (67.1)	-	-
	Yes	28 (22.2)	98 (77.8)	1.72 (1.13–2.71, *p* = 0.015)	1.67 (1.05–2.73, *p* = 0.035)
Omega 3	No	366 (30.9)	820 (69.1)	-	-
	Yes	56 (41.2)	80 (58.8)	0.64 (0.44–0.92, *p* = 0.015)	0.71 (0.48–1.07, *p* = 0.100)
Azithromycin	No	248 (29.6)	589 (70.4)	-	-
	Yes	174 (35.9)	311 (64.1)	0.75 (0.59–0.95, *p* = 0.019)	0.74 (0.57–0.96, *p* = 0.025)
Steroids	No	313 (28.8)	773 (71.2)	-	-
	Yes	109 (46.2)	127 (53.8)	0.47 (0.35–0.63, *p* < 0.001)	0.73 (0.52–1.03, *p* = 0.071)

** Subgroup analysis revealed that vaccination acutely and significantly decreased the symptoms duration by 59.1% vs. 74.1% (*p*-value < 0.001).

### 3.6 Infection after vaccination and associated symptoms

Among 2,337 participants, 734 (31.4%) were unvaccinated, 1,050 (44.9%) were vaccinated without reinfection, and 553 (23.7%) were vaccinated but reinfected. Depression was more common in the unvaccinated (50.3%) and reinfection (48.6%) groups than in the non-reinfection group (44.4%) (*p* = 0.037). Long-lasting symptoms (>2 weeks) were most frequent in the unvaccinated (78.7%), followed by non-reinfection (69.5%) and reinfection (59.1%) groups (*p* < 0.001). Chronic fatigue was highest in the reinfection group (9.4%) (*p* < 0.001) (Table 3, Supplementary Table S7).

**Table 3:** Infection after vaccination univariate comparison.

Infection after vaccination	Not vaccinated	Infected Before, No Reinfection After	Infection after vaccination	*p*	SMD
n	734	1,050	553		
Depression = Yes (%)	369 (50.3)	466 (44.4)	269 (48.6)	0.037	0.079
Symptom’s duration = more than 2 weeks (%)	578 (78.7)	730 (69.5)	327 (59.1)	<0.001	0.288
Chronic fatigue = Yes (%)	54 (7.4)	41 (3.9)	52 (9.4)	<0.001	0.149

Multivariable analysis linked reinfection risk to anger (OR 1.33, *p* = 0.051), insomnia (OR 1.44, *p* = 0.043), chest pain (OR 1.58, *p* = 0.009), joint pain (OR 1.46, *p* = 0.005), abdominal pain (OR 1.53, *p* = 0.004), and chronic fatigue (OR 2.32, *p* < 0.001). Lower reinfection risk was associated with irritability (OR 0.59, *p* = 0.003), fragmented sleep (OR 0.70, *p* = 0.042), sore throat (OR 0.79, *p* = 0.075), and muscle aches (OR 0.59, *p* < 0.001) (Supplementary Table S8).

### 3.7 Risk factors for long infection duration

The multivariate model identified factors linked to prolonged symptoms (>2 weeks). Increased risk was associated with vaccination (OR 1.58, *p* < 0.001), migraine (OR 1.48, *p* = 0.036), and naproxen use (OR 1.74, *p* = 0.002). Lower risk was seen with pre-infection vertigo (OR 0.68, *p* = 0.002), hospitalization (OR 0.36, *p* < 0.001), anaemia (OR 0.62, *p* = 0.003), hypertension (OR 0.62, *p* = 0.003), lower BMI (OR 0.97, *p* = 0.006), aspirin (OR 0.61, *p* = 0.001), azithromycin (OR 0.71, *p* = 0.001), and steroids (OR 0.71, p = 0.004). No significant association was found for paracetamol, ibuprofen, antioxidants, type-2 antihistamines, omega-3, or IBS (Table 4).

**Table 4:** Logistic regression model for symptoms duration; AIC = 2661.6, C-statistic = 0.689, H&L = Chi-sq (8) 7.12 (*p* = 0.524).

Dependent: symptoms duration		<2 weeks	>2 weeks	OR (univariable)	OR (multivariable)
Sex	Female	422 (31.9)	900 (68.1)	-	-
	Male	280 (27.6)	735 (72.4)	1.23 (1.03–1.47, *p* = 0.024)	1.19 (0.98–1.46, *p* = 0.080)
Before COVID vertigo dizziness	No	560 (28.3)	1,416 (71.7)	-	-
	Yes	142 (39.3)	219 (60.7)	0.61 (0.48–0.77, *p* < 0.001)	0.68 (0.53–0.87, *p* = 0.002)
Hospitalization	No	549 (26.5)	1,521 (73.5)	-	-
	Yes	153 (57.3)	114 (42.7)	0.27 (0.21–0.35, *p* < 0.001)	0.36 (0.27–0.48, *p* < 0.001)
COVID 19 vaccination	No	226 (40.9)	327 (59.1)	-	-
	Yes	476 (26.7)	1,308 (73.3)	1.90 (1.56–2.32, *p* < 0.001)	1.58 (1.27–1.95, *p* < 0.001)^**^
Pre-existing conditions anaemia	No	612 (29.0)	1,497 (71.0)	-	-
	Yes	90 (39.5)	138 (60.5)	0.63 (0.47–0.83, *p* = 0.001)	0.62 (0.46–0.85, *p* = 0.003)
Pre-existing conditions hypertension high blood pressure	No	595 (28.0)	1,527 (72.0)	-	-
	Yes	107 (49.8)	108 (50.2)	0.39 (0.30–0.52, *p* < 0.001)	0.62 (0.45–0.85, *p* = 0.003)
Pre-existing conditions migraine	No	651 (30.2)	1,504 (69.8)	-	-
	Yes	51 (28.0)	131 (72.0)	1.11 (0.80–1.57, *p* = 0.537)	1.48 (1.03–2.16, *p* = 0.036)
Pre-existing conditions irritable bowel syndrome (IBS)	No	637 (29.3)	1,535 (70.7)	-	-
	Yes	65 (39.4)	100 (60.6)	0.64 (0.46–0.89, *p* = 0.007)	0.73 (0.52–1.04, *p* = 0.082)
Pre-existing conditions mould	No	605 (29.5)	1,446 (70.5)	-	-
	Yes	97 (33.9)	189 (66.1)	0.82 (0.63–1.06, *p* = 0.127)	0.77 (0.58–1.02, *p* = 0.063)
BMI	Mean (SD)	26.3 (5.4)	25.0 (4.9)	0.95 (0.94–0.97, *p* < 0.001)	0.97 (0.96–0.99, *p* = 0.006)
Aspirin	No	470 (28.1)	1,201 (71.9)	-	-
	Yes	232 (34.8)	434 (65.2)	0.73 (0.60–0.89, *p* = 0.001)	0.61 (0.46–0.83, *p* = 0.001)
Naproxen	No	581 (30.4)	1,328 (69.6)	-	-
	Yes	121 (28.3)	307 (71.7)	1.11 (0.88–1.40, *p* = 0.378)	1.74 (1.22–2.49, *p* = 0.002)
Azithromycin	No	404 (27.2)	1,080 (72.8)	-	-
	Yes	298 (34.9)	555 (65.1)	0.70 (0.58–0.84, *p* < 0.001)	0.71 (0.59–0.86, *p* = 0.001)
Steroids	No	505 (26.8)	1,378 (73.2)	-	-
	Yes	197 (43.4)	257 (56.6)	0.48 (0.39–0.59, *p* < 0.001)	0.71 (0.56–0.90, *p* = 0.004)

** Subgroup analysis revealed that vaccination acutely significantly decreased the symptoms duration 59.1% vs. 74.1% (p-value < 0.001) infected after vaccination vs. infected before vaccination or did not receive vaccination, respectively.

### 3.8 Symptoms associated with prolonged infection duration

A runny nose (OR 1.25, *p* = 0.044) increased the odds of prolonged symptoms (>2 weeks) by 25%, while other symptoms, including brain fog, anxiety, anger, loss of smell, tachycardia, loss of appetite, shortness of breath, dry cough, and abdominal pain, reduced the odds by 22 to 40% (Supplementary Table S9).

### 3.9 CFS risk factors

CFS was associated with mental health disorders (OR 2.11, *p* = 0.001), vertigo/dizziness (OR 2.73, *p* < 0.001), smoking (OR 2.39, *p* = 0.005), hospitalization (OR 2.41, *p* < 0.001), vitamin D deficiency (OR 2.74, *p* < 0.001), asthma (OR 2.01, *p* = 0.013), diabetes type 2 (OR 2.45, *p* = 0.001), nightmares (OR 2.51, *p* = 0.001), and paracetamol use (OR 1.49, *p* = 0.029), while male sex (OR 0.64, *p* = 0.040), good pre-COVID health (OR 0.64, *p* = 0.023), and anti-type one histamines (OR 0.52, *p* = 0.024) reduced the risk (Supplementary Table S10).

### 3.10 Symptoms associated with chronic fatigue

Chronic fatigue was associated with brain fog (OR 1.52, *p* = 0.045), anxiety (OR 1.62, *p* = 0.028), depression (OR 2.41, *p* < 0.001), insomnia (OR 2.10, *p* < 0.001), palpitations (OR 1.93, *p* = 0.002), loss of appetite (OR 1.83, p = 0.002), joint pain (OR 1.53, *p* = 0.036), and muscle aches (OR 1.51, *p* = 0.041), while altered smell reduced the risk (OR 0.48, *p* = 0.019) (Supplementary Table S11).

### 3.11 Depression risk factors

Depression risk increased with age 40–49 (OR 1.43, *p* = 0.032), healthcare work (OR 1.24, *p* = 0.034), poor pre-COVID health (OR 2.27, *p* < 0.001), prior mental health diagnosis (OR 3.90, *p* < 0.001), tinnitus (OR 1.88, *p* = 0.001), vertigo (OR 2.54, *p* < 0.001), smoking (OR 1.41, *p* = 0.027), oxygen support (OR 2.30, *p* < 0.001), anemia (OR 1.40, *p* = 0.041), diabetes (OR 1.95, *p* = 0.001), migraine (OR 1.55, *p* = 0.016), IBS (OR 2.03, *p* < 0.001), insomnia (OR 1.74, *p* = 0.001), nightmares (OR 1.79, *p* = 0.018), aspirin (OR 1.70, *p* = 0.001), and omega-3 (OR 1.53, *p* = 0.010). Male sex (OR 0.64, *p* < 0.001), excellent pre-COVID health (OR 0.68, *p* < 0.001), vision problems (OR 0.70, *p* = 0.026), paracetamol (OR 0.76, *p* = 0.006), and anti-H2 histamines (OR 0.50, *p* < 0.001) lowered risk (Supplementary Table S12).

### 3.12 Symptoms associated with depression

Depression risk increased with migraine (OR 1.47, *p* = 0.003), tinnitus (OR 1.47, *p* = 0.039), vertigo (OR 1.49, *p* = 0.003), brain fog (OR 2.21, *p* < 0.001), depression (OR 2.22, *p* < 0.001), anger (OR 1.38, *p* = 0.020), emotional instability (OR 2.12, *p* < 0.001), insomnia (OR 1.50–2.65, *p* ≤ 0.001), tachycardia (OR 1.43, *p* = 0.013), and abdominal pain (OR 1.44, p = 0.010). Reproductive/urinary symptoms (OR 0.63, *p* = 0.009) and sore throat (OR 0.80, *p* = 0.042) lowered risk (Supplementary Table S13).

### 3.13 Summary of clustering and latent feature analysis

#### 3.13.1 HDBSCAN and PCA

HDBSCAN failed to identify meaningful clusters, classifying most patients as noise, while PCA revealed three principal components explaining 30% of variance but lacking clear separation (Supplementary Tables S14–S16, Supplementary Figures S3–S6).

#### 3.13.2 Neural network latent features

Deep learning models (SCARF & encoder bottleneck) showed strong correlations among outcome variables, suggesting shared underlying features rather than distinct subgroups. SCARF’s first latent feature correlated positively with all outcomes except reproductive and urinary symptoms, with a high similarity to encoder outputs (R^2^ = 0.5), reinforcing the multisystemic nature of COVID-19 (Supplementary Tables S17, S18).

#### 3.13.3 Encoder bottleneck layer analysis

The model fit was reasonable (AIC = 26,190.1, R^2^ = 0.35). Factors positively associated with encoder values included pre-COVID tinnitus, vertigo, dizziness, hospitalization, and insomnia, while male sex, antihistamine use, and vitamin D deficiency had negative associations (Supplementary Table S18).

#### 3.13.4 SCARF embeddings analysis

The model explained 40% of variance (AIC = 4,483.7). Pre-COVID tinnitus, vertigo, and hospitalization were positively associated with embeddings, whereas antihistamine use and mood-related irritability had negative associations (Supplementary Table S20).

### 3.14 Prediction models summary

The AutoML algorithm selected the GBM model as the best performer.

#### 3.14.1 Chronic fatigue

High AUC (0.87) but low accuracy (0.73), worse than NIR (0.94). High PPV (0.99), moderate sensitivity (0.72), and high specificity (0.90), but low NPV (0.18) and kappa (0.22), with significant false positive/negative imbalance.

#### 3.14.2 Depression

High AUC (0.82) and accuracy (0.76), outperforming NIR (0.52). Strong PPV (0.76), NPV (0.77), sensitivity (0.80), and moderate specificity (0.73) and kappa (0.52), with balanced false positives/negatives.

#### 3.14.3 Symptom duration

**Figure 3:**
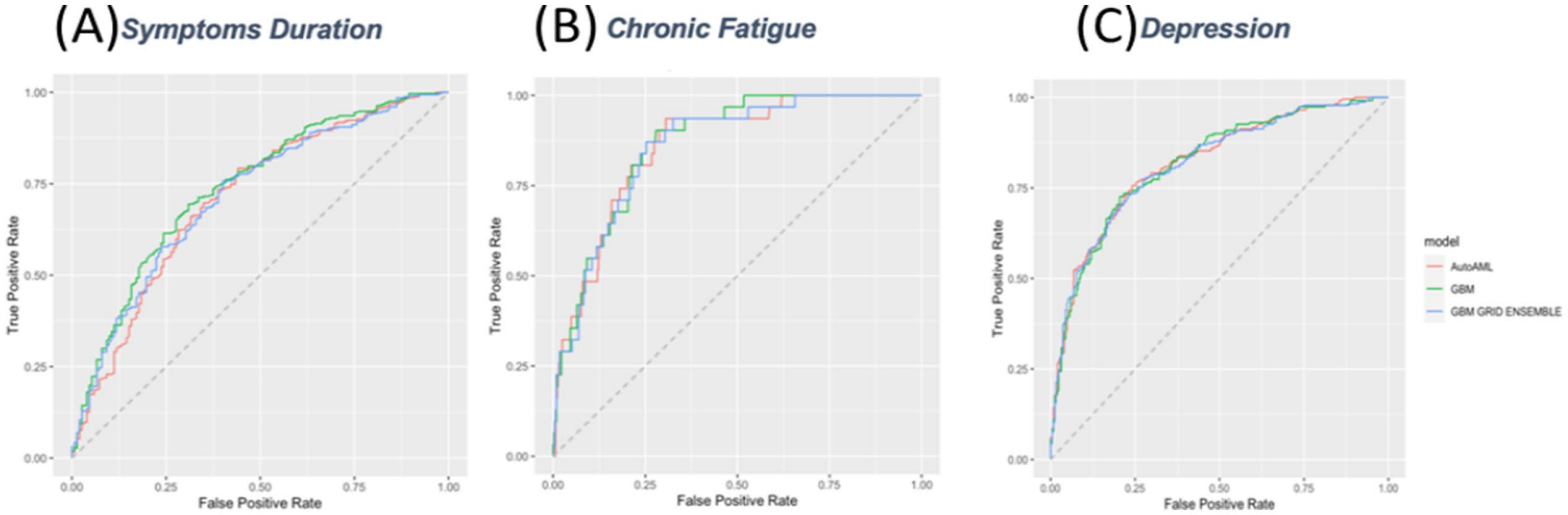
Receiver Operating Characteristic (ROC) Curves for Prediction Models. ROC curves for three predictive models evaluating **(A)** symptom duration, **(B)** chronic fatigue, and **(C)** depression. The models compared include AutoML (red), GBM (green), and GBM grid ensemble (blue). The x-axis represents the false positive rate, and the y-axis represents the true positive rate.

Moderate AUC (0.74) and accuracy (0.69), similar to NIR (0.68). Low PPV (0.51), high NPV (0.83), moderate sensitivity (0.69), specificity (0.69), and kappa (0.35), with notable false positive/negative imbalance (Figure 3).

Supplementary Tables S22–S24 provide scaled variable importance for predicting depression, symptom duration, and chronic fatigue.

### 3.15 Model deployment

The best-performing models are then implemented in a shiny app and deployed online at https://ahmedshaheen.shinyapps.io/shaheen-covid/.

## 4 Discussion

We analyzed 2,445 patients, with 43.5% males, primarily from Egypt, Pakistan, India, and Syria, and most aged 40–49 years. Non-smokers constituted 79.7, and 35.7% were healthcare professionals. Pre-existing conditions included gastrointestinal, hematological, respiratory, neurological, and allergic disorders. Among the sample, 12.4% were hospitalized, 75.8% were vaccinated, and common treatments included paracetamol (35.6%), aspirin (29.0%), and azithromycin (36.9%). Key symptoms included loss of smell (46.8%), dry cough (40.1%), and headaches (37.2%), lasting an average of 13.6 days. Reinfection after vaccination occurred in 23.7% and was associated with higher depression and chronic fatigue rates. Prolonged symptoms (>2 weeks) were linked to vaccination, migraine, and naproxen use, while factors such as hospitalization, hypertension, and steroid use reduced symptom duration. Specific symptoms and pre-existing conditions influenced the risk of chronic fatigue and prolonged infection duration. Detailed statistical analyses are available in the Supplementary material.

These findings are consistent with previous studies that have reported similar symptom profiles in long COVID patients (30–39).

Our analysis revealed several interesting associations. The prevalence of migraines (18.0%) in our cohort was higher than the estimated 10–15% in the general population (23), consistent with emerging research linking COVID-19 to an increased risk of migraines (24). While the exact mechanisms remain unclear, they may involve inflammatory, vascular, or neurological factors triggered by the viral infection, as suggested by recent neuroimaging studies (40, 41).

We also highlighted the substantial impact of COVID-19 on the genitourinary system, with 90.3% of participants reporting reproductive and urinary symptoms. This finding aligns with recent reports of alterations in menstrual cycles, sexual function, and urinary tract infections in COVID-19 patients (42, 43). The potential mechanisms, such as hormonal imbalance, immune dysregulation, endothelial damage, or direct viral invasion, warrant further investigation. Intriguingly, our results suggest that normal/regular menstrual cycles were associated with prolonged COVID-19 symptoms (> 2 weeks). This unexpected finding could be attributed to various factors, including hormonal fluctuations influencing immune responses (44, 45).

However, the lack of significant effect of pregnancy on COVID-19 outcomes should be interpreted cautiously due to the small sample size of pregnant patients in our cohort, contrasting with some previous studies that found increased risks for pregnant women (28).

Our machine learning models identified several factors associated with prolonged symptoms, including vaccination status, migraine, and naproxen use. Conversely, factors such as vertigo or dizziness before infection, hospitalization, and certain medications were associated with a decreased risk of prolonged symptoms. These findings provide valuable insights for risk stratification and potential therapeutic approaches in managing long COVID, building upon previous predictive models developed by Sudra et al. (46) The neurological implications of long COVID are particularly noteworthy. Our findings found high rates of depression (47.7%) and chronic fatigue (6.5%), which may be linked to various mechanisms including neuroinflammation, disruption of neurotransmitter systems, autonomic dysfunction, and the psychological impact of persistent symptoms. These findings are in line with the evidence in the literature and emphasize the need for comprehensive care approaches that address both the physical and mental health aspects of the condition (47–54).

## 5 Limitations

Despite the strengths of our study, including its large sample size and diverse cohort, several limitations must be acknowledged. The cross-sectional design limits our ability to establish causal relationships, and the focus on patients with symptoms lasting at least 4 weeks may have excluded individuals with shorter-term or milder cases of long-term COVID-19. The reliance on self-reported data introduces the possibility of recall bias, and the overrepresentation of participants from certain countries may limit the generalizability of our findings. Self-reported symptom data is prone to recall bias, leading to inaccurate symptom reporting, and subjective assessment bias, as perceptions vary between individuals. Selection bias can skew results if certain groups are more likely to participate, while social desirability bias may cause underreporting of stigmatized behaviors. These limitations are common in long COVID research and highlight the need for longitudinal studies with diverse populations (42).

Handling missing data and rare variables also limits current analysis, and further *post hoc* analysis can be useful. Lack of an external validation dataset limits the generalizability of the model, and we recommend future research focusing on external validation. The calibration results of our predictive models, as indicated by Brier scores, revealed varying levels of performance across different outcomes. The model for fatigue demonstrated good predictive performance (Brier score = 0.08), while models for symptom duration (Brier score = 0.18) and depression (Brier score = 0.17) showed moderate accuracy.

These results are comparable to other predictive models in COVID-19 research (46) and highlight both the potential utility of predictive models in managing long COVID and the inherent challenges in predicting complex, multifactorial outcomes. Our use of advanced machine learning techniques, including SCARF and encoder neural networks, provided valuable insights into the latent features associated with long COVID. However, the inability of the HDBSCAN method to yield meaningful clusters highlights the complex and heterogeneous nature of long COVID symptoms, which may not conform to distinct, easily separable categories. This aligns with recent discussions in the field about the challenges of categorizing long COVID symptoms (55).

## 6 Conclusion

Our findings significantly advance our understanding of long COVID, particularly in the context of low- and middle-income countries. Further, they underscore the need for comprehensive, multidisciplinary approaches to managing long COVID, addressing both physical and mental health aspects. Future research should focus on external validation of our predictive models, longer-term follow-up of patients, and more in-depth exploration of the mechanisms underlying the observed associations. As the global community continues to grapple with the long-term consequences of the COVID-19 pandemic, studies like ours provide critical insights to inform clinical practice, public health strategies, and future research directions.

## Data Availability

The datasets presented in this study can be found in online repositories. The names of the repository/repositories and accession number(s) can be found in the article/Supplementary material.
