# Supplementary material for: Deep learning analysis of long COVID and vaccine impact in low- and middle-income countries (LMICs): development of a risk calculator in a multicentric study
Source: Front Public Health. 2025 Jun 26;13:1416273. doi: 10.3389/fpubh.2025.1416273 (PMC12240947; doi:10.3389/fpubh.2025.1416273)

Contents

[1. Supplementary figures 1](#_Toc190932975)

[2. Supplementary tables 13](#_Toc190932976)

[3. Machine learning: symptoms duration 43](#_Toc190932977)

[4. Machine learning: fatigue 73](#_Toc190932978)

[5. Machine learning: depression 104](#_Toc190932979)

# Supplementary figures


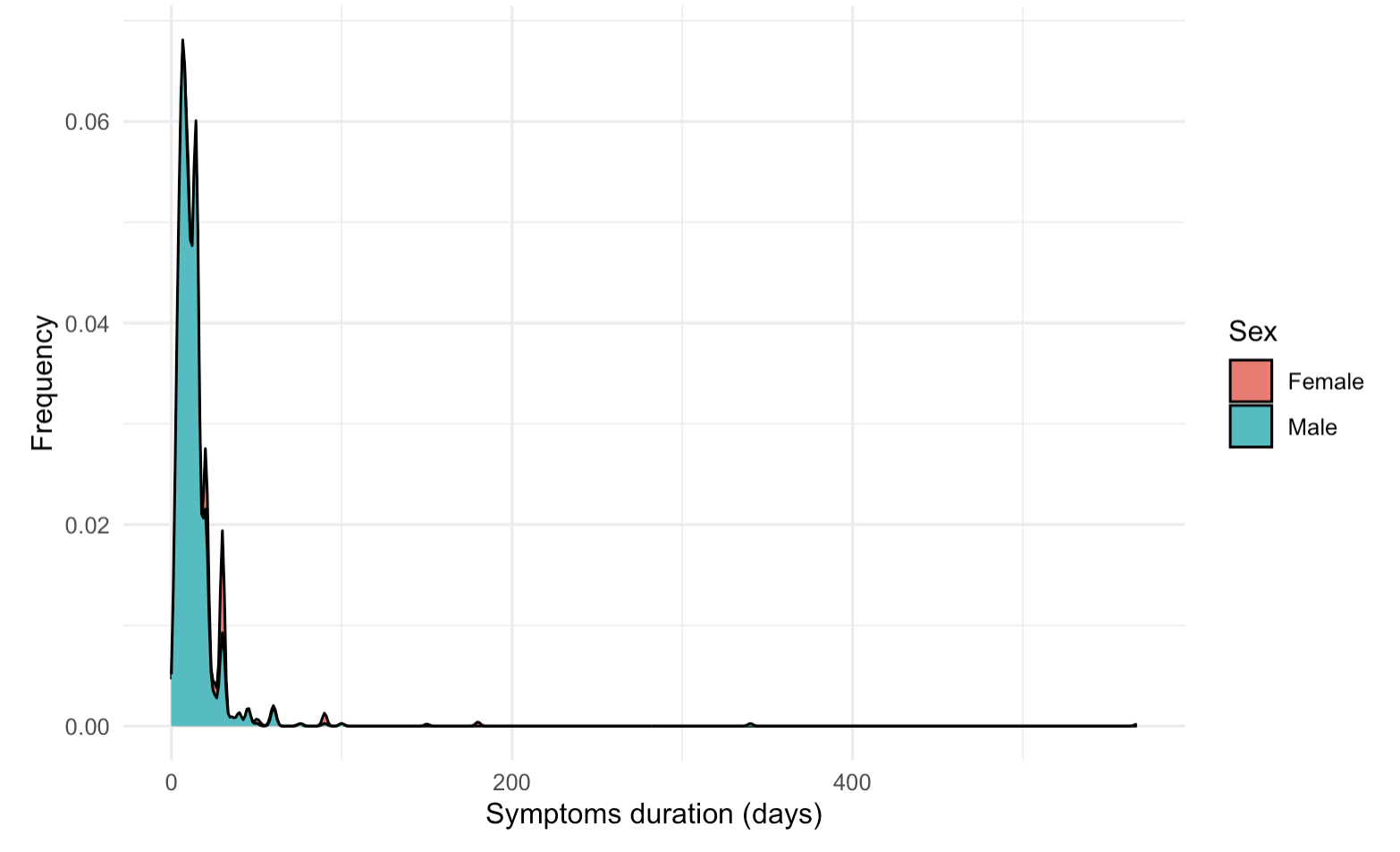


Figure 1: Symptoms duration distribution


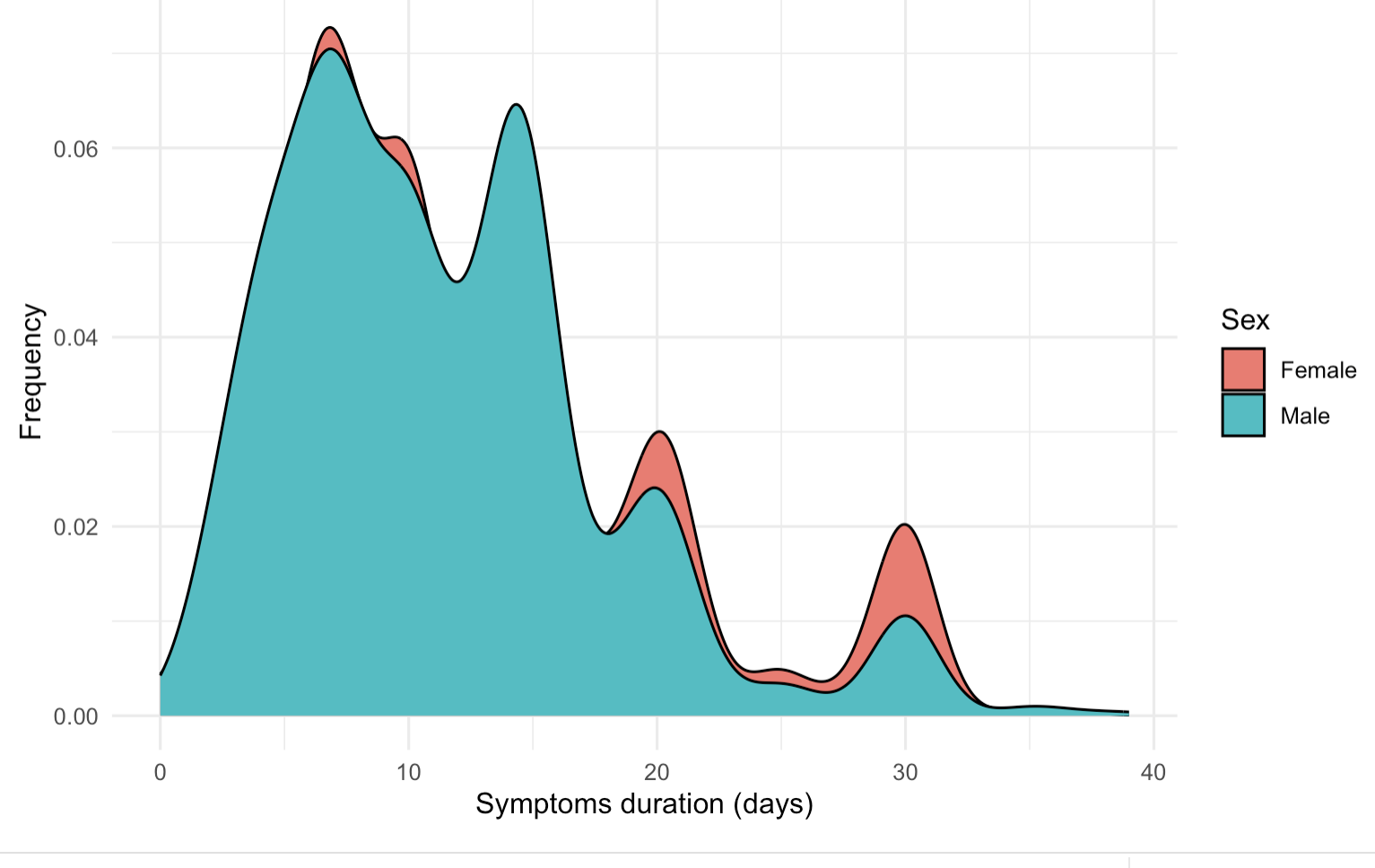


Figure 2: Symptoms duration after removal of outliers > 40 days


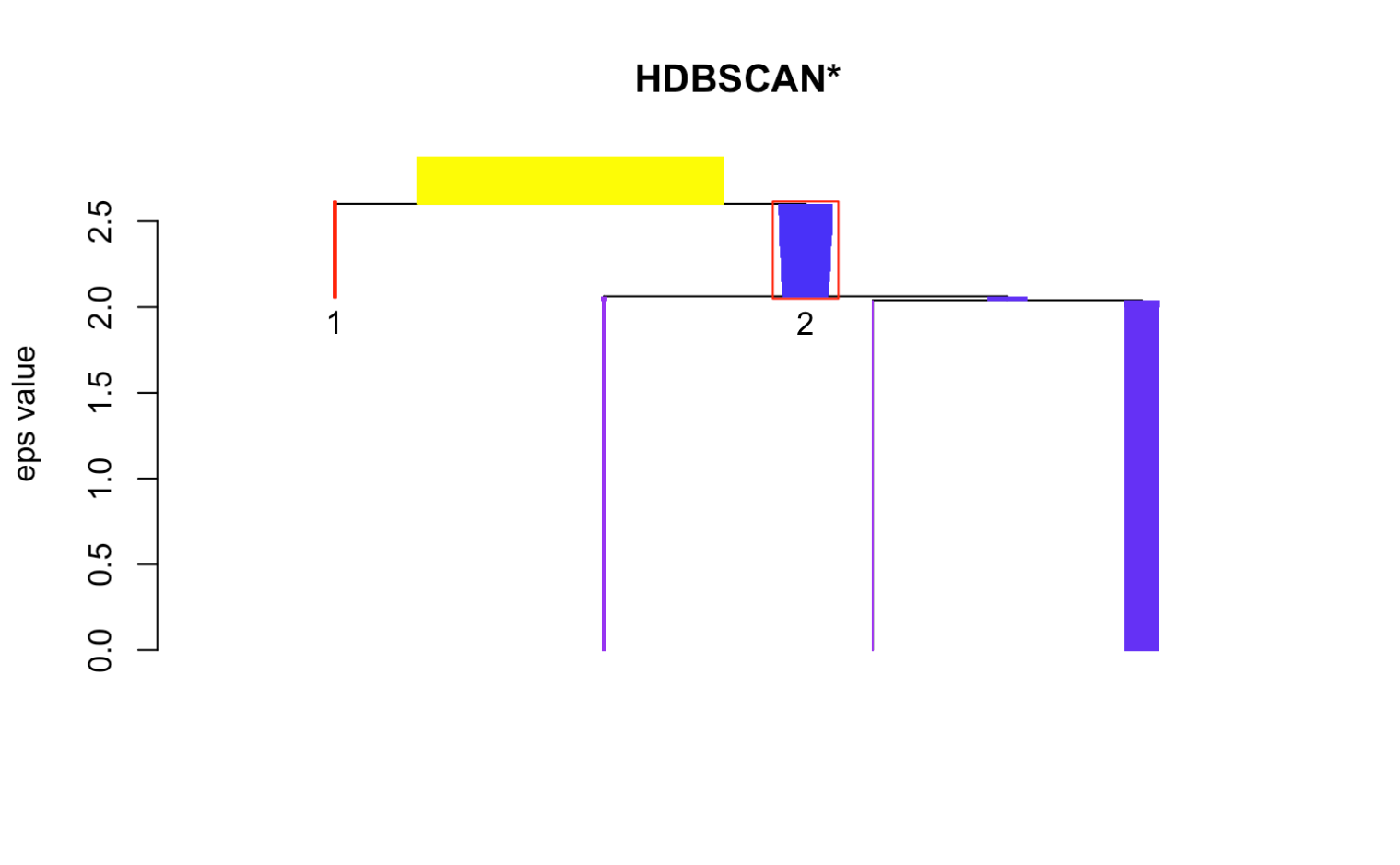


Figure 3: HDBSCAN Clustering


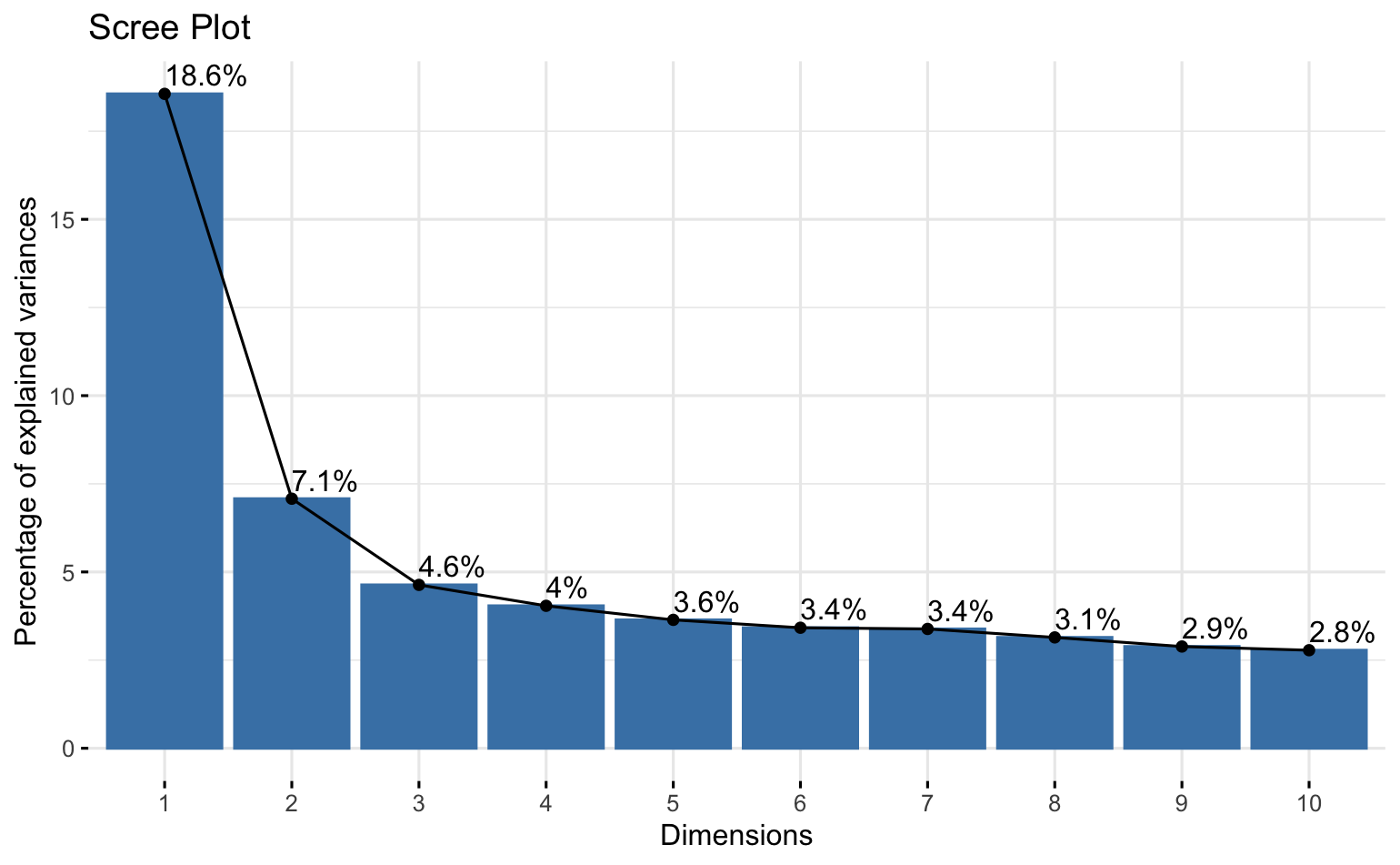


Figure 4: The elbow method shows that there are three components in the multidimensional outcome data.


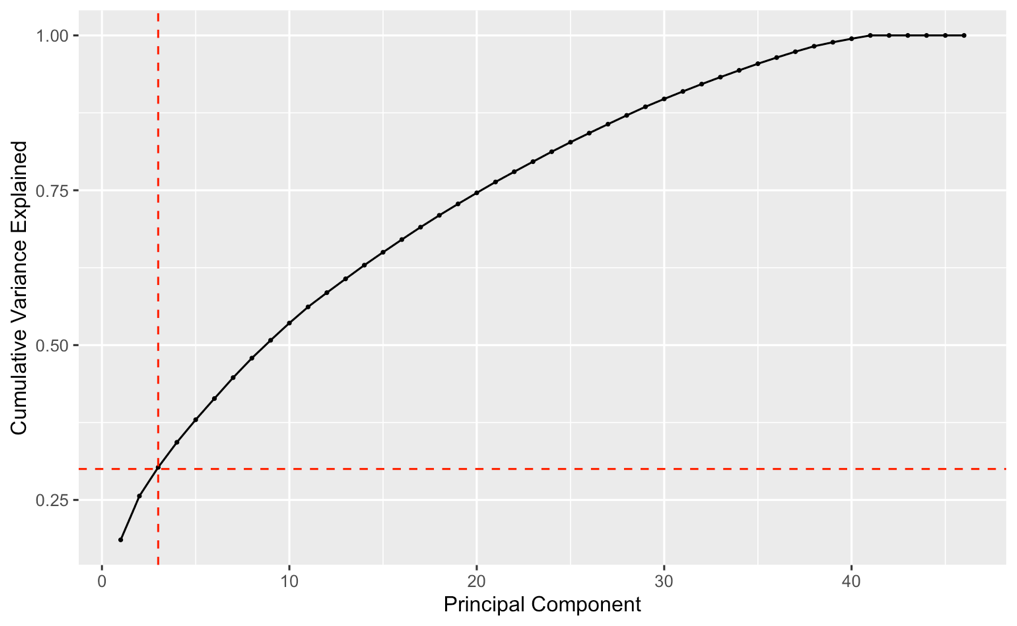


Figure 5: Cumulative variance explained by the components: the dashed red line is centered on the first three components


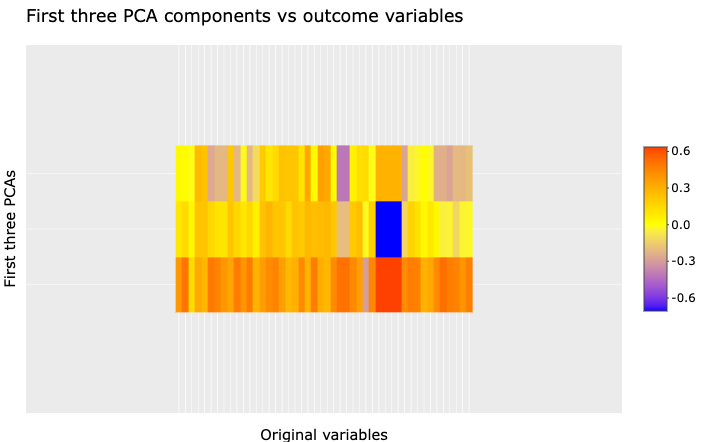


Figure 6: Correlation Matrix between PCA components vs original variables [(Interactive plot)](file:///C:\Users\ahmedshaheen\Desktop\Manuscript\PCA.html)


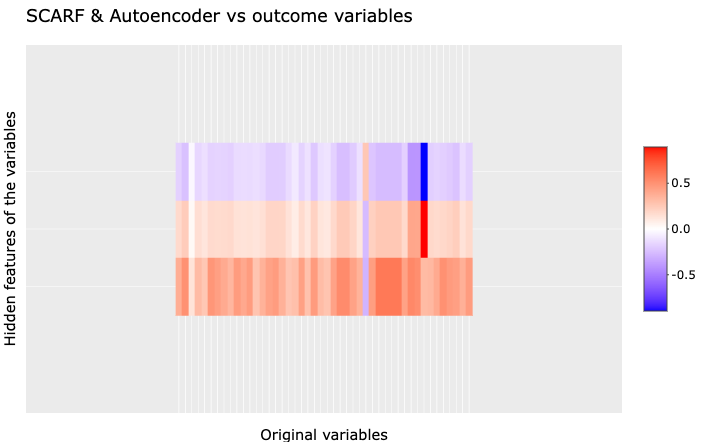


Figure 7: Correlation Matrix between the latent features of the neural networks’ vs original variables [(Interactive plot)](file:///C:\Users\ahmedshaheen\Desktop\Manuscript\Embeddings.html)


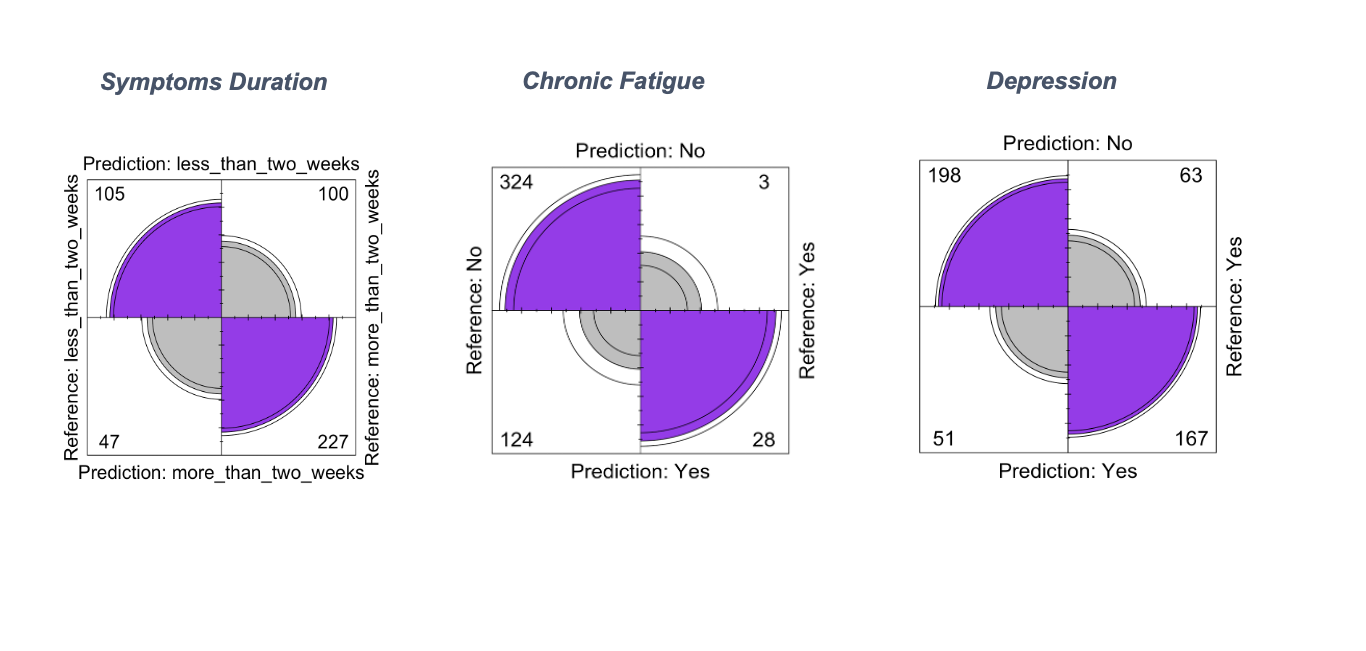


Figure 8: confusion matrix for models


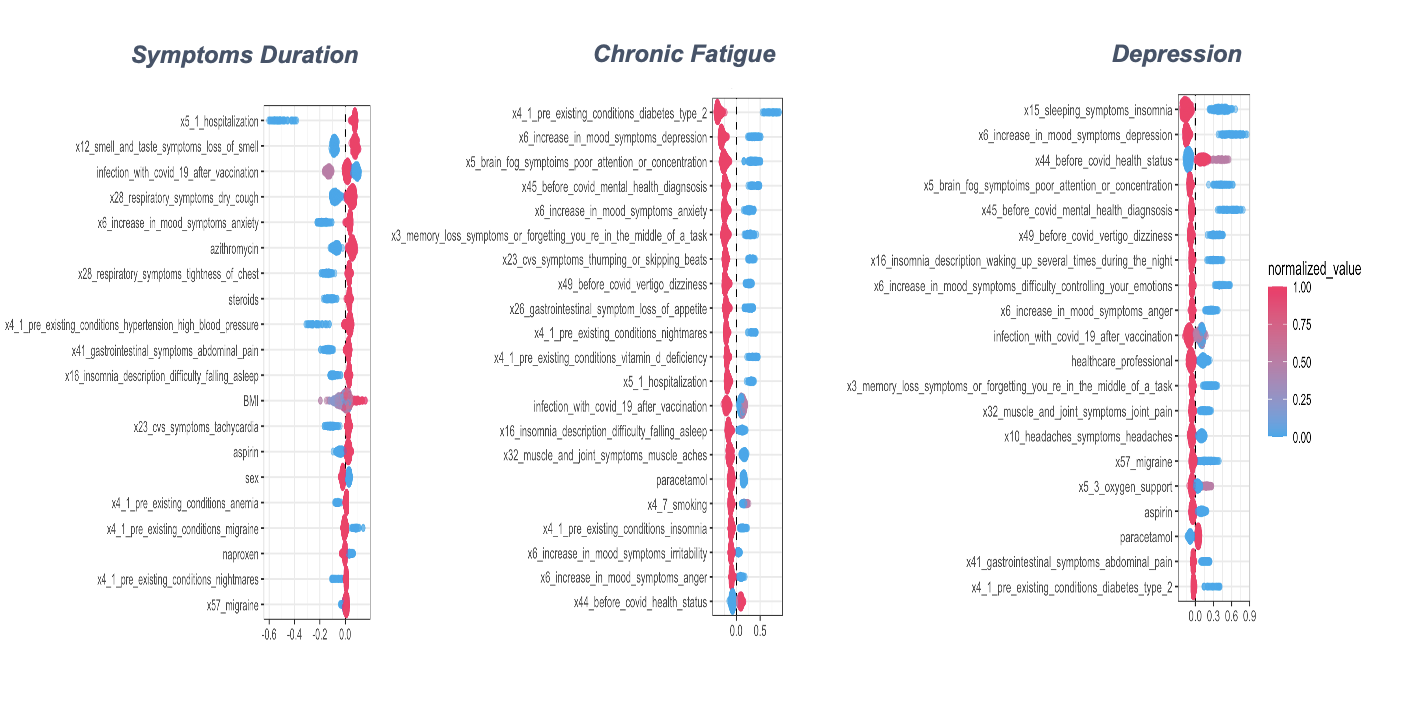


Figure 9: SHAP summary plot shows the contribution of the features for each instance (row of data). The sum of the feature contributions and the bias term is equal to the raw prediction of the model, i.e., prediction before applying inverse link function.


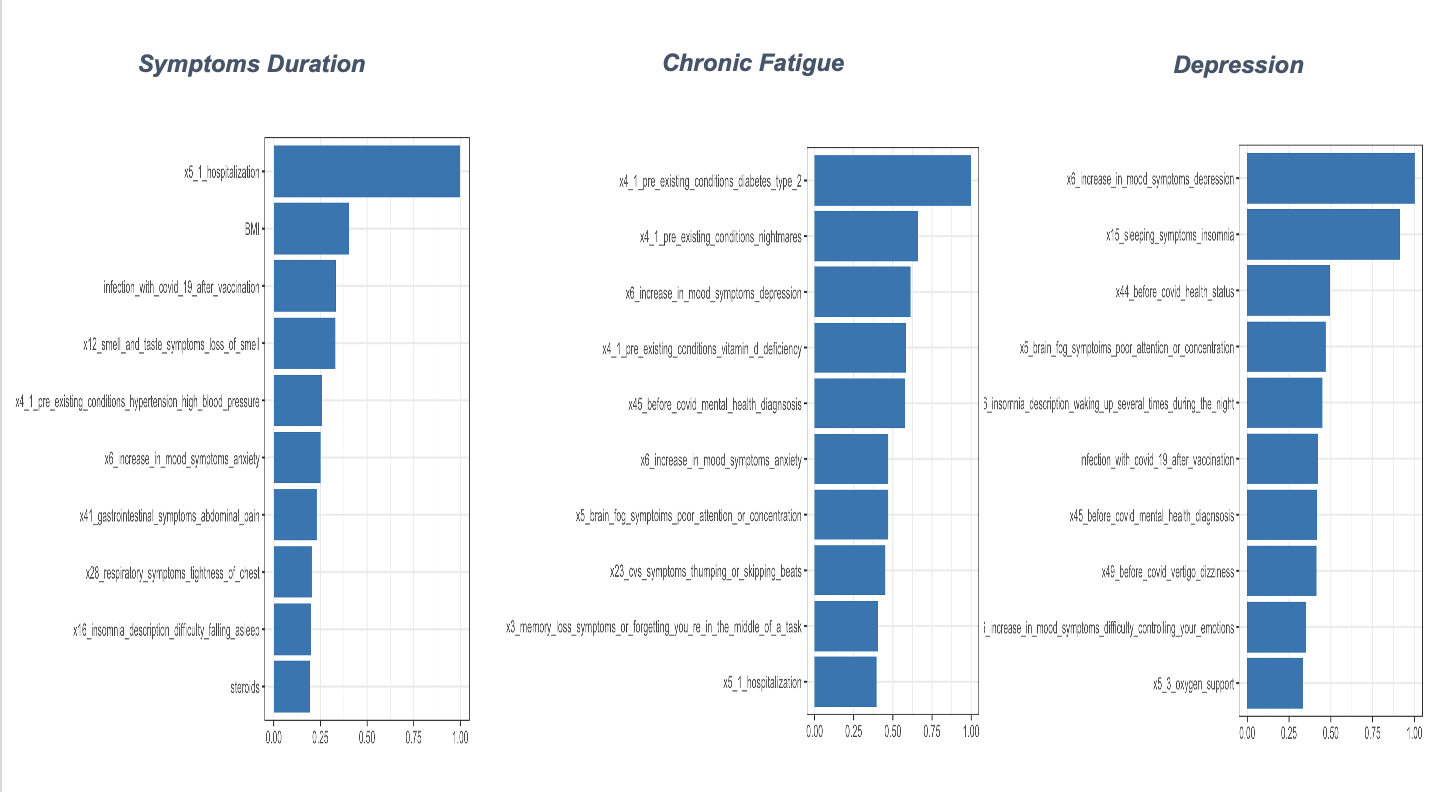


Figure 10: The variable importance plot shows the relative importance of the most important variables in the model


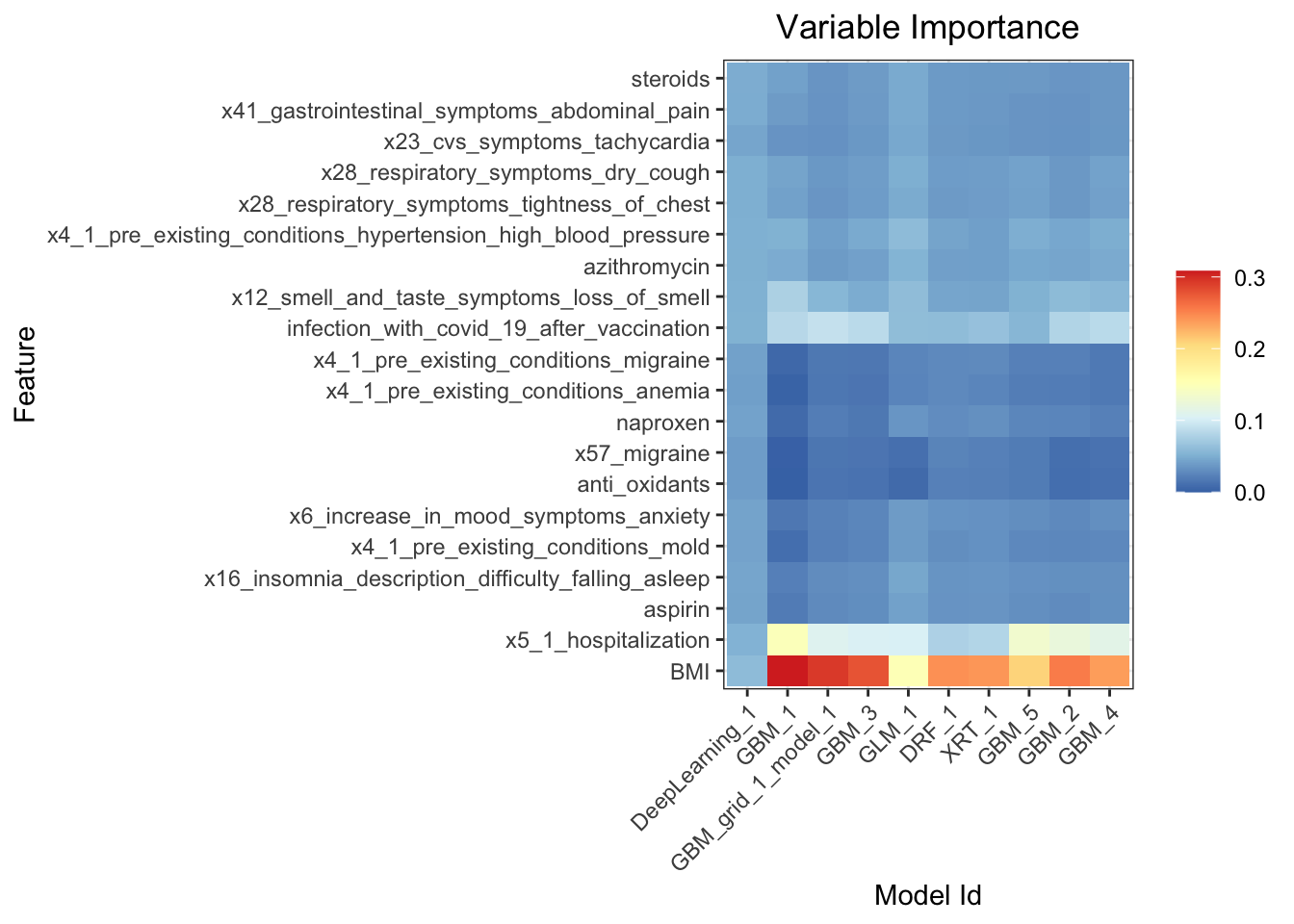


Figure 11: The variable importance matrix plot of the AUTOML models shows the relative importance of the most important variables in the models as a matrix for symptoms duration


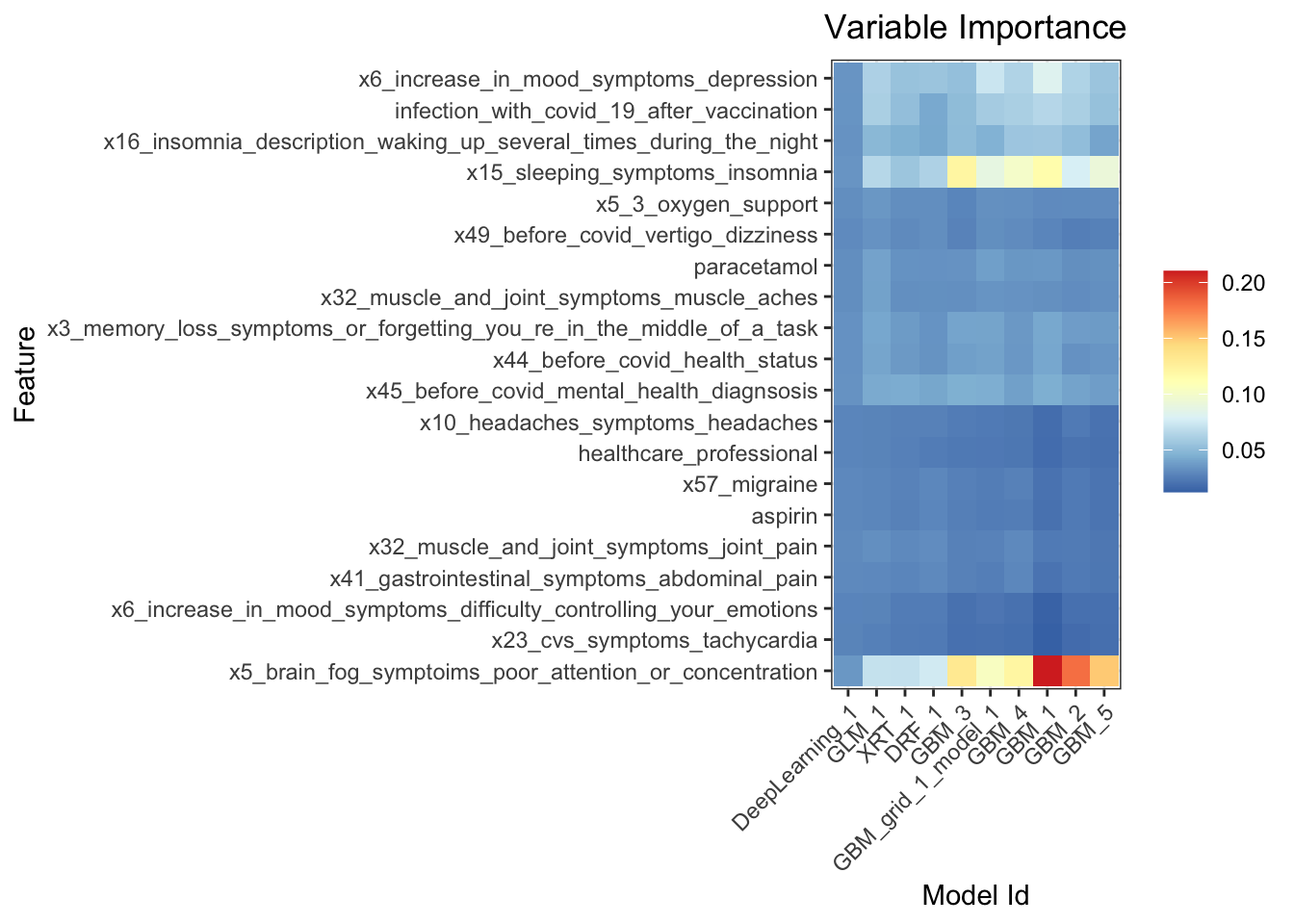


Figure 12: The variable importance matrix plot of the AUTOML models shows the relative importance of the most important variables in the models as a matrix for depression.


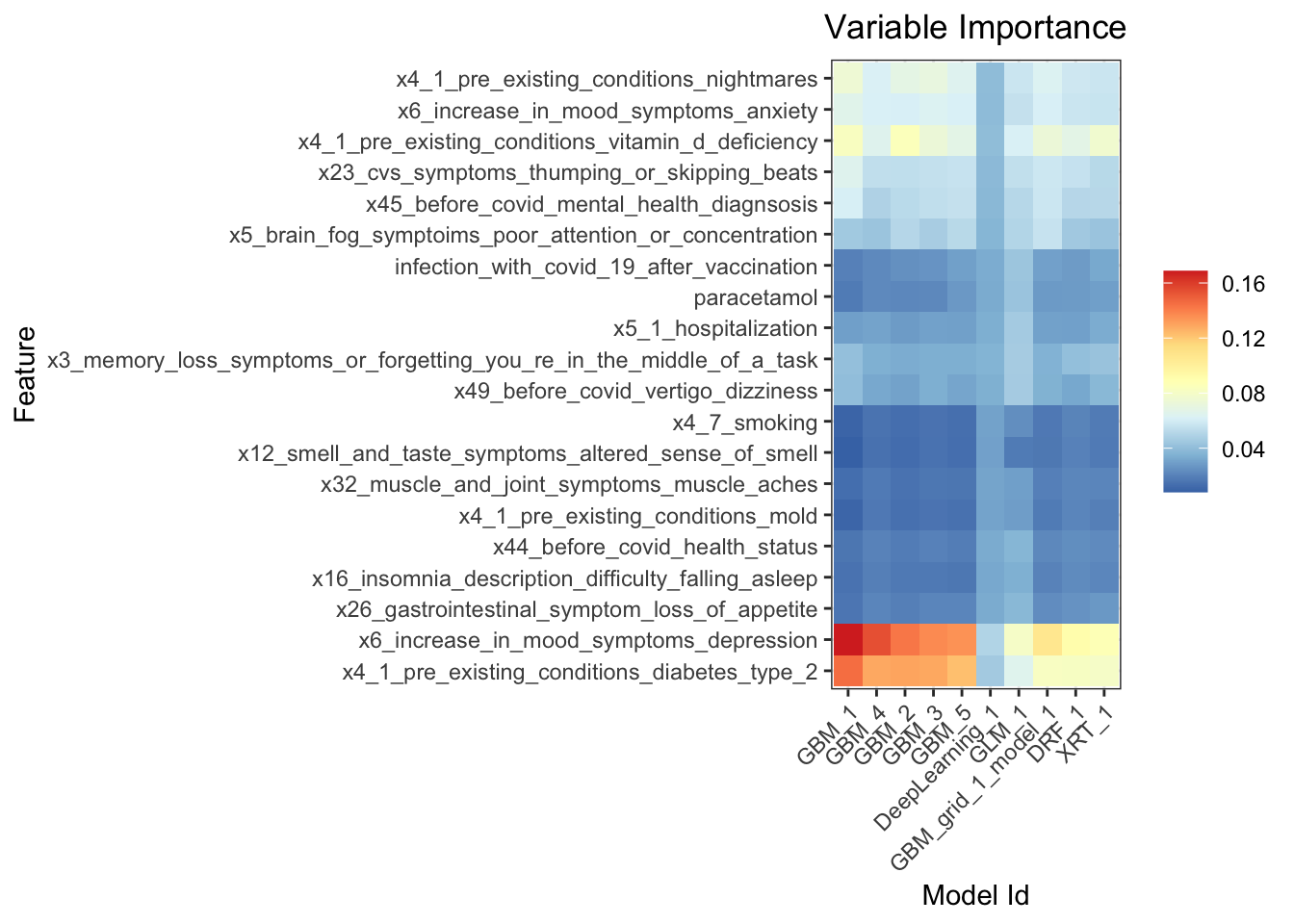


Figure 13: The variable importance matrix plot of the AUTOML models shows the relative importance of the most important variables in the models as a matrix for chronic fatigue

Figure 14-16: calibration plot


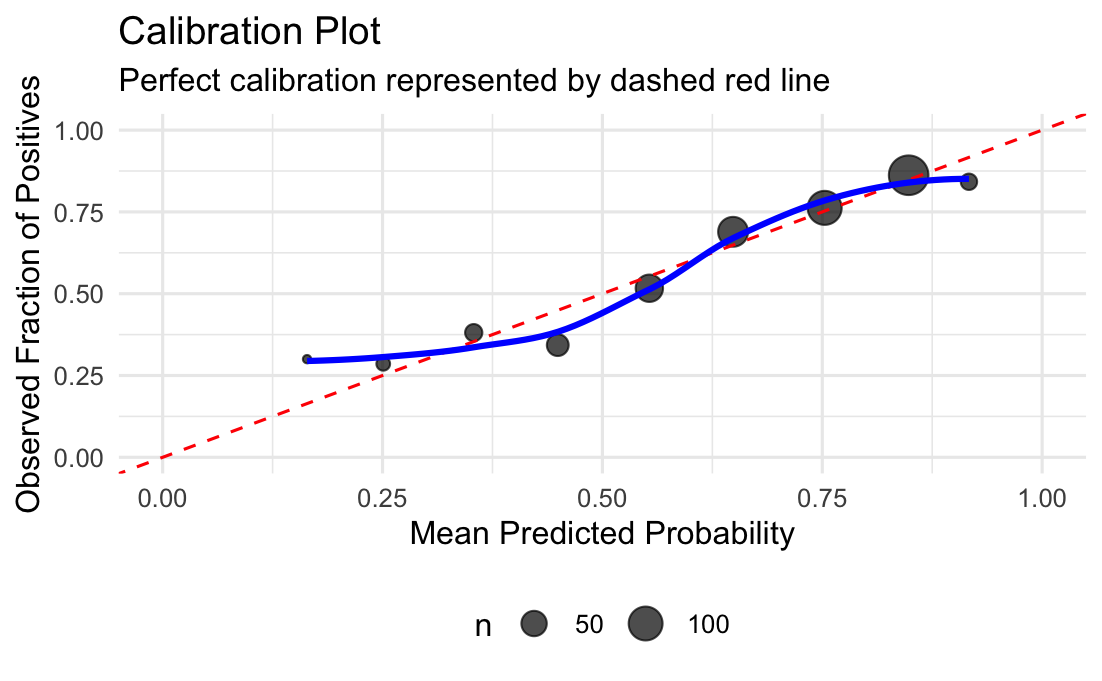


Symptoms Duration: Brier Score = 0.18


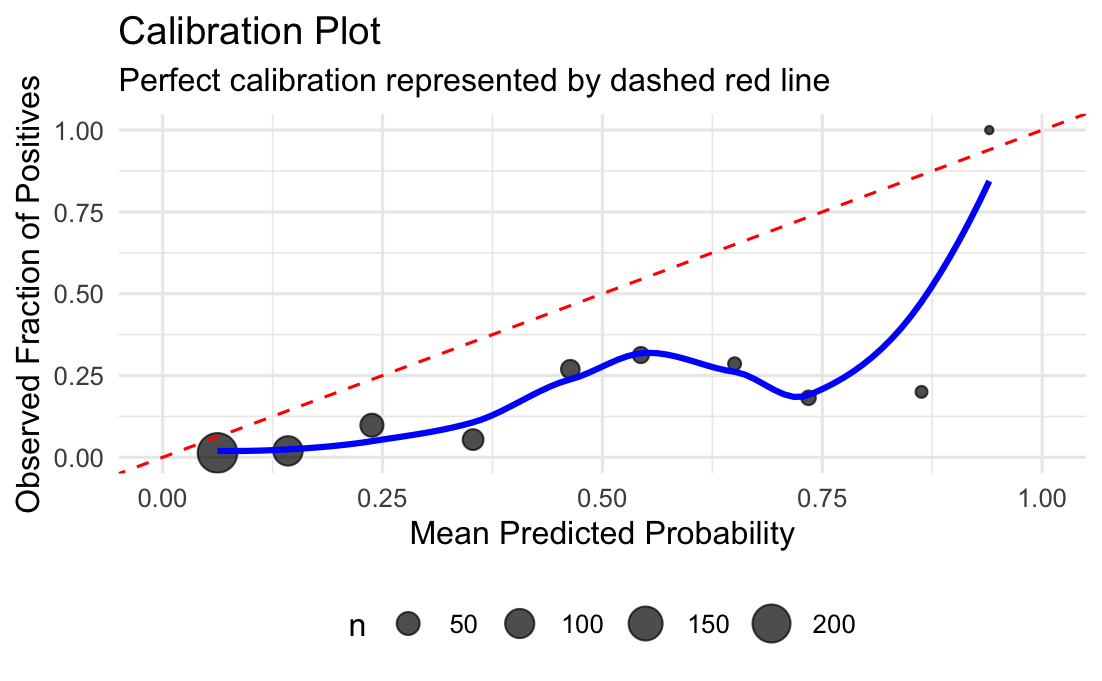


Fatigue: Barrier Score = 0.08


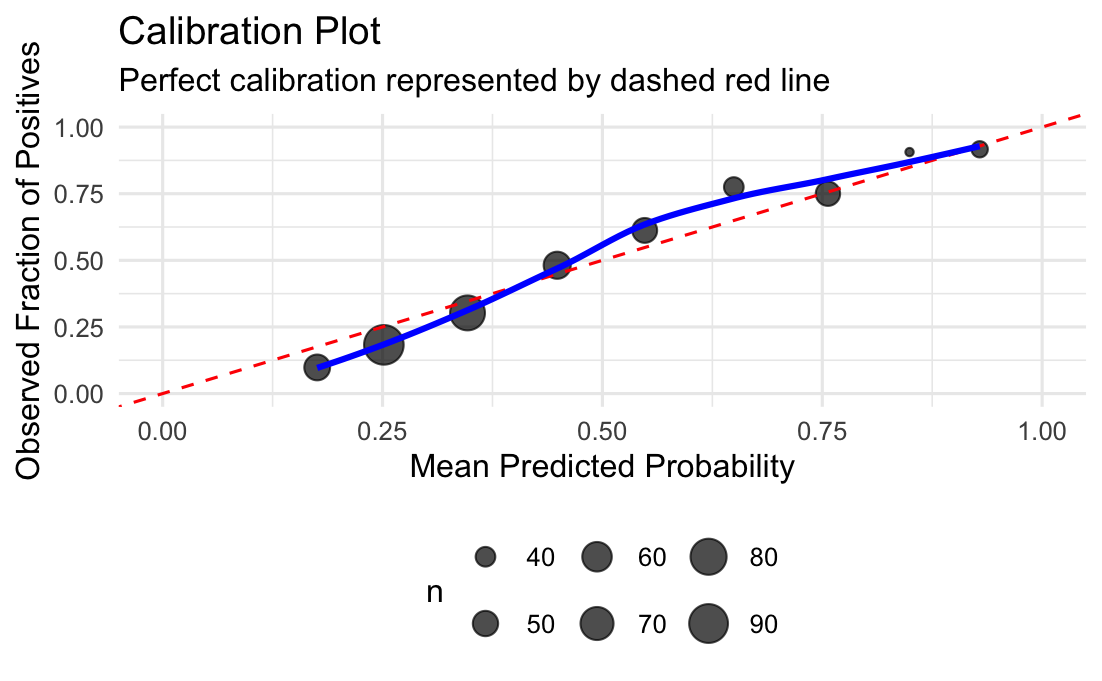


Depression: Brier Score = 0.17

# Supplementary tables

1. **Tables**

| **Medical History** | |
| --- | --- |
| Before covid health status (%) |  |
| Good | 750 (30.7) |
| Less than good | 207 (8.5) |
| More than good | 1488 (60.9) |
| Before covid mental health diagnsosis = Yes (%) | 235 (9.6) |
| Before covid tinnitus = 1 (%) | 198 (8.1) |
| Before covid vertigo/dizziness = Yes (%) | 384 (15.7) |
| Preexisting conditions: acid reflux disease = Yes (%) | 200 (8.2) |
| Preexisting conditions: anemia = Yes (%) | 243 (9.9) |
| Preexisting conditions: vision near-sighted/far-sighted = Yes (%) | 249 (10.2) |
| Preexisting conditions: vitamin-d deficiency = Yes (%) | 227 (9.3) |
| Preexisting conditions: asthma = Yes (%) | 156 (6.4) |
| Preexisting conditions: diabetes type two = Yes (%) | 168 (6.9) |
| Preexisting conditions: migraine = Yes (%) | 203 (8.3) |
| Preexisting conditions: hypertension = Yes (%) | 236 (9.7) |
| Preexisting conditions: environmental allergies dust = Yes (%) | 312 (12.8) |
| Preexisting conditions: mold infections = Yes (%) | 312 (12.8) |
| Preexisting conditions: food allergies = Yes (%) | 132 (5.4) |
| Preexisting conditions: allergies of unknown origin = Yes (%) | 94 (3.8) |
| Preexisting conditions: insomnia = Yes (%) | 241 (9.9) |
| Preexisting conditions: irritable bowel syndrome ibs = Yes (%) | 176 (7.2) |
| Preexisting conditions: restless leg syndrome = Yes (%) | 11 (0.4) |
| Preexisting conditions: conditions hepatitis a/b/c = Yes (%) | 18 (0.7) |
| Preexisting conditions: celiac disease = Yes (%) | 20 (0.8) |
| Preexisting conditions: other git issues = Yes (%) | 74 (3.0) |
| Preexisting conditions: vertigo = Yes (%) | 56 (2.3) |
| Preexisting conditions: eczema = Yes (%) | 77 (3.1) |
| Preexisting conditions: diabetes type 1 = Yes (%) | 26 (1.1) |
| Preexisting conditions: high cholesterol hyperlipidemia = Yes (%) | 75 (3.1) |
| Preexisting conditions: history of blood clotting = Yes (%) | 13 (0.5) |
| Preexisting conditions: hypotension low blood pressure = Yes (%) | 66 (2.7) |
| Preexisting conditions: mitral valve prolapse = Yes (%) | 15 (0.6) |
| Preexisting conditions: cancer all types = Yes (%) | 12 (0.5) |
| Preexisting conditions: auto immune rheumatological conditions = Yes (%) | 17 (0.7) |
| Preexisting conditions: night sweats = Yes (%) | 82 (3.4) |
| Preexisting conditions: sleep apnea = Yes (%) | 72 (2.9) |
| Preexisting conditions: nightmares = Yes (%) | 128 (5.2) |
| Preexisting conditions: lucid dreams dreams where you are  aware you are dreaming or have some control over what you dream = Yes (%) | 66 (2.7) |
| Preexisting conditions: coronary heart disease = Yes (%) | 47 (1.9) |
| Preexisting conditions: recurrent bacterial infections = Yes (%) | 23 (0.9) |
| Preexisting conditions: chronic obstructive pulmonary disease = Yes (%) | 50 (2.0) |
| Preexisting conditions: history of strokes = Yes (%) | 17 (0.7) |
| Preexisting conditions: peripheral neuropathy = Yes (%) | 16 (0.7) |
| Preexisting conditions: heart failure = Yes (%) | 14 (0.6) |
| Preexisting conditions: chemical allergies/ seasonal allergies = Yes (%) | 144 (5.9) |
| Preexisting conditions: other allergies = Yes (%) | 55 (2.2) |
| Preexisting conditions: viral skin conditions/coldsores = Yes (%) | 23 (0.9) |
| Preexisting conditions: herpes = Yes (%) | 23 (0.9) |
| Preexisting conditions: warts = Yes (%) | 23 (0.9) |
| Preexisting conditions: molluscum = Yes (%) | 23 (0.9) |
| Preexisting conditions: recurrent viral infections = Yes (%) | 29 (1.2) |
| Preexisting conditions: vivid dreams = Yes (%) | 37 (1.5) |
| Preexisting conditions: chronic kidney disease = Yes (%) | 13 (0.5) |

Table 1: Pre-infection medical history

| **Category** | **Subcategory/Type** | **Number** | **Percentage (%)** |
| --- | --- | --- | --- |
| **Hospitalization** | Yes | 304 | 12.4 |
| **Oxygen support** | No | 729 | 29.8 |
|  | Yes | 252 | 10.3 |
| **COVID-19 vaccination** | Yes | 1853 | 75.8 |
| **Vaccination shots** | 0 | 546 | 23.4 |
|  | 1 | 253 | 10.8 |
|  | 2 | 1347 | 57.6 |
|  | 3 | 183 | 7.8 |
|  | 4 | 8 | 0.3 |
| **Vaccine Type** | BBIBP-CorV (Sinopharm) | 242 | 9.9 |
|  | Comirnaty (Pfizer-BioNTech) | 257 | 10.5 |
|  | CoronaVac (Sinovac) | 525 | 21.5 |
|  | AstraZeneca (Vaxzevria/Covishield) | 563 | 23.0 |
|  | Moderna & Other types | 246 | 10.1 |
| **Medications Used** | Aspirin | 709 | 29.0 |
|  | Paracetamol | 871 | 35.6 |
|  | Ibuprofen | 459 | 18.8 |
|  | Naproxen | 459 | 18.8 |
|  | Anti-oxidants | 192 | 7.9 |
|  | Type one anti-histamine | 358 | 14.6 |
|  | Type two anti-histamine | 232 | 9.5 |
|  | Omega-3 | 215 | 8.8 |
|  | Azithromycin | 903 | 36.9 |
|  | Steroids | 496 | 20.3 |

Table 2: Vaccination, hospitalization, and treatments

| **COVID-19 ‎symptoms** | **N (%) / Mean (SD)‎** |
| --- | --- |
| Depression = Yes (%) | 1166 (47.7) |
| Symptom’s duration (mean (SD)) | 13.63 (17.50) |
| Chronic fatigue = Yes (%) | 159 (6.5) |
| Infection after vaccination (%) |  |
| Not Infected After Vaccination | 1099 (44.9) |
| Yes, Infected After Vaccination | 592 (24.2) |
| migraine = Yes (%) | 440 (18.0) |
| tinnitus experience = Yes (%) | 208 (8.5) |
| reproductive and urinary symptoms = Yes (%) | 2208 (90.3) |
| dizziness or vertigo experience = Yes (%) | 470 (19.2) |
| vertigo dizziness severity (mean (SD)) | 1.07 (2.04) |
| memory loss symptoms short term memory loss memory that lasts 30 seconds = Yes (%) | 407 (16.6) |
| memory loss symptoms or forgetting you are in the middle of a task = Yes (%) | 407 (16.6) |
| Brain fog symptoms poor attention or concentration = Yes (%) | 371 (15.2) |
| Brain fog symptoms difficulty with executive functioning planning = Yes (%) | 245 (10.0) |
| brain fog symptoms organizing = Yes (%) | 245 (10.0) |
| Brain fog symptoms figuring out the sequence of actions = Yes (%) | 245 (10.0) |
| Brain fog symptoms abstracting = Yes (%) | 245 (10.0) |
| Increase in mood symptoms anxiety = Yes (%) | 427 (17.5) |
| Increase in mood symptoms depression = Yes (%) | 410 (16.8) |
| Increase in mood symptoms mood swings = Yes (%) | 377 (15.4) |
| Increase in mood symptoms anger = Yes (%) | 452 (18.5) |
| Increase in mood symptoms difficulty controlling your emotions = Yes (%) | 264 (10.8) |
| Increase in mood symptoms irritability = Yes (%) | 360 (14.7) |
| Headaches symptoms headaches = Yes (%) | 910 (37.2) |
| Headaches symptoms behind the eyes = Yes (%) | 344 (14.1) |
| Smell and taste symptoms altered sense of smell = Yes (%) | 253 (10.3) |
| Smell and taste symptoms loss of taste = Yes (%) | 923 (37.8) |
| Smell and taste symptoms loss of smell = Yes (%) | 1145 (46.8) |
| Sleeping symptoms insomnia = Yes (%) | 634 (25.9) |
| Insomnia description difficulty falling asleep = Yes (%) | 538 (22.0) |
| Insomnia description of waking up several times during the night = Yes (%) | 361 (14.8) |
| Temperature symptoms temperature lability quick swings  in and out of fever or elevated temperature = Yes (%) | 534 (21.8) |
| CVS symptoms heart palpitations sensation or awareness of  your heart beating feeling like your heart is racing = Yes (%) | 346 (14.2) |
| CVS symptoms thumping or skipping beats = Yes (%) | 346 (14.2) |
| CVS symptoms tachycardia = Yes (%) | 353 (14.4) |
| Gastrointestinal symptom constipation = Yes (%) | 274 (11.2) |
| Gastrointestinal symptom diarrhea = Yes (%) | 518 (21.2) |
| Gastrointestinal symptom loss of appetite = Yes (%) | 428 (17.5) |
| Gastrointestinal symptom nausea = Yes (%) | 381 (15.6) |
| Gastrointestinal symptom vomiting = Yes (%) | 257 (10.5) |
| Respiratory symptoms cough with mucus production = Yes (%) | 408 (16.7) |
| Respiratory symptoms shortness of breath = Yes (%) | 575 (23.5) |
| Respiratory symptoms runny nose = Yes (%) | 627 (25.6) |
| Respiratory symptoms pain burning in chest = Yes (%) | 268 (11.0) |
| Respiratory symptoms sore throat = Yes (%) | 706 (28.9) |
| Respiratory symptoms sneezing = Yes (%) | 515 (21.1) |
| Respiratory symptoms tightness of chest = Yes (%) | 428 (17.5) |
| Respiratory symptoms dry cough = Yes (%) | 981 (40.1) |
| Muscle and joint symptoms joint pain = Yes (%) | 613 (25.1) |
| Muscle and joint symptoms muscle aches = Yes (%) | 774 (31.7) |
| Gastrointestinal symptoms abdominal pain = Yes (%) | 409 (16.7) |

*Table3; Symptoms and characteristics of COVID Infection*

| Overall | 1322 |
| --- | --- |
| menstruation = Yes (%) | 1003 (80.2) |
| pregnant = Yes (%) | 27 (2.0) |
| symptoms duration = more than two weeks (%) | 900 (68.1) |
| chronic fatigue = Yes (%) | 101 (7.6) |
| depression = Yes (%) | 715 (54.1) |

Table 4: Summary of female health-related variables

| Menstruation | No | Yes | p | test | SMD |
| --- | --- | --- | --- | --- | --- |
| n | 248 | 1003 |  |  |  |
| symptoms duration = more than two weeks (%) | 135 (54.4) | 721 (71.9) | <0.001 |  | 0.368 |
| chronic fatigue = Yes (%) | 26 (10.5) | 70 (7.0) | 0.085 |  | 0.124 |
| depression = Yes (%) | 137 (55.2) | 537 (53.5) | 0.681 |  | 0.034 |

Table 5: Comparison between female patients by menstruation

| Pregnant | No | Yes | p | test | SMD |
| --- | --- | --- | --- | --- | --- |
| n | 1295 | 27 |  |  |  |
| symptoms duration = more than two weeks (%) | 880 (68.0) | 20 (74.1) | 0.641 |  | 0.135 |
| chronic fatigue = Yes (%) | 99 (7.6) | 2 (7.4) | 1.000 |  | 0.009 |
| depressionBin = Yes (%) | 701 (54.1) | 14 (51.9) | 0.968 |  | 0.046 |

Table 6: Comparison between female patients by pregnancy

| **Infection after vaccination** | **(%)** | | | | | |
| --- | --- | --- | --- | --- | --- | --- |
| **Not vaccinated** | 734 (31.4) | | | | | |
| **No** | 1050 (44.9) | | | | | |
| **Yes** | 553 (23.7) | | | | | |
| **Dependent: infection after vaccination** |  | **Not vaccinated** | **No** | **Yes** | **OR (univariable)** | **OR (multivariable)** |
| tinnitus experience | No | 0 (0.0) | 1013 (65.5) | 533 (34.5) | - | - |
|  | Yes | 0 (0.0) | 86 (59.3) | 59 (40.7) | 1.30 (0.92-1.84, p=0.135) | 1.27 (0.86-1.86, p=0.218) |
| vertigo dizziness severity | Mean (SD) |  | 1.2 (2.2) | 1.2 (2.1) | 1.00 (0.95-1.05, p=0.987) | 0.95 (0.90-1.00, p=0.074) |
| increase in mood  symptoms mood swings | No | 0 (0.0) | 930 (64.4) | 513 (35.6) | - | - |
|  | Yes | 0 (0.0) | 169 (68.1) | 79 (31.9) | 0.85 (0.63-1.13, p=0.260) | 0.74 (0.53-1.03, p=0.078) |
| increase in mood  symptoms anger | No | 0 (0.0) | 885 (65.8) | 461 (34.2) | - | - |
|  | Yes | 0 (0.0) | 214 (62.0) | 131 (38.0) | 1.18 (0.92-1.50, p=0.196) | 1.33 (1.00-1.78, p=0.051) |
| increase in mood  symptoms irritability | No | 0 (0.0) | 924 (63.8) | 524 (36.2) | - | - |
|  | Yes | 0 (0.0) | 175 (72.0) | 68 (28.0) | 0.69 (0.50-0.92, p=0.013) | 0.59 (0.41-0.83, p=0.003) |
| sleeping  symptoms insomnia | No | 0 (0.0) | 815 (66.2) | 416 (33.8) | - | - |
|  | Yes | 0 (0.0) | 284 (61.7) | 176 (38.3) | 1.21 (0.97-1.51, p=0.087) | 1.44 (1.01-2.05, p=0.043) |
| insomnia description  difficulty falling asleep | No | 0 (0.0) | 856 (65.2) | 456 (34.8) | - | - |
|  | Yes | 0 (0.0) | 243 (64.1) | 136 (35.9) | 1.05 (0.83-1.33, p=0.685) | 0.74 (0.52-1.04, p=0.084) |
| insomnia description  waking up several times  during the night | No | 0 (0.0) | 928 (64.7) | 506 (35.3) | - | - |
|  | Yes | 0 (0.0) | 171 (66.5) | 86 (33.5) | 0.92 (0.69-1.22, p=0.573) | 0.70 (0.50-0.98, p=0.042) |
| gastrointestinal  symptom nausea | No | 0 (0.0) | 944 (66.5) | 475 (33.5) | - | - |
|  | Yes | 0 (0.0) | 155 (57.0) | 117 (43.0) | 1.50 (1.15-1.95, p=0.003) | 1.29 (0.95-1.76, p=0.102) |
| respiratory  symptoms runny nose | No | 0 (0.0) | 820 (63.9) | 463 (36.1) | - | - |
|  | Yes | 0 (0.0) | 279 (68.4) | 129 (31.6) | 0.82 (0.64-1.04, p=0.100) | 0.79 (0.61-1.02, p=0.075) |
| respiratory  symptoms pain  burning in chest | No | 0 (0.0) | 993 (66.2) | 507 (33.8) | - | - |
|  | Yes | 0 (0.0) | 106 (55.5) | 85 (44.5) | 1.57 (1.16-2.13, p=0.004) | 1.58 (1.12-2.22, p=0.009) |
| muscle and joint  symptoms joint pain | No | 0 (0.0) | 838 (67.4) | 405 (32.6) | - | - |
|  | Yes | 0 (0.0) | 261 (58.3) | 187 (41.7) | 1.48 (1.19-1.85, p=0.001) | 1.46 (1.12-1.90, p=0.005) |
| muscle and joint  symptoms muscle aches | No | 0 (0.0) | 729 (62.8) | 431 (37.2) | - | - |
|  | Yes | 0 (0.0) | 370 (69.7) | 161 (30.3) | 0.74 (0.59-0.92, p=0.006) | 0.59 (0.46-0.76, p<0.001) |
| gastrointestinal  symptoms  abdominal pain | No | 0 (0.0) | 936 (67.4) | 452 (32.6) | - | - |
|  | Yes | 0 (0.0) | 163 (53.8) | 140 (46.2) | 1.78 (1.38-2.29, p<0.001) | 1.53 (1.15-2.05, p=0.004) |
| chronic fatigue | No | 0 (0.0) | 1053 (66.3) | 535 (33.7) | - | - |
|  | Yes | 0 (0.0) | 46 (44.7) | 57 (55.3) | 2.44 (1.63-3.66, p<0.001) | 2.32 (1.49-3.63, p<0.001) |

Table 7: Infection after vaccination and associated symptoms: Number in data frame = 1691, AIC = 2129.1, C-statistic = 0.624, H&L = Chi-sq(8) 9.96 (p=0.268)

|  | No | Yes | p | SMD |
| --- | --- | --- | --- | --- |
| n | 553 | 1784 |  |  |
| Depression = Yes (%) | 269 (48.6) | 835 (46.8) | 0.479 | 0.037 |
| Symptoms duration = more than two weeks (%) | 327 (59.1) | 1308 (73.3) | <0.001 | 0.303 |
| Chronic fatigue = Yes (%) | 52 (9.4) | 95 (5.3) | 0.001 | 0.157 |

Table 8: Comparison between depression, symptoms duration and chronic fatigue by vaccination

| **Dependent: symptoms duration** |  | **less than two weeks** | **more than two weeks** | **OR (univariable)** | **OR (multivariable)** |
| --- | --- | --- | --- | --- | --- |
| dizziness or vertigo experience | No | 518 (27.4) | 1374 (72.6) | - | - |
|  | Yes | 184 (41.3) | 261 (58.7) | 0.53 (0.43-0.66, p<0.001) | 0.85 (0.67-1.08, p=0.169) |
| brain fog symptoims abstracting | No | 594 (28.1) | 1518 (71.9) | - | - |
|  | Yes | 108 (48.0) | 117 (52.0) | 0.42 (0.32-0.56, p<0.001) | 0.66 (0.48-0.90, p=0.008) |
| increase in mood symptoms anxiety | No | 530 (27.3) | 1413 (72.7) | - | - |
|  | Yes | 172 (43.7) | 222 (56.3) | 0.48 (0.39-0.61, p<0.001) | 0.76 (0.58-1.00, p=0.046) |
| increase in mood symptoms mood swings | No | 571 (28.8) | 1415 (71.2) | - | - |
|  | Yes | 131 (37.3) | 220 (62.7) | 0.68 (0.54-0.86, p=0.001) | 1.24 (0.94-1.66, p=0.130) |
| increase in mood symptoms anger | No | 523 (27.3) | 1393 (72.7) | - | - |
|  | Yes | 179 (42.5) | 242 (57.5) | 0.51 (0.41-0.63, p<0.001) | 0.77 (0.60-0.98, p=0.036) |
| loss of smell | No | 308 (24.7) | 941 (75.3) | - | - |
|  | Yes | 394 (36.2) | 694 (63.8) | 0.58 (0.48-0.69, p<0.001) | 0.72 (0.59-0.87, p=0.001) |
| tachycardia | No | 561 (27.9) | 1447 (72.1) | - | - |
|  | Yes | 141 (42.9) | 188 (57.1) | 0.52 (0.41-0.66, p<0.001) | 0.75 (0.58-0.98, p=0.032) |
| constipation | No | 596 (28.7) | 1484 (71.3) | - | - |
|  | Yes | 106 (41.2) | 151 (58.8) | 0.57 (0.44-0.75, p<0.001) | 0.84 (0.63-1.13, p=0.253) |
| loss of appetite | No | 527 (27.3) | 1403 (72.7) | - | - |
|  | Yes | 175 (43.0) | 232 (57.0) | 0.50 (0.40-0.62, p<0.001) | 0.73 (0.56-0.95, p=0.020) |
| symptom nausea | No | 560 (28.4) | 1410 (71.6) | - | - |
|  | Yes | 142 (38.7) | 225 (61.3) | 0.63 (0.50-0.79, p<0.001) | 1.28 (0.96-1.71, p=0.092) |
| shortness of breath | No | 473 (26.2) | 1330 (73.8) | - | - |
|  | Yes | 229 (42.9) | 305 (57.1) | 0.47 (0.39-0.58, p<0.001) | 0.73 (0.57-0.93, p=0.010) |
| runny nose | No | 515 (29.5) | 1231 (70.5) | - | - |
|  | Yes | 187 (31.6) | 404 (68.4) | 0.90 (0.74-1.11, p=0.326) | 1.25 (1.01-1.57, p=0.044) |
| tightness of chest | No | 526 (27.1) | 1416 (72.9) | - | - |
|  | Yes | 176 (44.6) | 219 (55.4) | 0.46 (0.37-0.58, p<0.001) | 0.79 (0.61-1.03, p=0.077) |
| dry cough | No | 362 (25.8) | 1043 (74.2) | - | - |
|  | Yes | 340 (36.5) | 592 (63.5) | 0.60 (0.51-0.72, p<0.001) | 0.80 (0.66-0.98, p=0.027) |
| gastrointestinal symptoms abdominal pain | No | 518 (26.6) | 1428 (73.4) | - | - |
|  | Yes | 184 (47.1) | 207 (52.9) | 0.41 (0.33-0.51, p<0.001) | 0.60 (0.47-0.78, p<0.001) |

Table 9: Logistic Regression mode for symptoms associated with long COVID syndrome: AIC = 2714.3, C-statistic = 0.669, H&L = Chi-sq(8) 12.80 (p=0.119)

| **Dependent: fatigue** |  | **No** | **Yes** | **OR (univariable)** | **OR (multivariable)** |
| --- | --- | --- | --- | --- | --- |
| Sex | Female | 1271 (92.0) | 111 (8.0) |  |  |
|  | Male | 1015 (95.5) | 48 (4.5) | 0.54 (0.38-0.76, p=0.001) | 0.64 (0.42-0.97, p=0.040) |
| Before covid health status | Good | 689 (91.9) | 61 (8.1) |  |  |
|  | Less than good | 175 (84.5) | 32 (15.5) | 2.07 (1.29-3.25, p=0.002) | 1.49 (0.89-2.45, p=0.125) |
|  | More than good | 1422 (95.6) | 66 (4.4) | 0.52 (0.37-0.75, p<0.001) | 0.64 (0.43-0.94, p=0.023) |
| Before covid mental health diagnsosis | No | 2090 (94.6) | 120 (5.4) |  |  |
|  | Yes | 196 (83.4) | 39 (16.6) | 3.47 (2.32-5.08, p<0.001) | 2.11 (1.33-3.29, p=0.001) |
| Before covid vertigo dizziness diagnosis | No | 1966 (95.4) | 95 (4.6) |  |  |
|  | Yes | 320 (83.3) | 64 (16.7) | 4.14 (2.94-5.79, p<0.001) | 2.73 (1.85-3.98, p<0.001) |
| Smoking habits | Never | 1916 (94.0) | 123 (6.0) |  |  |
|  | Occasionally | 232 (93.5) | 16 (6.5) | 1.07 (0.60-1.79, p=0.794) | 1.31 (0.70-2.31, p=0.368) |
|  | Regularly | 138 (87.3) | 20 (12.7) | 2.26 (1.33-3.66, p=0.002) | 2.39 (1.28-4.29, p=0.005) |
| Hospitalization due to covid | No | 2022 (94.4) | 119 (5.6) |  |  |
|  | Yes | 264 (86.8) | 40 (13.2) | 2.57 (1.74-3.74, p<0.001) | 2.41 (1.54-3.73, p<0.001) |
| Covid-19 vaccination | No | 535 (90.4) | 57 (9.6) |  |  |
|  | Yes | 1751 (94.5) | 102 (5.5) | 0.55 (0.39-0.77, p<0.001) | 0.71 (0.49-1.05, p=0.081) |
| Preexisting condition: vitamin-d deficiency | No | 2099 (94.6) | 119 (5.4) |  |  |
|  | Yes | 187 (82.4) | 40 (17.6) | 3.77 (2.53-5.52, p<0.001) | 2.74 (1.75-4.22, p<0.001) |
| Preexisting condition: asthma | No | 2151 (94.0) | 138 (6.0) |  |  |
|  | Yes | 135 (86.5) | 21 (13.5) | 2.42 (1.45-3.88, p<0.001) | 2.01 (1.13-3.44, p=0.013) |
| Preexisting condition: diabetes type2 two | No | 2148 (94.3) | 129 (5.7) |  |  |
|  | Yes | 138 (82.1) | 30 (17.9) | 3.62 (2.31-5.52, p<0.001) | 2.45 (1.44-4.06, p=0.001) |
| Preexisting condition: nightmares | No | 2185 (94.3) | 132 (5.7) |  |  |
|  | Yes | 101 (78.9) | 27 (21.1) | 4.43 (2.75-6.92, p<0.001) | 2.51 (1.46-4.21, p=0.001) |
| Covid treatments: aspirin | No | 1635 (94.2) | 101 (5.8) |  |  |
|  | Yes | 651 (91.8) | 58 (8.2) | 1.44 (1.03-2.01, p=0.032) | 1.10 (0.75-1.59, p=0.625) |
| Covid treatments: paracetamol | No | 1482 (94.2) | 92 (5.8) |  |  |
|  | Yes | 804 (92.3) | 67 (7.7) | 1.34 (0.97-1.86, p=0.077) | 1.49 (1.04-2.13, p=0.029) |
| Covid treatments: anti oxidants | No | 2104 (93.4) | 149 (6.6) |  |  |
|  | Yes | 182 (94.8) | 10 (5.2) | 0.78 (0.38-1.42, p=0.450) | 0.52 (0.23-1.03, p=0.080) |
| Covid treatments: type one histamine | No | 1947 (93.3) | 140 (6.7) |  |  |
|  | Yes | 339 (94.7) | 19 (5.3) | 0.78 (0.46-1.24, p=0.322) | 0.52 (0.28-0.89, p=0.024) |

Table 10: Logistic regression model for chronic fatigue syndrome: AIC = 1012.7, C-statistic = 0.804, H&L = Chi-sq(8) 10.35 (p=0.241)

| **Dependent: fatigue** |  | **No** | **Yes** | **OR (univariable)** | **OR (multivariable)** |
| --- | --- | --- | --- | --- | --- |
| Vertigo/dizziness severity | Mean (SD) | 1.0 (2.0) | 2.4 (2.7) | 1.28 (1.21-1.36, p<0.001) | 1.11 (1.03-1.18, p=0.004) |
| Memory loss symptoms/forgetting you’re in the middle of a task | No | 1945 (95.4) | 93 (4.6) |  |  |
|  | Yes | 341 (83.8) | 66 (16.2) | 4.05 (2.89-5.65, p<0.001) | 1.49 (0.99-2.22, p=0.052) |
| Brain fog symptoms: poor attention or concentration | No | 1982 (95.6) | 92 (4.4) |  |  |
|  | Yes | 304 (81.9) | 67 (18.1) | 4.75 (3.38-6.64, p<0.001) | 1.52 (1.00-2.28, p=0.045) |
| Brain fog symptoms: difficulty in abstracting | No | 2090 (95.0) | 110 (5.0) |  |  |
|  | Yes | 196 (80.0) | 49 (20.0) | 4.75 (3.27-6.82, p<0.001) | 1.55 (0.99-2.42, p=0.054) |
| Increase in mood symptoms anxiety | No | 1933 (95.8) | 85 (4.2) |  |  |
|  | Yes | 353 (82.7) | 74 (17.3) | 4.77 (3.42-6.64, p<0.001) | 1.62 (1.05-2.48, p=0.028) |
| increase in mood symptoms depression | No | 1955 (96.1) | 80 (3.9) |  |  |
|  | Yes | 331 (80.7) | 79 (19.3) | 5.83 (4.18-8.13, p<0.001) | 2.41 (1.59-3.66, p<0.001) |
| increase in mood symptoms irritability | No | 1969 (94.4) | 116 (5.6) |  |  |
|  | Yes | 317 (88.1) | 43 (11.9) | 2.30 (1.58-3.31, p<0.001) | 0.73 (0.45-1.15, p=0.188) |
| smell and taste  symptoms altered sense of smell | No | 2049 (93.5) | 143 (6.5) |  |  |
|  | Yes | 237 (93.7) | 16 (6.3) | 0.97 (0.55-1.60, p=0.903) | 0.48 (0.25-0.86, p=0.019) |
| insomnia description  difficulty falling asleep | No | 1832 (96.1) | 75 (3.9) |  |  |
|  | Yes | 454 (84.4) | 84 (15.6) | 4.52 (3.26-6.28, p<0.001) | 2.10 (1.44-3.07, p<0.001) |
| cvs symptoms thumping  or skipping beats | No | 2002 (95.4) | 97 (4.6) |  |  |
|  | Yes | 284 (82.1) | 62 (17.9) | 4.51 (3.19-6.33, p<0.001) | 1.93 (1.28-2.88, p=0.002) |
| gastrointestinal symptom  loss of appetite | No | 1925 (95.4) | 92 (4.6) |  |  |
|  | Yes | 361 (84.3) | 67 (15.7) | 3.88 (2.77-5.42, p<0.001) | 1.83 (1.24-2.70, p=0.002) |
| respiratory symptoms sore throat | No | 1634 (94.0) | 105 (6.0) |  |  |
|  | Yes | 652 (92.4) | 54 (7.6) | 1.29 (0.91-1.80, p=0.144) | 0.81 (0.54-1.21, p=0.308) |
| respiratory symptoms dry cough | No | 1379 (94.2) | 85 (5.8) |  |  |
|  | Yes | 907 (92.5) | 74 (7.5) | 1.32 (0.96-1.83, p=0.089) | 0.73 (0.49-1.06, p=0.100) |
| muscle and joint  symptoms joint pain | No | 1758 (96.0) | 74 (4.0) |  |  |
|  | Yes | 528 (86.1) | 85 (13.9) | 3.82 (2.76-5.31, p<0.001) | 1.53 (1.03-2.28, p=0.036) |
| muscle and joint  symptoms muscle aches | No | 1600 (95.8) | 71 (4.2) |  |  |
|  | Yes | 686 (88.6) | 88 (11.4) | 2.89 (2.09-4.01, p<0.001) | 1.51 (1.02-2.24, p=0.041) |

Table 11: Logistic regression model for symptoms associated with chronic fatigue: AIC = 953.5, C-statistic = 0.842, H&L = Chi-sq(8) 5.00 (p=0.757)

| **Dependent: depression** |  | **No** | **Yes** | **OR (univariable)** | **OR (multivariable)** |
| --- | --- | --- | --- | --- | --- |
| age group | 18-29 | 135 (50.6) | 132 (49.4) |  |  |
|  | 30-39 | 116 (47.5) | 128 (52.5) | 1.13 (0.80-1.60, p=0.495) | 0.92 (0.62-1.37, p=0.679) |
|  | 40-49 | 677 (49.5) | 692 (50.5) | 1.05 (0.80-1.36, p=0.740) | 1.43 (1.03-1.99, p=0.032) |
|  | 50-59 | 203 (65.1) | 109 (34.9) | 0.55 (0.39-0.77, p<0.001) | 0.71 (0.48-1.05, p=0.087) |
|  | > 60 | 148 (58.5) | 105 (41.5) | 0.73 (0.51-1.03, p=0.070) | 0.81 (0.54-1.20, p=0.296) |
| sex | Female | 630 (45.6) | 752 (54.4) |  |  |
|  | Male | 649 (61.1) | 414 (38.9) | 0.53 (0.45-0.63, p<0.001) | 0.64 (0.53-0.78, p<0.001) |
| healthcare professional | No | 834 (54.4) | 699 (45.6) |  |  |
|  | Yes | 445 (48.8) | 467 (51.2) | 1.25 (1.06-1.48, p=0.007) | 1.24 (1.02-1.52, p=0.034) |
| before covid health status | Good | 352 (46.9) | 398 (53.1) |  |  |
|  | Less than good | 55 (26.6) | 152 (73.4) | 2.44 (1.75-3.46, p<0.001) | 2.27 (1.57-3.33, p<0.001) |
|  | More than good | 872 (58.6) | 616 (41.4) | 0.62 (0.52-0.75, p<0.001) | 0.68 (0.56-0.83, p<0.001) |
| before covid mental health diagnsosis | No | 1239 (56.1) | 971 (43.9) |  |  |
|  | Yes | 40 (17.0) | 195 (83.0) | 6.22 (4.43-8.95, p<0.001) | 3.90 (2.69-5.77, p<0.001) |
| before covid tinnitus | No | 1225 (54.5) | 1022 (45.5) |  |  |
|  | Yes | 54 (27.3) | 144 (72.7) | 3.20 (2.33-4.45, p<0.001) | 1.88 (1.30-2.74, p=0.001) |
| before covid vertigo dizziness | No | 1181 (57.3) | 880 (42.7) |  |  |
|  | Yes | 98 (25.5) | 286 (74.5) | 3.92 (3.08-5.03, p<0.001) | 2.54 (1.93-3.37, p<0.001) |
| smoking | Never | 1074 (52.7) | 965 (47.3) |  |  |
|  | Occasionally | 125 (50.4) | 123 (49.6) | 1.10 (0.84-1.43, p=0.499) | 1.41 (1.04-1.91, p=0.027) |
|  | Regularly | 80 (50.6) | 78 (49.4) | 1.09 (0.78-1.50, p=0.621) | 1.23 (0.83-1.80, p=0.301) |
| oxygen support | I was not hospitalized | 827 (56.5) | 637 (43.5) |  |  |
|  | No | 356 (48.8) | 373 (51.2) | 1.36 (1.14-1.63, p=0.001) | 1.15 (0.94-1.41, p=0.171) |
|  | Yes | 96 (38.1) | 156 (61.9) | 2.11 (1.61-2.78, p<0.001) | 2.30 (1.66-3.20, p<0.001) |
| Preexisting conditions: anemia | No | 1193 (54.2) | 1009 (45.8) |  |  |
|  | Yes | 86 (35.4) | 157 (64.6) | 2.16 (1.64-2.85, p<0.001) | 1.40 (1.02-1.94, p=0.041) |
| Preexisting conditions: vision near sighted far sighted | No | 1142 (52.0) | 1054 (48.0) |  |  |
|  | Yes | 137 (55.0) | 112 (45.0) | 0.89 (0.68-1.15, p=0.367) | 0.70 (0.51-0.96, p=0.026) |
| Preexisting conditions: asthma | No | 1217 (53.2) | 1072 (46.8) |  |  |
|  | Yes | 62 (39.7) | 94 (60.3) | 1.72 (1.24-2.41, p=0.001) | 1.39 (0.95-2.04, p=0.094) |
| Preexisting conditions: diabetes type 2 | No | 1216 (53.4) | 1061 (46.6) |  |  |
|  | Yes | 63 (37.5) | 105 (62.5) | 1.91 (1.39-2.65, p<0.001) | 1.95 (1.32-2.90, p=0.001) |
| Preexisting conditions: migraine | No | 1213 (54.1) | 1029 (45.9) |  |  |
|  | Yes | 66 (32.5) | 137 (67.5) | 2.45 (1.81-3.34, p<0.001) | 1.55 (1.09-2.21, p=0.016) |
| Preexisting conditions: irritable bowel syndrome ibs | No | 1228 (54.1) | 1041 (45.9) |  |  |
|  | Yes | 51 (29.0) | 125 (71.0) | 2.89 (2.08-4.08, p<0.001) | 2.03 (1.40-2.97, p<0.001) |
| Preexisting conditions: insomnia | No | 1205 (54.7) | 999 (45.3) |  |  |
|  | Yes | 74 (30.7) | 167 (69.3) | 2.72 (2.05-3.64, p<0.001) | 1.74 (1.24-2.45, p=0.001) |
| Preexisting conditions: nightmares | No | 1248 (53.9) | 1069 (46.1) |  |  |
|  | Yes | 31 (24.2) | 97 (75.8) | 3.65 (2.45-5.60, p<0.001) | 1.79 (1.11-2.92, p=0.018) |
| Preexisting conditions: food allergies | No | 1213 (52.4) | 1100 (47.6) |  |  |
|  | Yes | 66 (50.0) | 66 (50.0) | 1.10 (0.78-1.57, p=0.585) | 0.68 (0.45-1.03, p=0.073) |
| aspirin | No | 960 (55.3) | 776 (44.7) |  |  |
|  | Yes | 319 (45.0) | 390 (55.0) | 1.51 (1.27-1.80, p<0.001) | 1.70 (1.25-2.31, p=0.001) |
| paracetamol | No | 783 (49.7) | 791 (50.3) |  |  |
|  | Yes | 496 (56.9) | 375 (43.1) | 0.75 (0.63-0.88, p=0.001) | 0.76 (0.63-0.93, p=0.006) |
| naproxen | No | 1058 (53.3) | 928 (46.7) |  |  |
|  | Yes | 221 (48.1) | 238 (51.9) | 1.23 (1.00-1.50, p=0.048) | 0.74 (0.52-1.05, p=0.092) |
| anti oxidants | No | 1169 (51.9) | 1084 (48.1) |  |  |
|  | Yes | 110 (57.3) | 82 (42.7) | 0.80 (0.60-1.08, p=0.151) | 0.68 (0.47-0.96, p=0.031) |
| anti type two histamine | No | 1137 (51.4) | 1076 (48.6) |  |  |
|  | Yes | 142 (61.2) | 90 (38.8) | 0.67 (0.51-0.88, p=0.005) | 0.50 (0.36-0.70, p<0.001) |
| omega 3 | No | 1197 (53.7) | 1033 (46.3) |  |  |
|  | Yes | 82 (38.1) | 133 (61.9) | 1.88 (1.41-2.51, p<0.001) | 1.53 (1.11-2.13, p=0.010) |

Table 12: Logistic regression model for depression :AIC = 2901.7, C-statistic = 0.758, H&L = Chi-sq(8) 9.38 (p=0.311)

| **Dependent: depression** |  | **No** | **Yes** | **OR (univariable)** | **OR (multivariable)** |
| --- | --- | --- | --- | --- | --- |
| migraine | No | 1127 (56.2) | 878 (43.8) |  |  |
|  | Yes | 152 (34.5) | 288 (65.5) | 2.43 (1.96-3.02, p<0.001) | 1.47 (1.14-1.88, p=0.003) |
| tinnitus experience | No | 1220 (54.5) | 1017 (45.5) |  |  |
|  | Yes | 59 (28.4) | 149 (71.6) | 3.03 (2.23-4.17, p<0.001) | 1.47 (1.02-2.14, p=0.039) |
| reproductive and urinary symptoms | No | 65 (27.4) | 172 (72.6) |  |  |
|  | Yes | 1214 (55.0) | 994 (45.0) | 0.31 (0.23-0.41, p<0.001) | 0.63 (0.45-0.89, p=0.009) |
| dizziness or vertigo experience | No | 1140 (57.7) | 835 (42.3) |  |  |
|  | Yes | 139 (29.6) | 331 (70.4) | 3.25 (2.62-4.05, p<0.001) | 1.49 (1.15-1.94, p=0.003) |
| memory loss symptoms or forgetting you re in the middle of a task | No | 1164 (57.1) | 874 (42.9) |  |  |
|  | Yes | 115 (28.3) | 292 (71.7) | 3.38 (2.69-4.28, p<0.001) | 1.24 (0.94-1.65, p=0.131) |
| brain fog symptoms poor attention or concentration | No | 1197 (57.7) | 877 (42.3) |  |  |
|  | Yes | 82 (22.1) | 289 (77.9) | 4.81 (3.73-6.27, p<0.001) | 2.21 (1.64-3.00, p<0.001) |
| brain fog symptoms abstracting | No | 1222 (55.5) | 978 (44.5) |  |  |
|  | Yes | 57 (23.3) | 188 (76.7) | 4.12 (3.05-5.65, p<0.001) | 1.20 (0.83-1.74, p=0.328) |
| increase in mood symptoms depression | No | 1193 (58.6) | 842 (41.4) |  |  |
|  | Yes | 86 (21.0) | 324 (79.0) | 5.34 (4.16-6.91, p<0.001) | 2.22 (1.65-3.00, p<0.001) |
| increase in mood symptoms anger | No | 1147 (57.6) | 846 (42.4) |  |  |
|  | Yes | 132 (29.2) | 320 (70.8) | 3.29 (2.64-4.11, p<0.001) | 1.38 (1.05-1.80, p=0.020) |
| increase in mood symptoms difficulty controlling your emotions | No | 1232 (56.5) | 949 (43.5) |  |  |
|  | Yes | 47 (17.8) | 217 (82.2) | 5.99 (4.36-8.40, p<0.001) | 2.12 (1.47-3.11, p<0.001) |
| headaches symptoms headaches | No | 935 (60.9) | 600 (39.1) |  |  |
|  | Yes | 344 (37.8) | 566 (62.2) | 2.56 (2.17-3.04, p<0.001) | 1.19 (0.97-1.46, p=0.098) |
| insomnia description difficulty falling asleep | No | 1116 (58.5) | 791 (41.5) |  |  |
|  | Yes | 163 (30.3) | 375 (69.7) | 3.25 (2.65-3.99, p<0.001) | 1.50 (1.18-1.91, p=0.001) |
| insomnia description waking up several times during the night | No | 1201 (57.6) | 883 (42.4) |  |  |
|  | Yes | 78 (21.6) | 283 (78.4) | 4.93 (3.80-6.47, p<0.001) | 2.65 (1.97-3.58, p<0.001) |
| cvs symptoms tachycardia | No | 1165 (55.7) | 927 (44.3) |  |  |
|  | Yes | 114 (32.3) | 239 (67.7) | 2.63 (2.08-3.36, p<0.001) | 1.43 (1.08-1.91, p=0.013) |
| respiratory symptoms cough with mucus production | No | 1104 (54.2) | 933 (45.8) |  |  |
|  | Yes | 175 (42.9) | 233 (57.1) | 1.58 (1.27-1.95, p<0.001) | 1.21 (0.93-1.56, p=0.153) |
| respiratory symptoms sore throat | No | 922 (53.0) | 817 (47.0) |  |  |
|  | Yes | 357 (50.6) | 349 (49.4) | 1.10 (0.93-1.31, p=0.271) | 0.80 (0.65-0.99, p=0.042) |
| respiratory symptoms tightness of chest | No | 1111 (55.1) | 906 (44.9) |  |  |
|  | Yes | 168 (39.3) | 260 (60.7) | 1.90 (1.54-2.35, p<0.001) | 0.86 (0.66-1.12, p=0.261) |
| muscle and joint symptoms joint pain | No | 1056 (57.6) | 776 (42.4) |  |  |
|  | Yes | 223 (36.4) | 390 (63.6) | 2.38 (1.97-2.88, p<0.001) | 1.26 (0.99-1.59, p=0.057) |
| muscle and joint symptoms muscle aches | No | 931 (55.7) | 740 (44.3) |  |  |
|  | Yes | 348 (45.0) | 426 (55.0) | 1.54 (1.30-1.83, p<0.001) | 0.90 (0.72-1.11, p=0.333) |
| gastrointestinal symptoms abdominal pain | No | 1158 (56.9) | 878 (43.1) |  |  |
|  | Yes | 121 (29.6) | 288 (70.4) | 3.14 (2.50-3.96, p<0.001) | 1.44 (1.09-1.89, p=0.010) |

Table 13: Logistic regression model for high risk associated symptoms for depression in covid infected patients: AIC = 2834.2, C-statistic = 0.771, H&L = Chi-sq(8) 20.23 (p=0.009)

| Cluster | Noise | Cluster 0 | Cluster 1 |
| --- | --- | --- | --- |
| Number of patients | 1994 | 16 | 435 |

Table 14: Clusters count

| cluster | variable | correlation |
| --- | --- | --- |
| Noise | migraine | 0.1788693 |
| Noise | tinnitus experience | 0.1450188 |
| Noise | reproductive and urinary symptoms | -0.1558118 |
| Noise | dizziness or vertigo experience | 0.2159453 |
| Noise | vertigo dizziness severity | 0.2670892 |
| Noise | memory loss symptoms Short-term memory loss memory that lasts 30 seconds | 0.2125303 |
| Noise | memory loss symptoms or forgetting you're in the middle of a task | 0.2125303 |
| Noise | Brain fog symptoms poor attention or concentration | 0.2011446 |
| Noise | brain fog symptoms difficulty with executive functioning planning | 0.1587075 |
| Noise | Brain fog symptoms organizing | 0.1587075 |
| Noise | brain fog symptoms figuring out the sequence of actions | 0.1587075 |
| Noise | Brain fog symptoms abstracting | 0.1587075 |
| Noise | increase in mood symptoms anxiety | 0.2187656 |
| Noise | increase in mood symptoms depression | 0.2134693 |
| Noise | increase in mood symptoms mood swings | 0.2030585 |
| Noise | increase in mood symptoms anger | 0.2264859 |
| Noise | increase in mood symptoms difficulty controlling your emotions | 0.1654626 |
| only noise | increase in mood symptoms irritability | 0.1976168 |
| Noise | headaches symptoms headaches | 0.3509075 |
| Noise | headaches symptoms behind the eyes | 0.1924385 |
| Noise | smell and taste symptoms altered sense of smell | 0.1615718 |
| Noise | smell and taste symptoms loss of taste | 0.2333117 |
| Noise | smell and taste symptoms loss of smell | 0.2878360 |
| Noise | sleeping symptoms insomnia | 0.2765793 |
| Noise | insomnia description of difficulty falling asleep | 0.2449684 |
| Noise | insomnia description of waking up several times during the night | 0.1979386 |
| Noise | temperature symptoms temperature lability  quick swings in and out of fever or elevated temperature | 0.2360868 |
| Noise | CVS symptoms heart palpitations sensation or  awareness of your heart beating feeling like your heart is racing | 0.1930890 |
| Noise | CVS symptoms thumping or skipping beats | 0.1930890 |
| Noise | CVS symptoms tachycardia | 0.1953585 |
| Noise | gastrointestinal symptom constipation | 0.1689550 |
| Noise | gastrointestinal symptom diarrhoea | 0.2388336 |
| Noise | gastrointestinal symptoms loss of appetite | 0.2190759 |
| Noise | gastrointestinal symptom nausea | 0.2043306 |
| Noise | gastrointestinal symptom vomiting | 0.1629929 |
| Noise | respiratory symptoms cough with mucus production | 0.2128434 |
| Noise | respiratory symptoms shortness of breath | 0.2562582 |
| Noise | respiratory symptoms runny nose | 0.2672201 |
| Noise | respiratory symptoms pain burning in the chest | 0.1668645 |
| Noise | respiratory symptoms sore throat | 0.2588139 |
| Noise | respiratory symptoms sneezing | 0.2430831 |
| Noise | respiratory symptoms tightness of the chest | 0.2190759 |
| Noise | respiratory symptoms dry cough | 0.2946427 |
| Noise | muscle and joint symptoms joint pain | 0.2556380 |
| Noise | muscle and joint symptoms muscle aches | 0.2919348 |
| Noise | gastrointestinal symptoms abdominal pain | 0.2131564 |
| Cluster one | migraine | 0.1732515 |
| Cluster one | tinnitus experience | -0.0247483 |
| Cluster one | reproductive and urinary symptoms | 0.0265902 |
| Cluster one | dizziness or vertigo experience | -0.0395924 |
| Cluster one | vertigo dizziness severity | -0.0433590 |
| Cluster one | memory loss symptoms short term memory loss memory that lasts 30 seconds | -0.0362695 |
| Cluster one | memory loss symptoms or forgetting you re in the middle of a task | -0.0362695 |
| Cluster one | brain fog symptoms poor attention or concentration | -0.0343264 |
| Cluster one | brain fog symptoms difficulty with executive functioning planning | -0.0270843 |
| Cluster one | brain fog symptoms organizing | -0.0270843 |
| Cluster one | brain fog symptoms figuring out the sequence of actions | -0.0270843 |
| Cluster one | brain fog symptoms abstracting | -0.0270843 |
| Cluster one | increase in mood symptoms anxiety | -0.0373336 |
| Cluster one | increase in mood symptoms depression | -0.0364297 |
| Cluster one | increase in mood symptoms mood swings | -0.0346531 |
| Cluster one | increase in mood symptoms anger | -0.0386511 |
| Cluster one | increase in mood symptoms difficulty controlling your emotions | -0.0282371 |
| Cluster one | increase in mood symptoms irritability | -0.0337244 |
| Cluster one | headaches symptoms headaches | -0.0519966 |
| Cluster one | headaches symptoms behind the eyes | -0.0328407 |
| Cluster one | smell and taste symptoms altered sense of smell | -0.0275731 |
| Cluster one | smell and taste symptoms loss of taste | -0.0632033 |
| Cluster one | smell and taste symptoms loss of smell | -0.0761688 |
| Cluster one | sleeping symptoms insomnia | -0.0480210 |
| Cluster one | insomnia description difficulty falling asleep | -0.0308640 |
| Cluster one | insomnia description waking up several times during the night | -0.0337793 |
| Cluster one | temperature symptoms temperature lability quick  swings in and out of fever or elevated temperature | -0.0429029 |
| Cluster one | CVS symptoms heart palpitations sensation or  awareness of your heart beating feeling like your heart is racing | -0.0329517 |
| Cluster one | CVS symptoms thumping or skipping beats | -0.0329517 |
| Cluster one | CVS symptoms tachycardia | -0.0333390 |
| Cluster one | gastrointestinal symptom constipation | -0.0288331 |
| Cluster one | gastrointestinal symptom diarrhea | -0.0420795 |
| Cluster one | gastrointestinal symptom loss of appetite | -0.0373865 |
| Cluster one | gastrointestinal symptom nausea | -0.0348702 |
| Cluster one | gastrointestinal symptom vomiting | -0.0278156 |
| Cluster one | respiratory symptoms cough with mucus production | -0.0363229 |
| Cluster one | respiratory symptoms shortness of breath | -0.0450049 |
| Cluster one | respiratory symptoms runny nose | -0.0476632 |
| Cluster one | respiratory symptoms pain burning in chest | -0.0284764 |
| Cluster one | respiratory symptoms sore throat | -0.0405197 |
| Cluster one | respiratory symptoms sneezing | -0.0419248 |
| Cluster one | respiratory symptoms tightness of chest | -0.0373865 |
| Cluster one | respiratory symptoms dry cough | -0.0664370 |
| Cluster one | muscle and joint symptoms joint pain | -0.0469476 |
| Cluster one | muscle and joint symptoms muscle aches | -0.0552368 |
| Cluster one | gastrointestinal symptoms abdominal pain | -0.0363763 |
| Cluster two | migraine | -0.2179294 |
| Cluster two | tinnitus experience | -0.1418552 |
| Cluster two | reproductive and urinary symptoms | 0.1524127 |
| Cluster two | dizziness or vertigo experience | -0.2106567 |
| Cluster two | vertigo dizziness severity | -0.2617309 |
| Cluster two | memory loss symptoms short term memory loss memory that lasts 30 seconds | -0.2078939 |
| Cluster two | memory loss symptoms or forgetting you re in the middle of a task | -0.2078939 |
| Cluster two | brain fog symptoms poor attention or concentration | -0.1967565 |
| Cluster two | brain fog symptoms difficulty with executive functioning planning | -0.1552452 |
| Cluster two | brain fog symptoms organizing | -0.1552452 |
| Cluster two | brain fog symptoms figuring out the sequence of actions | -0.1552452 |
| Cluster two | brain fog symptoms abstracting | -0.1552452 |
| Cluster two | increase in mood symptoms anxiety | -0.2139932 |
| Cluster two | increase in mood symptoms depression | -0.2088124 |
| Cluster two | increase in mood symptoms mood swings | -0.1986287 |
| Cluster two | increase in mood symptoms anger | -0.2215451 |
| Cluster two | increase in mood symptoms difficulty controlling your emotions | -0.1618530 |
| Cluster two | increase in mood symptoms irritability | -0.1933058 |
| Cluster two | headaches symptoms headaches | -0.3449154 |
| Cluster two | headaches symptoms behind the eyes | -0.1882404 |
| Cluster two | smell and taste symptoms altered sense of smell | -0.1580471 |
| Cluster two | smell and taste symptoms loss of taste | -0.2232913 |
| Cluster two | smell and taste symptoms loss of smell | -0.2758542 |
| Cluster two | sleeping symptoms insomnia | -0.2703725 |
| Cluster two | insomnia description difficulty falling asleep | -0.2419311 |
| Cluster two | insomnia description waking up several times during the night | -0.1936205 |
| Cluster two | temperature symptoms temperature lability quick swings in and out of fever or elevated temperature | -0.2303855 |
| Cluster two | CVS symptoms heart palpitations sensation or awareness of your heart beating feeling like your heart is racing | -0.1888767 |
| Cluster two | CVS symptoms thumping or skipping beats | -0.1888767 |
| Cluster two | CVS symptoms tachycardia | -0.1910967 |
| Cluster two | gastrointestinal symptom constipation | -0.1652692 |
| Cluster two | gastrointestinal symptom diarrhea | -0.2333448 |
| Cluster two | gastrointestinal symptom loss of appetite | -0.2142967 |
| Cluster two | gastrointestinal symptom nausea | -0.1998731 |
| Cluster two | gastrointestinal symptom vomiting | -0.1594371 |
| Cluster two | respiratory symptoms cough with mucus production | -0.2082002 |
| Cluster two | respiratory symptoms shortness of breath | -0.2503995 |
| Cluster two | respiratory symptoms runny nose | -0.2609562 |
| Cluster two | respiratory symptoms pain burning in chest | -0.1632243 |
| Cluster two | respiratory symptoms sore throat | -0.2539370 |
| Cluster two | respiratory symptoms sneezing | -0.2376871 |
| Cluster two | respiratory symptoms tightness of chest | -0.2142967 |
| Cluster two | respiratory symptoms dry cough | -0.2848092 |
| Cluster two | muscle and joint symptoms joint pain | -0.2493609 |

Table 15: Clusters correlation with the symptoms

| component | variable | Correlation >= 0.3 |
| --- | --- | --- |
| 1 | migraine | 0.3307484 |
| 1 | dizziness or vertigo experience | 0.4765465 |
| 1 | vertigo dizziness severity | 0.4650483 |
| 1 | memory loss symptoms short term  memory loss memory that lasts 30 seconds | 0.5094477 |
| 1 | memory loss symptoms or forgetting  you re in the middle of a task | 0.5094477 |
| 1 | brain fog symptoms poor attention or concentration | 0.4322597 |
| 1 | brain fog symptoms difficulty  with executive functioning planning | 0.6358214 |
| 1 | brain fog symptoms organizing | 0.6358214 |
| 1 | brain fog symptoms figuring out  the sequence of actions | 0.6358214 |
| 1 | brain fog symptoms abstracting | 0.6358214 |
| 1 | increase in mood symptoms anxiety | 0.5184317 |
| 1 | increase in mood symptoms depression | 0.4748632 |
| 1 | increase in mood symptoms mood swings | 0.4706471 |
| 1 | increase in mood symptoms anger | 0.4352869 |
| 1 | increase in mood symptoms  difficulty controlling your emotions | 0.4599080 |
| 1 | increase in mood symptoms irritability | 0.3933549 |
| 1 | headaches symptoms headaches | 0.5018773 |
| 1 | headaches symptoms behind the eyes | 0.3883802 |
| 1 | smell and taste symptoms loss of smell | 0.3221324 |
| 1 | sleeping symptoms insomnia | 0.4889700 |
| 1 | insomnia description difficulty falling asleep | 0.4547468 |
| 1 | insomnia description  waking up several times during the night | 0.3868535 |
| 1 | temperature symptoms temperature lability  quick swings in and out  of fever or elevated temperature | 0.3441842 |
| 1 | CVS symptoms heart palpitations sensation or  awareness of your heart beating  feeling like your heart is racing | 0.4789630 |
| 1 | CVS symptoms thumping or skipping beats | 0.4789630 |
| 1 | CVS symptoms tachycardia | 0.3980268 |
| 1 | gastrointestinal symptom diarrhea | 0.3699877 |
| 1 | gastrointestinal symptom loss of appetite | 0.4295250 |
| 1 | gastrointestinal symptom nausea | 0.4599029 |
| 1 | gastrointestinal symptom vomiting | 0.3628246 |
| 1 | respiratory symptoms shortness of breath | 0.4723422 |
| 1 | respiratory symptoms pain burning in chest | 0.4375157 |
| 1 | respiratory symptoms sneezing | 0.3087049 |
| 1 | respiratory symptoms tightness of chest | 0.4331550 |
| 1 | respiratory symptoms dry cough | 0.3011109 |
| 1 | muscle and joint symptoms joint pain | 0.4379077 |
| 1 | muscle and joint symptoms muscle aches | 0.3585333 |
| 1 | gastrointestinal symptoms abdominal pain | 0.4444533 |
| 2 | brain fog symptoms  difficulty with executive functioning planning | -0.7065951 |
| 2 | brain fog symptoms organizing | -0.7065951 |
| 2 | brain fog symptoms  figuring out the sequence of actions | -0.7065951 |
| 2 | brain fog symptoms abstracting | -0.7065951 |
| 3 | memory loss symptoms  short term memory loss  memory that lasts 30 seconds | -0.4114572 |
| 3 | memory loss symptoms or  forgetting you re in the middle of a task | -0.4114572 |
| 3 | respiratory symptoms runny nose | 0.3326078 |
| 3 | respiratory symptoms sore throat | 0.3335912 |
| 3 | respiratory symptoms sneezing | 0.3572643 |

Table 16: PCA components correlation with symptoms

**Autoencoder Bottleneck Layer and SCARF Latent Feature**

| Min. | 1st Qu. | Median | Mean | 3rd Qu. | Max. |
| --- | --- | --- | --- | --- | --- |
| 0.02932 | 0.13701 | 0.17854 | 0.20224 | 0.22792 | 0.89834 |

Table 17: The summary statistics of the square root of R2 for the correlation between the first SCARF latent feature and the outcome variables are:

The mean-variance explained by the first SCARF latent feature is 0.2.

| Min. | 1st Qu. | Median | Mean | 3rd Qu. | Max. |
| --- | --- | --- | --- | --- | --- |
| 0.1334 | 0.3344 | 0.4315 | 0.4139 | 0.4608 | 0.6075 |

Table 18: The summary statistics of the square root of R2 for the correlation between the encoder bottleneck layer output and the outcome variables are:

The mean-variance explained by the encoder bottleneck layer output is 0.4

| Dependent: encoder output |  | unit | value | Coefficient (univariable) | Coefficient (multivariable) |
| --- | --- | --- | --- | --- | --- |
| patient country | Egypt | Mean (sd) | 13.1 (67.1) |  |  |
|  | India | Mean (sd) | -28.8 (46.9) | -41.93 (-50.47 to -33.38, p<0.001) | -28.86 (-36.87 to -20.85, p<0.001) |
|  | Other | Mean (sd) | -0.9 (61.9) | -14.03 (-20.28 to -7.78, p<0.001) | -7.78 (-13.26 to -2.30, p=0.005) |
|  | Pakistan | Mean (sd) | -2.4 (60.9) | -15.51 (-23.35 to -7.68, p<0.001) | -10.27 (-17.40 to -3.14, p=0.005) |
|  | Syria | Mean (sd) | 10.9 (59.9) | -2.22 (-12.18 to 7.73, p=0.661) | -5.45 (-14.31 to 3.41, p=0.228) |
| sex | Female | Mean (sd) | 7.5 (66.5) |  |  |
|  | Male | Mean (sd) | -10.1 (55.2) | -17.63 (-22.58 to -12.68, p<0.001) | -5.18 (-9.69 to -0.66, p=0.025) |
| before covid health status | Good | Mean (sd) | 5.1 (63.4) |  |  |
|  | Less than good | Mean (sd) | 18.8 (67.0) | 13.73 (4.18 to 23.29, p=0.005) | 4.28 (-3.68 to 12.23, p=0.292) |
|  | More than good | Mean (sd) | -5.5 (60.6) | -10.53 (-15.98 to -5.09, p<0.001) | -2.23 (-6.84 to 2.39, p=0.344) |
| before covid tinnitus | 0 | Mean (sd) | -5.3 (57.7) |  |  |
|  | 1 | Mean (sd) | 57.4 (82.5) | 62.68 (53.94 to 71.41, p<0.001) | 33.73 (25.94 to 41.52, p<0.001) |
| before covid vertigo dizziness | No | Mean (sd) | -10.9 (53.9) |  |  |
|  | Yes | Mean (sd) | 57.2 (73.4) | 68.10 (61.85 to 74.36, p<0.001) | 43.93 (37.91 to 49.95, p<0.001) |
| smoking | Never | Mean (sd) | -1.1 (61.5) |  |  |
|  | Occasionally | Mean (sd) | 0.4 (67.0) | 1.46 (-6.78 to 9.70, p=0.728) | 6.46 (-0.45 to 13.37, p=0.067) |
|  | Regularly | Mean (sd) | 10.2 (66.6) | 11.20 (1.09 to 21.31, p=0.030) | 5.76 (-2.90 to 14.43, p=0.192) |
| hospitalization | No | Mean (sd) | -2.8 (61.7) |  |  |
|  | Yes | Mean (sd) | 18.4 (65.2) | 21.19 (13.72 to 28.65, p<0.001) | 14.75 (8.00 to 21.51, p<0.001) |
| covid 19 vaccination | No | Mean (sd) | 5.8 (64.3) |  |  |
|  | Yes | Mean (sd) | -2.1 (61.8) | -7.88 (-13.66 to -2.11, p=0.008) | -9.90 (-22.71 to 2.90, p=0.130) |
| type of the vaccine | BBIBP-CorV (Beijing Institute of Biological Products: China National Pharmaceutical Group (Sinopharm)) | Mean (sd) | 8.4 (64.0) |  |  |
|  | Comirnaty (BNT162b2) ( Pfizer, BioNTech: Fosun Pharma) | Mean (sd) | -4.0 (63.2) | -12.41 (-23.36 to -1.46, p=0.026) | -2.40 (-11.55 to 6.75, p=0.607) |
|  | CoronaVac (Sinovac) | Mean (sd) | 0.1 (61.2) | -8.30 (-17.79 to 1.20, p=0.087) | -0.28 (-8.37 to 7.81, p=0.946) |
|  | COVID-19 Vaccine AstraZeneca (AZD1222): also known as Vaxzevria and Covishield ( BARDA, OWS) | Mean (sd) | -7.5 (58.5) | -15.92 (-25.32 to -6.53, p=0.001) | 0.70 (-7.49 to 8.89, p=0.867) |
|  | Moderna & Other types | Mean (sd) | -0.3 (61.7) | -8.72 (-19.78 to 2.35, p=0.123) | -1.86 (-11.17 to 7.45, p=0.695) |
|  | Not vaccinated | Mean (sd) | 4.6 (65.7) | -3.79 (-13.08 to 5.49, p=0.423) | -11.75 (-25.92 to 2.42, p=0.104) |
| Preexisting conditions: acid reflux disease | No | Mean (sd) | -3.8 (58.9) |  |  |
|  | Yes | Mean (sd) | 40.9 (83.4) | 44.76 (35.90 to 53.63, p<0.001) | 12.70 (4.80 to 20.59, p=0.002) |
| Preexisting conditions: anemia | No | Mean (sd) | -4.0 (59.7) |  |  |
|  | Yes | Mean (sd) | 34.2 (75.2) | 38.13 (29.98 to 46.27, p<0.001) | 13.66 (6.48 to 20.84, p<0.001) |
| 1 pre existing conditions  vitamin d deficiency | No | Mean (sd) | -4.5 (59.4) |  |  |
|  | Yes | Mean (sd) | 41.8 (75.4) | 46.31 (37.97 to 54.65, p<0.001) | 21.69 (14.32 to 29.06, p<0.001) |
| Preexisting conditions: asthma | No | Mean (sd) | -2.1 (60.8) |  |  |
|  | Yes | Mean (sd) | 28.3 (78.5) | 30.39 (20.32 to 40.46, p<0.001) | 7.08 (-1.60 to 15.76, p=0.110) |
| Preexisting conditions: diabetes type 2 | No | Mean (sd) | -1.8 (61.4) |  |  |
|  | Yes | Mean (sd) | 22.3 (72.6) | 24.09 (14.34 to 33.84, p<0.001) | 9.53 (0.74 to 18.32, p=0.034) |
| Preexisting conditions:  hypertension high blood pressure | No | Mean (sd) | -2.4 (61.3) |  |  |
|  | Yes | Mean (sd) | 20.3 (69.6) | 22.63 (14.28 to 30.97, p<0.001) | 3.83 (-3.76 to 11.42, p=0.322) |
| Preexisting conditions: migraine | No | Mean (sd) | -4.3 (59.0) |  |  |
|  | Yes | Mean (sd) | 45.4 (79.4) | 49.74 (40.98 to 58.50, p<0.001) | 19.02 (11.19 to 26.84, p<0.001) |
| Preexisting conditions:  irritable bowel syndrome ibs | No | Mean (sd) | -3.2 (60.9) |  |  |
|  | Yes | Mean (sd) | 38.5 (70.1) | 41.71 (32.27 to 51.16, p<0.001) | 15.28 (7.11 to 23.44, p<0.001) |
| Preexisting conditions: insomnia | No | Mean (sd) | -5.6 (58.4) |  |  |
|  | Yes | Mean (sd) | 49.4 (75.4) | 54.98 (46.96 to 63.00, p<0.001) | 23.61 (16.29 to 30.93, p<0.001) |
| Preexisting conditions: nightmares | No | Mean (sd) | -3.5 (59.6) |  |  |
|  | Yes | Mean (sd) | 59.3 (81.6) | 62.78 (51.93 to 73.62, p<0.001) | 18.80 (9.13 to 28.46, p<0.001) |
| Preexisting conditions: mold | No | Mean (sd) | -3.1 (60.4) |  |  |
|  | Yes | Mean (sd) | 19.8 (72.1) | 22.95 (15.57 to 30.32, p<0.001) | 7.77 (1.34 to 14.21, p=0.018) |
| Preexisting conditions: food allergies | No | Mean (sd) | -1.5 (61.3) |  |  |
|  | Yes | Mean (sd) | 22.8 (76.9) | 24.31 (13.38 to 35.23, p<0.001) | 6.44 (-2.89 to 15.77, p=0.176) |
| naproxen | No | Mean (sd) | -2.4 (60.5) |  |  |
|  | Yes | Mean (sd) | 9.5 (69.8) | 11.94 (5.61 to 18.27, p<0.001) | 6.30 (0.99 to 11.60, p=0.020) |
| anti type one histamine | No | Mean (sd) | -0.4 (61.8) |  |  |
|  | Yes | Mean (sd) | 0.8 (66.5) | 1.19 (-5.82 to 8.20, p=0.740) | -3.74 (-9.91 to 2.44, p=0.236) |
| azithromycin | No | Mean (sd) | -4.3 (60.3) |  |  |
|  | Yes | Mean (sd) | 6.8 (65.4) | 11.06 (5.95 to 16.18, p<0.001) | 6.61 (2.28 to 10.94, p=0.003) |
| steroids | No | Mean (sd) | -5.5 (59.7) |  |  |
|  | Yes | Mean (sd) | 20.9 (68.4) | 26.47 (20.39 to 32.54, p<0.001) | 16.00 (10.48 to 21.52, p<0.001) |

Table 19: Linear regression model with the encoder bottleneck layer as a dependent variable (a low dimensional representation of the infection outcome symptoms: AIC = 26190.1, R-squared = 0.35, Adjusted R-squared = 0.34

| Dependent: embeddings |  | unit | value | Coefficient (univariable) | Coefficient (multivariable) |
| --- | --- | --- | --- | --- | --- |
| before covid mental health diagnsosis | No | Mean (sd) | 0.3 (0.7) |  |  |
|  | Yes | Mean (sd) | 0.7 (1.0) | 0.36 (0.26 to 0.47, p<0.001) | 0.06 (-0.02 to 0.15, p=0.148) |
| before covid tinnitus | No | Mean (sd) | 0.2 (0.6) |  |  |
|  | Yes | Mean (sd) | 1.9 (1.1) | 1.72 (1.63 to 1.81, p<0.001) | 1.61 (1.51 to 1.70, p<0.001) |
| before covid vertigo dizziness | No | Mean (sd) | 0.3 (0.7) |  |  |
|  | Yes | Mean (sd) | 0.9 (1.1) | 0.61 (0.53 to 0.69, p<0.001) | 0.22 (0.15 to 0.29, p<0.001) |
| hospitalization | No | Mean (sd) | 0.3 (0.8) |  |  |
|  | Yes | Mean (sd) | 0.5 (0.9) | 0.20 (0.11 to 0.29, p<0.001) | 0.20 (0.12 to 0.28, p<0.001) |
| Preexisting conditions: vitamin d deficiency | No | Mean (sd) | 0.3 (0.8) |  |  |
|  | Yes | Mean (sd) | 0.6 (0.9) | 0.23 (0.12 to 0.33, p<0.001) | 0.08 (-0.00 to 0.17, p=0.053) |
| Preexisting conditions: migraine | No | Mean (sd) | 0.3 (0.8) |  |  |
|  | Yes | Mean (sd) | 0.6 (0.9) | 0.23 (0.12 to 0.35, p<0.001) | 0.09 (-0.00 to 0.18, p=0.058) |
| Preexisting conditions: irritable bowel syndrome ibs | No | Mean (sd) | 0.3 (0.8) |  |  |
|  | Yes | Mean (sd) | 0.6 (0.9) | 0.22 (0.10 to 0.34, p<0.001) | 0.14 (0.05 to 0.24, p=0.003) |
| Preexisting conditions: nightmares | No | Mean (sd) | 0.3 (0.7) |  |  |
|  | Yes | Mean (sd) | 0.8 (1.1) | 0.51 (0.37 to 0.64, p<0.001) | 0.15 (0.04 to 0.26, p=0.010) |
| anti type one histamine | No | Mean (sd) | 0.4 (0.8) |  |  |
|  | Yes | Mean (sd) | 0.3 (0.7) | -0.06 (-0.14 to 0.03, p=0.213) | -0.08 (-0.15 to -0.01, p=0.026) |
| steroids | No | Mean (sd) | 0.3 (0.8) |  |  |
|  | Yes | Mean (sd) | 0.4 (0.8) | 0.11 (0.03 to 0.19, p=0.005) | 0.05 (-0.01 to 0.12, p=0.111) |

Table 20: Linear regression model with SCARF embeddings(X0) as a dependent variable (a low dimensional representation of the infection outcome symptoms: AIC = 4483.7, R-squared = 0.4, Adjusted R-squared = 0.4

|  | Chronic Fatigue | Depression | Symptoms Duration |
| --- | --- | --- | --- |
| AUC | 0.87 | 0.82 | 0.74 |
| Brier Score | 0.06570519. | 0.1749413 | 0.1937747 |
| Accuracy | 0.7349 | 0.762 | 0.6931 |
| 95% CI | (0.6929, 0.7739) | (0.7213,0.7995) | (0.6497, 0.7342) |
| No Information Rate | 0.9353 | 0.5198 | 0.6827 |
| P-Value [Acc > NIR] | 1 | <2e-16 | 0.3311 |
| Kappa | 0.2224 | 0.5223 | 0.3521 |
| Mcnemar's Test P-Value | <2e-16 | 0.3029 | 1.80E-05 |
| Sensitivity | 0.7232 | 0.7952 | 0.6908 |
| Specificity | 0.9032 | 0.7261 | 0.6942 |
| Pos Pred Value | 0.9908 | 0.7586 | 0.5122 |
| Neg Pred Value | 0.1842 | 0.7661 | 0.8285 |
| Prevalence | 0.9353 | 0.5198 | 0.3173 |
| Detection Rate | 0.6764 | 0.4134 | 0.2192 |
| Detection Prevalence | 0.6827 | 0.5449 | 0.428 |
| Balanced Accuracy | 0.8132 | 0.7606 | 0.6925 |

Table 21: Accuracy metrices for gradient boosting machines (GMB)

|  | Variable | scaled importance | percentage |
| --- | --- | --- | --- |
| 1 | increase in depressed mood | 1 | 0.106480376 |
| 2 | insomnia | 0.913021874 | 0.0972189124 |
| 3 | before covid health status | 0.493855663 | 0.0525859366 |
| 4 | brain fog symptoms poor attention or concentration | 0.469670353 | 0.0500106758 |
| 5 | waking up several times during the night | 0.450046121 | 0.0479210802 |
| 6 | infection with covid 19 after vaccination | 0.425302624 | 0.0452863833 |
| 7 | before covid mental health diagnsosis | 0.415821397 | 0.0442768187 |
| 8 | before covid vertigo dizziness | 0.414714165 | 0.0441589202 |
| 9 | increase in mood symptoms difficulty  controlling your emotions | 0.353715963 | 0.0376638088 |
| 10 | oxygen support | 0.334392865 | 0.035606278 |
| 11 | memory loss symptoms or forgetting you re  in the middle of a task | 0.326719032 | 0.0347891654 |
| 12 | headaches symptoms headaches | 0.298612806 | 0.0317964039 |
| 13 | muscle and joint symptoms joint pain | 0.297634512 | 0.0316922347 |
| 14 | migraine | 0.281416409 | 0.029965325 |
| 15 | healthcare professional | 0.28034327 | 0.0298510567 |
| 16 | increase in mood symptoms anger | 0.270913791 | 0.0288470023 |
| 17 | muscle and joint symptoms muscle aches | 0.254314833 | 0.027079539 |
| 18 | aspirin | 0.205095513 | 0.0218386473 |
| 19 | gastrointestinal symptoms abdominal pain | 0.182929749 | 0.0194784285 |
| 20 | paracetamol | 0.174155062 | 0.0185440965 |
| 21 | brain fog symptoms abstracting | 0.151616298 | 0.0161441604 |
| 22 | pre existing conditions diabetes type 2 | 0.149440663 | 0.015912498 |
| 23 | CVS symptoms thumping or skipping beats | 0.140574801 | 0.0149684576 |
| 24 | respiratory symptoms cough with mucus production | 0.132663129 | 0.0141260199 |
| 25 | omega 3 | 0.132302884 | 0.0140876608 |
| 26 | naproxen | 0.118621634 | 0.0126308762 |
| 27 | respiratory symptoms tightness of chest | 0.114109117 | 0.0121503817 |
| 28 | anti type two histamine | 0.111387217 | 0.0118605527 |
| 29 | tinnitus experience | 0.108125734 | 0.0115132688 |
| 30 | CVS symptoms tachycardia | 0.103955396 | 0.0110692097 |
| 31 | pre existing conditions vision near sighted far sighted | 0.076761771 | 0.0081736223 |
| 32 | pre existing conditions nightmares | 0.075190018 | 0.0080062613 |
| 33 | anti oxidants | 0.063720594 | 0.0067849928 |
| 34 | pre existing conditions irritable bowel syndrome ibs | 0.063474197 | 0.0067587564 |
| 35 | pre existing conditions food allergies | 0.006782394 | 0.0007221918 |

Table 22: The scaled importance of the variables for the depression model

|  | variable | relative importance | scaled importance | percentage |
| --- | --- | --- | --- | --- |
| 1 | pre existing conditions diabetes type two | 838.5575 | 1 | 0.11161723 |
| 2 | pre existing conditions nightmares | 555.716 | 0.66270471 | 0.073969264 |
| 3 | increase in mood symptoms depression | 512.74945 | 0.61146606 | 0.068250148 |
| 4 | pre existing conditions vitamin d deficiency | 488.22693 | 0.58222237 | 0.064986048 |
| 5 | before covid mental health diagnsosis | 486.24197 | 0.57985526 | 0.064721838 |
| 6 | increase in mood symptoms anxiety | 395.49188 | 0.47163359 | 0.052642435 |
| 7 | brain fog symptoms poor attention or concentration | 394.22079 | 0.47011779 | 0.052473245 |
| 8 | CVS symptoms thumping or skipping beats | 378.30646 | 0.45113956 | 0.050354948 |
| 9 | memory loss symptoms or forgetting you re in the middle of a task | 338.73123 | 0.40394515 | 0.045087238 |
| 10 | hospitalization | 331.82513 | 0.39570946 | 0.044167994 |
| 11 | before covid vertigo dizziness | 287.76624 | 0.34316816 | 0.03830348 |
| 12 | gastrointestinal symptom loss of appetite | 276.77728 | 0.33006357 | 0.036840782 |
| 13 | insomnia description difficulty falling asleep | 241.33426 | 0.28779691 | 0.032123094 |
| 14 | muscle and joint symptoms muscle aches | 174.26871 | 0.20781963 | 0.023196251 |
| 15 | infection with covid 19 after vaccination | 163.84651 | 0.19539091 | 0.021808992 |
| 16 | increase in mood symptoms irritability | 163.19957 | 0.19461942 | 0.02172288 |
| 17 | paracetamol | 161.96965 | 0.19315271 | 0.02155917 |
| 18 | pre-existing conditions insomnia | 141.13669 | 0.1683089 | 0.018786173 |
| 19 | increase in mood symptoms anger | 132.23813 | 0.15769715 | 0.017601719 |
| 20 | before covid health status | 121.01552 | 0.14431392 | 0.01610792 |
| 21 | smoking | 121.01072 | 0.1443082 | 0.016107281 |
| 22 | pre existing conditions mold | 119.01041 | 0.14192278 | 0.015841028 |
| 23 | omega 3 | 104.73107 | 0.12489432 | 0.013940358 |
| 24 | smell and taste symptoms altered sense of smell | 88.99924 | 0.10613374 | 0.011846354 |
| 25 | aspirin | 85.53922 | 0.10200758 | 0.011385804 |
| 26 | pre existing conditions vision near sighted far sighted | 81.57204 | 0.09727663 | 0.010857748 |
| 27 | respiratory symptoms dry cough | 77.00585 | 0.09183133 | 0.010249959 |
| 28 | anti oxidants | 73.41665 | 0.08755112 | 0.009772214 |
| 29 | type vaccine | 70.1718 | 0.08368156 | 0.009340304 |
| 30 | anti-type one histamine | 60.42504 | 0.07205831 | 0.008042949 |
| 31 | naproxen | 47.2942 | 0.05639947 | 0.006295153 |

Table 23: The scaled importance of the variables for the chronic fatigue model

|  | variable | relative importance | scaled importance | percentage |
| --- | --- | --- | --- | --- |
| 1 | hospitalization | 154.070312 | 1 | 0.236558229 |
| 2 | BMI | 62.151985 | 0.40340014 | 0.095427622 |
| 3 | infection with covid 19 after vaccination | 51.257229 | 0.33268725 | 0.078699907 |
| 4 | smell and taste symptoms loss of smell | 50.846489 | 0.33002133 | 0.078069261 |
| 5 | pre-existing conditions hypertension high blood pressure | 40.157444 | 0.26064362 | 0.061657393 |
| 6 | increase in mood symptoms anxiety | 38.684525 | 0.25108357 | 0.059395885 |
| 7 | gastrointestinal symptoms abdominal pain | 35.396069 | 0.22973971 | 0.054346818 |
| 8 | respiratory symptoms tightness of the chest | 31.102413 | 0.20187155 | 0.047754377 |
| 9 | insomnia description difficulty falling asleep | 30.912626 | 0.20063973 | 0.04746298 |
| 10 | steroids | 29.848188 | 0.19373095 | 0.045828651 |
| 11 | respiratory symptoms dry cough | 27.647285 | 0.1794459 | 0.042449404 |
| 12 | azithromycin | 25.457605 | 0.16523368 | 0.039087388 |
| 13 | CVS symptoms tachycardia | 22.849661 | 0.14830671 | 0.035083172 |
| 14 | aspirin | 11.004047 | 0.07142224 | 0.016895519 |
| 15 | sex | 7.620526 | 0.04946135 | 0.01170049 |
| 16 | naproxen | 6.892515 | 0.04473617 | 0.010582708 |
| 17 | pre existing conditions anemia | 5.766493 | 0.03742767 | 0.008853823 |
| 18 | pre existing conditions migraine | 5.294567 | 0.03436461 | 0.008129232 |
| 19 | pre existing conditions nightmares | 4.627678 | 0.03003615 | 0.007105297 |
| 20 | migraine | 4.144969 | 0.0269031 | 0.00636415 |
| 21 | pre existing conditions mold | 3.142699 | 0.02039782 | 0.004825273 |
| 22 | anti oxidants | 2.424412 | 0.01573575 | 0.003722421 |

Table 24: The scaled importance of the variables for the symptoms duration model

# Machine learning: symptoms duration

Load libraries

## Connection successful!

##

## R is connected to the H2O cluster:

## H2O cluster uptime: 1 days 7 hours

## H2O cluster timezone: Africa/Cairo

## H2O data parsing timezone: UTC

## H2O cluster version: 3.44.0.3

## H2O cluster version age: 8 months and 6 days

## H2O cluster name: H2O_started_from_R_ahmedshaheen_ojv136

## H2O cluster total nodes: 1

## H2O cluster total memory: 1.93 GB

## H2O cluster total cores: 8

## H2O cluster allowed cores: 8

## H2O cluster healthy: TRUE

## H2O Connection ip: localhost

## H2O Connection port: 54321

## H2O Connection proxy: NA

## H2O Internal Security: FALSE

## R Version: R version 4.4.1 (2024-06-14)

Laod data files

Get the data for analysis

# dfAnalysis <- read.csv("/Volumes/Ahmed Shaheen/Old Files/long-covid-shaheen/model_data.csv")[,-1]

#

# # Convert them to factors using lapply

# dfAnalysis[names(dfAnalysis %>% select(!c("BMI")))] <- lapply(dfAnalysis[names(dfAnalysis %>% select(!c("BMI")))], as.factor)

#

#

# levels(dfAnalysis$age_group) <- c("18-29", "30-39", "40-49", "50-59","> 60")

# levels(dfAnalysis$infection_with_covid_19_after_vaccination) <- c("Not vaccinated", "No","Yes")

#

# levels(dfAnalysis$household_income) <- c("More than $10,000","Don’t know/Not sure", "Less than $10,000")

Get the outcomes data

# outcomes <- read.csv("/Volumes/Ahmed Shaheen/Old Files/long-covid-shaheen/df1_outcomes.csv")[-1] %>%

# select(!c("x52_dizziness_or_vertigo_symptoms","x56_tinnitus_symptoms",

# "x32_muscle_and_joint_symptoms_muscle_and_joint_issues",

# "x10_headaches_symptoms_diffuse_entire_brain","x26_gastrointestinal_symptom_abdominal_pain",

# "x3_memory_loss_symptoms_i_e_remembering_a_phone_number_before_writing_it_down",

# "x22_temperature_symptoms_other_temperature_issues_not_listed_above_or_below",

# "x24_generic_symptoms_dizziness_vertigo_unsteadiness_or_balance_issues"))

#

# outcomes$depression <- factor(outcomes$depression,

# levels=c("Mild", "Moderate", "Moderately severe", "None", "Severe"),

# labels = c("Mild", "Moderate", "ModeratelySevere", "None", "Severe"))

#

#

# outcomes$depression <- as.character(outcomes$depression)

#

# outcomes$depressionBin[outcomes$depression %in% c("Mild", "Moderate", "ModeratelySevere", "Severe")] <- "Yes"

#

# outcomes$depressionBin[outcomes$depression %in% c("None")] <- "No"

#

# outcomes$depression <- as.factor(outcomes$depression)

#

# outcomes$depressionBin <- as.factor(outcomes$depressionBin)

#

# # Convert them to factors using lapply

# outcomes[names(outcomes %>% select(!c("x53_vertigo_dizziness_severity")))] <- lapply(outcomes[names(outcomes %>% select(!c("x53_vertigo_dizziness_severity")))], factor)

#

# Embeddings <- read.csv("/Volumes/Ahmed Shaheen/Old Files/long-covid-shaheen/dataWithembddings.csv")

# Data <- read.csv("/Volumes/Ahmed Shaheen/Old Files/long-covid-shaheen/dfAnalysis.csv")

#

# longCovid <- Data$longCovid

# encoder_output <- Data$layer_output

# embeddings <- Embeddings$X0

# symptoms_duration <- Data$symptoms_duration

# set.seed(123)

# dat <- dfAnalysis %>% select(!c("covid_19_vaccination",

# "household_income","vaccine_shots_n"))

#

# dat <- cbind(dat,outcomes[,1:46]) %>%

# mutate_if(is.factor, ~as.numeric(as.factor(.)))

#

#

# my_preprocess <- preProcess(dat, method = c("center", "scale", "YeoJohnson"))

# dat <- predict(my_preprocess, dat)

#

# #Remove highly correlated variables

# cor_matrix <- cor(dat)

# cor_matrix_rm <- cor_matrix

# cor_matrix_rm[upper.tri(cor_matrix_rm)] <- 0

# diag(cor_matrix_rm) <- 0

#

# dat <- dat[ , !apply(cor_matrix_rm, 2, function(x) any(x > 0.99))]

# heatmap(cor(dat))

#

#

# dat <- dat %>% select(!names(dat)[nearZeroVar(dat)])

#

# dat$symptoms_durationBin <- ifelse(symptoms_duration >= 15, "less_than_two_weeks", "more_than_two_weeks")

# dat$symptoms_durationBin <- as.factor(dat$symptoms_durationBin)

#

# dat <- dat %>% na.omit()

#

# trainIndex <- caret::createDataPartition(dat$symptoms_durationBin, p = .8,

# list = FALSE,

# times = 1)

#

# datTrain <- dat[ trainIndex,]

# datTest <- dat[-trainIndex,]

# full.model <- glm(symptoms_durationBin ~ ., data = datTrain,binomial())

# step.model <- MASS::stepAIC(full.model, direction = "backward", trace = FALSE)

# vects <- ls()

# spare <- c("dat","step.model")

# rem <- setdiff(vects, spare)

# rm(rem)

# dput(names(step.model$model))

data <- read.csv("/Volumes/Ahmed Shaheen/Old Files/long-covid-shaheen/app/app_data.csv")

symptoms_durationVars <- c("patient_country", "sex", "x5_1_hospitalization",

"infection_with_covid_19_after_vaccination", "x4_1_pre_existing_conditions_anemia",

"x4_1_pre_existing_conditions_hypertension_high_blood_pressure",

"x4_1_pre_existing_conditions_migraine", "x4_1_pre_existing_conditions_nightmares",

"x4_1_pre_existing_conditions_mold", "BMI", "aspirin", "naproxen",

"anti_oxidants", "azithromycin", "steroids", "x57_migraine",

"x6_increase_in_mood_symptoms_anxiety", "x12_smell_and_taste_symptoms_loss_of_smell",

"x16_insomnia_description_difficulty_falling_asleep", "x23_cvs_symptoms_tachycardia",

"x28_respiratory_symptoms_tightness_of_chest", "x28_respiratory_symptoms_dry_cough",

"x41_gastrointestinal_symptoms_abdominal_pain")

data <- data[,symptoms_durationVars] %>% mutate_if(is.character, ~as.factor(as.character(.))) %>% mutate_if(is.factor, ~as.numeric(as.factor(.)))

data$symptoms_durationBin <- as.factor(read.csv("/Volumes/Ahmed Shaheen/Old Files/long-covid-shaheen/app/app_data.csv")$symptoms_durationBin)

my_preprocess <- preProcess(data, method = c("center", "scale", "YeoJohnson"))

data <- predict(my_preprocess, data)

trainIndex <- caret::createDataPartition(data$symptoms_durationBin, p = .8, list = FALSE, times = 1)

Select variables

AutoML

## | | | 0% | |==== | 6%

## 17:48:25.299: AutoML: XGBoost is not available; skipping it. | |============ | 18% | |======================================================================| 100%

## AutoML Details

## ==============

## Project Name: AutoML_21_20240826_174825

## Leader Model ID: StackedEnsemble_BestOfFamily_1_AutoML_21_20240826_174825

## Algorithm: stackedensemble

##

## Total Number of Models Trained: 12

## Start Time: 2024-08-26 18:48:25 UTC

## End Time: 2024-08-26 18:48:30 UTC

## Duration: 5 s

##

## Leaderboard

## ===========

## model_id auc logloss

## 1 StackedEnsemble_BestOfFamily_1_AutoML_21_20240826_174825 0.7194840 0.5616247

## 2 StackedEnsemble_AllModels_1_AutoML_21_20240826_174825 0.7185105 0.5624902

## 3 GLM_1_AutoML_21_20240826_174825 0.7115854 0.5670435

## 4 GBM_1_AutoML_21_20240826_174825 0.7079611 0.5670370

## 5 GBM_grid_1_AutoML_21_20240826_174825_model_1 0.7072210 0.5674536

## 6 GBM_4_AutoML_21_20240826_174825 0.7015708 0.5711839

## 7 GBM_5_AutoML_21_20240826_174825 0.7002132 0.5716922

## 8 XRT_1_AutoML_21_20240826_174825 0.6988837 0.5924069

## 9 GBM_2_AutoML_21_20240826_174825 0.6901449 0.5772733

## 10 GBM_3_AutoML_21_20240826_174825 0.6879720 0.5794014

## aucpr mean_per_class_error rmse mse

## 1 0.8222532 0.4439287 0.4351509 0.1893563

## 2 0.8175900 0.4303512 0.4353550 0.1895340

## 3 0.8259215 0.4265342 0.4379798 0.1918263

## 4 0.8176265 0.4260443 0.4377577 0.1916318

## 5 0.8252996 0.4602359 0.4385401 0.1923174

## 6 0.8142209 0.4421901 0.4396807 0.1933191

## 7 0.8155357 0.4262057 0.4398929 0.1935058

## 8 0.8098212 0.4495550 0.4443889 0.1974815

## 9 0.8048534 0.4638971 0.4425997 0.1958945

## 10 0.8025228 0.4535335 0.4434381 0.1966374

##

## [12 rows x 7 columns]

## [1] "Best Best-AML Test AUC: 0.757081924995976"


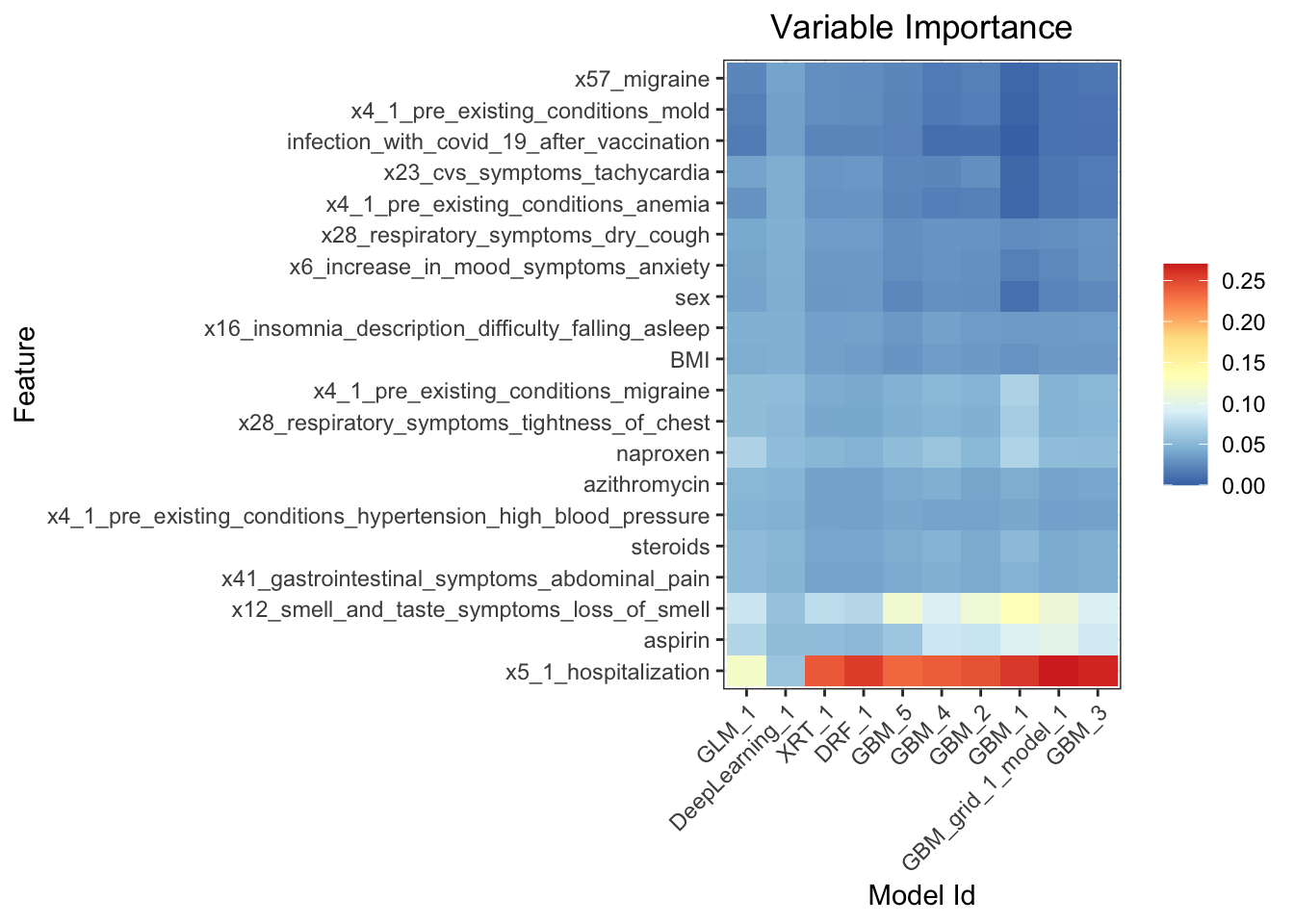

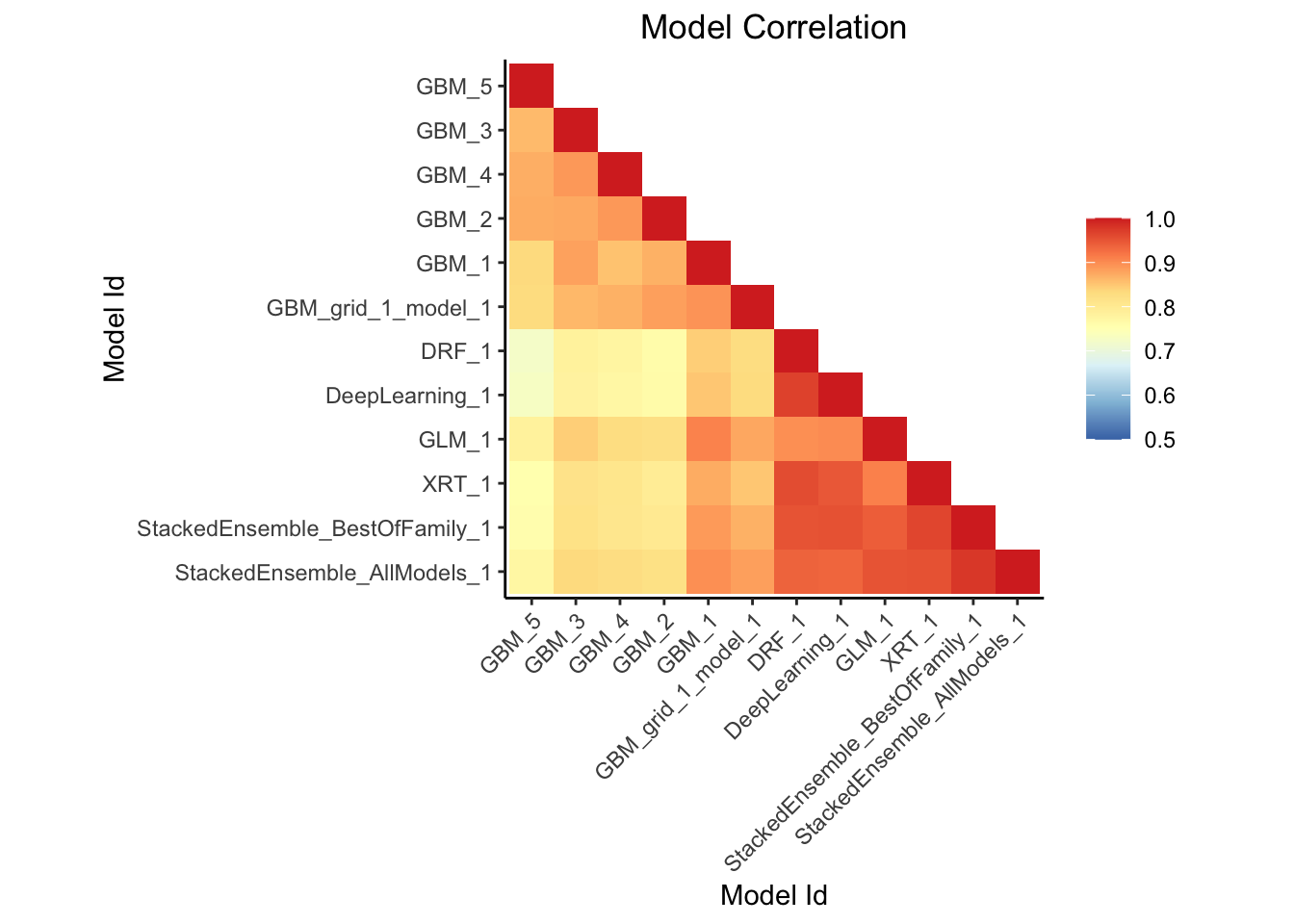


GMB

## | | | 0% | |======================================================================| 100%

## | | | 0% | |======================================================================| 100%

## [1] "Best Base-learner Test AUC: 0.756428054080155"

## [1] "Ensemble Test AUC: 0.757705617254145"

## [1] 0.7564281

#ROC curve for all the models


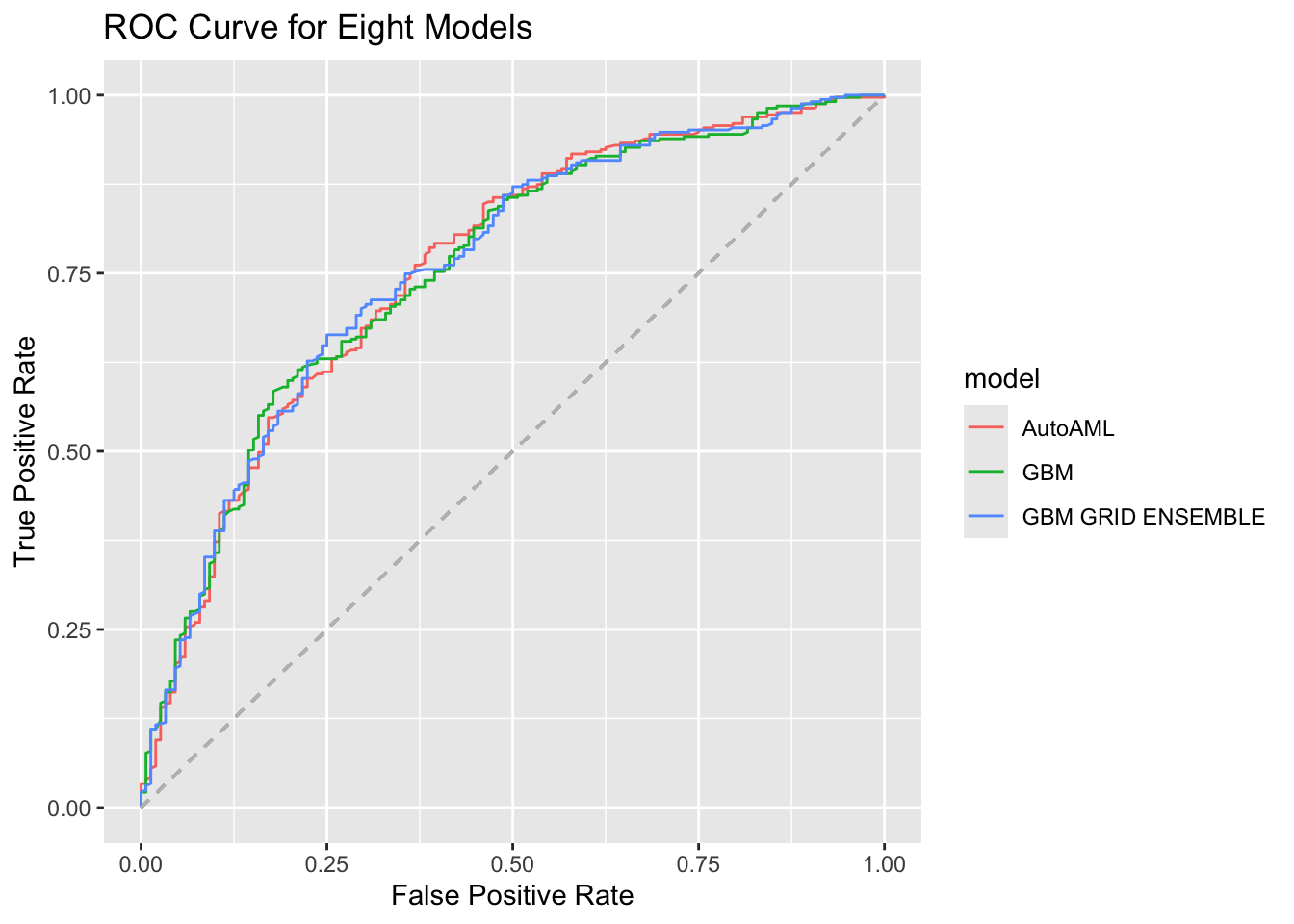


Table for model comparison

## models_names Index

## 1 GBM GRID ENSEMBLE 2

## # A tibble: 3 × 22

## threshold f1 f2 f0point5 accuracy precision recall specificity

## <dbl> <dbl> <dbl> <dbl> <dbl> <dbl> <dbl> <dbl>

## 1 0.520 0.839 0.884 0.798 0.760 0.773 0.917 0.421

## 2 0.457 0.834 0.899 0.779 0.743 0.745 0.948 0.303

## 3 0.550 0.833 0.892 0.781 0.743 0.75 0.936 0.329

## # ℹ 14 more variables: absolute_mcc <dbl>, min_per_class_accuracy <dbl>,

## # mean_per_class_accuracy <dbl>, tns <dbl>, fns <dbl>, fps <dbl>, tps <dbl>,

## # tnr <dbl>, fnr <dbl>, fpr <dbl>, tpr <dbl>, idx <int>, model_name <chr>,

## # AUC <dbl>

Save the best performing model

## [1] "/Volumes/Ahmed Shaheen/Old Files/long-covid-shaheen/Models/symptoms duration/final_grid_model_4"

Load the model

loaded_model <- h2o.loadModel(model_path)

Make predictions

## | | | 0% | |======================================================================| 100%

test$symptoms_durationBin

## symptoms_durationBin

## 1 less_than_two_weeks

## 2 less_than_two_weeks

## 3 less_than_two_weeks

## 4 less_than_two_weeks

## 5 less_than_two_weeks

## 6 less_than_two_weeks

##

## [479 rows x 1 column]

Brier Score

## [1] 0.1841417

Calibration

# Convert h2o frames to data frames

test_df <- as.data.frame(test)

prediction_df <- as.data.frame(prediction)

# Prepare data for calibration plot

calibration_data <- data.frame(

actual = test_df$symptoms_durationBin,

predicted = prediction_df$more_than_two_weeks

)

# Create bins

num_bins <- 10

calibration_data$bin <- cut(calibration_data$predicted,

breaks = seq(0, 1, length.out = num_bins + 1),

include.lowest = TRUE)

# Calculate mean predicted and actual values for each bin

calibration_summary <- calibration_data %>%

group_by(bin) %>%

summarize(

mean_predicted = mean(predicted),

mean_actual = mean(actual == "more_than_two_weeks"),

n = n()

)

# Create the calibration plot

ggplot(calibration_summary, aes(x = mean_predicted, y = mean_actual)) +

geom_point(aes(size = n), alpha = 0.7) +

geom_abline(intercept = 0, slope = 1, linetype = "dashed", color = "red") +

geom_smooth(method = "loess", se = FALSE, color = "blue") +

xlim(0, 1) + ylim(0, 1) +

labs(

x = "Mean Predicted Probability",

y = "Observed Fraction of Positives",

title = "Calibration Plot",

subtitle = "Perfect calibration represented by dashed red line"

) +

theme_minimal() +

theme(legend.position = "bottom")

## `geom_smooth()` using formula = 'y ~ x'

## Warning in simpleLoess(y, x, w, span, degree = degree, parametric = parametric,

## : Chernobyl! trL>n 6

## Warning in simpleLoess(y, x, w, span, degree = degree, parametric = parametric,

## : Chernobyl! trL>n 6

## Warning in sqrt(sum.squares/one.delta): NaNs produced


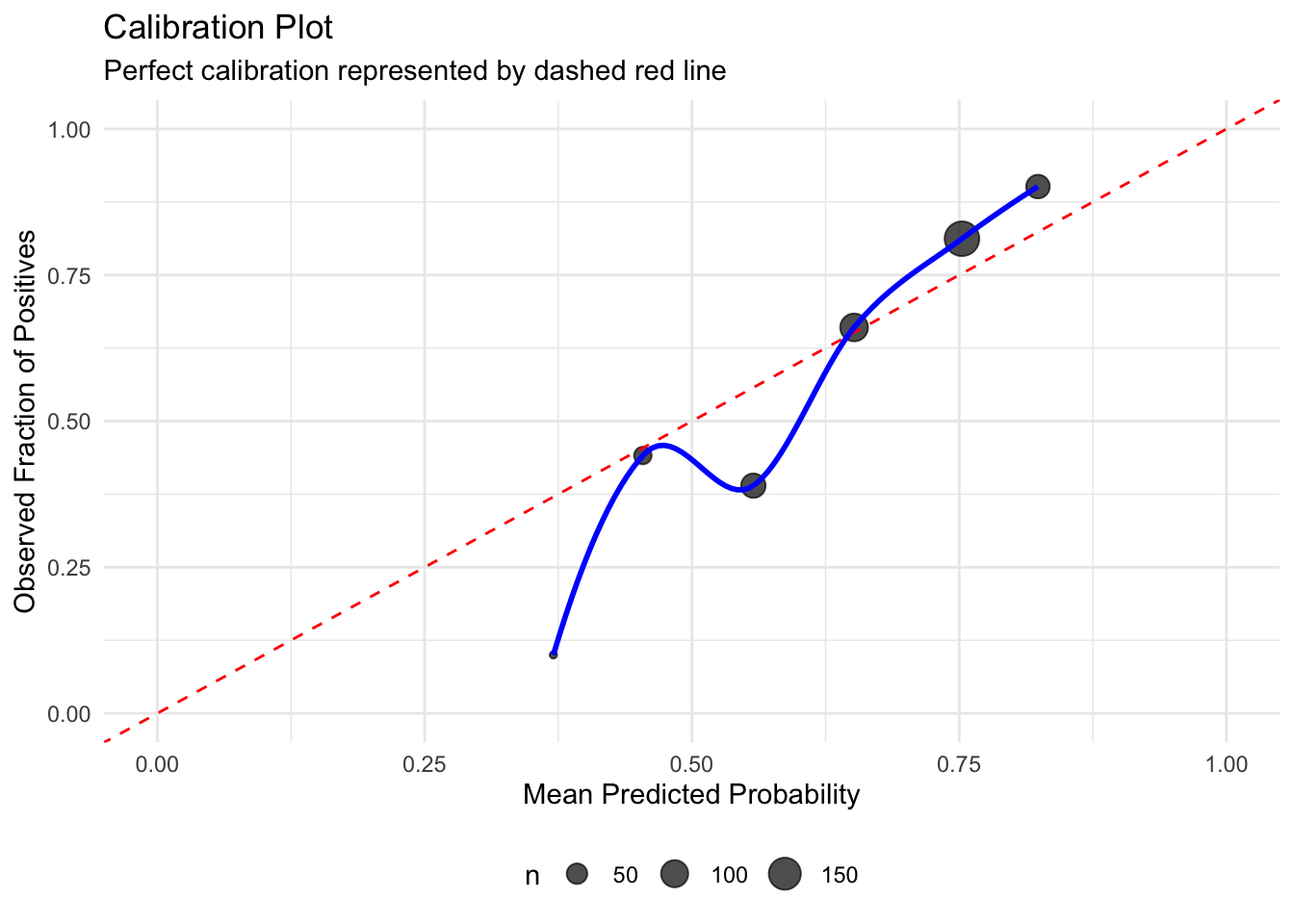


# Print the plot

print(last_plot())

## `geom_smooth()` using formula = 'y ~ x'

## Warning in simpleLoess(y, x, w, span, degree = degree, parametric = parametric,

## : Chernobyl! trL>n 6

## Warning in simpleLoess(y, x, w, span, degree = degree, parametric = parametric,

## : Chernobyl! trL>n 6

## Warning in sqrt(sum.squares/one.delta): NaNs produced


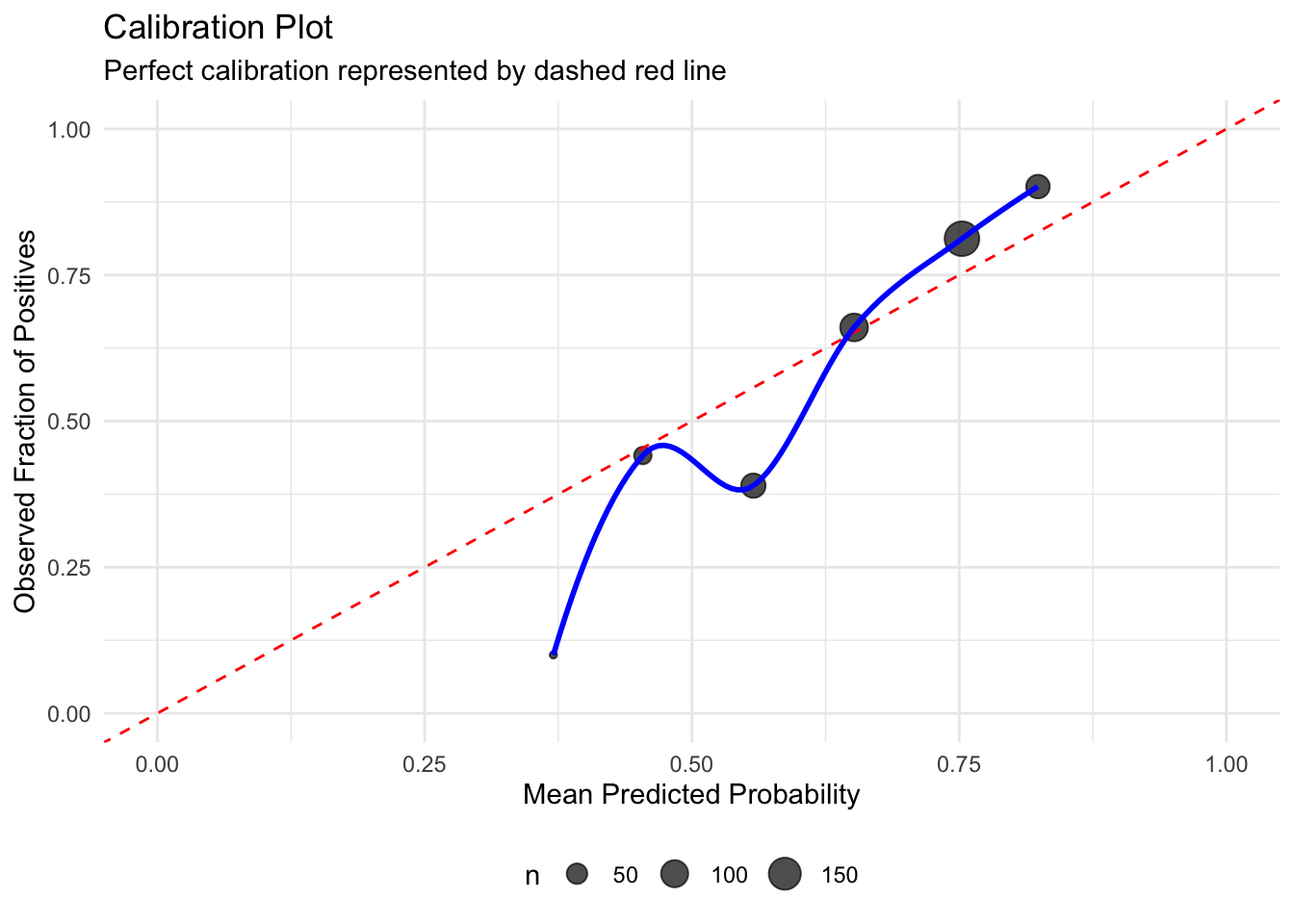


## Min. 1st Qu. Median Mean 3rd Qu. Max.

## 0.3247 0.5975 0.7119 0.6803 0.7734 0.8597

## [1] 479

## [1] 479

## threshold

## 1 0.7218288

## Min. 1st Qu. Median Mean 3rd Qu. Max.

## 0.3247 0.5975 0.7119 0.6803 0.7734 0.8597


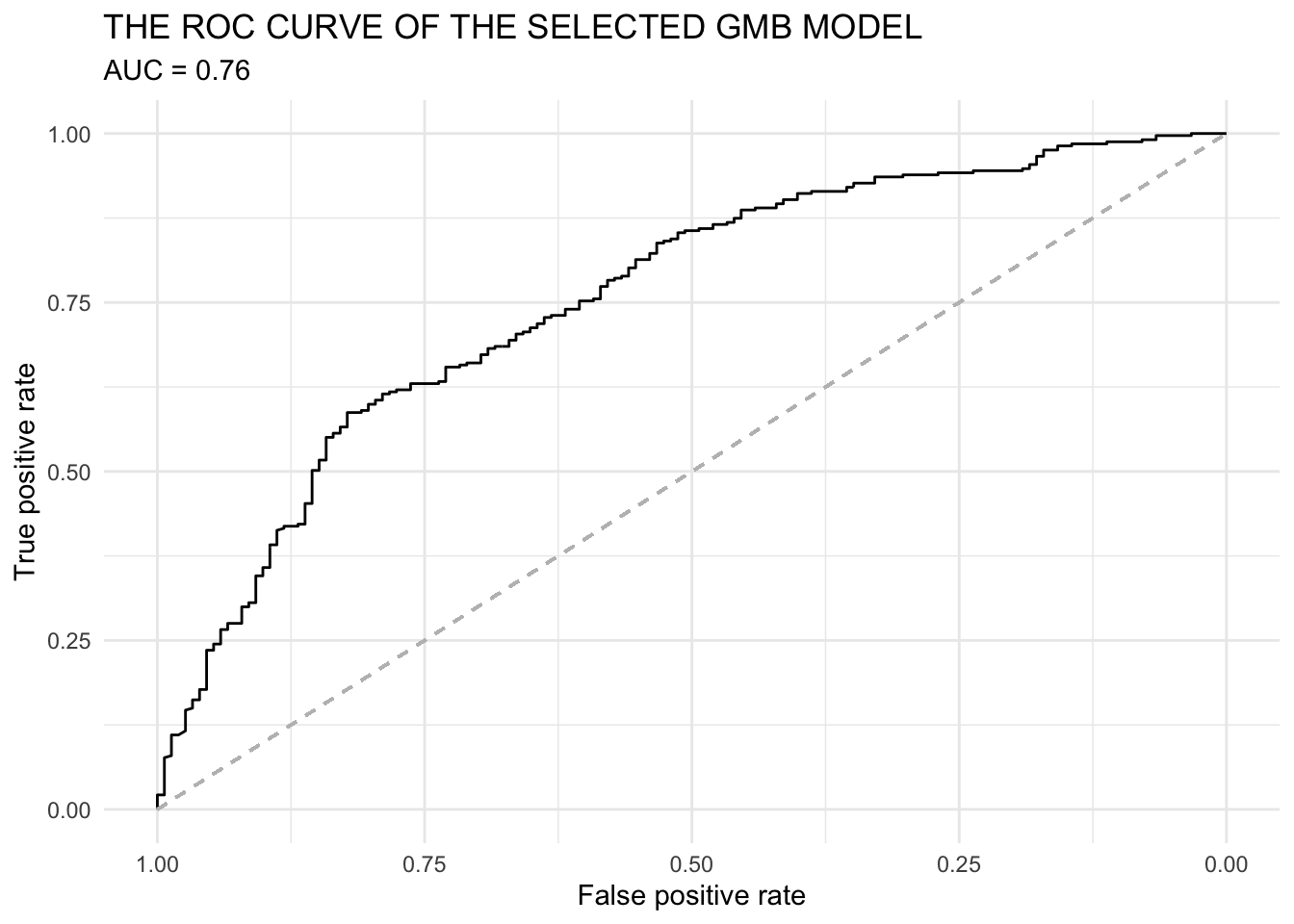


## Confusion Matrix and Statistics

##

## Reference

## Prediction less_than_two_weeks more_than_two_weeks

## less_than_two_weeks 125 135

## more_than_two_weeks 27 192

##

## Accuracy : 0.6618

## 95% CI : (0.6175, 0.7041)

## No Information Rate : 0.6827

## P-Value [Acc > NIR] : 0.8486

##

## Kappa : 0.3441

##

## Mcnemar's Test P-Value : <2e-16

##

## Sensitivity : 0.8224

## Specificity : 0.5872

## Pos Pred Value : 0.4808

## Neg Pred Value : 0.8767

## Prevalence : 0.3173

## Detection Rate : 0.2610

## Detection Prevalence : 0.5428

## Balanced Accuracy : 0.7048

##

## 'Positive' Class : less_than_two_weeks

##

## Reference

## Prediction less_than_two_weeks more_than_two_weeks

## less_than_two_weeks 125 135

## more_than_two_weeks 27 192


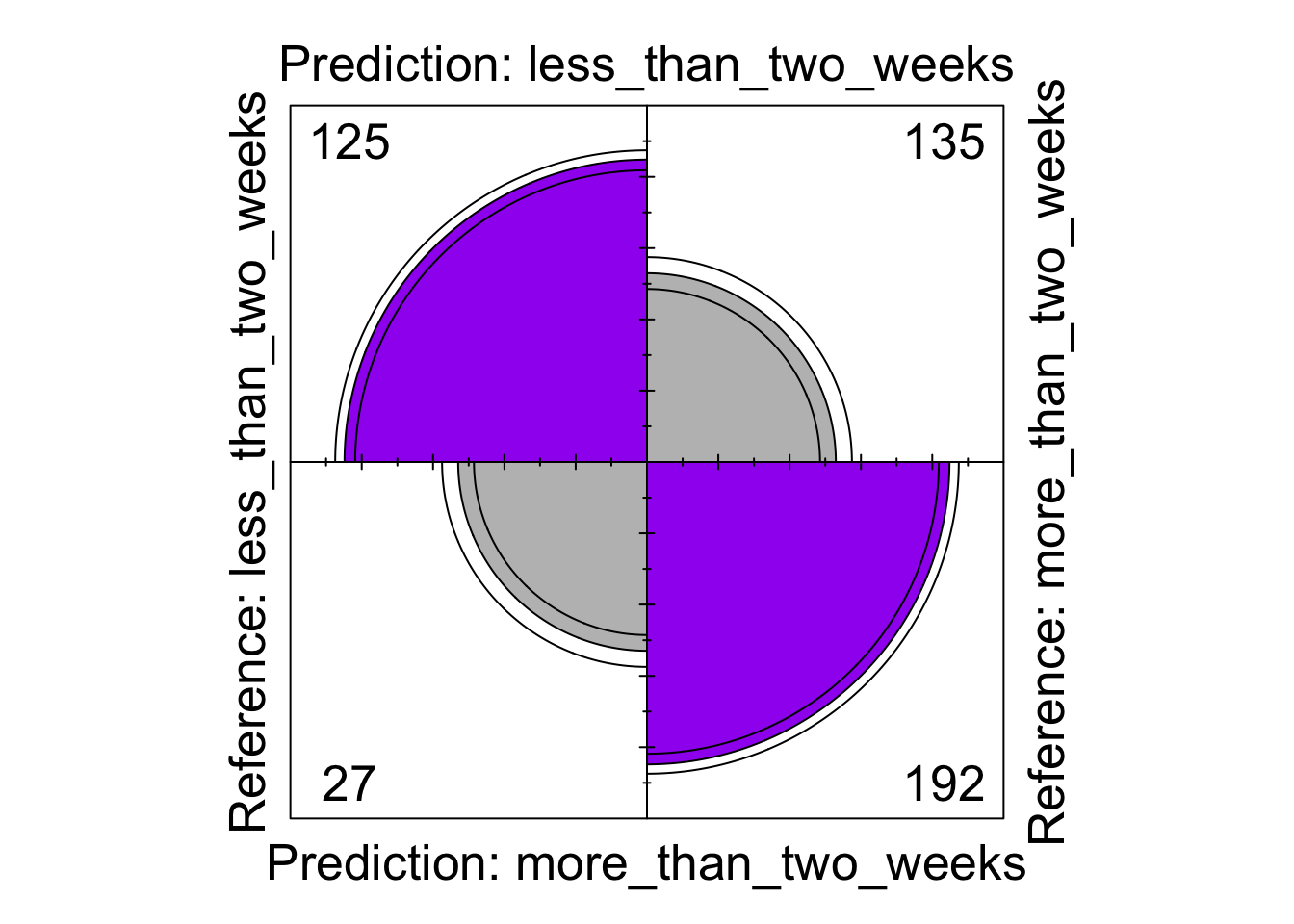


## Min. 1st Qu. Median Mean 3rd Qu. Max.

## 0.3247 0.5975 0.7119 0.6803 0.7734 0.8597


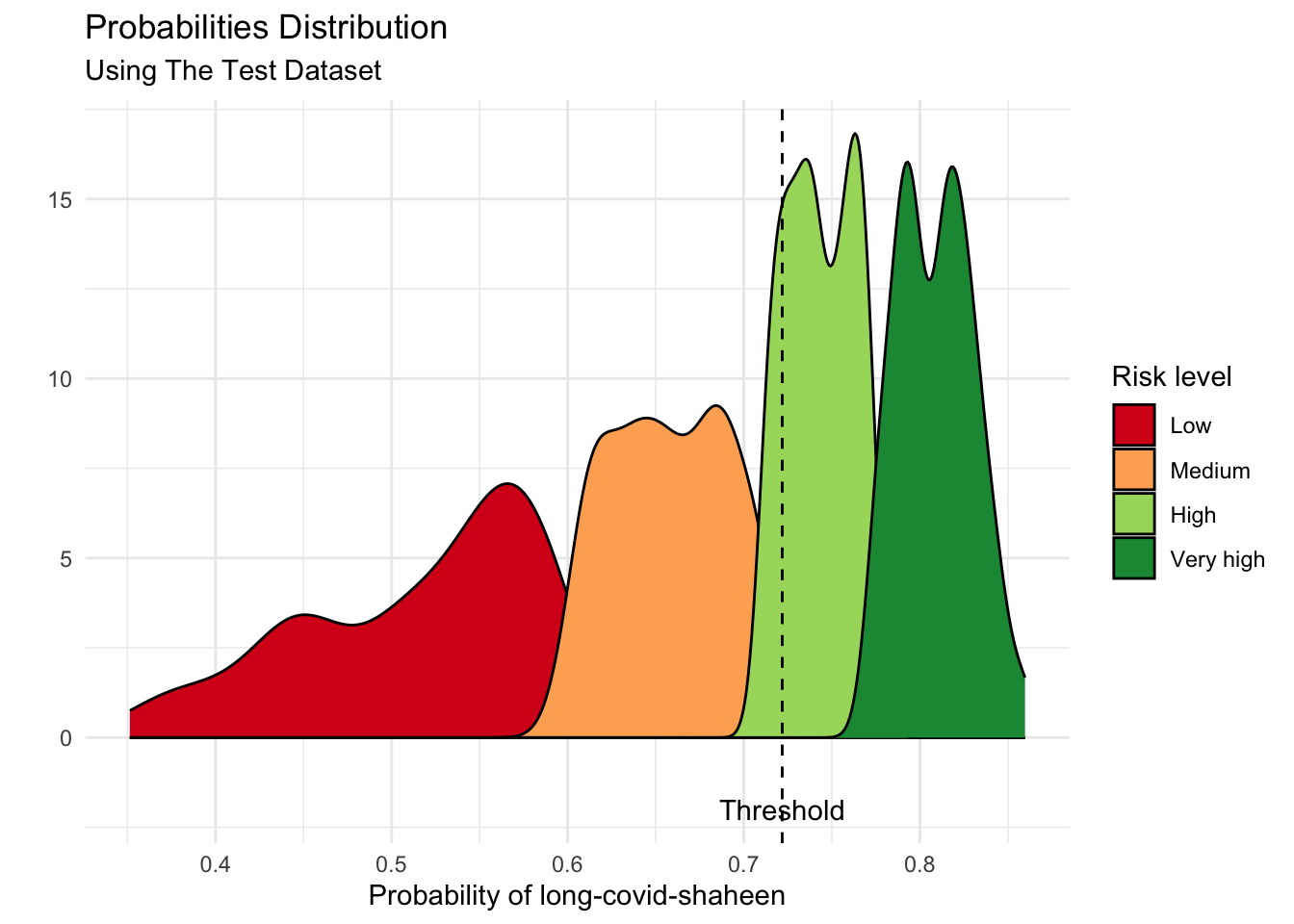


Metrics at different thresholds


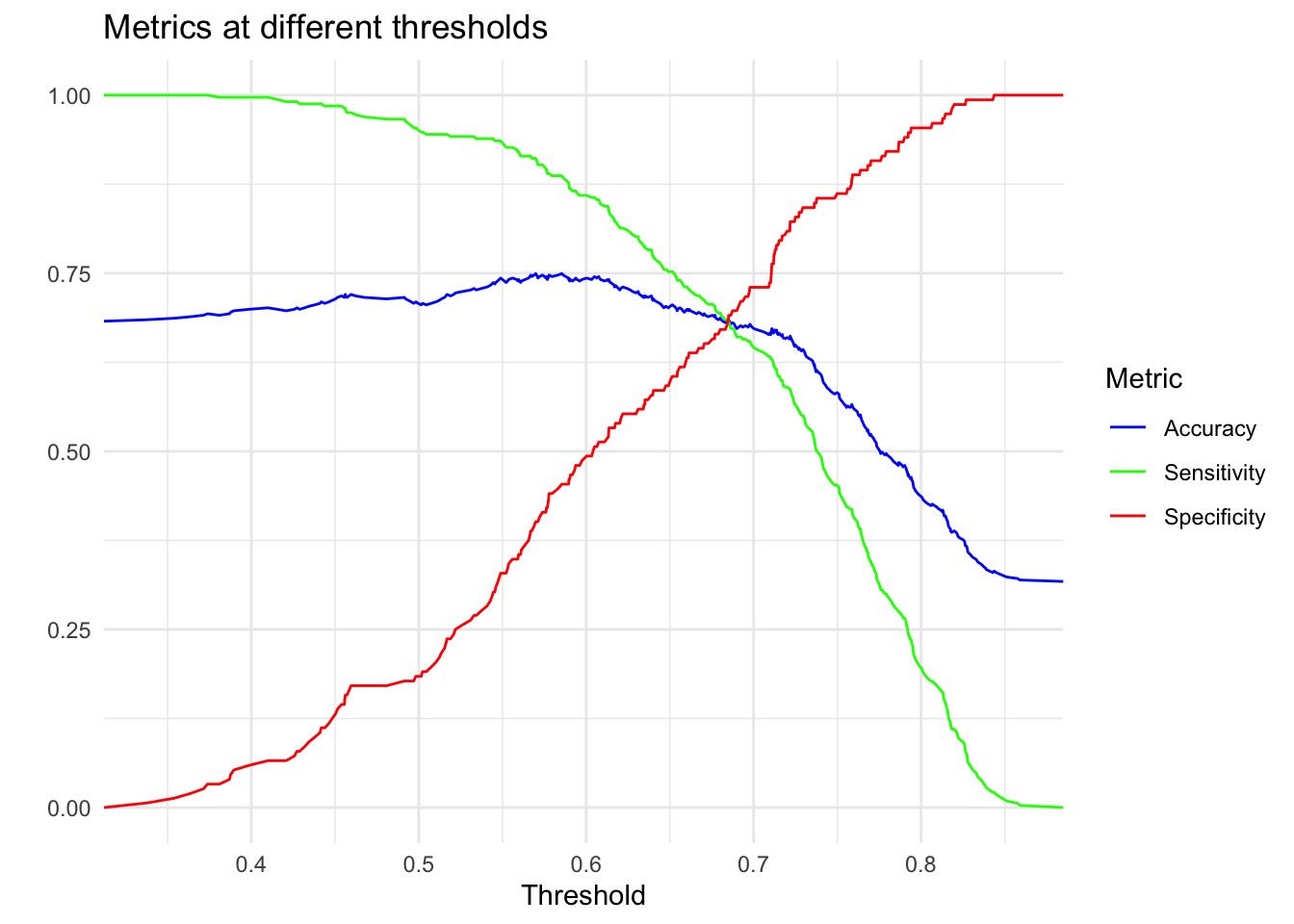


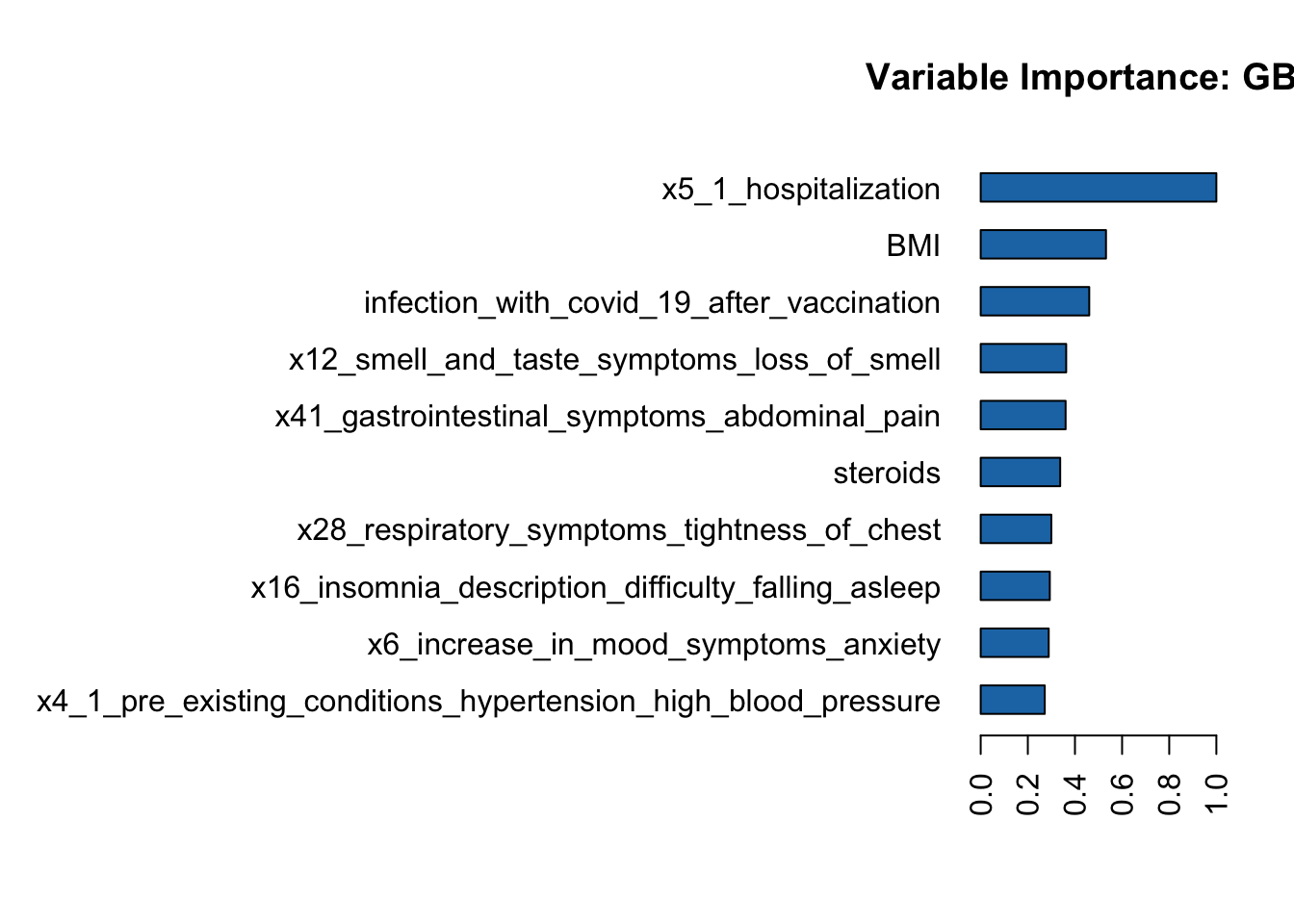


## variable

## 1 x5_1_hospitalization

## 2 BMI

## 3 infection_with_covid_19_after_vaccination

## 4 x12_smell_and_taste_symptoms_loss_of_smell

## 5 x41_gastrointestinal_symptoms_abdominal_pain

## 6 steroids

## 7 x28_respiratory_symptoms_tightness_of_chest

## 8 x16_insomnia_description_difficulty_falling_asleep

## 9 x6_increase_in_mood_symptoms_anxiety

## 10 x4_1_pre_existing_conditions_hypertension_high_blood_pressure

## 11 x23_cvs_symptoms_tachycardia

## 12 x28_respiratory_symptoms_dry_cough

## 13 azithromycin

## 14 aspirin

## 15 sex

## 16 x57_migraine

## 17 naproxen

## 18 x4_1_pre_existing_conditions_mold

## 19 x4_1_pre_existing_conditions_anemia

## 20 x4_1_pre_existing_conditions_migraine

## 21 x4_1_pre_existing_conditions_nightmares

## 22 anti_oxidants

## relative_importance scaled_importance percentage

## 1 157.811691 1.00000000 0.181089563

## 2 83.907242 0.53169218 0.096283904

## 3 72.693199 0.46063253 0.083415744

## 4 57.248276 0.36276321 0.065692631

## 5 56.865688 0.36033888 0.065253610

## 6 53.238224 0.33735285 0.061091080

## 7 47.308167 0.29977606 0.054286315

## 8 46.281818 0.29327243 0.053108576

## 9 45.544136 0.28859798 0.052262083

## 10 42.900631 0.27184698 0.049228650

## 11 36.093845 0.22871465 0.041417836

## 12 34.501919 0.21862714 0.039591093

## 13 32.663231 0.20697599 0.037481191

## 14 19.307198 0.12234327 0.022155088

## 15 18.064718 0.11447009 0.020729338

## 16 13.396529 0.08488933 0.015372572

## 17 12.090228 0.07661174 0.013873586

## 18 11.333157 0.07181443 0.013004844

## 19 10.782144 0.06832284 0.012372554

## 20 8.877810 0.05625571 0.010187323

## 21 6.010090 0.03808393 0.006896603

## 22 4.536642 0.02874719 0.005205815


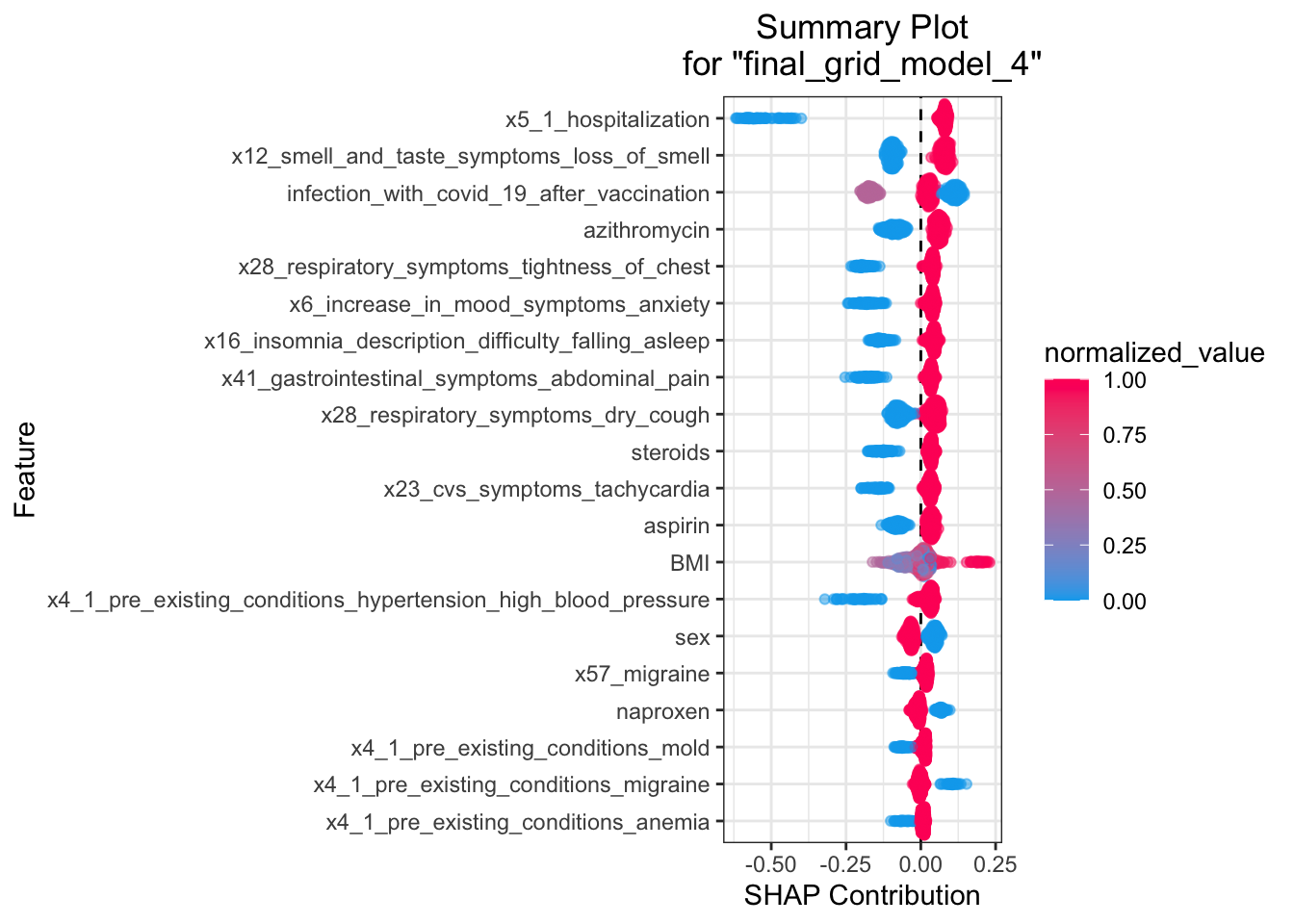


## Gains/Lift Table: Avg response rate: 68.16 %, avg score: 68.13 %

## group cumulative_data_fraction lower_threshold lift cumulative_lift

## 1 1 0.01094320 0.845401 1.467125 1.467125

## 2 2 0.02032308 0.840937 1.222604 1.354270

## 3 3 0.03022408 0.834597 1.467125 1.391240

## 4 4 0.04116727 0.831479 1.327399 1.374269

## 5 5 0.05002606 0.828514 1.380824 1.375430

## 6 6 0.10005211 0.811911 1.360147 1.367789

## 7 7 0.15059927 0.798218 1.240250 1.324982

## 8 8 0.20010422 0.785810 1.235474 1.302838

## 9 9 0.30015633 0.763474 1.260811 1.288829

## 10 10 0.40020844 0.737550 1.237887 1.276093

## 11 11 0.50026055 0.711263 1.123268 1.245528

## 12 12 0.59979156 0.679328 0.944798 1.195624

## 13 13 0.69984367 0.639892 0.947518 1.160154

## 14 14 0.79989578 0.582992 0.794693 1.114442

## 15 15 0.89994789 0.499460 0.649509 1.062753

## 16 16 1.00000000 0.223785 0.435553 1.000000

## response_rate score cumulative_response_rate cumulative_score

## 1 1.000000 0.851800 1.000000 0.851800

## 2 0.833333 0.843225 0.923077 0.847842

## 3 1.000000 0.837113 0.948276 0.844328

## 4 0.904762 0.832608 0.936709 0.841212

## 5 0.941176 0.829526 0.937500 0.839143

## 6 0.927083 0.819584 0.932292 0.829364

## 7 0.845361 0.803846 0.903114 0.820799

## 8 0.842105 0.793444 0.888021 0.814031

## 9 0.859375 0.775191 0.878472 0.801085

## 10 0.843750 0.750395 0.869792 0.788412

## 11 0.765625 0.724930 0.848958 0.775716

## 12 0.643979 0.696463 0.814944 0.762564

## 13 0.645833 0.659479 0.790767 0.747827

## 14 0.541667 0.612343 0.759609 0.730880

## 15 0.442708 0.544059 0.724378 0.710110

## 16 0.296875 0.422268 0.681605 0.681311

## capture_rate cumulative_capture_rate gain cumulative_gain

## 1 0.016055 0.016055 46.712538 46.712538

## 2 0.011468 0.027523 22.260449 35.426958

## 3 0.014526 0.042049 46.712538 39.123959

## 4 0.014526 0.056575 32.739916 37.426935

## 5 0.012232 0.068807 38.082389 37.543005

## 6 0.068043 0.136850 36.014749 36.778877

## 7 0.062691 0.199541 24.025032 32.498175

## 8 0.061162 0.260703 23.547401 30.283790

## 9 0.126147 0.386850 26.081088 28.882889

## 10 0.123853 0.510703 23.788704 27.609343

## 11 0.112385 0.623089 12.326787 24.552832

## 12 0.094037 0.717125 -5.520198 19.562433

## 13 0.094801 0.811927 -5.248152 16.015425

## 14 0.079511 0.891437 -20.530708 11.444182

## 15 0.064985 0.956422 -35.049137 6.275267

## 16 0.043578 1.000000 -56.444715 0.000000

## kolmogorov_smirnov

## 1 0.016055

## 2 0.022613

## 3 0.037139

## 4 0.048392

## 5 0.058987

## 6 0.115574

## 7 0.153715

## 8 0.190327

## 9 0.272284

## 10 0.347037

## 11 0.385773

## 12 0.368517

## 13 0.352025

## 14 0.287509

## 15 0.177371

## 16 0.000000

##

##

## Confusion Matrix

## ================

##

## > Confusion matrix shows a predicted class vs an actual class.

##

##

##

## final_grid_model_4

## ------------------

##

## | | less_than_two_weeks | more_than_two_weeks | Error | Rate

## |:---:|:---:|:---:|:---:|:---:|

## | **less_than_two_weeks** |50 | 102 | 0.671052631578947 | =102/152 |

## | **more_than_two_weeks** |21 | 306 | 0.0642201834862385 | =21/327 |

## | **Totals** |71 | 408 | 0.25678496868476 | =123/479 |

##

##

## Learning Curve Plot

## ===================

##

## > Learning curve plot shows the loss function/metric dependent on number of iterations or trees for tree-based algorithms. This plot can be useful for determining whether the model overfits.


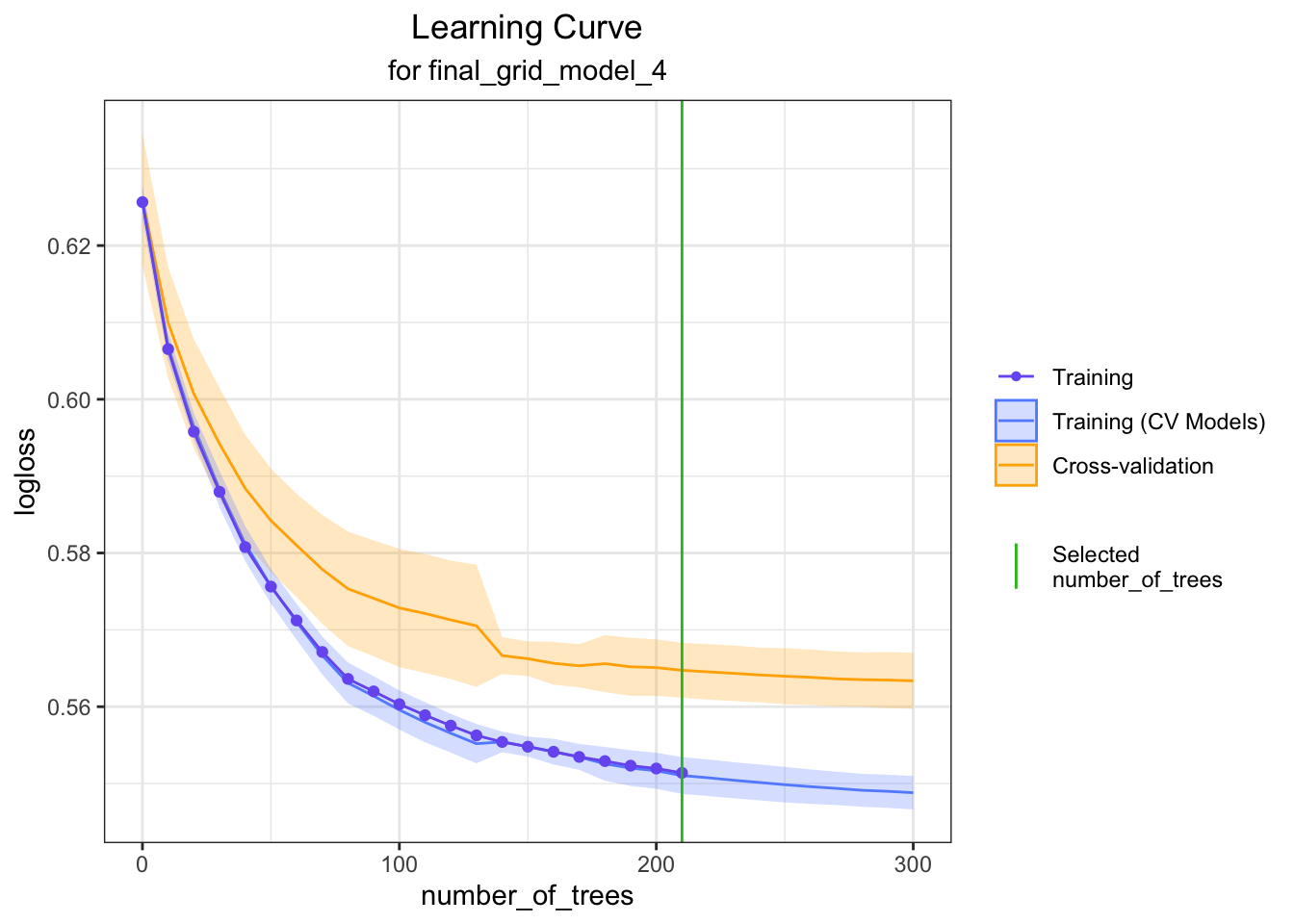


##

##

## Variable Importance

## ===================

##

## > The variable importance plot shows the relative importance of the most important variables in the model.


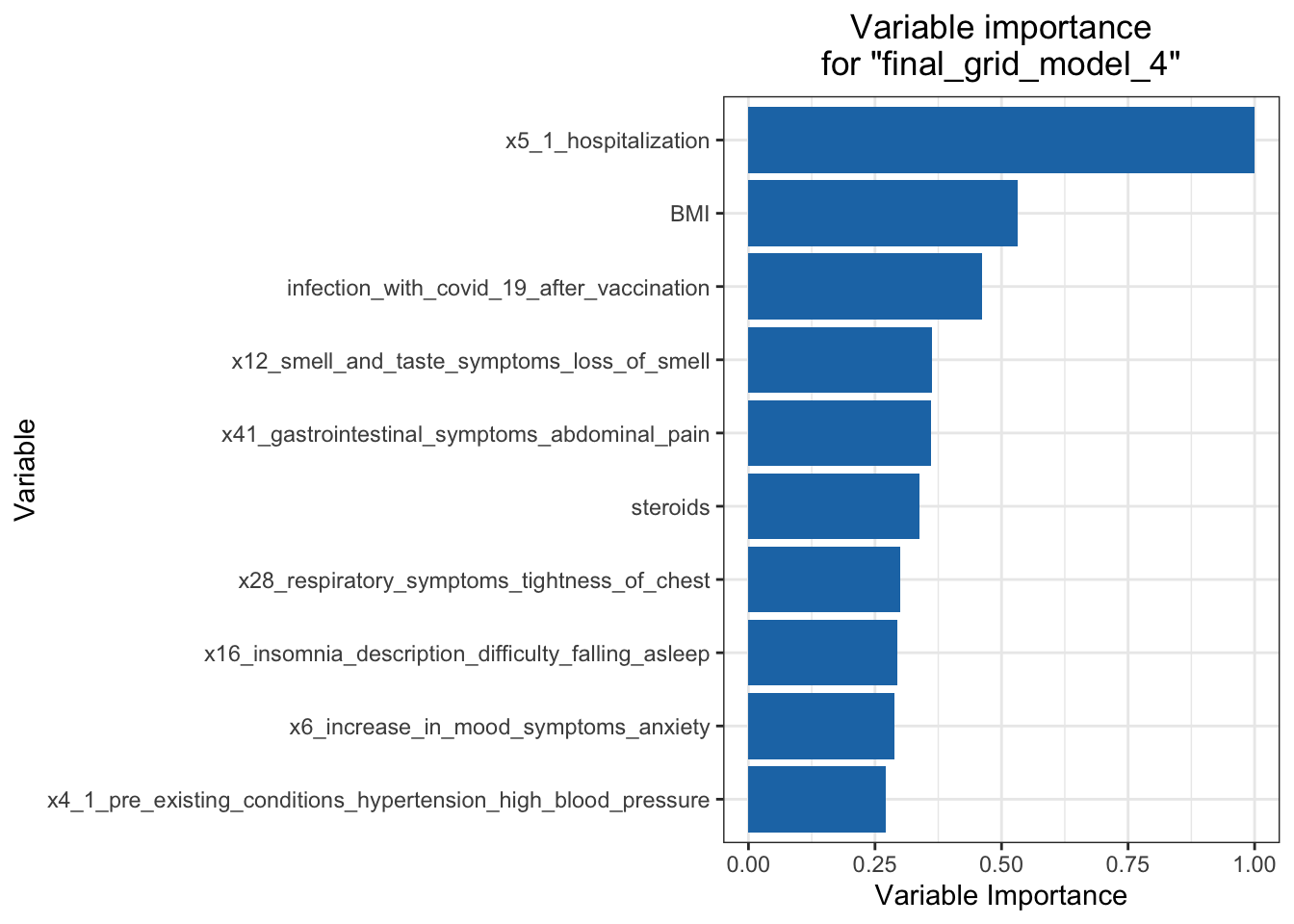


##

##

## SHAP Summary

## ============

##

## > SHAP summary plot shows the contribution of the features for each instance (row of data). The sum of the feature contributions and the bias term is equal to the raw prediction of the model, i.e., prediction before applying inverse link function.


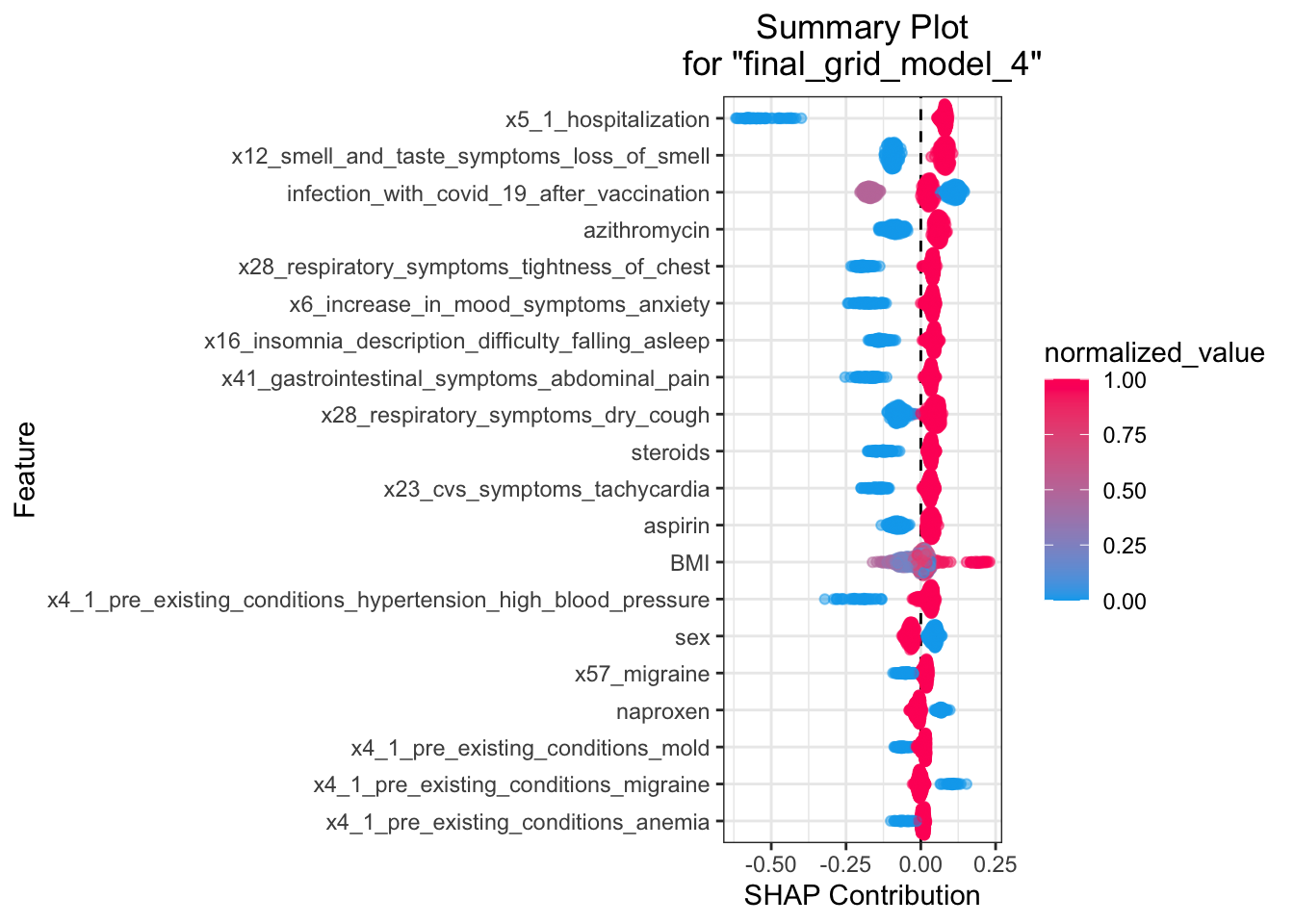


##

##

## Partial Dependence Plots

## ========================

##

## > Partial dependence plot (PDP) gives a graphical depiction of the marginal effect of a variable on the response. The effect of a variable is measured in change in the mean response. PDP assumes independence between the feature for which is the PDP computed and the rest.


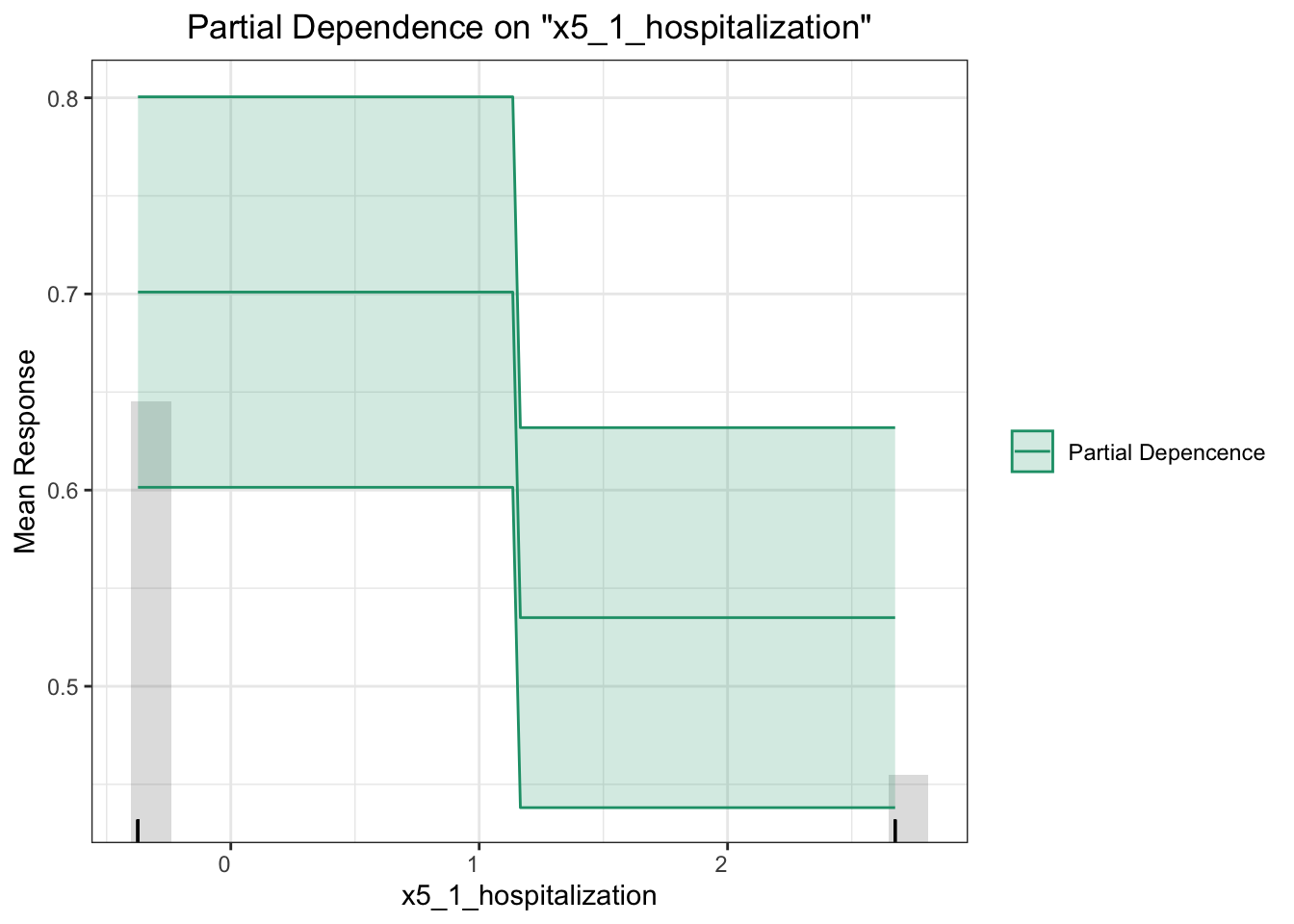

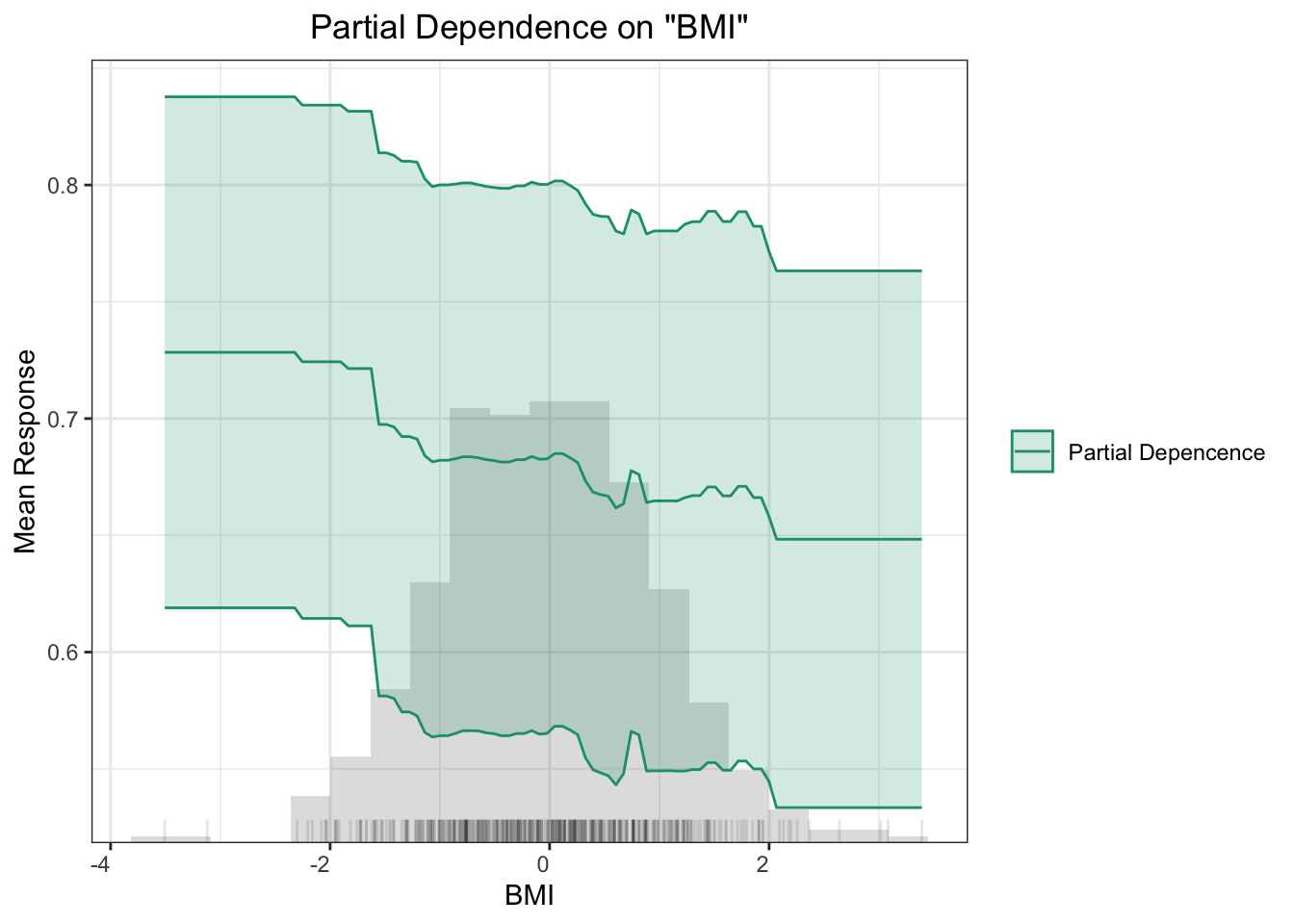

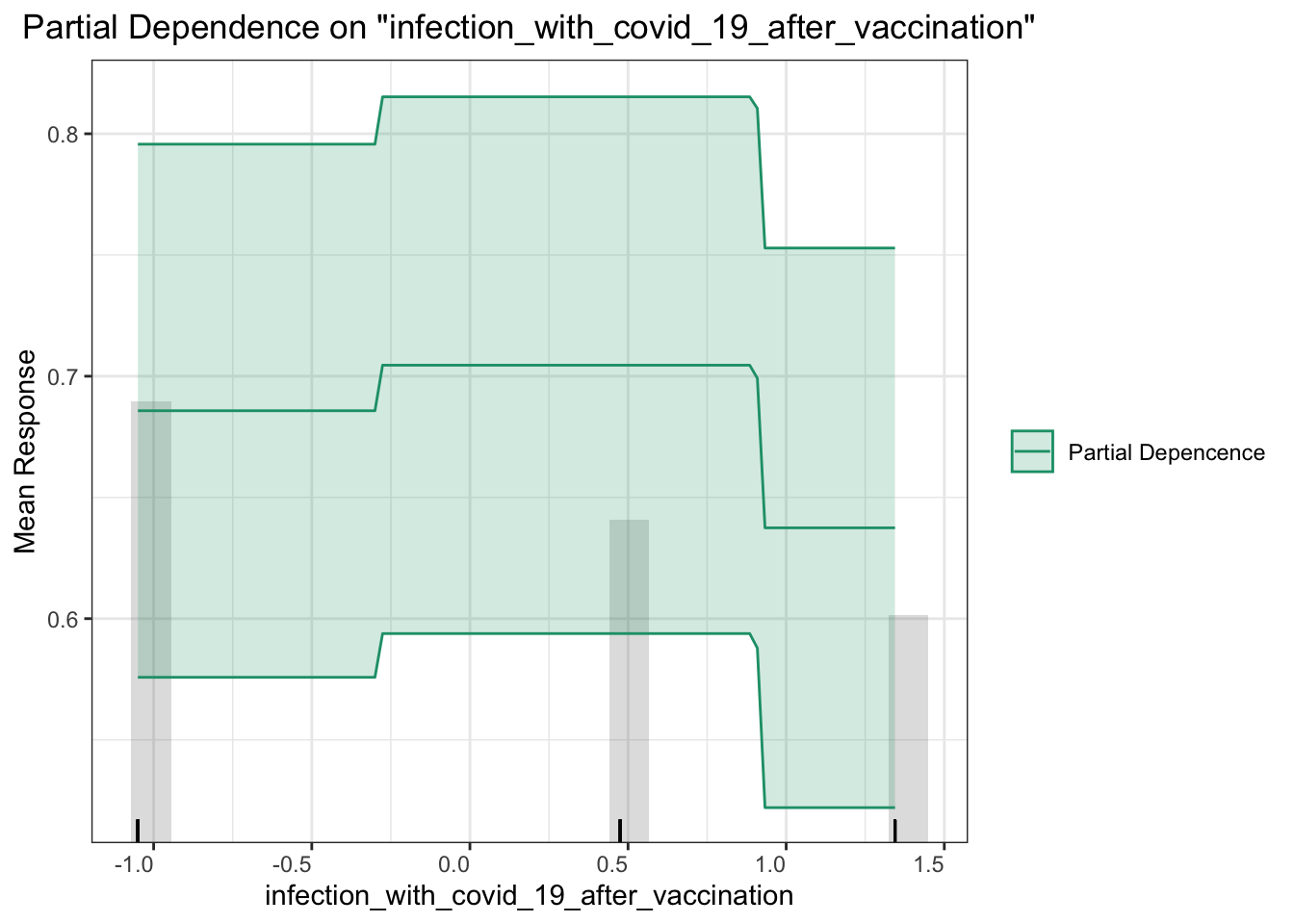

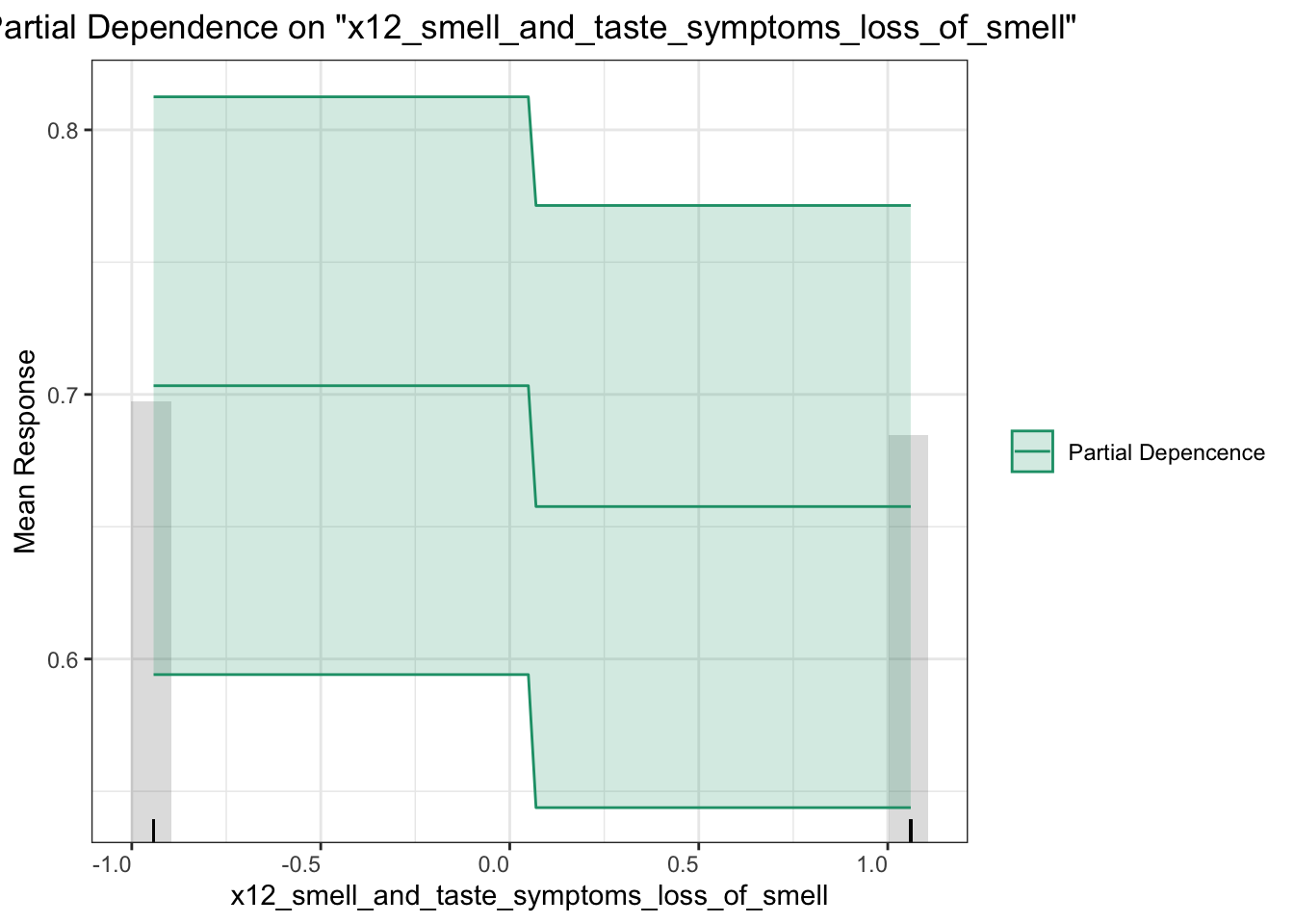

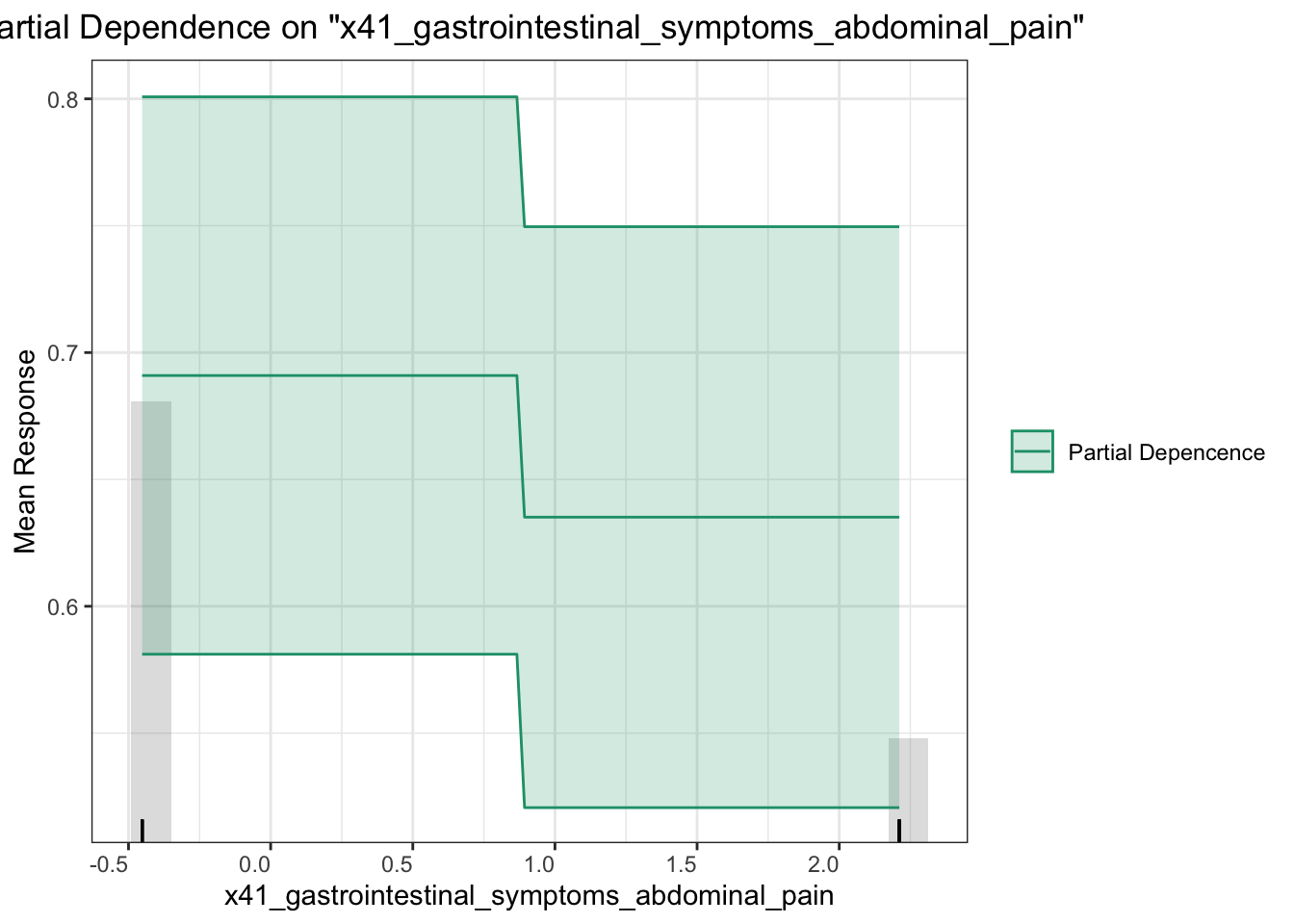


# Machine learning: fatigue

Load libraries

Get the data for analysis

# dfAnalysis <- read.csv("/Volumes/Ahmed Shaheen/Old Files/long-covid-shaheen/model_data.csv")[,-1]

#

# # Convert them to factors using lapply

# dfAnalysis[names(dfAnalysis %>% select(!c("BMI")))] <- lapply(dfAnalysis[names(dfAnalysis %>% select(!c("BMI")))], as.factor)

#

#

# levels(dfAnalysis$age_group) <- c("18-29", "30-39", "40-49", "50-59","> 60")

# levels(dfAnalysis$infection_with_covid_19_after_vaccination) <- c("Not vaccinated", "No","Yes")

#

# levels(dfAnalysis$household_income) <- c("More than $10,000","Don’t know/Not sure", "Less than $10,000")

Get the outcomes data

# outcomes <- read.csv("/Volumes/Ahmed Shaheen/Old Files/long-covid-shaheen/df1_outcomes.csv")[-1] %>%

# select(!c("x52_dizziness_or_vertigo_symptoms","x56_tinnitus_symptoms",

# "x32_muscle_and_joint_symptoms_muscle_and_joint_issues",

# "x10_headaches_symptoms_diffuse_entire_brain","x26_gastrointestinal_symptom_abdominal_pain",

# "x3_memory_loss_symptoms_i_e_remembering_a_phone_number_before_writing_it_down",

# "x22_temperature_symptoms_other_temperature_issues_not_listed_above_or_below",

# "x24_generic_symptoms_dizziness_vertigo_unsteadiness_or_balance_issues"))

#

# outcomes$depression <- factor(outcomes$depression,

# levels=c("Mild", "Moderate", "Moderately severe", "None", "Severe"),

# labels = c("Mild", "Moderate", "ModeratelySevere", "None", "Severe"))

#

#

# outcomes$depression <- as.character(outcomes$depression)

#

# outcomes$depressionBin[outcomes$depression %in% c("Mild", "Moderate", "ModeratelySevere", "Severe")] <- "Yes"

#

# outcomes$depressionBin[outcomes$depression %in% c("None")] <- "No"

#

# outcomes$depression <- as.factor(outcomes$depression)

#

# outcomes$depressionBin <- as.factor(outcomes$depressionBin)

#

# # Convert them to factors using lapply

# outcomes[names(outcomes %>% select(!c("x53_vertigo_dizziness_severity")))] <- lapply(outcomes[names(outcomes %>% select(!c("x53_vertigo_dizziness_severity")))], factor)

#

# Embeddings <- read.csv("/Volumes/Ahmed Shaheen/Old Files/long-covid-shaheen/dataWithembddings.csv")

# Data <- read.csv("/Volumes/Ahmed Shaheen/Old Files/long-covid-shaheen/dfAnalysis.csv")

#

# longCovid <- Data$longCovid

# encoder_output <- Data$layer_output

# embeddings <- Embeddings$X0

# symptoms_duration <- Data$symptoms_duration

# set.seed(123)

# dat <- dfAnalysis %>% select(!c("covid_19_vaccination",

# "household_income","vaccine_shots_n"))

#

# dat <- cbind(dat,outcomes[,1:46]) %>%

# mutate_if(is.factor, ~as.numeric(as.factor(.)))

#

#

# my_preprocess <- preProcess(dat, method = c("center", "scale", "YeoJohnson"))

# dat <- predict(my_preprocess, dat)

#

# #Remove highly correlated variables

# cor_matrix <- cor(dat)

# cor_matrix_rm <- cor_matrix

# cor_matrix_rm[upper.tri(cor_matrix_rm)] <- 0

# diag(cor_matrix_rm) <- 0

#

# dat <- dat[ , !apply(cor_matrix_rm, 2, function(x) any(x > 0.99))]

# heatmap(cor(dat))

#

#

# dat <- dat %>% select(!names(dat)[nearZeroVar(dat)])

#

# dat$fatigue <- outcomes$chronic_fatigue

#

# dat <- dat %>% na.omit()

#

# trainIndex <- caret::createDataPartition(dat$fatigue, p = .8,

# list = FALSE,

# times = 1)

#

# datTrain <- dat[ trainIndex,]

# datTest <- dat[-trainIndex,]

# full.model <- glm(fatigue ~ ., data = datTrain,binomial())

# step.model <- MASS::stepAIC(full.model, direction = "backward", trace = FALSE)

# vects <- ls()

# spare <- c("dat","step.model")

# rem <- setdiff(vects, spare)

# rm(rem)

# dput(names(step.model$model))

data <- read.csv("/Volumes/Ahmed Shaheen/Old Files/long-covid-shaheen/app/app_data.csv")

fatigueVars <- c("sex", "x44_before_covid_health_status", "x45_before_covid_mental_health_diagnsosis",

"x49_before_covid_vertigo_dizziness", "x4_7_smoking", "x5_1_hospitalization",

"type_vaccine", "infection_with_covid_19_after_vaccination",

"x4_1_pre_existing_conditions_vision_near_sighted_far_sighted",

"x4_1_pre_existing_conditions_vitamin_d_deficiency",

"x4_1_pre_existing_conditions_diabetes_type_2",

"x4_1_pre_existing_conditions_insomnia", "x4_1_pre_existing_conditions_nightmares",

"x4_1_pre_existing_conditions_mold", "aspirin", "paracetamol",

"naproxen", "anti_oxidants", "anti_type_one_histamine", "omega_3",

"x3_memory_loss_symptoms_or_forgetting_you_re_in_the_middle_of_a_task",

"x5_brain_fog_symptoims_poor_attention_or_concentration", "x6_increase_in_mood_symptoms_anxiety",

"x6_increase_in_mood_symptoms_depression", "x6_increase_in_mood_symptoms_anger",

"x6_increase_in_mood_symptoms_irritability",

"x12_smell_and_taste_symptoms_altered_sense_of_smell",

"x16_insomnia_description_difficulty_falling_asleep",

"x23_cvs_symptoms_thumping_or_skipping_beats",

"x26_gastrointestinal_symptom_loss_of_appetite", "x28_respiratory_symptoms_dry_cough",

"x32_muscle_and_joint_symptoms_muscle_aches")

data <- data[,fatigueVars] %>% mutate_if(is.character, ~as.factor(as.character(.))) %>% mutate_if(is.factor, ~as.numeric(as.factor(.)))

data$fatigue <- as.factor(read.csv("/Volumes/Ahmed Shaheen/Old Files/long-covid-shaheen/app/app_data.csv")$fatigue)

my_preprocess <- preProcess(data, method = c("center", "scale", "YeoJohnson"))

data <- predict(my_preprocess, data)

trainIndex <- caret::createDataPartition(data$fatigue, p = .8, list = FALSE, times = 1)

Select variables

AutoML

## | | | 0% | |== | 3%

## 17:55:03.886: AutoML: XGBoost is not available; skipping it. | |======== | 12% | |============ | 18% | |=================== | 26% | |======================================================================| 100%

## AutoML Details

## ==============

## Project Name: AutoML_23_20240826_175503

## Leader Model ID: StackedEnsemble_AllModels_1_AutoML_23_20240826_175503

## Algorithm: stackedensemble

##

## Total Number of Models Trained: 12

## Start Time: 2024-08-26 18:55:04 UTC

## End Time: 2024-08-26 18:55:13 UTC

## Duration: 9 s

##

## Leaderboard

## ===========

## model_id auc logloss

## 1 StackedEnsemble_AllModels_1_AutoML_23_20240826_175503 0.9511339 0.2818350

## 2 GBM_2_AutoML_23_20240826_175503 0.9468814 0.2939419

## 3 StackedEnsemble_BestOfFamily_1_AutoML_23_20240826_175503 0.9466987 0.2931912

## 4 GBM_5_AutoML_23_20240826_175503 0.9465786 0.2946837

## 5 GBM_grid_1_AutoML_23_20240826_175503_model_1 0.9463034 0.2941701

## 6 GBM_3_AutoML_23_20240826_175503 0.9460843 0.2962196

## 7 GBM_4_AutoML_23_20240826_175503 0.9435397 0.3029947

## 8 GBM_1_AutoML_23_20240826_175503 0.9386897 0.3168195

## 9 DRF_1_AutoML_23_20240826_175503 0.9222654 0.3926303

## 10 XRT_1_AutoML_23_20240826_175503 0.9200296 0.3943224

## aucpr mean_per_class_error rmse mse

## 1 0.9526241 0.1209367 0.2944493 0.08670039

## 2 0.9482889 0.1257558 0.3012593 0.09075717

## 3 0.9478982 0.1247570 0.3011630 0.09069918

## 4 0.9484311 0.1251457 0.3018437 0.09110965

## 5 0.9465626 0.1261891 0.3009660 0.09058052

## 6 0.9474322 0.1352340 0.3031361 0.09189152

## 7 0.9436690 0.1185150 0.3037444 0.09226069

## 8 0.9378119 0.1285772 0.3128281 0.09786144

## 9 0.9113952 0.1568274 0.3474362 0.12071194

## 10 0.9129911 0.1524418 0.3491660 0.12191687

##

## [12 rows x 7 columns]

## [1] "Best Best-AML Test AUC: 0.864307315668203"


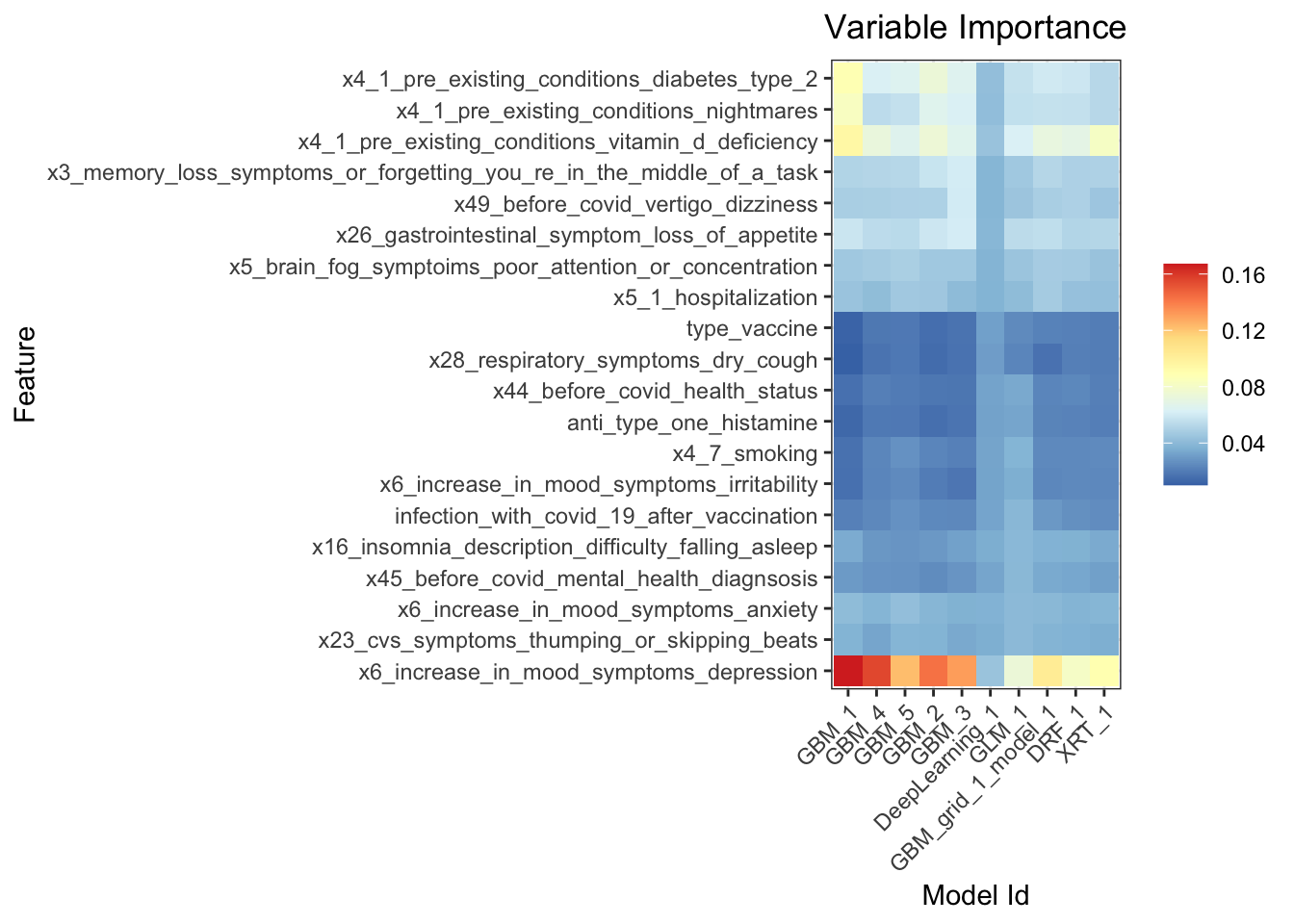

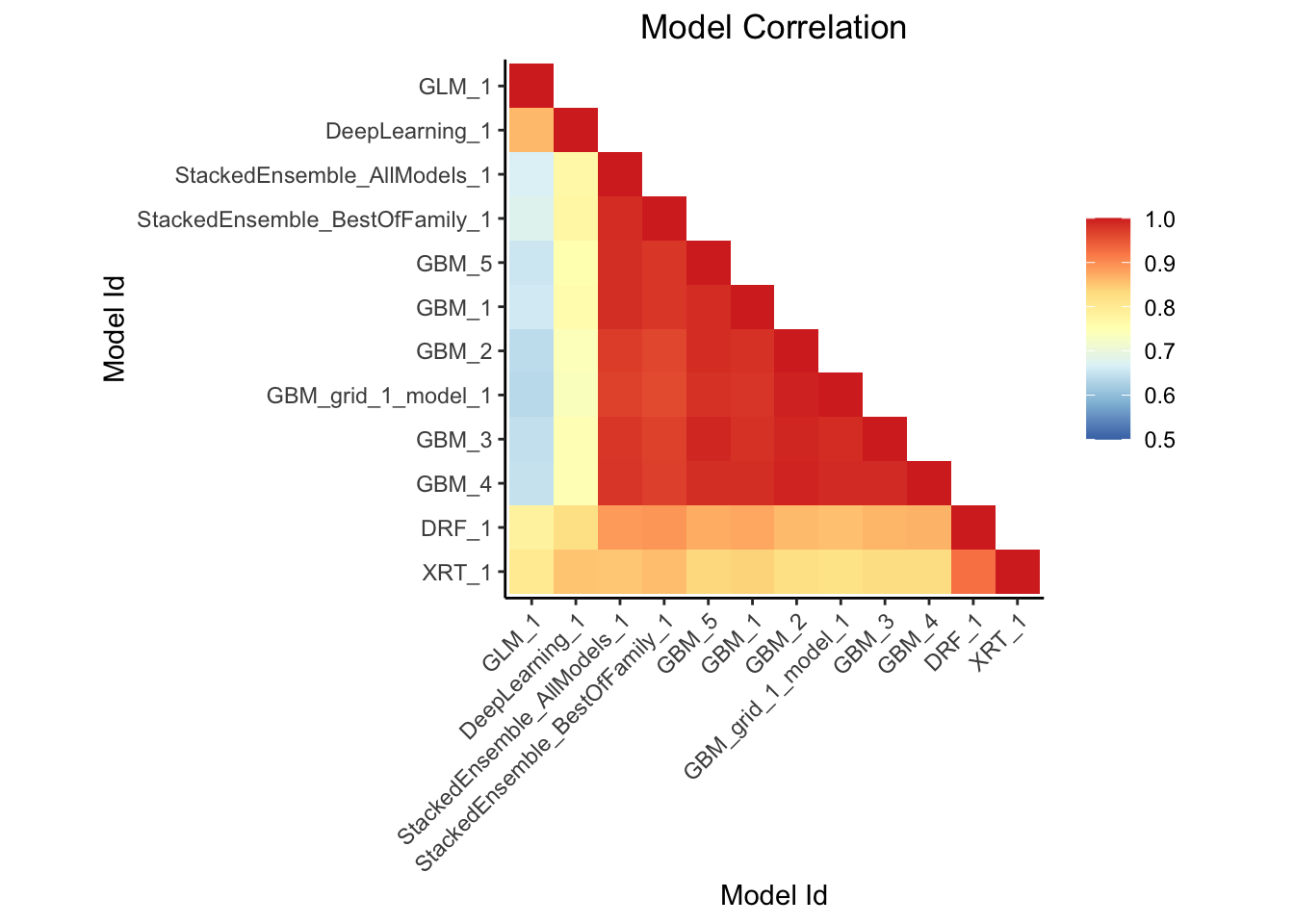


GMB

## | | | 0% | |======================================================================| 100%

## | | | 0% | |======================================================================| 100%

## [1] "Best Base-learner Test AUC: 0.868735599078341"

## [1] "Ensemble Test AUC: 0.858618951612903"

## [1] 0.8687356

#ROC curve for all the models


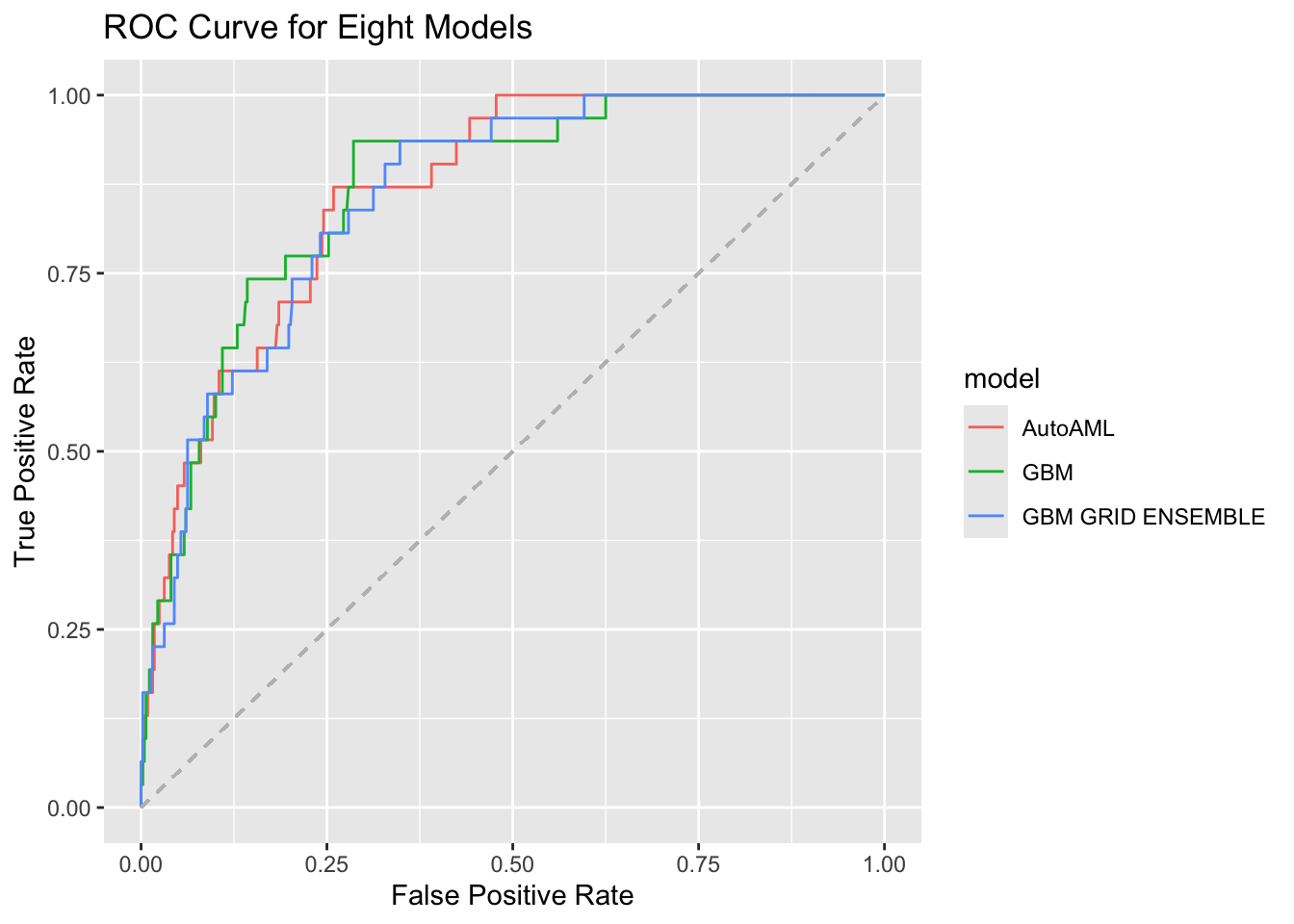


Table for model comparison

## models_names Index

## 1 GBM 3

## # A tibble: 3 × 22

## threshold f1 f2 f0point5 accuracy precision recall specificity

## <dbl> <dbl> <dbl> <dbl> <dbl> <dbl> <dbl> <dbl>

## 1 0.268 0.418 0.438 0.4 0.919 0.389 0.452 0.951

## 2 0.389 0.427 0.476 0.386 0.910 0.364 0.516 0.938

## 3 0.539 0.4 0.518 0.326 0.875 0.290 0.645 0.891

## # ℹ 14 more variables: absolute_mcc <dbl>, min_per_class_accuracy <dbl>,

## # mean_per_class_accuracy <dbl>, tns <dbl>, fns <dbl>, fps <dbl>, tps <dbl>,

## # tnr <dbl>, fnr <dbl>, fpr <dbl>, tpr <dbl>, idx <int>, model_name <chr>,

## # AUC <dbl>

Save the best performing model

## [1] "/Volumes/Ahmed Shaheen/Old Files/long-covid-shaheen/Models/fatigue/final_grid_model_1"

Load the model

loaded_model <- h2o.loadModel(model_path)

Make predictions

## | | | 0% | |======================================================================| 100%

Brier Score

## [1] 0.1362375

Calibration

# Convert h2o frames to data frames

test_df <- as.data.frame(test)

prediction_df <- as.data.frame(prediction)

# Prepare data for calibration plot

calibration_data <- data.frame(

actual = test_df$fatigue,

predicted = prediction_df$Yes

)

# Create bins

num_bins <- 10

calibration_data$bin <- cut(calibration_data$predicted,

breaks = seq(0, 1, length.out = num_bins + 1),

include.lowest = TRUE)

# Calculate mean predicted and actual values for each bin

calibration_summary <- calibration_data %>%

group_by(bin) %>%

summarize(

mean_predicted = mean(predicted),

mean_actual = mean(actual == "Yes"),

n = n()

)

# Create the calibration plot

ggplot(calibration_summary, aes(x = mean_predicted, y = mean_actual)) +

geom_point(aes(size = n), alpha = 0.7) +

geom_abline(intercept = 0, slope = 1, linetype = "dashed", color = "red") +

geom_smooth(method = "loess", se = FALSE, color = "blue") +

xlim(0, 1) + ylim(0, 1) +

labs(

x = "Mean Predicted Probability",

y = "Observed Fraction of Positives",

title = "Calibration Plot",

subtitle = "Perfect calibration represented by dashed red line"

) +

theme_minimal() +

theme(legend.position = "bottom")

## `geom_smooth()` using formula = 'y ~ x'

## Warning: Removed 1 row containing missing values or values outside the scale range

## (`geom_smooth()`).


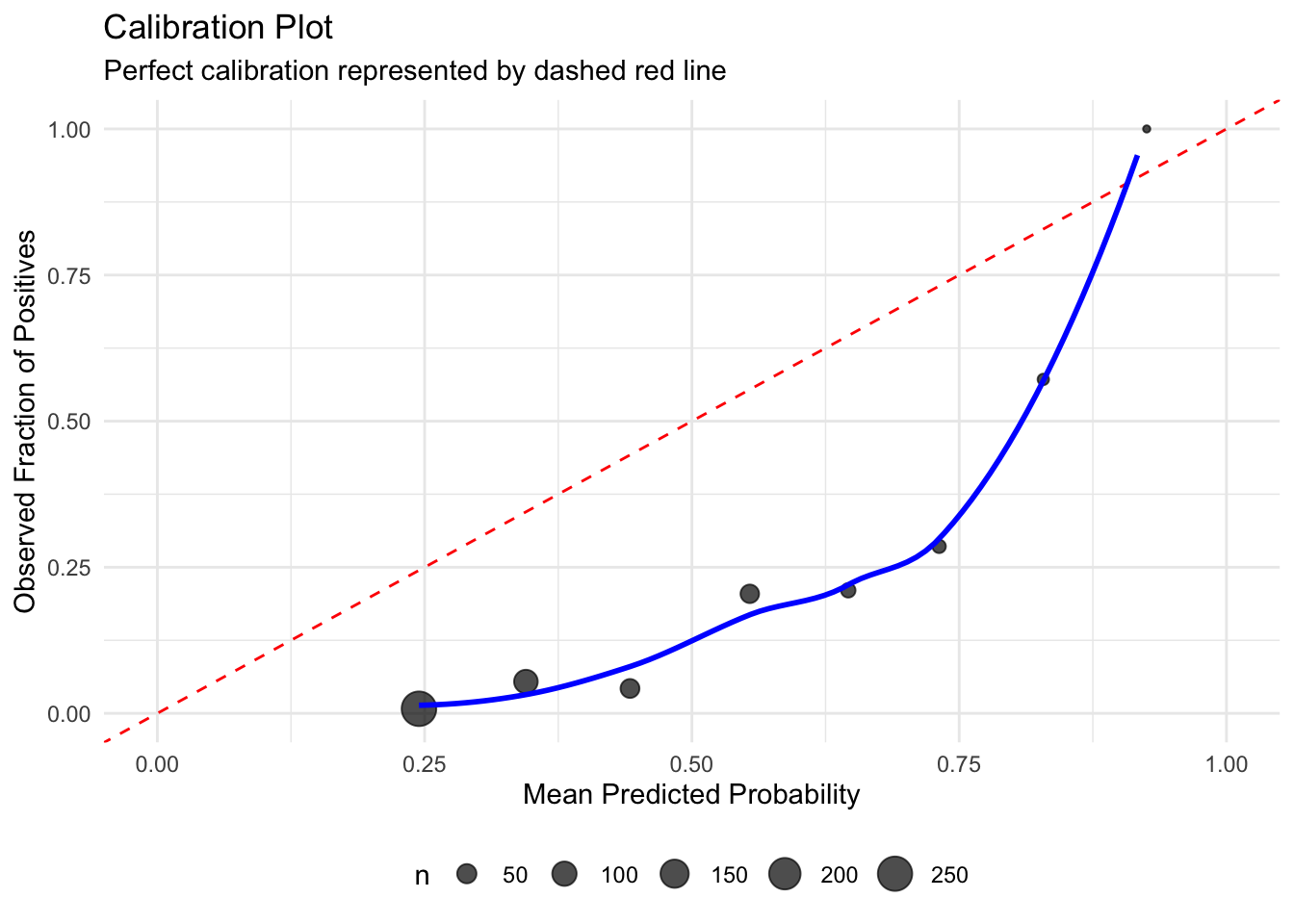


# Print the plot

print(last_plot())

## `geom_smooth()` using formula = 'y ~ x'

## Warning: Removed 1 row containing missing values or values outside the scale range

## (`geom_smooth()`).


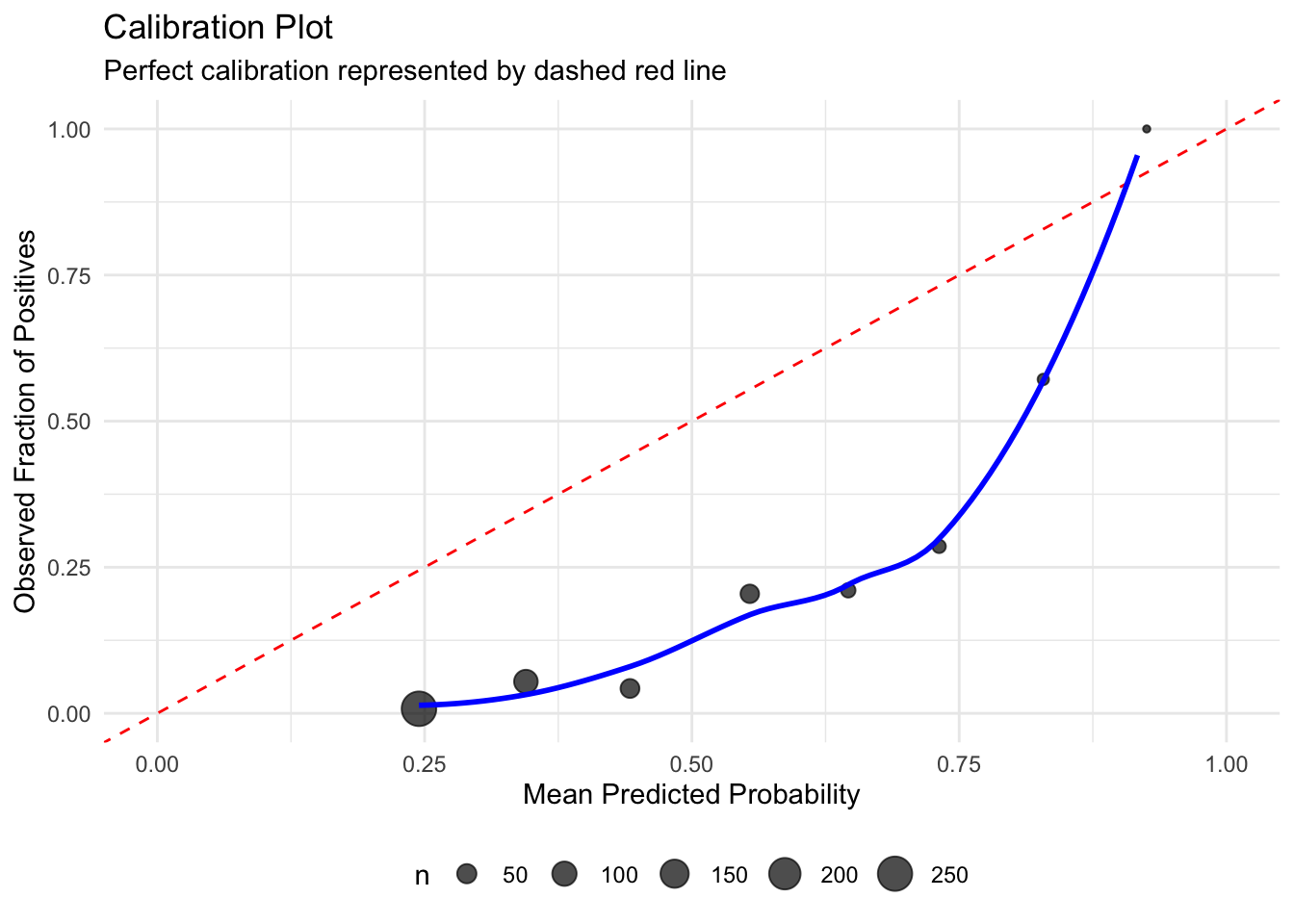


## Min. 1st Qu. Median Mean 3rd Qu. Max.

## 0.2259 0.2303 0.2871 0.3518 0.4232 0.9254

## [1] 479

## [1] 479

## threshold

## 1 0.3688798

## Min. 1st Qu. Median Mean 3rd Qu. Max.

## 0.2259 0.2303 0.2871 0.3518 0.4232 0.9254


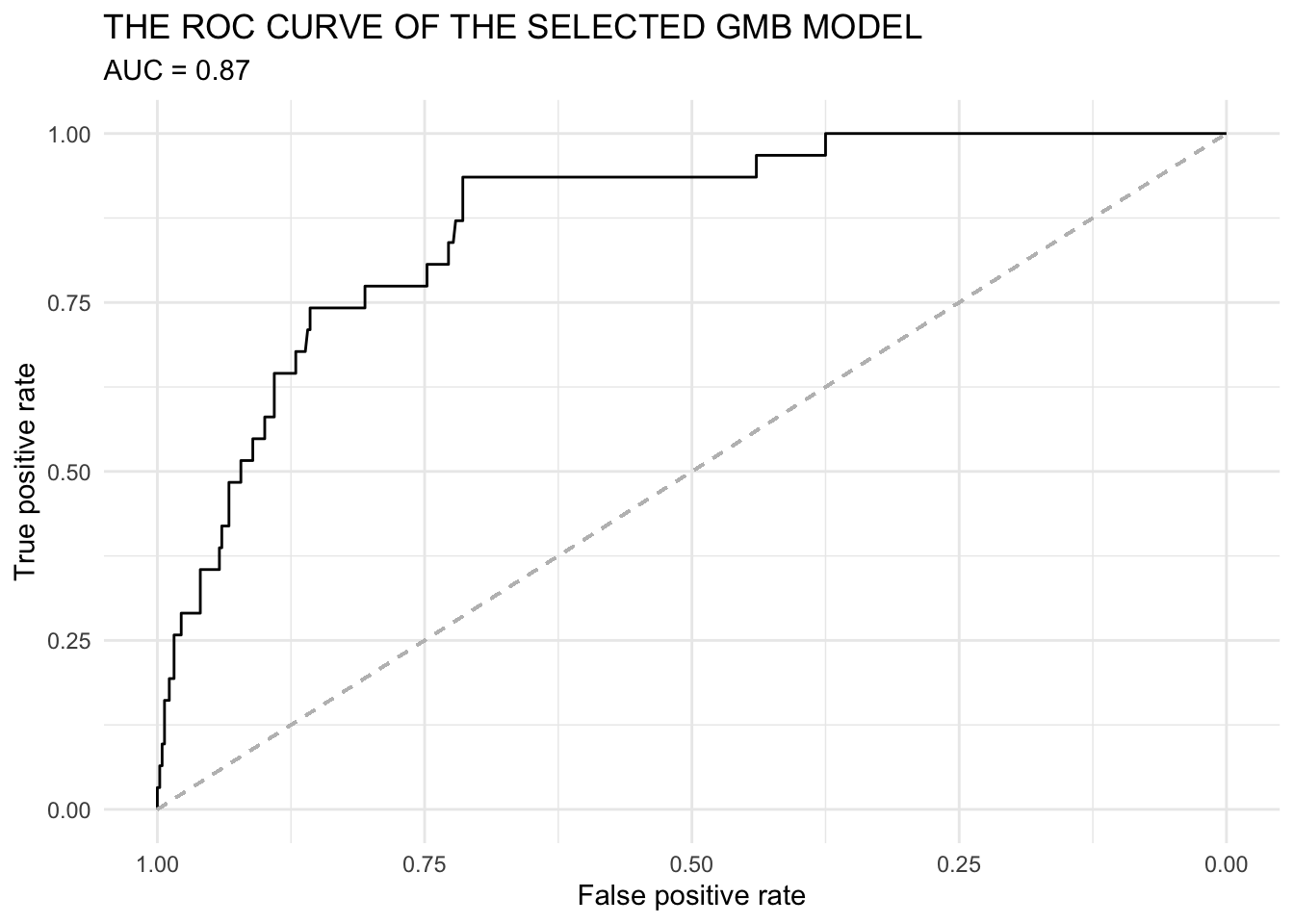


## Confusion Matrix and Statistics

##

## Reference

## Prediction No Yes

## No 320 2

## Yes 128 29

##

## Accuracy : 0.7286

## 95% CI : (0.6864, 0.768)

## No Information Rate : 0.9353

## P-Value [Acc > NIR] : 1

##

## Kappa : 0.2247

##

## Mcnemar's Test P-Value : <2e-16

##

## Sensitivity : 0.7143

## Specificity : 0.9355

## Pos Pred Value : 0.9938

## Neg Pred Value : 0.1847

## Prevalence : 0.9353

## Detection Rate : 0.6681

## Detection Prevalence : 0.6722

## Balanced Accuracy : 0.8249

##

## 'Positive' Class : No

##

## Reference

## Prediction No Yes

## No 320 2

## Yes 128 29


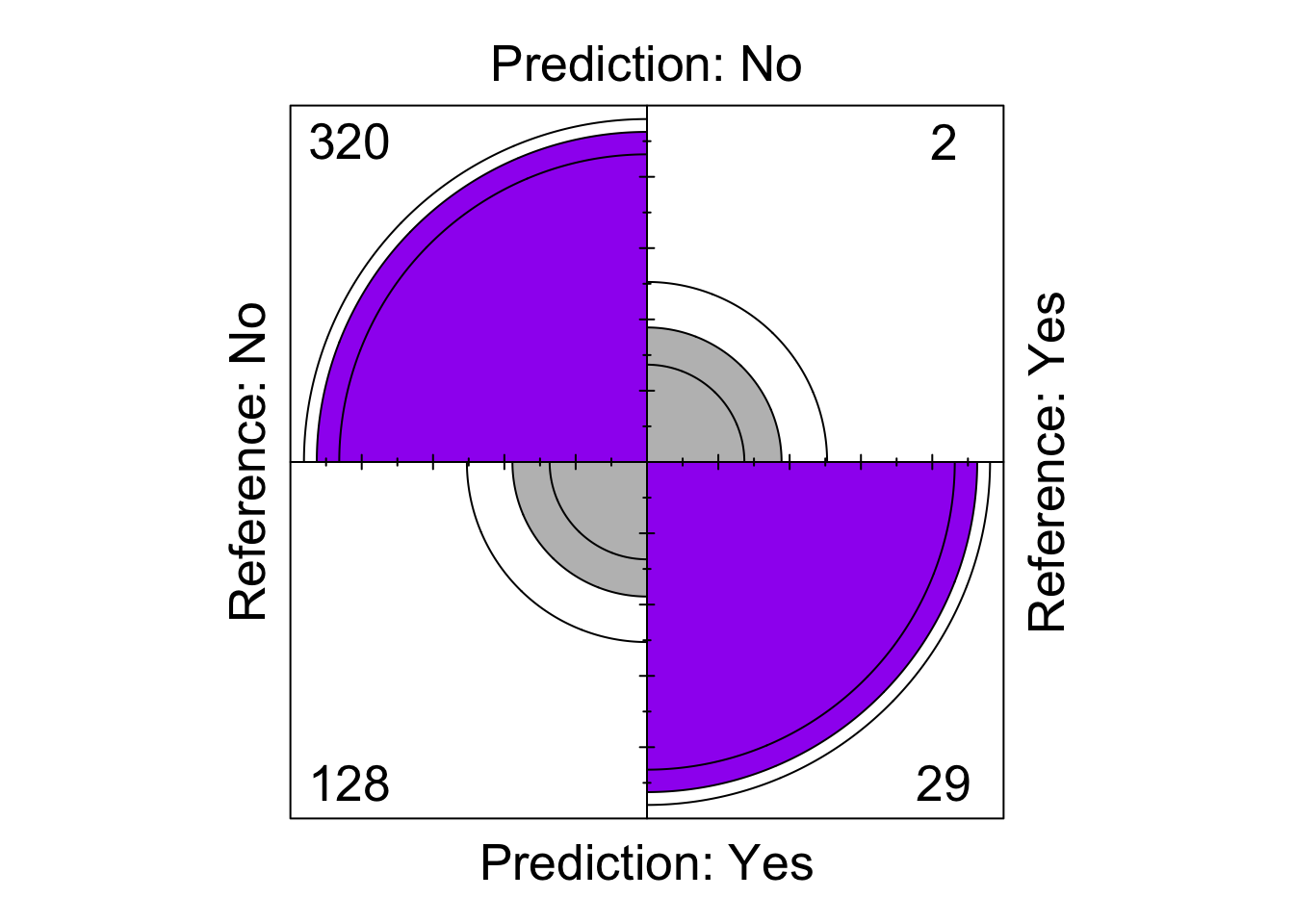


## Min. 1st Qu. Median Mean 3rd Qu. Max.

## 0.2259 0.2303 0.2871 0.3518 0.4232 0.9254


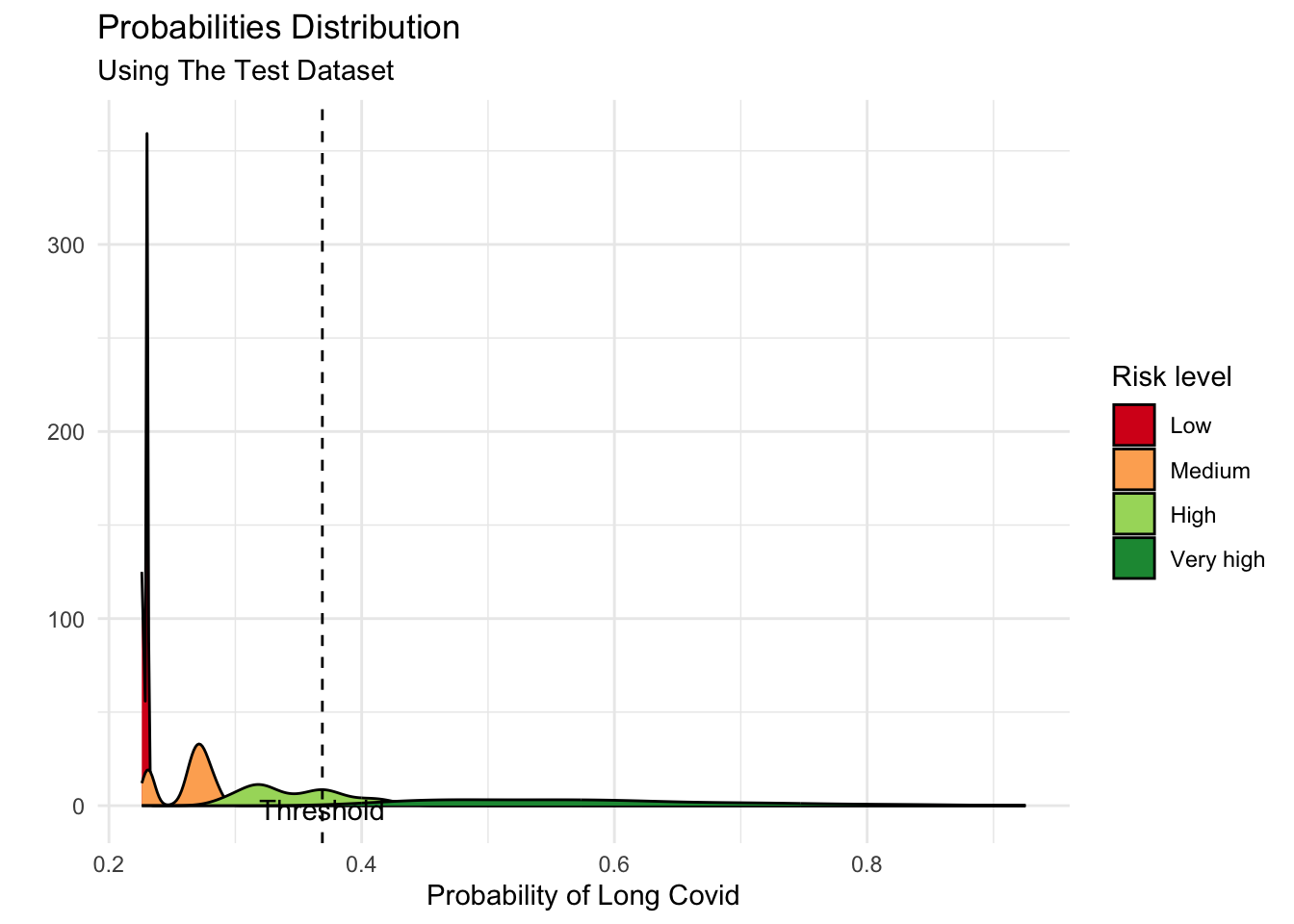


Metrics at different thresholds


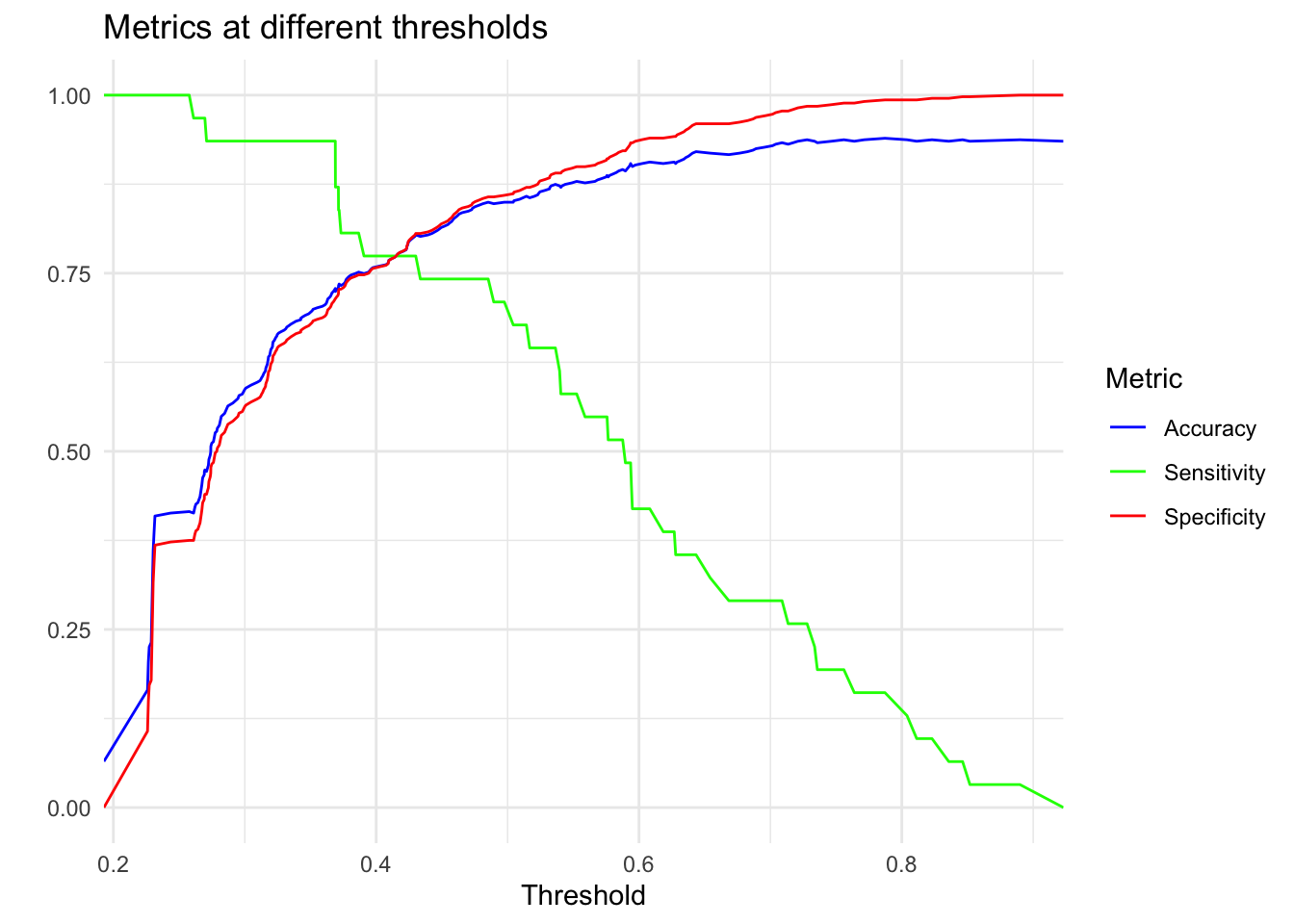


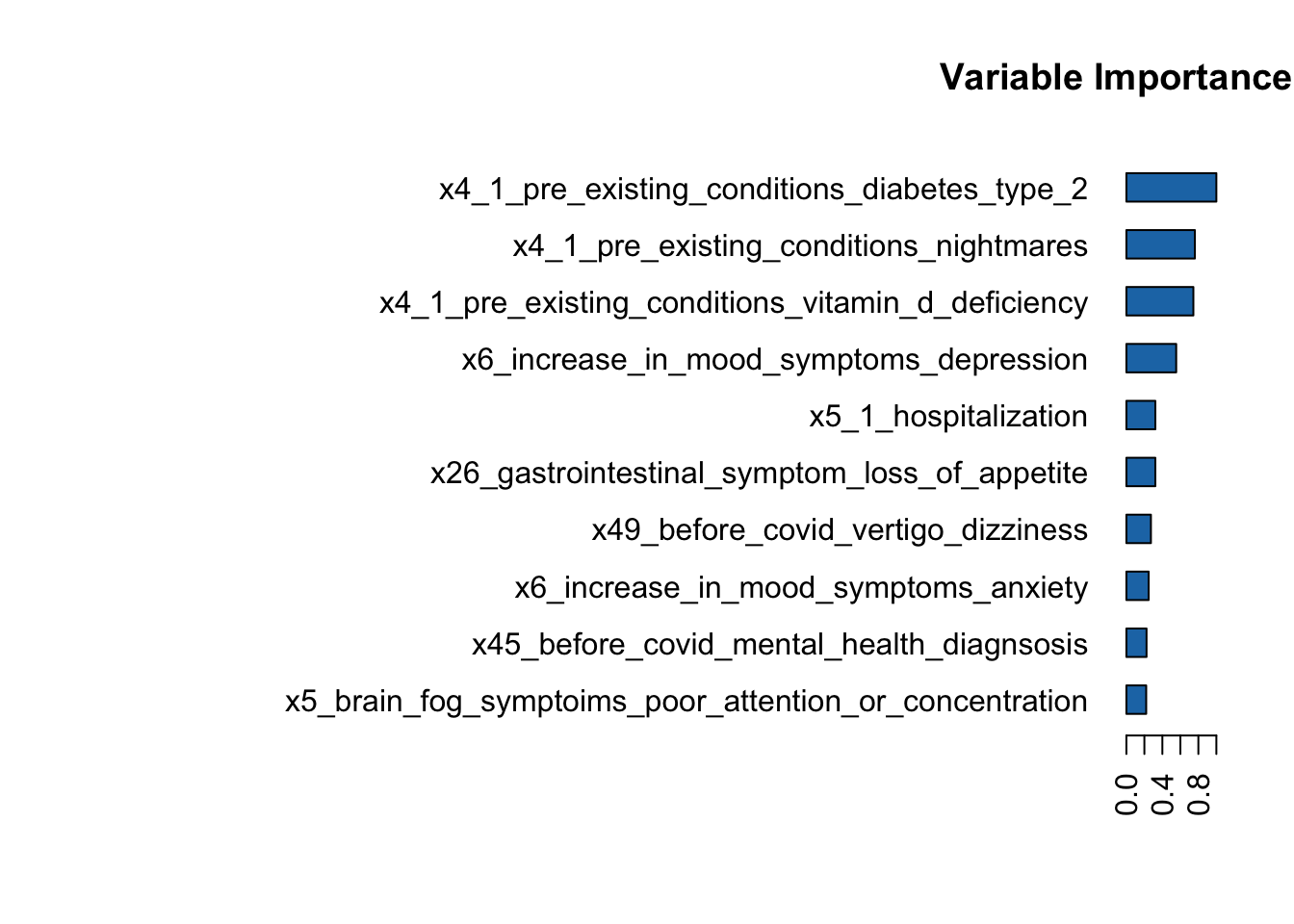


## variable

## 1 x4_1_pre_existing_conditions_diabetes_type_2

## 2 x4_1_pre_existing_conditions_nightmares

## 3 x4_1_pre_existing_conditions_vitamin_d_deficiency

## 4 x6_increase_in_mood_symptoms_depression

## 5 x5_1_hospitalization

## 6 x26_gastrointestinal_symptom_loss_of_appetite

## 7 x49_before_covid_vertigo_dizziness

## 8 x6_increase_in_mood_symptoms_anxiety

## 9 x45_before_covid_mental_health_diagnsosis

## 10 x5_brain_fog_symptoims_poor_attention_or_concentration

## 11 x23_cvs_symptoms_thumping_or_skipping_beats

## 12 x16_insomnia_description_difficulty_falling_asleep

## 13 x3_memory_loss_symptoms_or_forgetting_you_re_in_the_middle_of_a_task

## 14 infection_with_covid_19_after_vaccination

## 15 x4_7_smoking

## 16 x32_muscle_and_joint_symptoms_muscle_aches

## 17 x44_before_covid_health_status

## 18 type_vaccine

## 19 x4_1_pre_existing_conditions_vision_near_sighted_far_sighted

## 20 x4_1_pre_existing_conditions_insomnia

## 21 x4_1_pre_existing_conditions_mold

## 22 aspirin

## 23 paracetamol

## 24 naproxen

## 25 anti_oxidants

## 26 anti_type_one_histamine

## 27 omega_3

## 28 x6_increase_in_mood_symptoms_anger

## 29 x6_increase_in_mood_symptoms_irritability

## 30 x12_smell_and_taste_symptoms_altered_sense_of_smell

## 31 x28_respiratory_symptoms_dry_cough

## relative_importance scaled_importance percentage

## 1 1033.045654 1.000000000 0.186669707

## 2 786.466980 0.761309025 0.142113332

## 3 769.172241 0.744567520 0.138988201

## 4 571.953003 0.553657044 0.103350998

## 5 332.486298 0.321850536 0.060079745

## 6 332.359222 0.321727526 0.060056783

## 7 281.827301 0.272812048 0.050925745

## 8 255.679230 0.247500417 0.046200830

## 9 231.390427 0.223988578 0.041811882

## 10 227.503616 0.220226101 0.041109542

## 11 212.055664 0.205272306 0.038318121

## 12 185.068726 0.179148642 0.033441624

## 13 168.880005 0.163477775 0.030516348

## 14 132.206818 0.127977710 0.023889562

## 15 7.973788 0.007718718 0.001440851

## 16 6.014049 0.005821668 0.001086729

## 17 0.000000 0.000000000 0.000000000

## 18 0.000000 0.000000000 0.000000000

## 19 0.000000 0.000000000 0.000000000

## 20 0.000000 0.000000000 0.000000000

## 21 0.000000 0.000000000 0.000000000

## 22 0.000000 0.000000000 0.000000000

## 23 0.000000 0.000000000 0.000000000

## 24 0.000000 0.000000000 0.000000000

## 25 0.000000 0.000000000 0.000000000

## 26 0.000000 0.000000000 0.000000000

## 27 0.000000 0.000000000 0.000000000

## 28 0.000000 0.000000000 0.000000000

## 29 0.000000 0.000000000 0.000000000

## 30 0.000000 0.000000000 0.000000000

## 31 0.000000 0.000000000 0.000000000


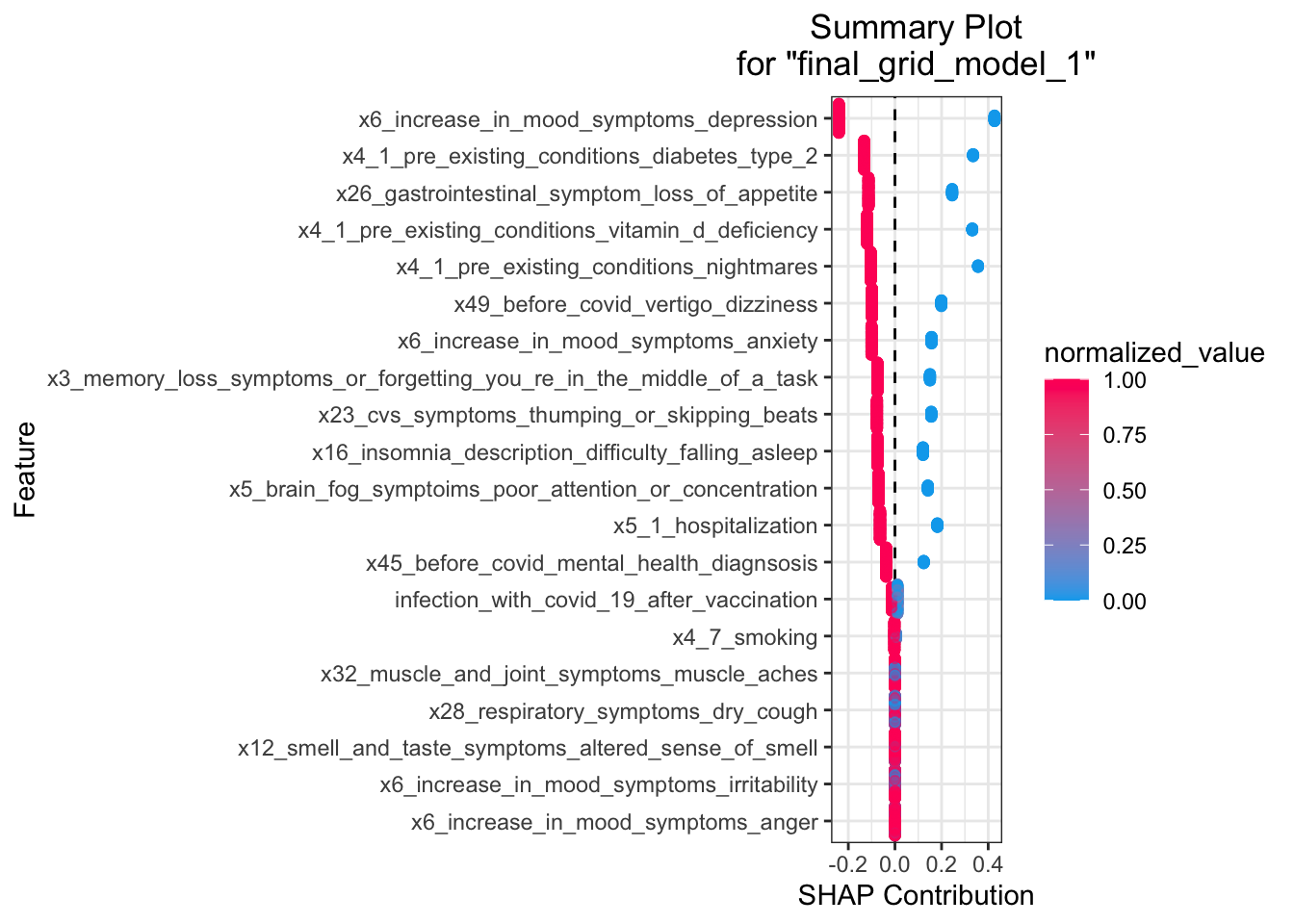


## Gains/Lift Table: Avg response rate: 51.69 %, avg score: 51.67 %

## group cumulative_data_fraction lower_threshold lift cumulative_lift

## 1 1 0.01042209 0.892410 1.934476 1.934476

## 2 2 0.02032308 0.872927 1.934476 1.934476

## 3 3 0.03022408 0.854087 1.832661 1.901123

## 4 4 0.04012507 0.846257 1.934476 1.909353

## 5 5 0.05002606 0.836144 1.832661 1.894174

## 6 6 0.10005211 0.795663 1.853873 1.874023

## 7 7 0.15007817 0.751794 1.833722 1.860590

## 8 8 0.20010422 0.718458 1.773269 1.838760

## 9 9 0.30015633 0.654951 1.692666 1.790062

## 10 10 0.40020844 0.580126 1.471008 1.710298

## 11 11 0.50026055 0.513696 1.188897 1.606018

## 12 12 0.59979156 0.433762 1.012815 1.507580

## 13 13 0.69984367 0.366473 0.594448 1.377036

## 14 14 0.80041688 0.309393 0.290673 1.240533

## 15 15 0.89994789 0.262842 0.050641 1.108935

## 16 16 1.00000000 0.225949 0.020151 1.000000

## response_rate score cumulative_response_rate cumulative_score

## 1 1.000000 0.909023 1.000000 0.909023

## 2 1.000000 0.882548 1.000000 0.896125

## 3 0.947368 0.863425 0.982759 0.885413

## 4 1.000000 0.850659 0.987013 0.876837

## 5 0.947368 0.841970 0.979167 0.869936

## 6 0.958333 0.813114 0.968750 0.841525

## 7 0.947917 0.773386 0.961806 0.818812

## 8 0.916667 0.732738 0.950521 0.797294

## 9 0.875000 0.686896 0.925347 0.760494

## 10 0.760417 0.615131 0.884115 0.724154

## 11 0.614583 0.545994 0.830208 0.688522

## 12 0.523560 0.472800 0.779322 0.652724

## 13 0.307292 0.398721 0.711839 0.616411

## 14 0.150259 0.336064 0.641276 0.581185

## 15 0.026178 0.281015 0.573248 0.547987

## 16 0.010417 0.235565 0.516936 0.516729

## capture_rate cumulative_capture_rate gain cumulative_gain

## 1 0.020161 0.020161 93.447581 93.447581

## 2 0.019153 0.039315 93.447581 93.447581

## 3 0.018145 0.057460 83.266129 90.112278

## 4 0.019153 0.076613 93.447581 90.935274

## 5 0.018145 0.094758 83.266129 89.417423

## 6 0.092742 0.187500 85.387265 87.402344

## 7 0.091734 0.279234 83.372186 86.058958

## 8 0.088710 0.367944 77.326949 83.875956

## 9 0.169355 0.537298 69.266633 79.006181

## 10 0.147177 0.684476 47.100764 71.029827

## 11 0.118952 0.803427 18.889659 60.601794

## 12 0.100806 0.904234 1.281456 50.758019

## 13 0.059476 0.963710 -40.555171 37.703564

## 14 0.029234 0.992944 -70.932747 24.053299

## 15 0.005040 0.997984 -94.935927 10.893518

## 16 0.002016 1.000000 -97.984921 0.000000

## kolmogorov_smirnov

## 1 0.020161

## 2 0.039315

## 3 0.056381

## 4 0.075534

## 5 0.092601

## 6 0.181028

## 7 0.267368

## 8 0.347447

## 9 0.490912

## 10 0.588467

## 11 0.627591

## 12 0.630232

## 13 0.546234

## 14 0.398553

## 15 0.202946

## 16 0.000000

##

##

## Confusion Matrix

## ================

##

## > Confusion matrix shows a predicted class vs an actual class.

##

##

##

## final_grid_model_1

## ------------------

##

## | | No | Yes | Error | Rate

## |:---:|:---:|:---:|:---:|:---:|

## | **No** |399 | 49 | 0.109375 | =49/448 |

## | **Yes** |11 | 20 | 0.354838709677419 | =11/31 |

## | **Totals** |410 | 69 | 0.125260960334029 | =60/479 |

##

##

## Learning Curve Plot

## ===================

##

## > Learning curve plot shows the loss function/metric dependent on number of iterations or trees for tree-based algorithms. This plot can be useful for determining whether the model overfits.


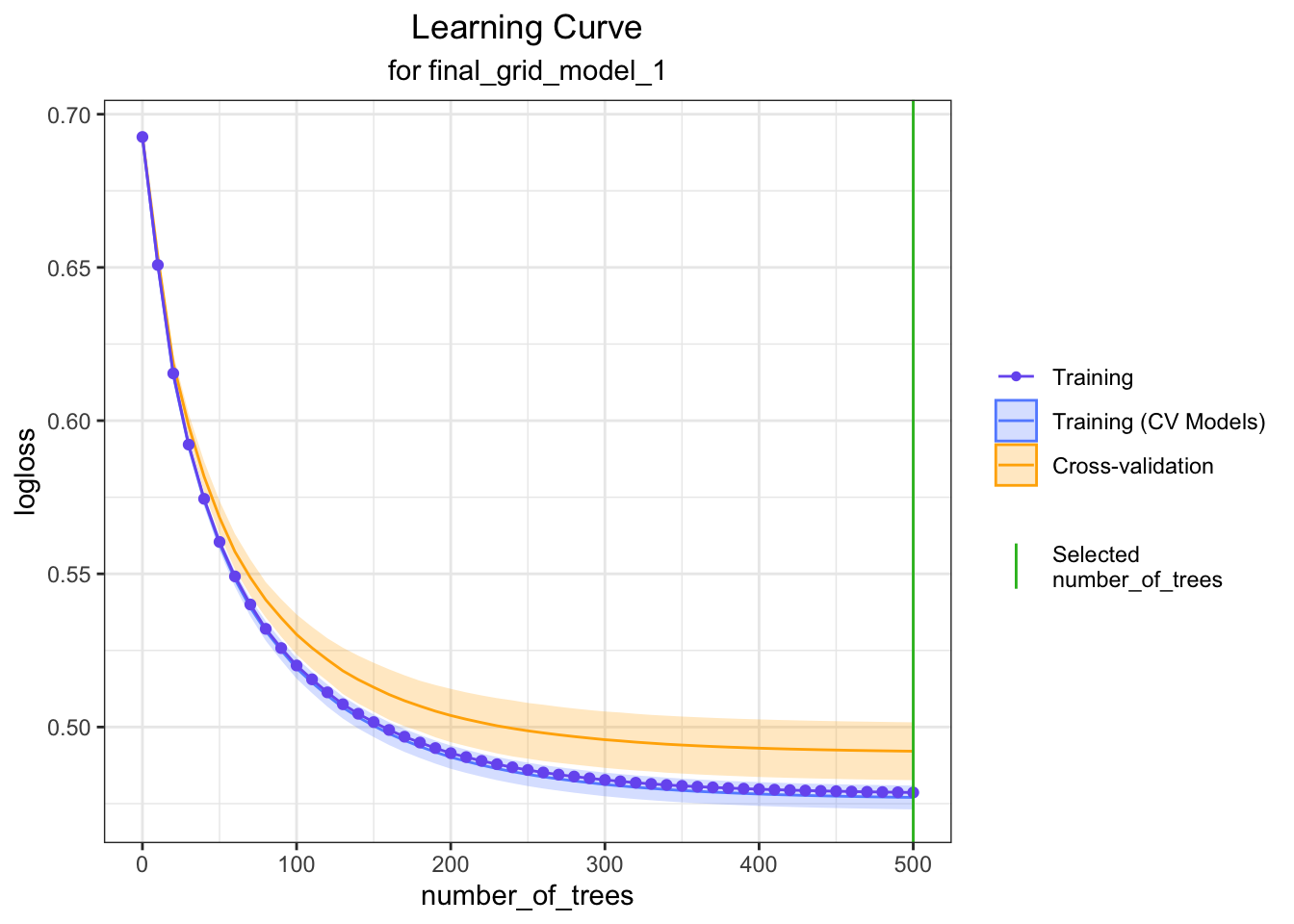


##

##

## Variable Importance

## ===================

##

## > The variable importance plot shows the relative importance of the most important variables in the model.


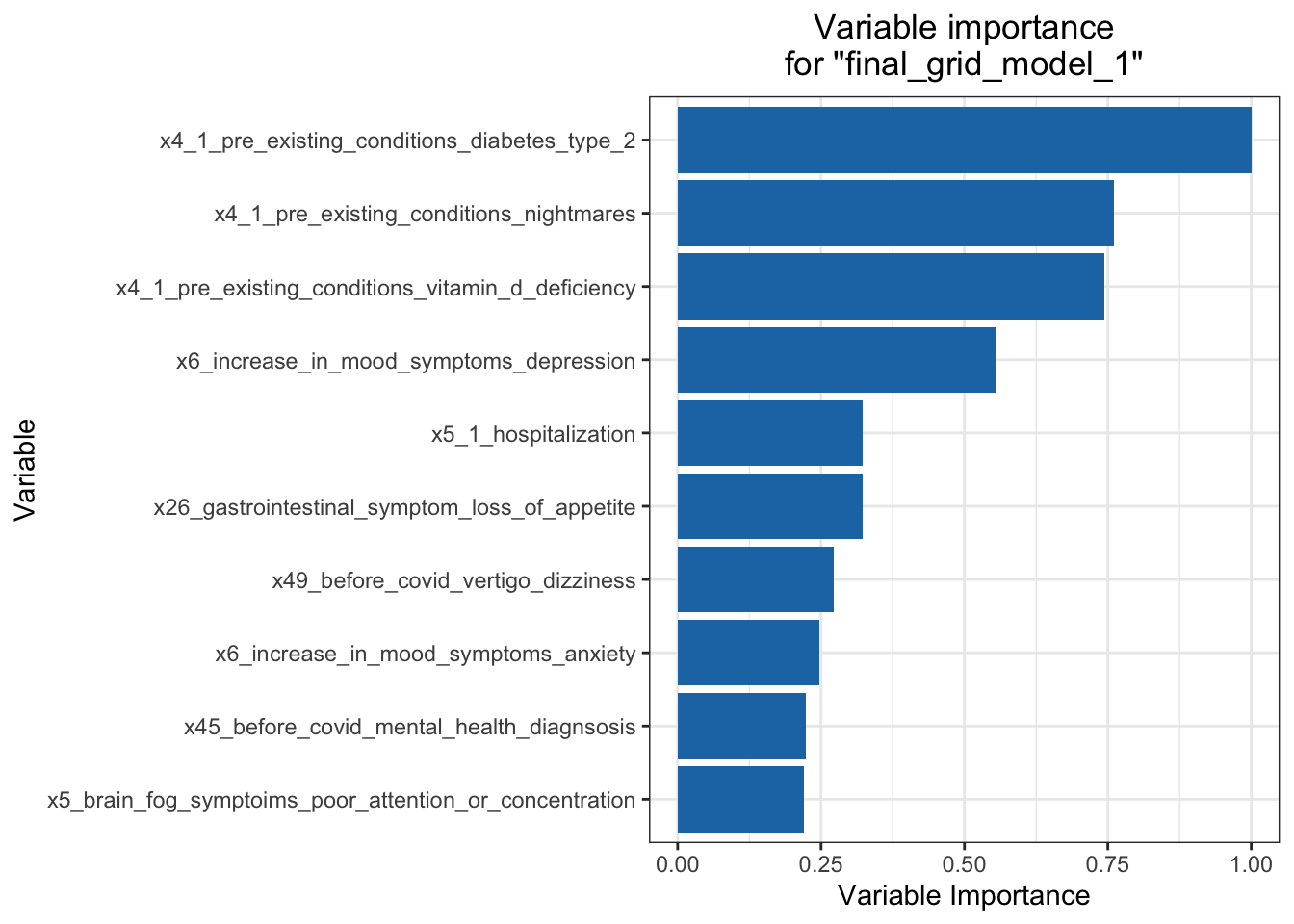


##

##

## SHAP Summary

## ============

##

## > SHAP summary plot shows the contribution of the features for each instance (row of data). The sum of the feature contributions and the bias term is equal to the raw prediction of the model, i.e., prediction before applying inverse link function.


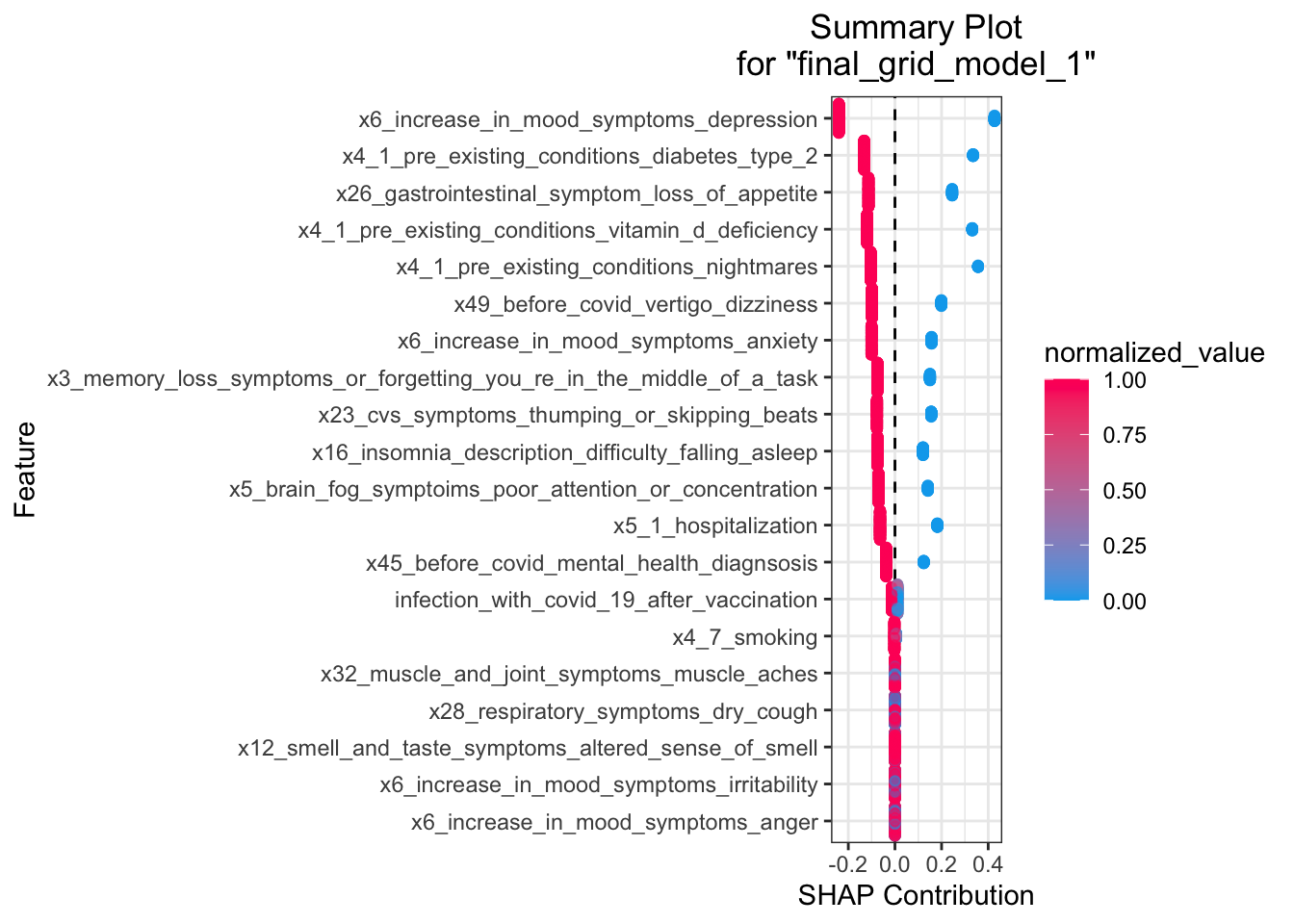


##

##

## Partial Dependence Plots

## ========================

##

## > Partial dependence plot (PDP) gives a graphical depiction of the marginal effect of a variable on the response. The effect of a variable is measured in change in the mean response. PDP assumes independence between the feature for which is the PDP computed and the rest.


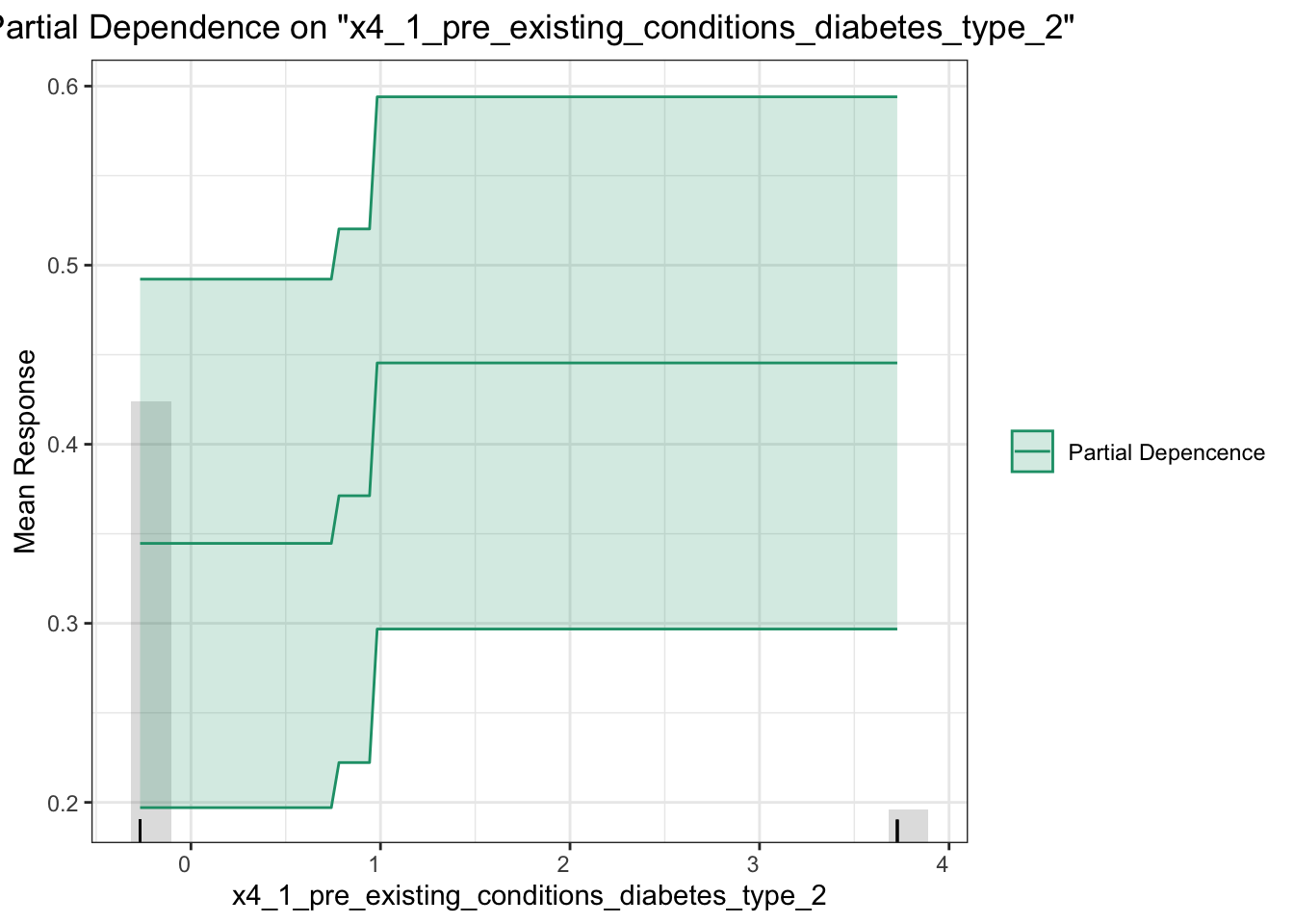

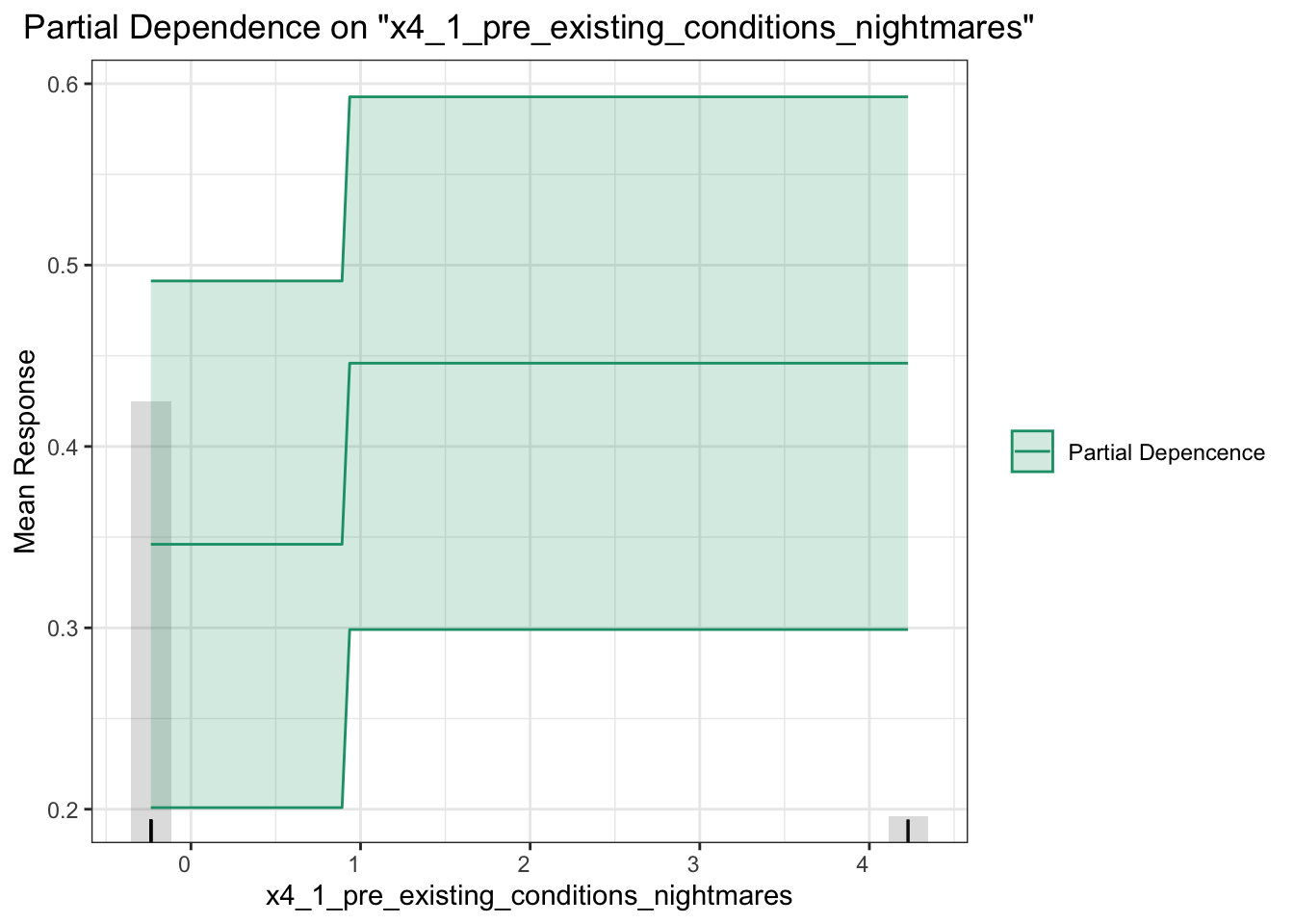

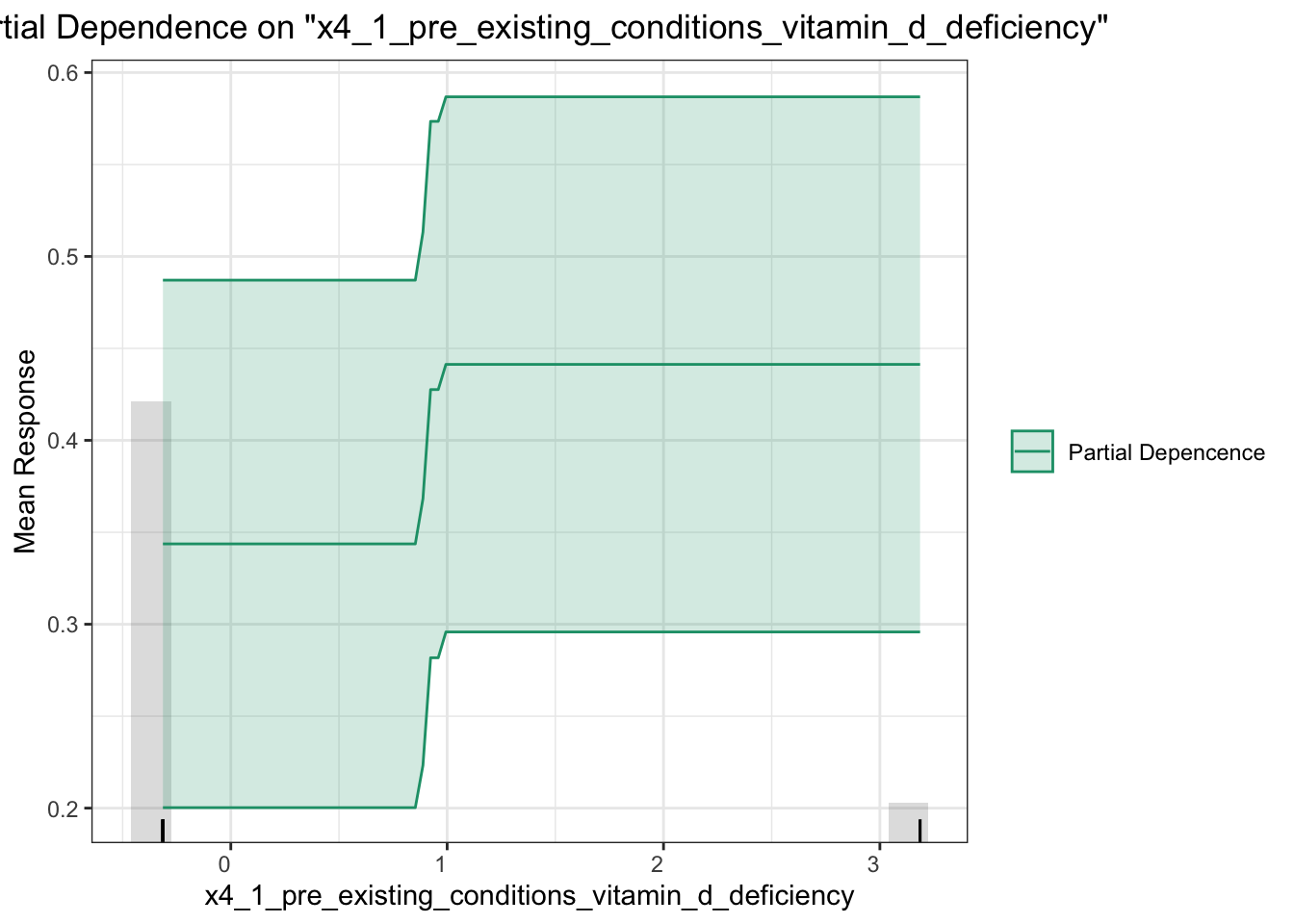

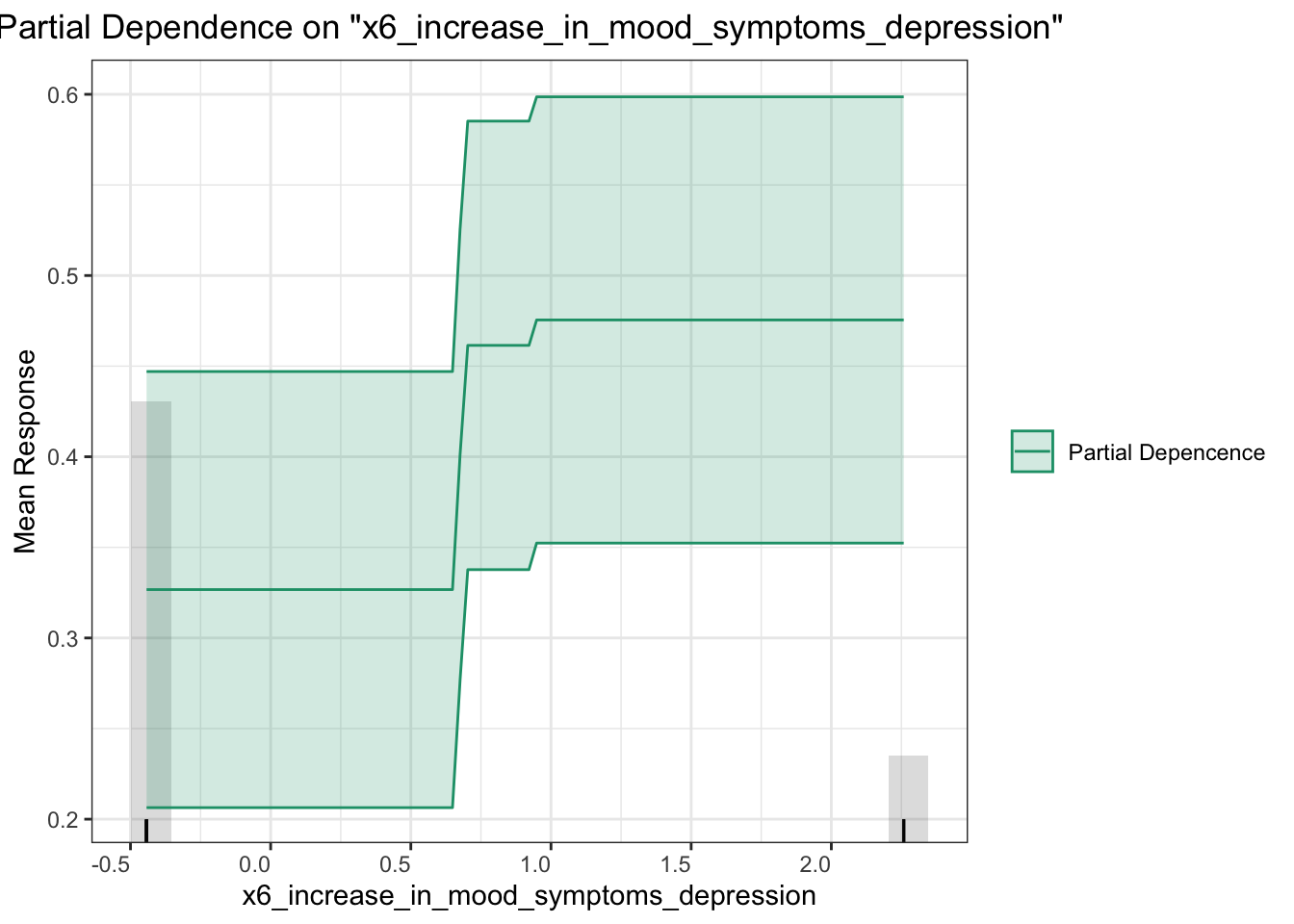

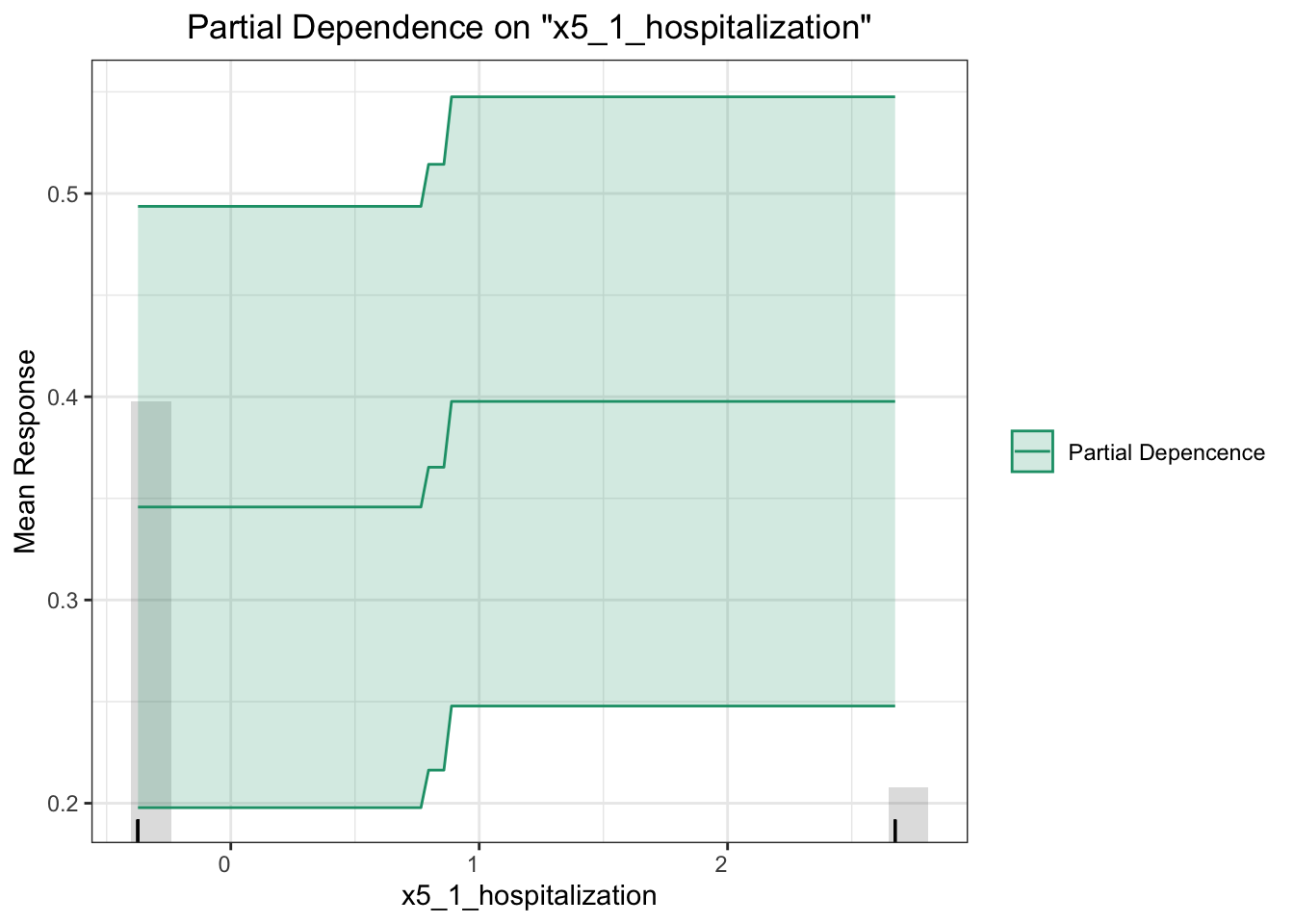


# Machine learning: depression

Load libraries

Get the data for analysis

# dfAnalysis <- read.csv("/Volumes/Ahmed Shaheen/Old Files/long-covid-shaheen/model_data.csv")[,-1]

#

# # Convert them to factors using lapply

# dfAnalysis[names(dfAnalysis %>% select(!c("BMI")))] <- lapply(dfAnalysis[names(dfAnalysis %>% select(!c("BMI")))], as.factor)

#

#

# levels(dfAnalysis$age_group) <- c("18-29", "30-39", "40-49", "50-59","> 60")

# levels(dfAnalysis$infection_with_covid_19_after_vaccination) <- c("Not vaccinated", "No","Yes")

#

# levels(dfAnalysis$household_income) <- c("More than $10,000","Don’t know/Not sure", "Less than $10,000")

Get the outcomes data

# outcomes <- read.csv("/Volumes/Ahmed Shaheen/Old Files/long-covid-shaheen/df1_outcomes.csv")[-1] %>%

# select(!c("x52_dizziness_or_vertigo_symptoms","x56_tinnitus_symptoms",

# "x32_muscle_and_joint_symptoms_muscle_and_joint_issues",

# "x10_headaches_symptoms_diffuse_entire_brain","x26_gastrointestinal_symptom_abdominal_pain",

# "x3_memory_loss_symptoms_i_e_remembering_a_phone_number_before_writing_it_down",

# "x22_temperature_symptoms_other_temperature_issues_not_listed_above_or_below",

# "x24_generic_symptoms_dizziness_vertigo_unsteadiness_or_balance_issues"))

#

# outcomes$depression <- factor(outcomes$depression,

# levels=c("Mild", "Moderate", "Moderately severe", "None", "Severe"),

# labels = c("Mild", "Moderate", "ModeratelySevere", "None", "Severe"))

#

#

# outcomes$depression <- as.character(outcomes$depression)

#

# outcomes$depressionBin[outcomes$depression %in% c("Mild", "Moderate", "ModeratelySevere", "Severe")] <- "Yes"

#

# outcomes$depressionBin[outcomes$depression %in% c("None")] <- "No"

#

# outcomes$depression <- as.factor(outcomes$depression)

#

# outcomes$depressionBin <- as.factor(outcomes$depressionBin)

#

# # Convert them to factors using lapply

# outcomes[names(outcomes %>% select(!c("x53_vertigo_dizziness_severity")))] <- lapply(outcomes[names(outcomes %>% select(!c("x53_vertigo_dizziness_severity")))], factor)

#

# Embeddings <- read.csv("/Volumes/Ahmed Shaheen/Old Files/long-covid-shaheen/dataWithembddings.csv")

# Data <- read.csv("/Volumes/Ahmed Shaheen/Old Files/long-covid-shaheen/dfAnalysis.csv")

#

# longCovid <- Data$longCovid

# encoder_output <- Data$layer_output

# embeddings <- Embeddings$X0

# symptoms_duration <- Data$symptoms_duration

# set.seed(123)

# dat <- dfAnalysis %>% select(!c("covid_19_vaccination",

# "household_income","vaccine_shots_n"))

#

# dat <- cbind(dat,outcomes[,1:46]) %>%

# mutate_if(is.factor, ~as.numeric(as.factor(.)))

#

#

# my_preprocess <- preProcess(dat, method = c("center", "scale", "YeoJohnson"))

# dat <- predict(my_preprocess, dat)

#

# #Remove highly correlated variables

# cor_matrix <- cor(dat)

# cor_matrix_rm <- cor_matrix

# cor_matrix_rm[upper.tri(cor_matrix_rm)] <- 0

# diag(cor_matrix_rm) <- 0

#

# dat <- dat[ , !apply(cor_matrix_rm, 2, function(x) any(x > 0.99))]

# heatmap(cor(dat))

#

#

# dat <- dat %>% select(!names(dat)[nearZeroVar(dat)])

#

# dat$depression <- outcomes$depressionBin

#

# dat <- dat %>% na.omit()

#

# trainIndex <- caret::createDataPartition(dat$depression, p = .8,

# list = FALSE,

# times = 1)

#

# datTrain <- dat[ trainIndex,]

# datTest <- dat[-trainIndex,]

# full.model <- glm(depression ~ ., data = datTrain,binomial())

# step.model <- MASS::stepAIC(full.model, direction = "backward", trace = FALSE)

# vects <- ls()

# spare <- c("dat","step.model")

# rem <- setdiff(vects, spare)

# rm(rem)

# dput(names(step.model$model))

data <- read.csv("/Volumes/Ahmed Shaheen/Old Files/long-covid-shaheen/app/app_data.csv")

depressionVars <- c("sex", "healthcare_professional", "x44_before_covid_health_status",

"x45_before_covid_mental_health_diagnsosis", "x49_before_covid_vertigo_dizziness",

"x5_3_oxygen_support", "infection_with_covid_19_after_vaccination",

"x4_1_pre_existing_conditions_vision_near_sighted_far_sighted",

"x4_1_pre_existing_conditions_diabetes_type_2", "x4_1_pre_existing_conditions_irritable_bowel_syndrome_ibs",

"x4_1_pre_existing_conditions_nightmares", "x4_1_pre_existing_conditions_food_allergies",

"aspirin", "paracetamol", "naproxen", "anti_oxidants", "anti_type_two_histamine",

"omega_3", "x57_migraine", "x55_tinnitus_experience", "x3_memory_loss_symptoms_or_forgetting_you_re_in_the_middle_of_a_task",

"x5_brain_fog_symptoims_poor_attention_or_concentration", "x5_brain_fog_symptoims_abstracting",

"x6_increase_in_mood_symptoms_depression", "x6_increase_in_mood_symptoms_anger",

"x6_increase_in_mood_symptoms_difficulty_controlling_your_emotions",

"x10_headaches_symptoms_headaches", "x15_sleeping_symptoms_insomnia",

"x16_insomnia_description_waking_up_several_times_during_the_night",

"x23_cvs_symptoms_thumping_or_skipping_beats", "x23_cvs_symptoms_tachycardia",

"x28_respiratory_symptoms_cough_with_mucus_production", "x28_respiratory_symptoms_tightness_of_chest",

"x32_muscle_and_joint_symptoms_joint_pain", "x32_muscle_and_joint_symptoms_muscle_aches",

"x41_gastrointestinal_symptoms_abdominal_pain")

data <- data[,depressionVars] %>% mutate_if(is.character, ~as.factor(as.character(.))) %>% mutate_if(is.factor, ~as.numeric(as.factor(.)))

data$depression <- as.factor(read.csv("/Volumes/Ahmed Shaheen/Old Files/long-covid-shaheen/app/app_data.csv")$depression)

my_preprocess <- preProcess(data, method = c("center", "scale", "YeoJohnson"))

data <- predict(my_preprocess, data)

trainIndex <- caret::createDataPartition(data$depression, p = .8, list = FALSE, times = 1)

Select variables

AutoML

## | | | 0% | |== | 3%

## 15:24:57.57: AutoML: XGBoost is not available; skipping it. | |====== | 9% | |============ | 18% | |================================= | 47% | |======================================================================| 100%

## AutoML Details

## ==============

## Project Name: AutoML_5_20240826_152457

## Leader Model ID: StackedEnsemble_AllModels_1_AutoML_5_20240826_152457

## Algorithm: stackedensemble

##

## Total Number of Models Trained: 12

## Start Time: 2024-08-26 16:24:57 UTC

## End Time: 2024-08-26 16:25:05 UTC

## Duration: 8 s

##

## Leaderboard

## ===========

## model_id auc logloss

## 1 StackedEnsemble_AllModels_1_AutoML_5_20240826_152457 0.8106166 0.5279594

## 2 StackedEnsemble_BestOfFamily_1_AutoML_5_20240826_152457 0.8103571 0.5281153

## 3 GBM_grid_1_AutoML_5_20240826_152457_model_1 0.8080619 0.5320530

## 4 GBM_1_AutoML_5_20240826_152457 0.8050453 0.5354235

## 5 GBM_4_AutoML_5_20240826_152457 0.8028393 0.5376546

## 6 GBM_2_AutoML_5_20240826_152457 0.8024633 0.5378463

## 7 GLM_1_AutoML_5_20240826_152457 0.8024557 0.5374769

## 8 GBM_5_AutoML_5_20240826_152457 0.8005565 0.5420368

## 9 GBM_3_AutoML_5_20240826_152457 0.7999407 0.5406196

## 10 XRT_1_AutoML_5_20240826_152457 0.7957316 0.5483892

## aucpr mean_per_class_error rmse mse

## 1 0.8063497 0.2631354 0.4190906 0.1756369

## 2 0.8072067 0.2856400 0.4193225 0.1758313

## 3 0.8017435 0.2923462 0.4210896 0.1773164

## 4 0.8052652 0.2578753 0.4226218 0.1786092

## 5 0.7967214 0.2548168 0.4232649 0.1791532

## 6 0.7960404 0.2646674 0.4234309 0.1792937

## 7 0.8015138 0.2760472 0.4238838 0.1796775

## 8 0.7927281 0.2725312 0.4254363 0.1809961

## 9 0.7928672 0.2704862 0.4250325 0.1806526

## 10 0.7899250 0.2774503 0.4284140 0.1835385

##

## [12 rows x 7 columns]

## [1] "Best Best-AML Test AUC: 0.784546883184914"


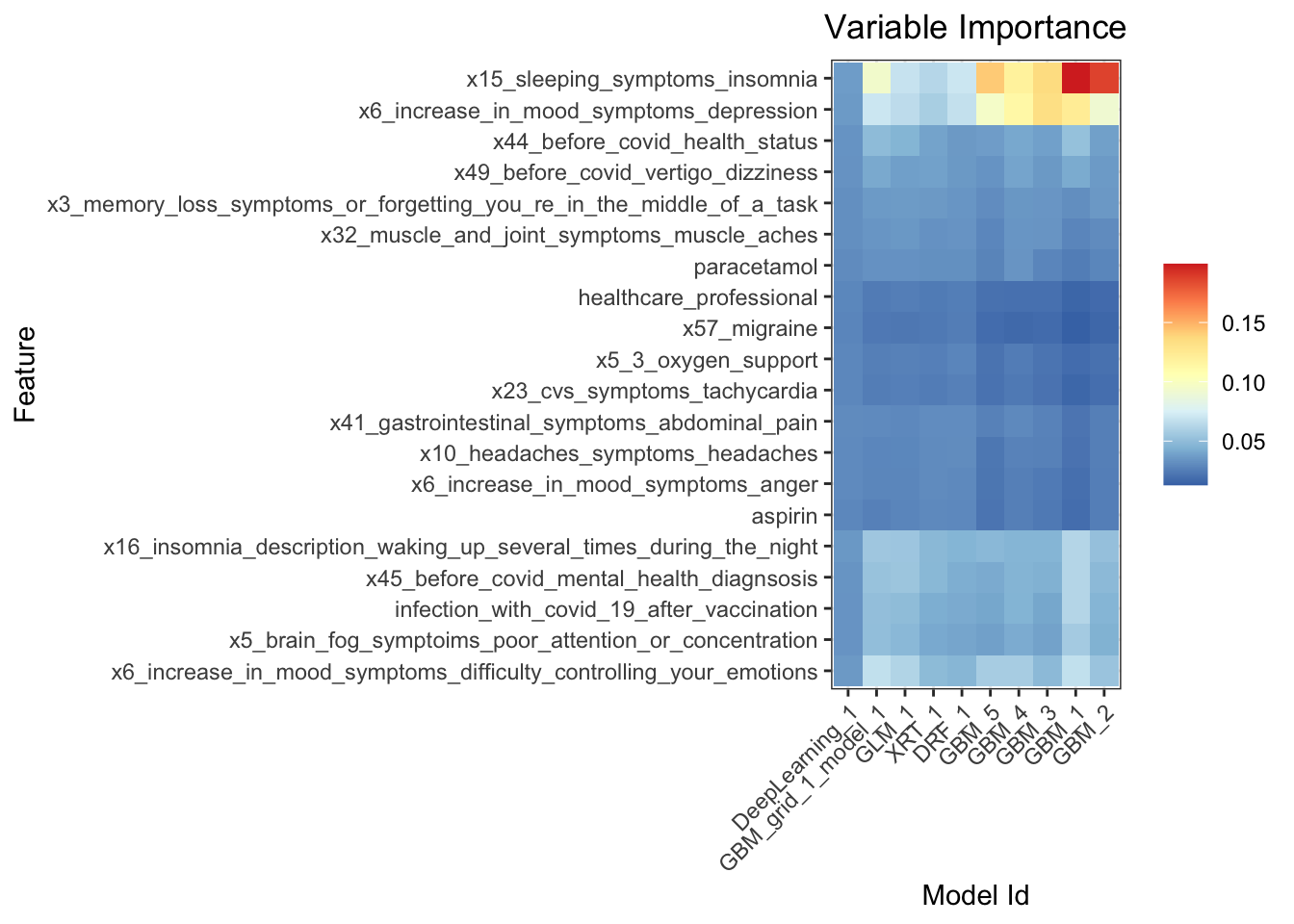

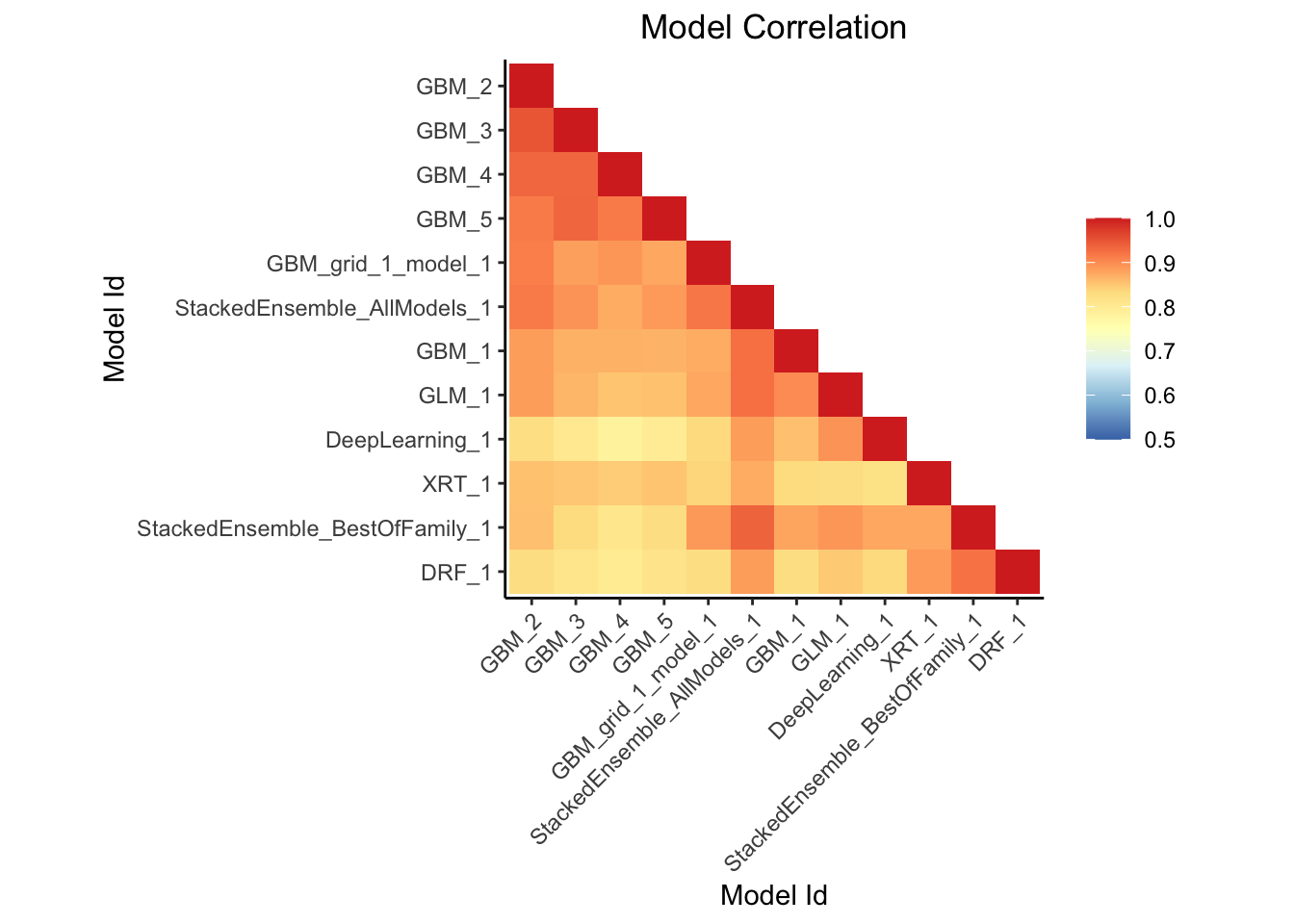


GMB

## | | | 0% | | | 1% | |= | 1% | |= | 2% | |== | 2% | |== | 3% | |== | 4% | |=== | 4% | |=== | 5% | |==== | 5% | |==== | 6% | |===== | 6% | |===== | 7% | |===== | 8% | |====== | 8% | |====== | 9% | |======= | 9% | |======= | 10% | |======= | 11% | |======== | 11% | |======== | 12% | |======================================================================| 100%

## | | | 0% | |======================================================================| 100%

## [1] "Best Base-learner Test AUC: 0.791400384145277"

## [1] "Ensemble Test AUC: 0.779352191374192"

## [1] 0.7914004

#ROC curve for all the models


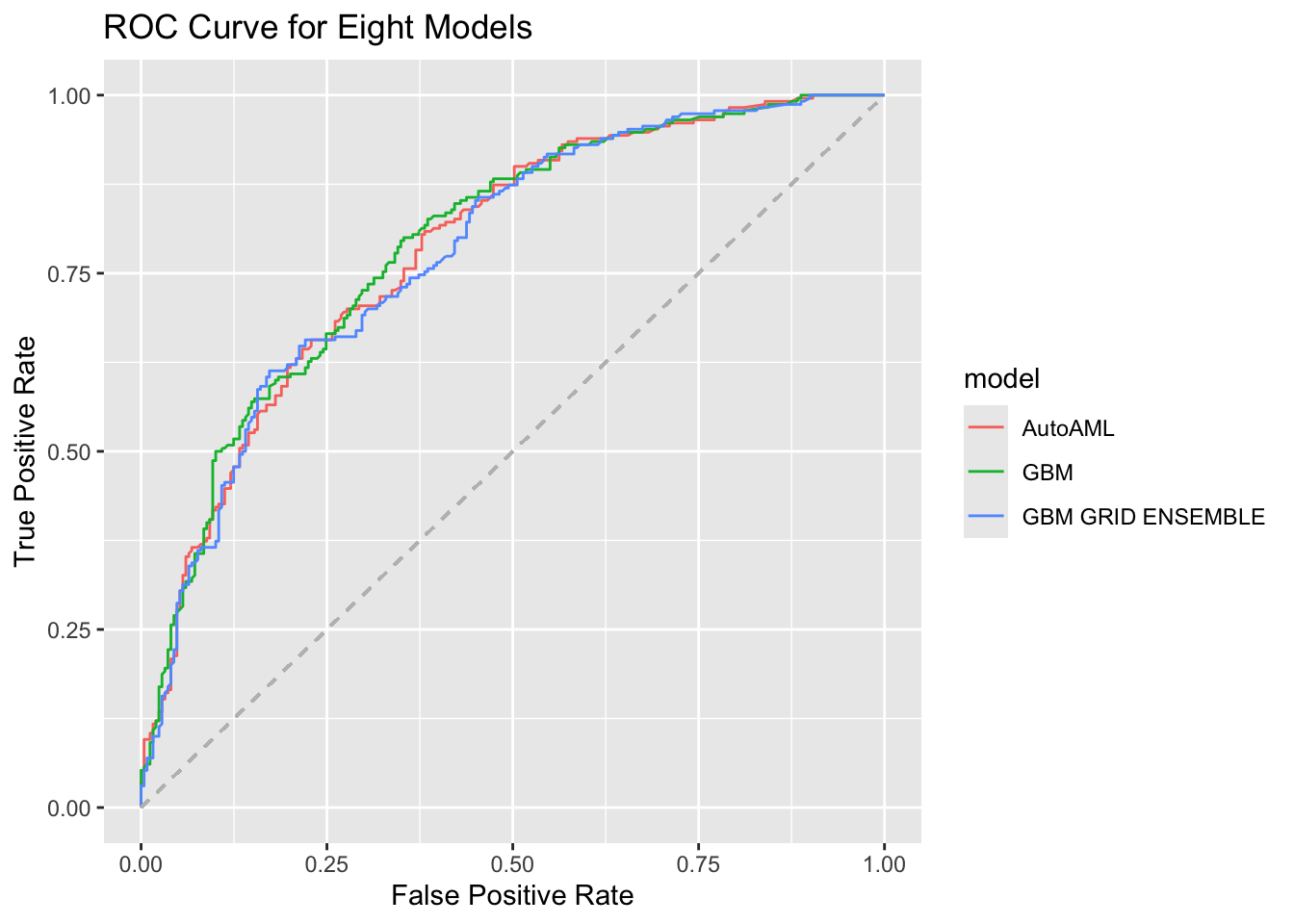


Table for model comparison

## models_names Index

## 1 GBM 3

## # A tibble: 3 × 22

## threshold f1 f2 f0point5 accuracy precision recall specificity

## <dbl> <dbl> <dbl> <dbl> <dbl> <dbl> <dbl> <dbl>

## 1 0.291 0.737 0.827 0.664 0.691 0.623 0.9 0.498

## 2 0.239 0.731 0.833 0.652 0.676 0.608 0.917 0.454

## 3 0.290 0.737 0.818 0.670 0.697 0.632 0.883 0.526

## # ℹ 14 more variables: absolute_mcc <dbl>, min_per_class_accuracy <dbl>,

## # mean_per_class_accuracy <dbl>, tns <dbl>, fns <dbl>, fps <dbl>, tps <dbl>,

## # tnr <dbl>, fnr <dbl>, fpr <dbl>, tpr <dbl>, idx <int>, model_name <chr>,

## # AUC <dbl>

Save the best performing model

## [1] "/Volumes/Ahmed Shaheen/Old Files/long-covid-shaheen/Models/depression/final_grid_model_11"

Load the model

loaded_model <- h2o.loadModel(model_path)

Make predictions

## | | | 0% | |======================================================================| 100%

Brier Score

## [1] 0.1895655

Calibration

# Convert h2o frames to data frames

test_df <- as.data.frame(test)

prediction_df <- as.data.frame(prediction)

# Prepare data for calibration plot

calibration_data <- data.frame(

actual = test_df$depression,

predicted = prediction_df$Yes

)

# Create bins

num_bins <- 10

calibration_data$bin <- cut(calibration_data$predicted,

breaks = seq(0, 1, length.out = num_bins + 1),

include.lowest = TRUE)

# Calculate mean predicted and actual values for each bin

calibration_summary <- calibration_data %>%

group_by(bin) %>%

summarize(

mean_predicted = mean(predicted),

mean_actual = mean(actual == "Yes"),

n = n()

)

# Create the calibration plot

ggplot(calibration_summary, aes(x = mean_predicted, y = mean_actual)) +

geom_point(aes(size = n), alpha = 0.7) +

geom_abline(intercept = 0, slope = 1, linetype = "dashed", color = "red") +

geom_smooth(method = "loess", se = FALSE, color = "blue") +

xlim(0, 1) + ylim(0, 1) +

labs(

x = "Mean Predicted Probability",

y = "Observed Fraction of Positives",

title = "Calibration Plot",

subtitle = "Perfect calibration represented by dashed red line"

) +

theme_minimal() +

theme(legend.position = "bottom")

## `geom_smooth()` using formula = 'y ~ x'

## Warning: Removed 1 row containing missing values or values outside the scale range

## (`geom_smooth()`).


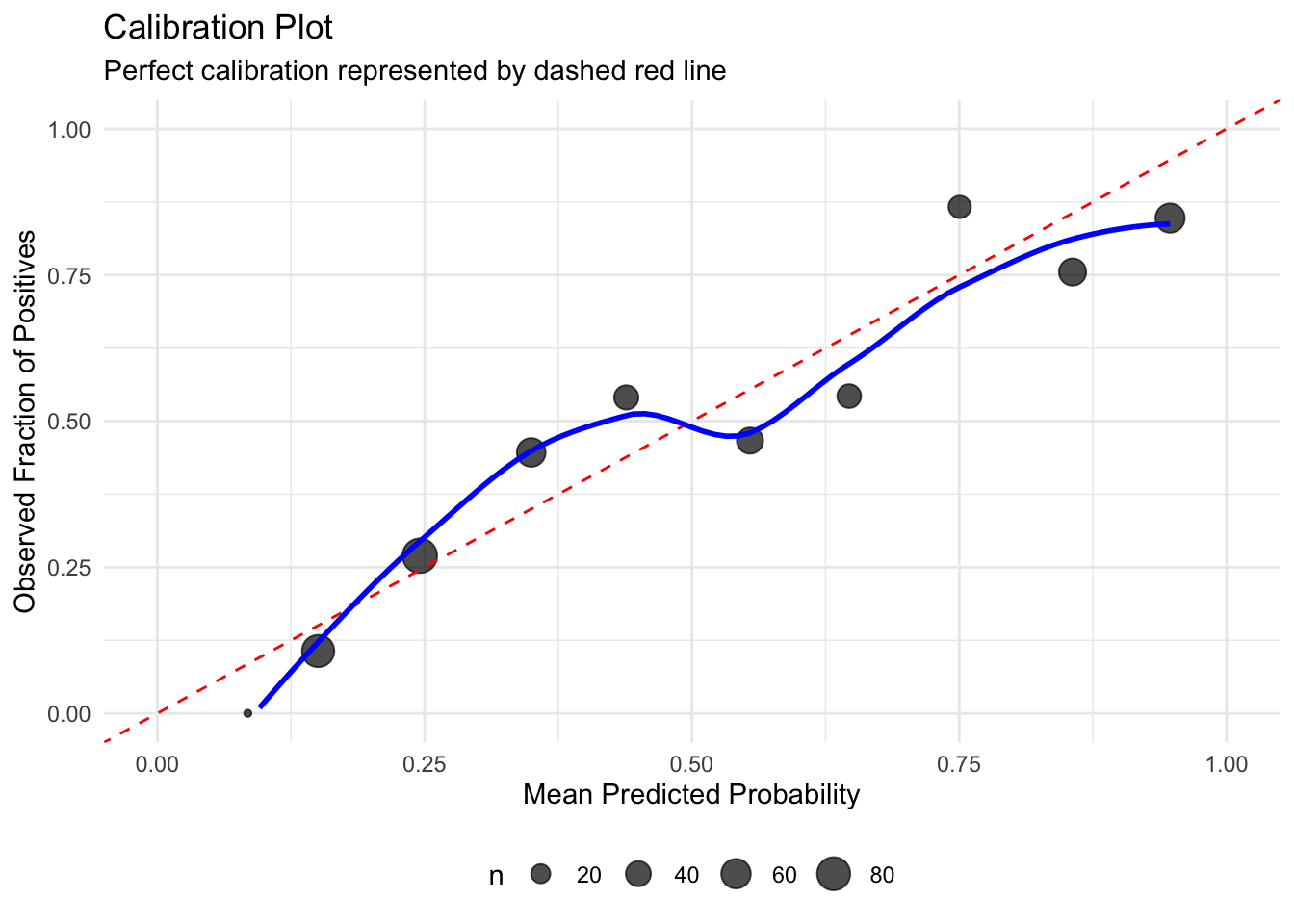


# Print the plot

print(last_plot())

## `geom_smooth()` using formula = 'y ~ x'

## Warning: Removed 1 row containing missing values or values outside the scale range

## (`geom_smooth()`).


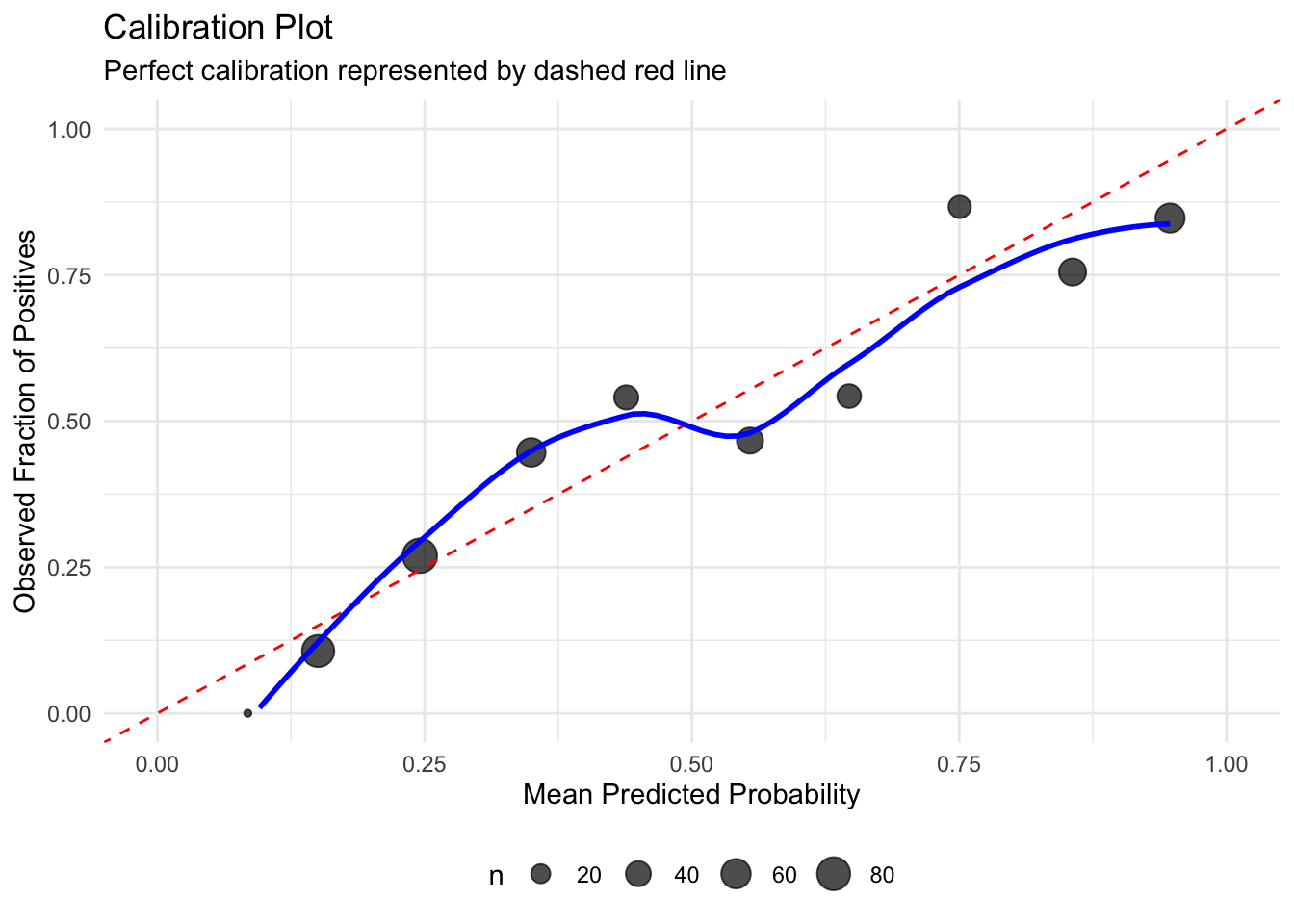


# Calculate and print Brier Score

brier_score <- DescTools::BrierScore(as.numeric(test_df$depression == "Yes"),

pred = prediction_df$Yes)

cat("Brier Score:", brier_score, "\n")

## Brier Score: 0.1895655

## Min. 1st Qu. Median Mean 3rd Qu. Max.

## 0.07037 0.23561 0.42578 0.49524 0.76230 0.98862

## [1] 479

## [1] 479

## threshold

## 1 0.3656033

## Min. 1st Qu. Median Mean 3rd Qu. Max.

## 0.07037 0.23561 0.42578 0.49524 0.76230 0.98862


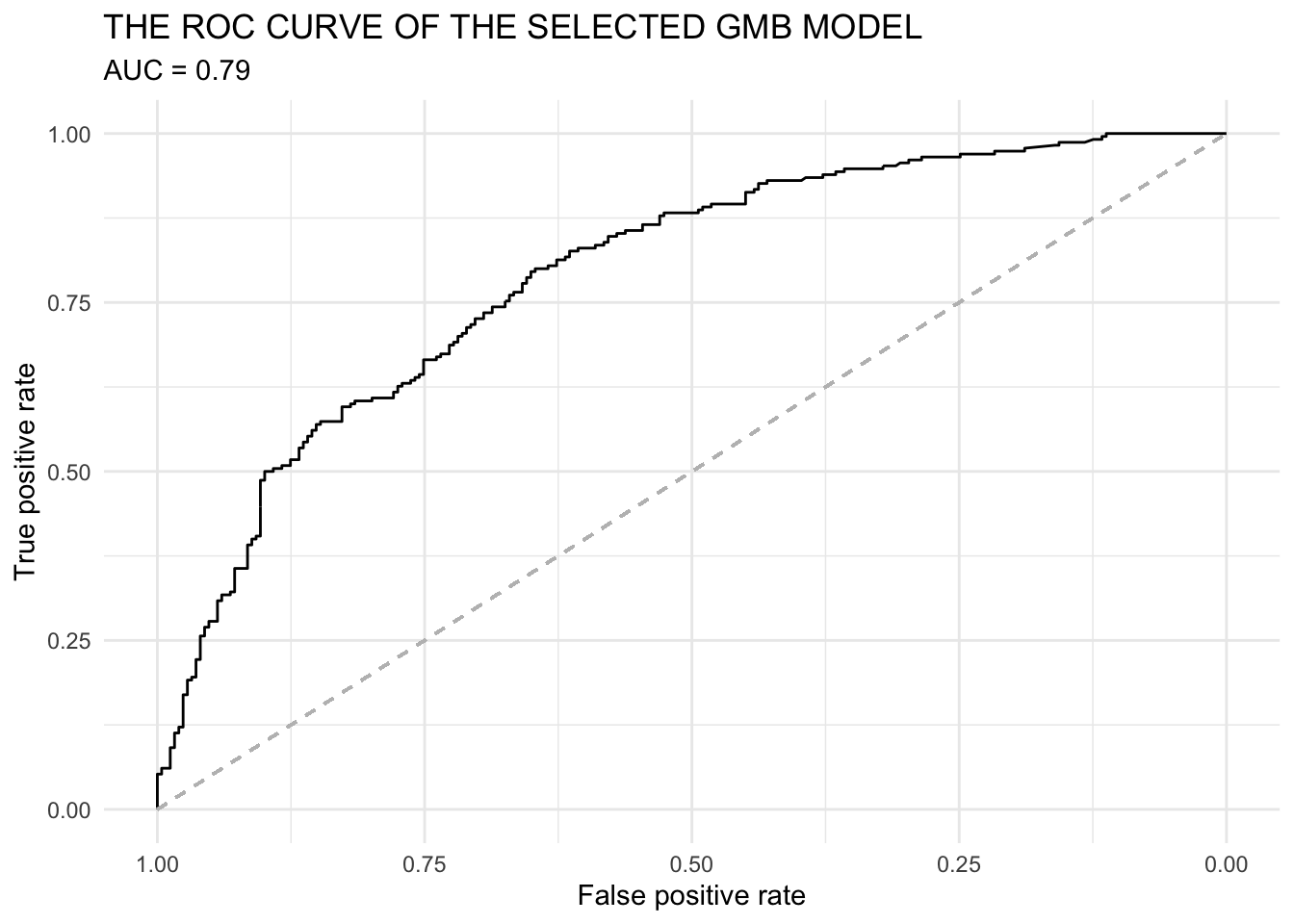


## [1] 98.44934

## Confusion Matrix and Statistics

##

## Reference

## Prediction No Yes

## No 161 46

## Yes 88 184

##

## Accuracy : 0.7203

## 95% CI : (0.6777, 0.76)

## No Information Rate : 0.5198

## P-Value [Acc > NIR] : < 2.2e-16

##

## Kappa : 0.4435

##

## Mcnemar's Test P-Value : 0.0003973

##

## Sensitivity : 0.6466

## Specificity : 0.8000

## Pos Pred Value : 0.7778

## Neg Pred Value : 0.6765

## Prevalence : 0.5198

## Detection Rate : 0.3361

## Detection Prevalence : 0.4322

## Balanced Accuracy : 0.7233

##

## 'Positive' Class : No

##

## Reference

## Prediction No Yes

## No 161 46

## Yes 88 184


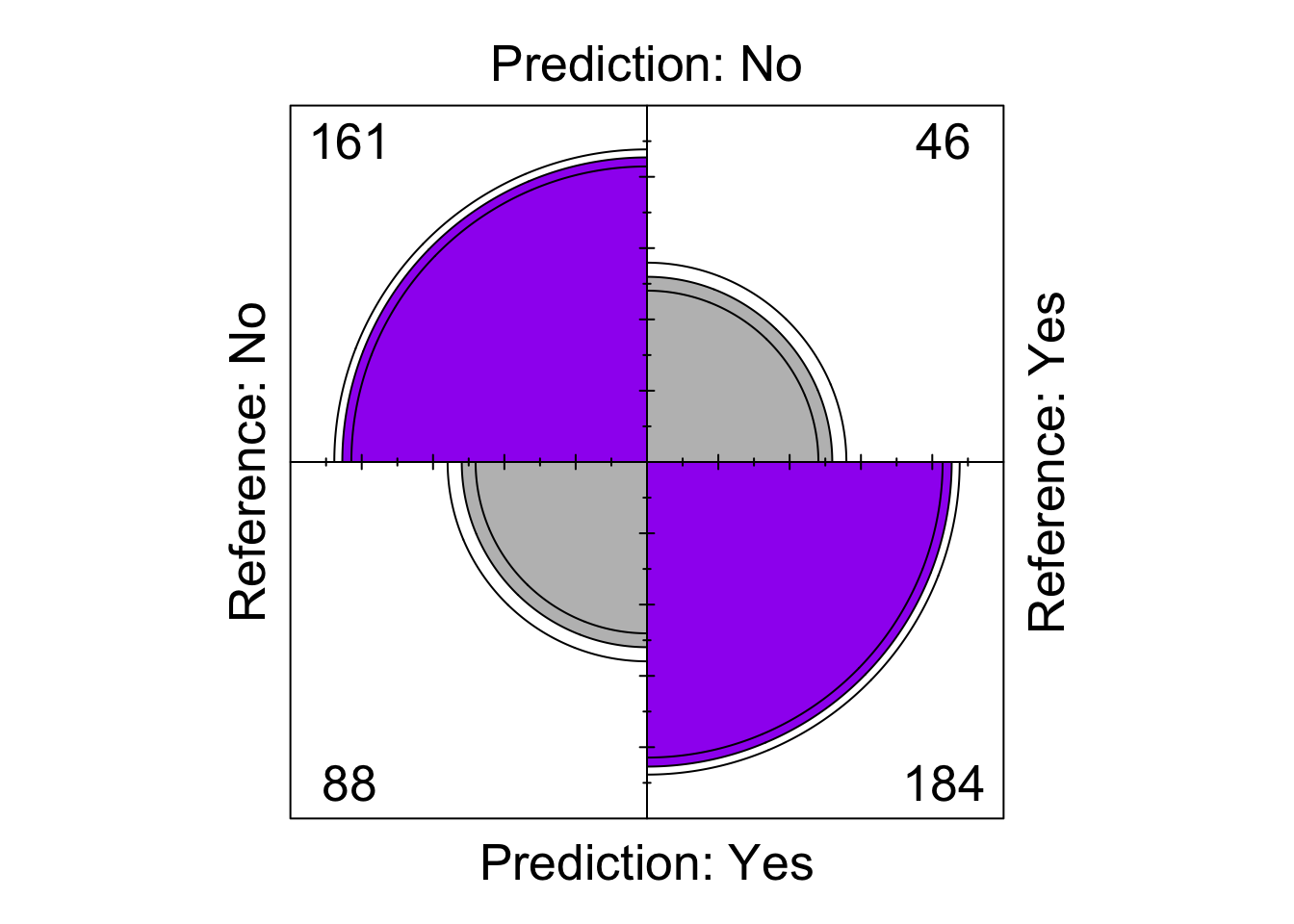


## Min. 1st Qu. Median Mean 3rd Qu. Max.

## 0.07037 0.23561 0.42578 0.49524 0.76230 0.98862


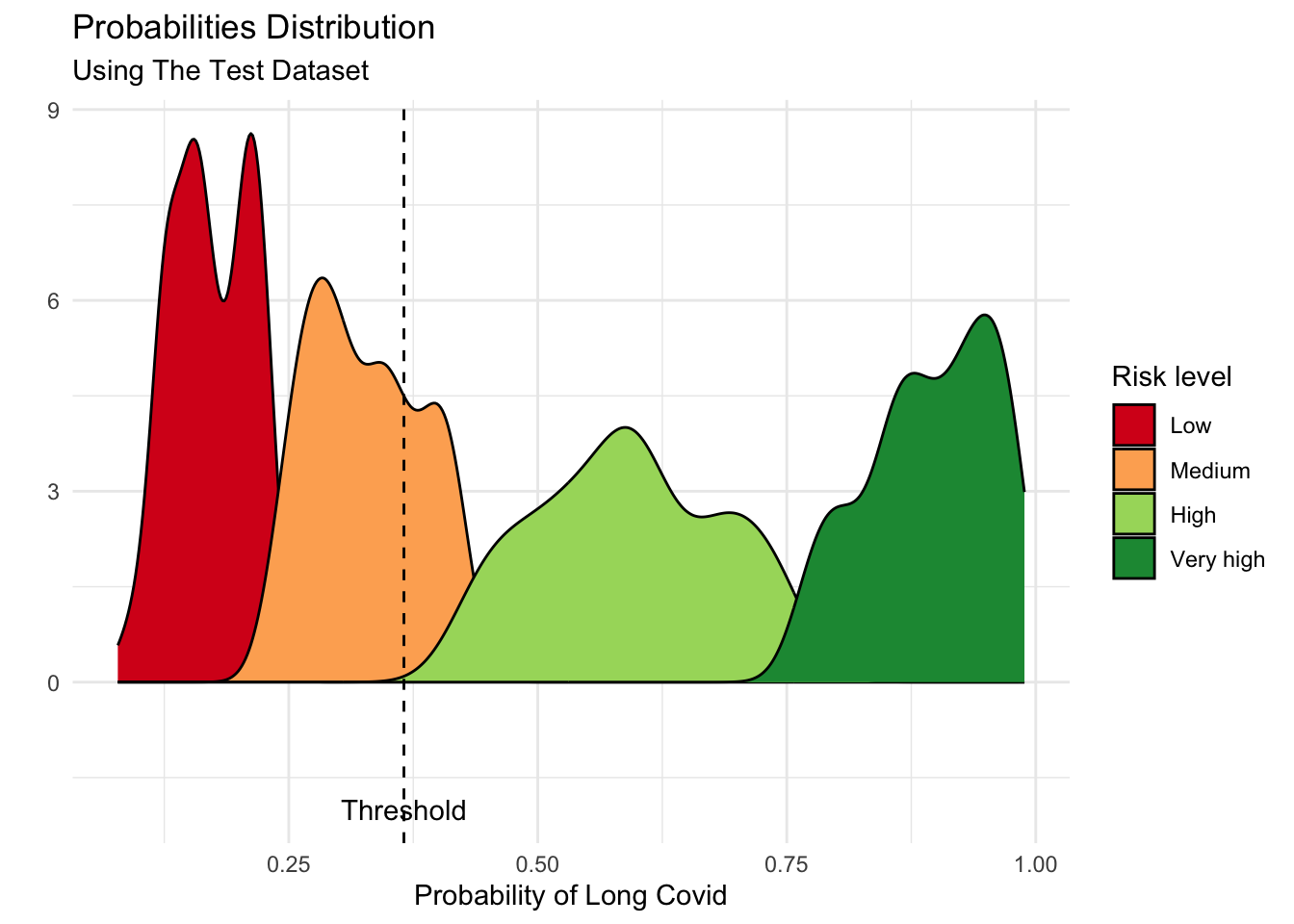


Metrics at different thresholds


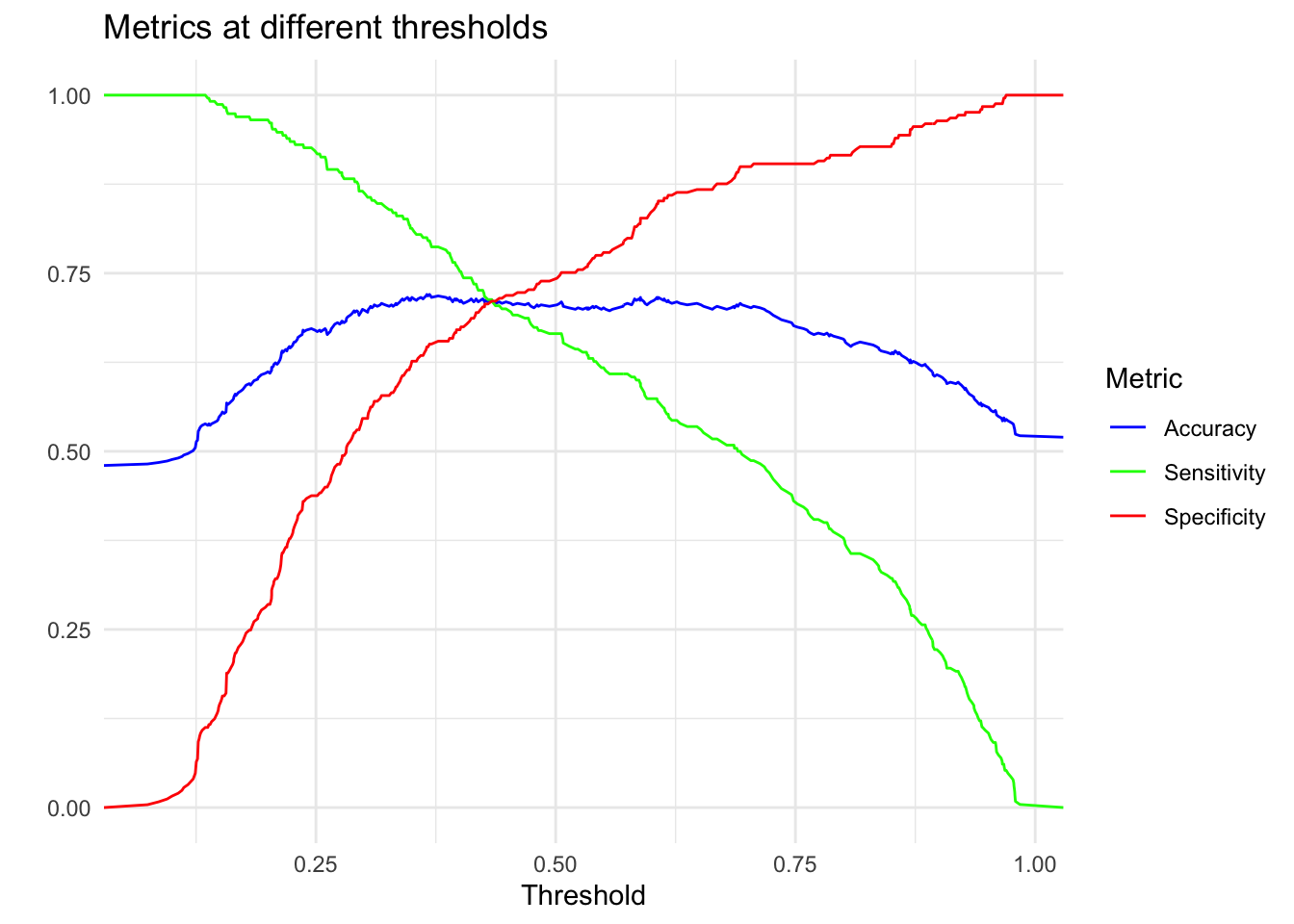


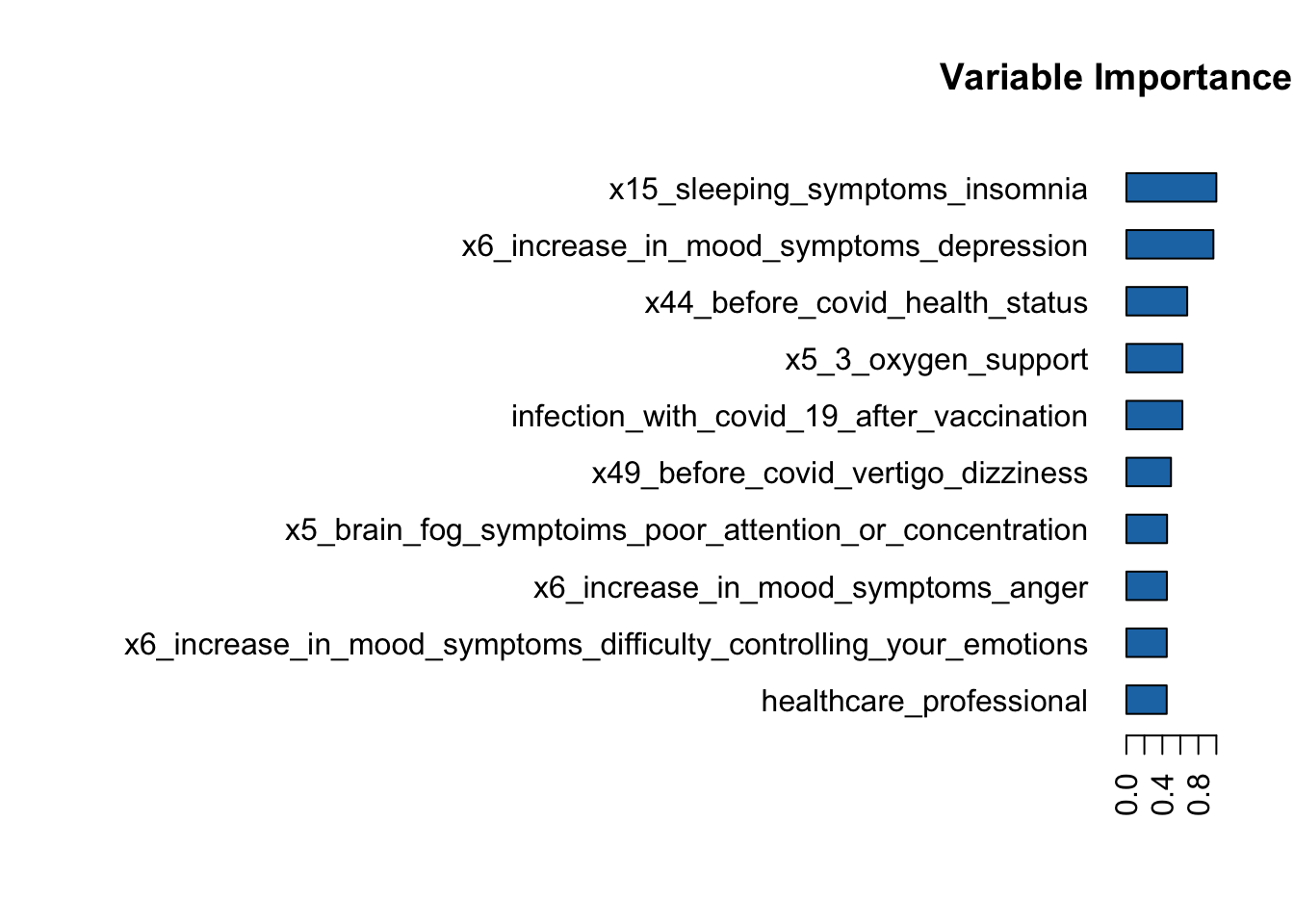


## variable

## 1 x15_sleeping_symptoms_insomnia

## 2 x6_increase_in_mood_symptoms_depression

## 3 x44_before_covid_health_status

## 4 x5_3_oxygen_support

## 5 infection_with_covid_19_after_vaccination

## 6 x49_before_covid_vertigo_dizziness

## 7 x5_brain_fog_symptoims_poor_attention_or_concentration

## 8 x6_increase_in_mood_symptoms_anger

## 9 x6_increase_in_mood_symptoms_difficulty_controlling_your_emotions

## 10 healthcare_professional

## 11 paracetamol

## 12 x10_headaches_symptoms_headaches

## 13 x32_muscle_and_joint_symptoms_muscle_aches

## 14 x32_muscle_and_joint_symptoms_joint_pain

## 15 x16_insomnia_description_waking_up_several_times_during_the_night

## 16 x57_migraine

## 17 x41_gastrointestinal_symptoms_abdominal_pain

## 18 x28_respiratory_symptoms_tightness_of_chest

## 19 x45_before_covid_mental_health_diagnsosis

## 20 aspirin

## 21 x23_cvs_symptoms_thumping_or_skipping_beats

## 22 x23_cvs_symptoms_tachycardia

## 23 x3_memory_loss_symptoms_or_forgetting_you_re_in_the_middle_of_a_task

## 24 omega_3

## 25 naproxen

## 26 x28_respiratory_symptoms_cough_with_mucus_production

## 27 anti_type_two_histamine

## 28 x55_tinnitus_experience

## 29 x4_1_pre_existing_conditions_vision_near_sighted_far_sighted

## 30 x4_1_pre_existing_conditions_irritable_bowel_syndrome_ibs

## 31 x5_brain_fog_symptoims_abstracting

## 32 anti_oxidants

## 33 x4_1_pre_existing_conditions_food_allergies

## 34 x4_1_pre_existing_conditions_diabetes_type_2

## 35 x4_1_pre_existing_conditions_nightmares

## relative_importance scaled_importance percentage

## 1 310.88535 1.0000000 0.07194507

## 2 300.77179 0.9674685 0.06960459

## 3 210.23155 0.6762350 0.04865177

## 4 193.68498 0.6230110 0.04482257

## 5 193.52687 0.6225024 0.04478598

## 6 154.14745 0.4958337 0.03567279

## 7 140.59073 0.4522269 0.03253550

## 8 139.92230 0.4500769 0.03238081

## 9 139.49030 0.4486873 0.03228084

## 10 139.26865 0.4479743 0.03222954

## 11 131.42113 0.4227318 0.03041347

## 12 130.33002 0.4192221 0.03016096

## 13 127.35087 0.4096393 0.02947153

## 14 122.56542 0.3942464 0.02836408

## 15 117.54551 0.3780992 0.02720237

## 16 117.20979 0.3770193 0.02712468

## 17 116.57343 0.3749724 0.02697742

## 18 116.04877 0.3732848 0.02685600

## 19 115.03330 0.3700184 0.02662100

## 20 114.37899 0.3679137 0.02646958

## 21 105.91811 0.3406983 0.02451156

## 22 96.85479 0.3115450 0.02241413

## 23 93.08763 0.2994275 0.02154233

## 24 88.80643 0.2856565 0.02055158

## 25 87.87124 0.2826484 0.02033516

## 26 83.08186 0.2672428 0.01922680

## 27 77.56515 0.2494976 0.01795012

## 28 77.46288 0.2491686 0.01792645

## 29 76.41331 0.2457926 0.01768356

## 30 75.77315 0.2437334 0.01753542

## 31 71.72748 0.2307200 0.01659917

## 32 70.69671 0.2274044 0.01636063

## 33 68.01524 0.2187792 0.01574008

## 34 64.13979 0.2063133 0.01484323

## 35 52.75788 0.1697021 0.01220923


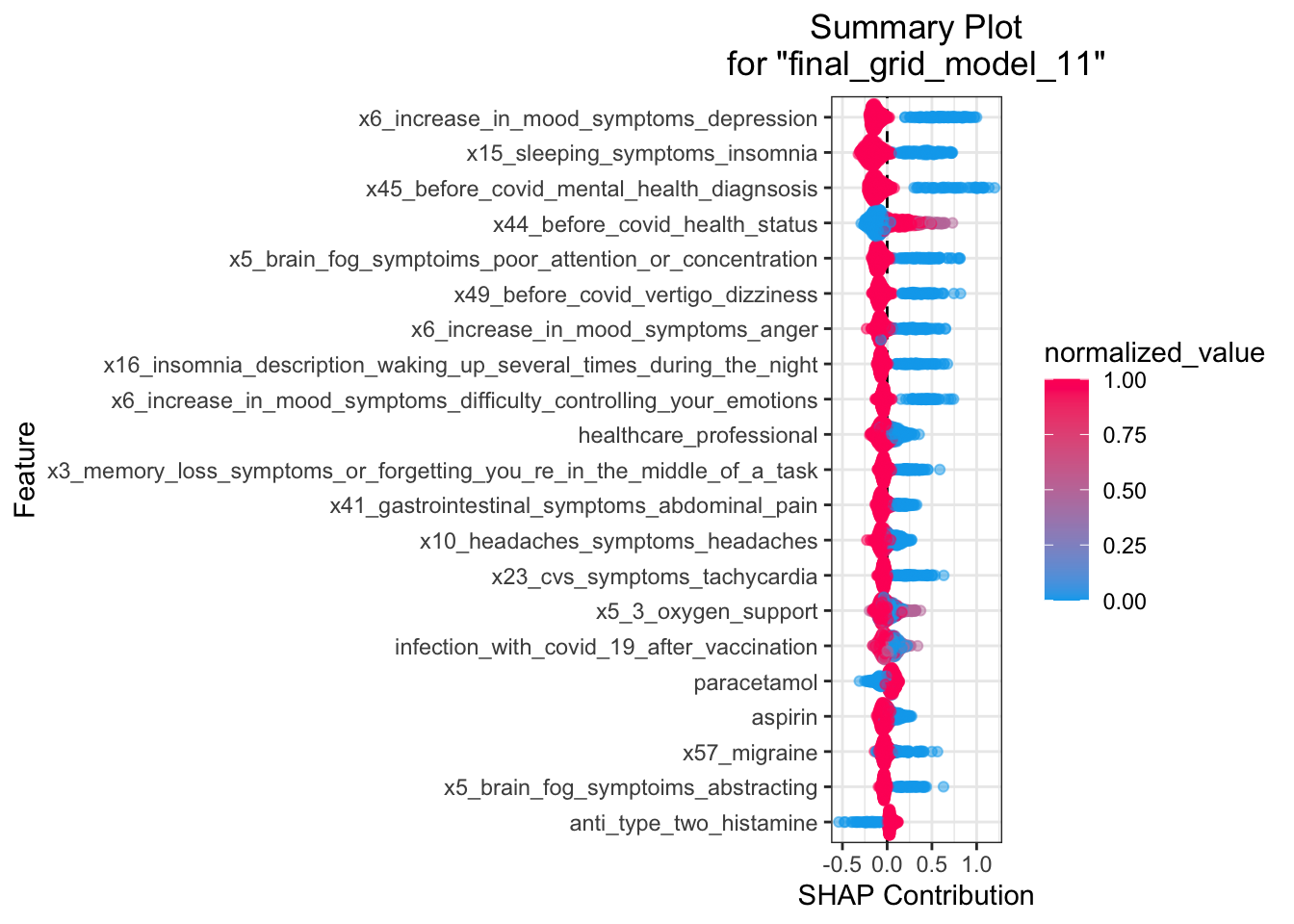


## Gains/Lift Table: Avg response rate: 47.94 %, avg score: 48.32 %

## group cumulative_data_fraction lower_threshold lift cumulative_lift

## 1 1 0.01042209 0.978748 2.085870 2.085870

## 2 2 0.02032308 0.973789 2.085870 2.085870

## 3 3 0.03022408 0.970068 2.085870 2.085870

## 4 4 0.04012507 0.966527 2.085870 2.085870

## 5 5 0.05002606 0.962967 2.085870 2.085870

## 6 6 0.10005211 0.944656 2.085870 2.085870

## 7 7 0.15007817 0.919279 2.085870 2.085870

## 8 8 0.20010422 0.884841 2.085870 2.085870

## 9 9 0.30015633 0.764628 2.085870 2.085870

## 10 10 0.40020844 0.511307 1.868591 2.031550

## 11 11 0.50026055 0.366092 0.771337 1.779507

## 12 12 0.60187598 0.280928 0.342297 1.536861

## 13 13 0.69984367 0.220842 0.244091 1.355893

## 14 14 0.80093799 0.181936 0.258046 1.217323

## 15 15 0.90515894 0.146215 0.187728 1.098774

## 16 16 1.00000000 0.064046 0.057304 1.000000

## response_rate score cumulative_response_rate cumulative_score

## 1 1.000000 0.982453 1.000000 0.982453

## 2 1.000000 0.975972 1.000000 0.979296

## 3 1.000000 0.971591 1.000000 0.976772

## 4 1.000000 0.968084 1.000000 0.974628

## 5 1.000000 0.964682 1.000000 0.972660

## 6 1.000000 0.953415 1.000000 0.963037

## 7 1.000000 0.933644 1.000000 0.953239

## 8 1.000000 0.903387 1.000000 0.940776

## 9 1.000000 0.829573 1.000000 0.903709

## 10 0.895833 0.639554 0.973958 0.837670

## 11 0.369792 0.424745 0.853125 0.755085

## 12 0.164103 0.320616 0.736797 0.681733

## 13 0.117021 0.247607 0.650037 0.620962

## 14 0.123711 0.200844 0.583604 0.567935

## 15 0.090000 0.163569 0.526770 0.521376

## 16 0.027473 0.118386 0.479416 0.483156

## capture_rate cumulative_capture_rate gain cumulative_gain

## 1 0.021739 0.021739 108.586957 108.586957

## 2 0.020652 0.042391 108.586957 108.586957

## 3 0.020652 0.063043 108.586957 108.586957

## 4 0.020652 0.083696 108.586957 108.586957

## 5 0.020652 0.104348 108.586957 108.586957

## 6 0.104348 0.208696 108.586957 108.586957

## 7 0.104348 0.313043 108.586957 108.586957

## 8 0.104348 0.417391 108.586957 108.586957

## 9 0.208696 0.626087 108.586957 108.586957

## 10 0.186957 0.813043 86.859149 103.155005

## 11 0.077174 0.890217 -22.866282 77.950747

## 12 0.034783 0.925000 -65.770346 53.686147

## 13 0.023913 0.948913 -75.590888 35.589287

## 14 0.026087 0.975000 -74.195428 21.732271

## 15 0.019565 0.994565 -81.227174 9.877412

## 16 0.005435 1.000000 -94.269589 0.000000

## kolmogorov_smirnov

## 1 0.021739

## 2 0.042391

## 3 0.063043

## 4 0.083696

## 5 0.104348

## 6 0.208696

## 7 0.313043

## 8 0.417391

## 9 0.626087

## 10 0.793023

## 11 0.749076

## 12 0.620696

## 13 0.478443

## 14 0.334359

## 15 0.171742

## 16 0.000000

##

##

## Confusion Matrix

## ================

##

## > Confusion matrix shows a predicted class vs an actual class.

##

##

##

## final_grid_model_11

## -------------------

##

## | | No | Yes | Error | Rate

## |:---:|:---:|:---:|:---:|:---:|

## | **No** |131 | 118 | 0.473895582329317 | =118/249 |

## | **Yes** |27 | 203 | 0.117391304347826 | =27/230 |

## | **Totals** |158 | 321 | 0.302713987473904 | =145/479 |

##

##

## Learning Curve Plot

## ===================

##

## > Learning curve plot shows the loss function/metric dependent on number of iterations or trees for tree-based algorithms. This plot can be useful for determining whether the model overfits.


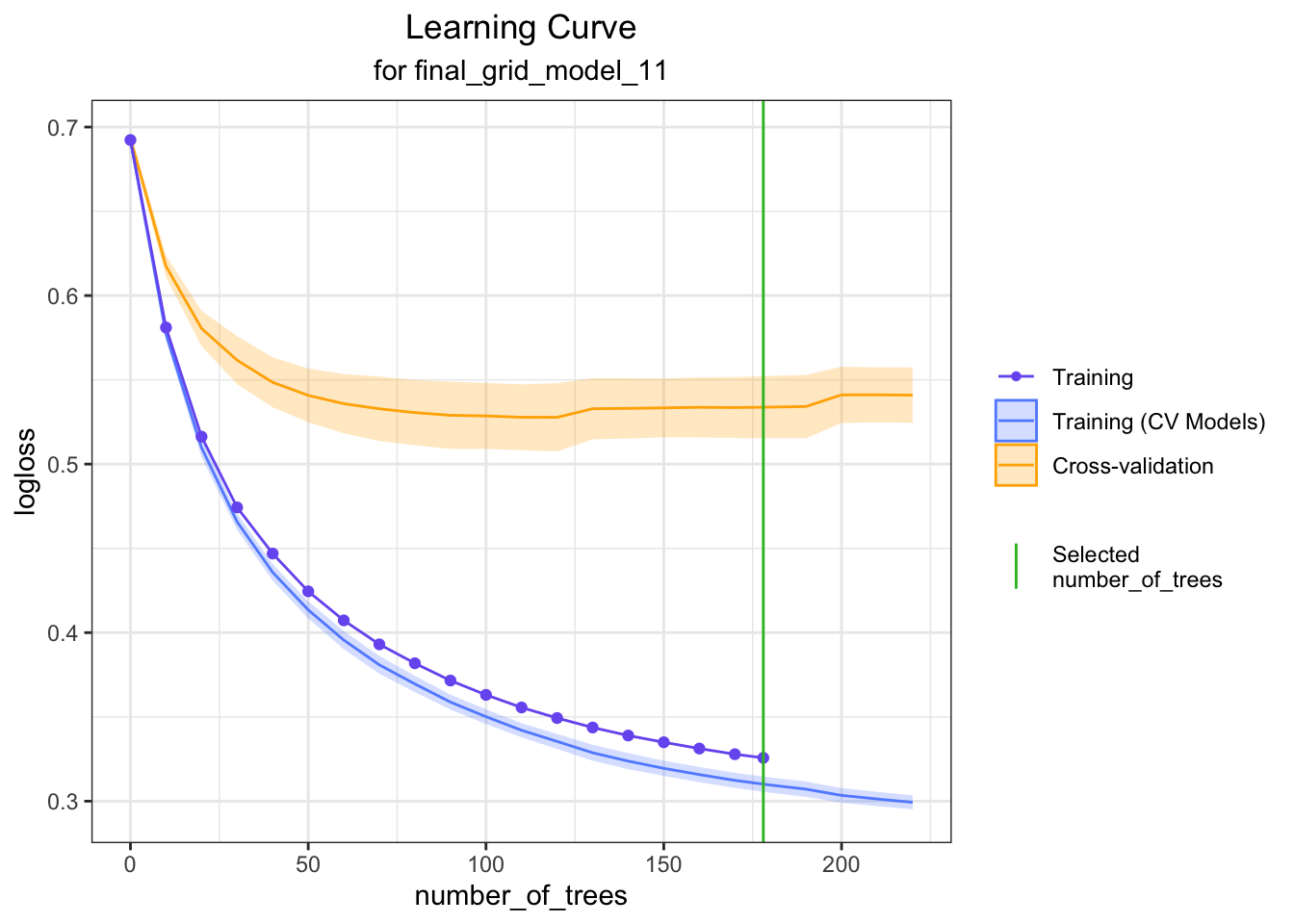


##

##

## Variable Importance

## ===================

##

## > The variable importance plot shows the relative importance of the most important variables in the model.


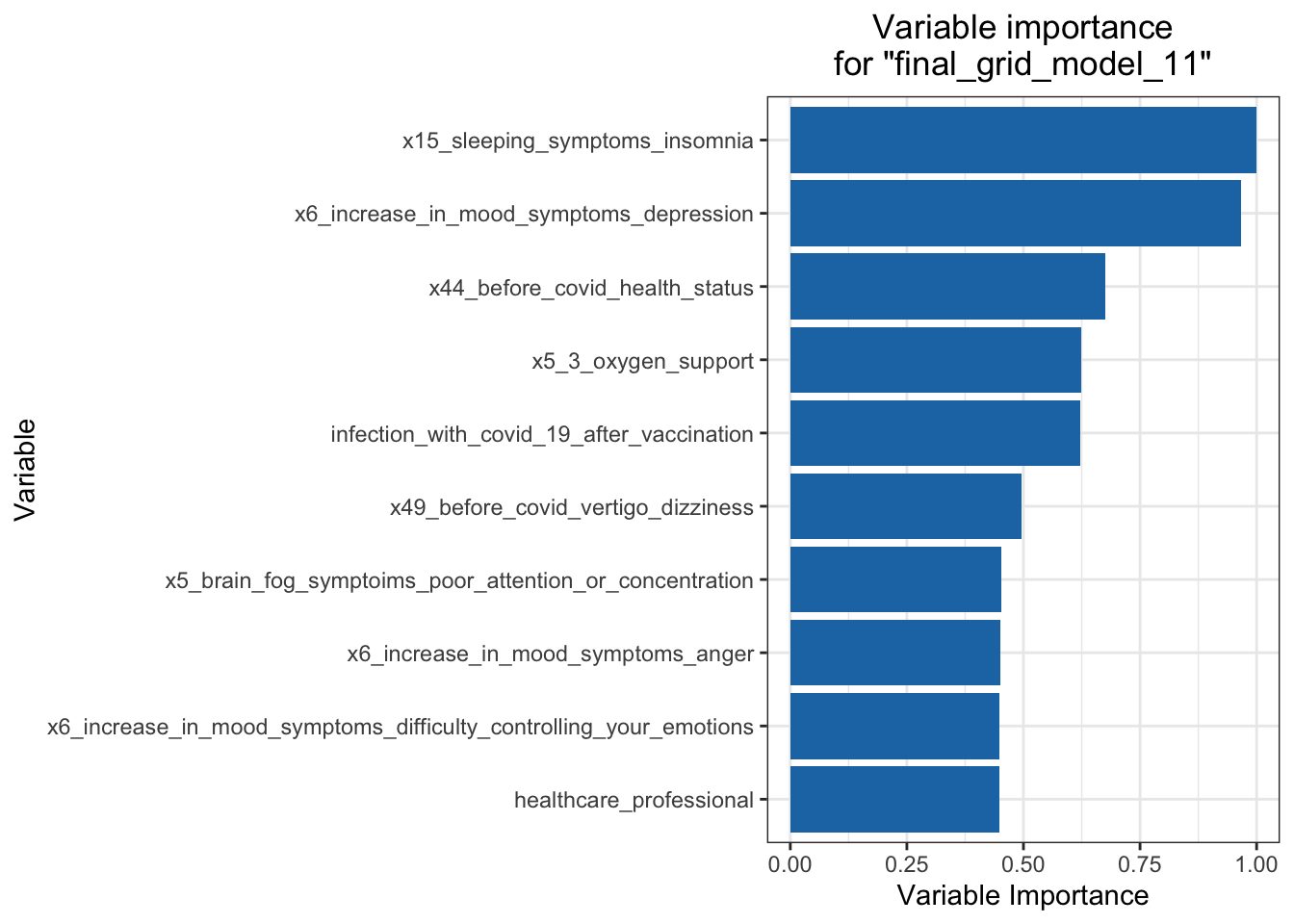


##

##

## SHAP Summary

## ============

##

## > SHAP summary plot shows the contribution of the features for each instance (row of data). The sum of the feature contributions and the bias term is equal to the raw prediction of the model, i.e., prediction before applying inverse link function.


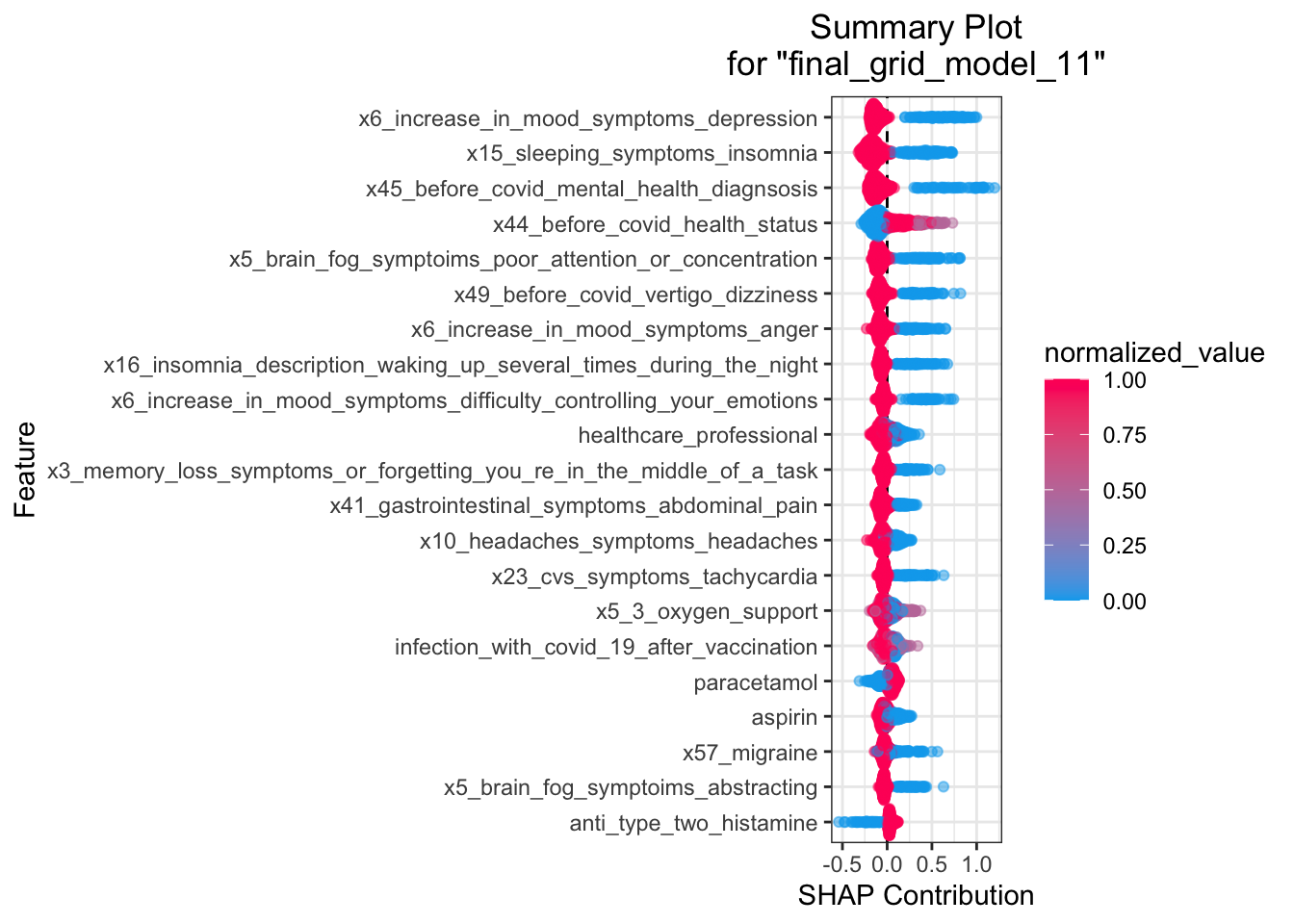


##

##

## Partial Dependence Plots

## ========================

##

## > Partial dependence plot (PDP) gives a graphical depiction of the marginal effect of a variable on the response. The effect of a variable is measured in change in the mean response. PDP assumes independence between the feature for which is the PDP computed and the rest.


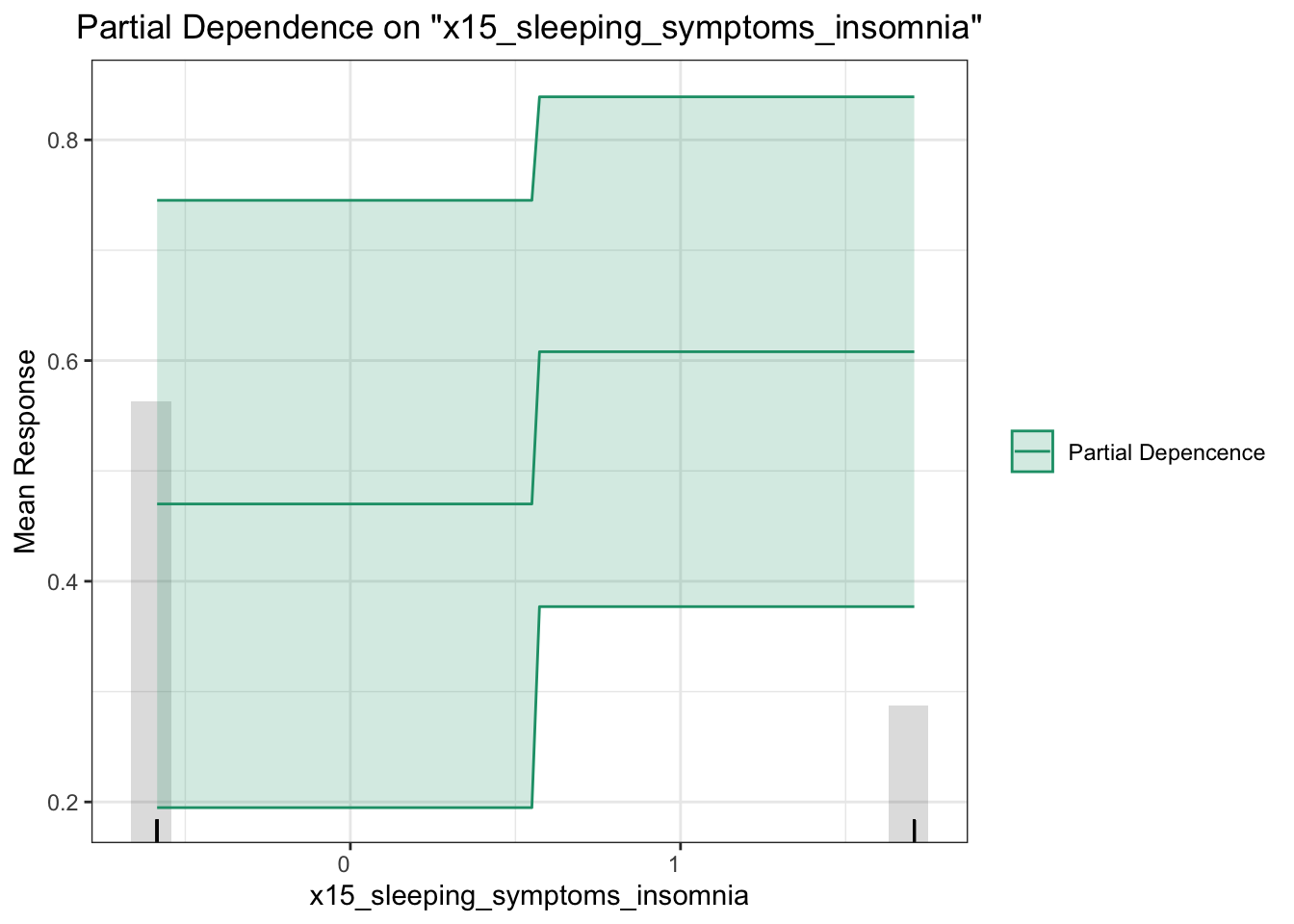

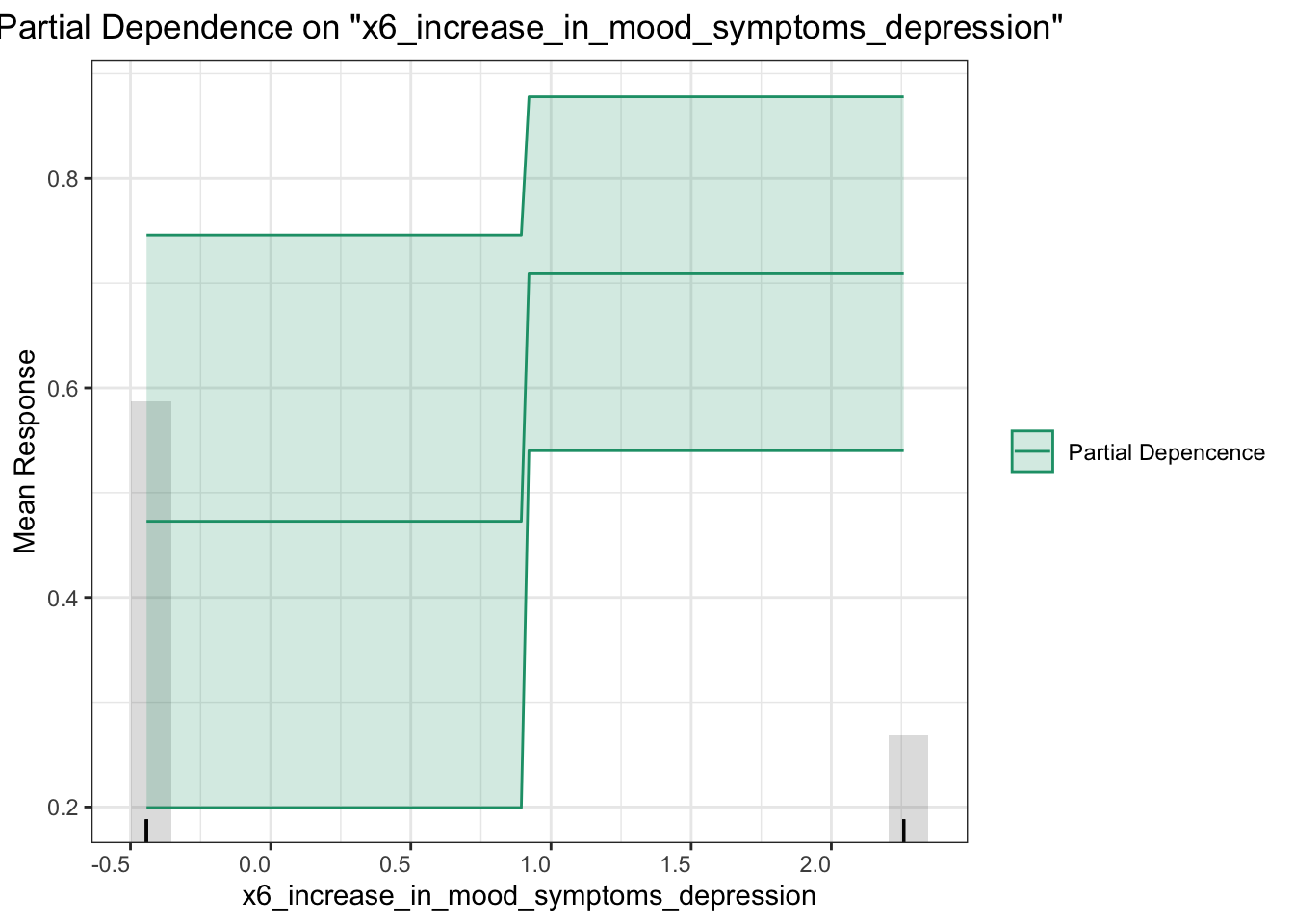

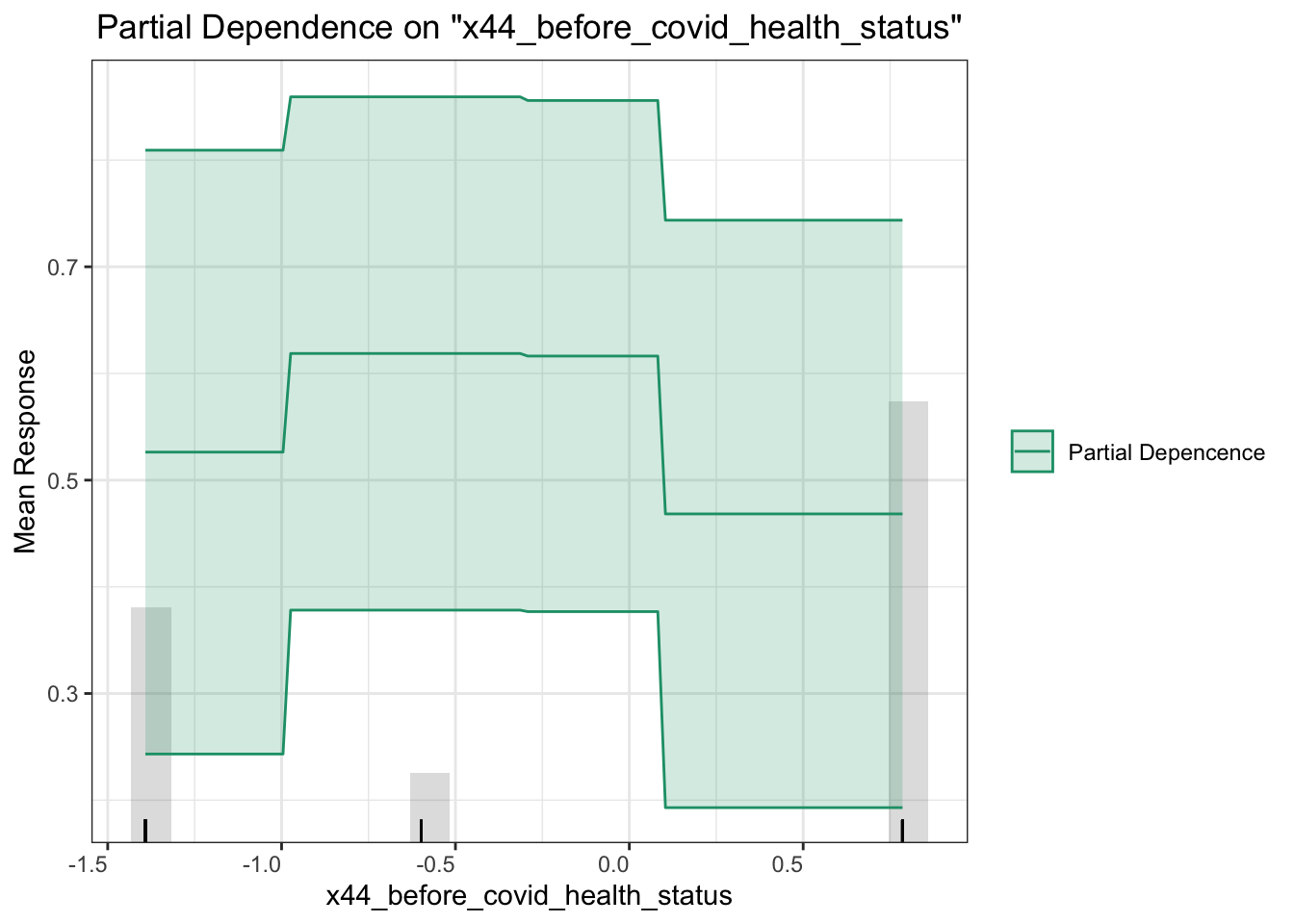

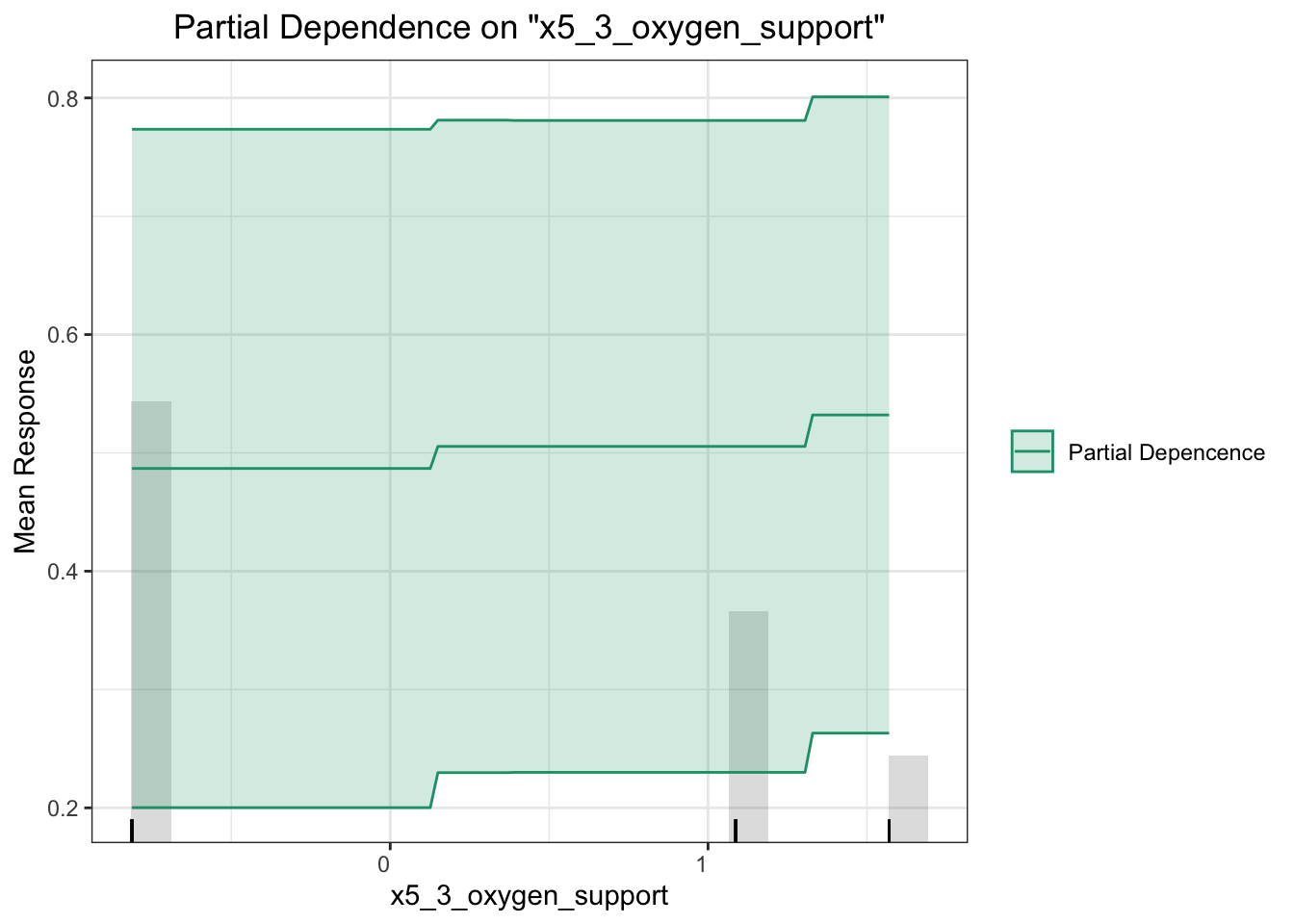

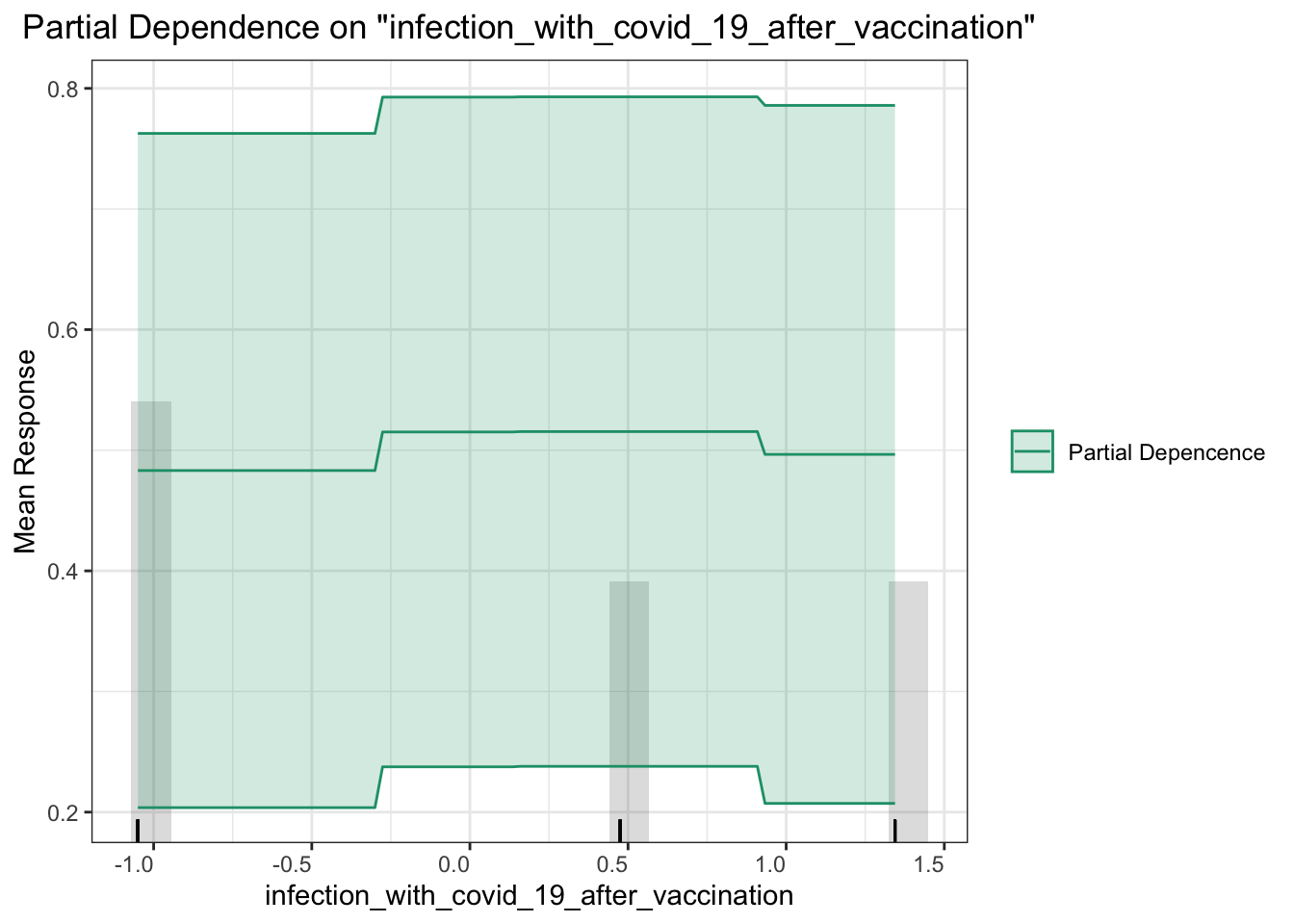

Supplement: Supplementary file 1 [file Data_Sheet_1.docx]
