# Supplementary material for: Deep learning analysis of long COVID and vaccine impact in low- and middle-income countries (LMICs): development of a risk calculator in a multicentric study
Source: Front Public Health. 2025 Jun 26;13:1416273. doi: 10.3389/fpubh.2025.1416273 (PMC12240947; doi:10.3389/fpubh.2025.1416273)
Supplement: Supplementary file 2 [file Data_Sheet_2.docx]

| **First Name** | **Last Name** | **Affiliation** | **Country** | **ORCID ID** | **Email** | **Role in the study** |
| --- | --- | --- | --- | --- | --- | --- |
| Ahmed | Shaheen | Alexandria Faculty of Medicine | Egypt | 0000-0001-9731-5065 | ahmeds1999haheen@gmail.com | Principal Investigator |
| Nour | Shaheen | Alexandria Faculty of Medicine | Egypt | https://orcid.org/0000-0001-8663-4044 | nourshaheen40@gmail.com | Co-Principal Investigator |
| Fahimeh | Saeed | Psychosis Research Center, University of Social Welfare and Rehabilitation Sciences, Tehran, Iran | Iran |  | Fa.saeed@uswr.ac.ir | National/Regional Leader |
| Sheikh | Shoib | Psychosis Research Centre, University of Social Welfare and Rehabilitation Sciences, Tehran, Iran  Department of Health Services, Srinagar, India. | India |  | Sheikhshoib22@gmail.com | Supervisor |
| Mudathiru | Buhari | University of South Florida, Division of Infectious Disease, Tampa, Florida, USA. |  |  | bayidc@gmail.com | Supervisor |
| Vishal ‎ | Bharmauria | University of South Florida, Department of Neurosurgery, Brian and spine, Tampa, Florida. | USA | https://orcid.org/0000-0002-9511-2827 | vishalbharmauria@usf.edu | Supervisor |
| Oliver | Flouty | University of South Florida, Department of Neurosurgery, Brian and spine, Tampa, Florida. | USA | https://orcid.org/0000-0001-5990-7297 | oliverflouty@gmail.com | Supervisor |
| Karim | Alasar | Mansoura University, Faculty of Medicine, Egypt | Egypt | 0009-0000-4076-007X | Karimalasar1@gmail.com | Data Collection |
| Mohamed | Mortagy | Department of Internal Medicine, New Giza University School of Medicine, New Giza, Egypt | Egypt |  |  | hospital Leader |
| Ismail | Mohamadin | New Giza University, School of Medicine | Egypt | 0009-0002-0401-7355 | ismail.mohamadin@ngu.edu.eg | Local Team Leader |
| Abdullah | Malikzai | Kabul University of Medical Sciences | Afghanistan | 0000-0002-9440-4393 | abdullahmkz2022@gmail.com | Data Collection |
| Ahmad | Bebi | University of Aleppo, Faculty of Medicine, Aleppo, Syria |  |  | ahmad.bebi@hotmail.com | Data Collection |
| Ahmed | Alrifaee | Alexandria Faculty of Medicine, Alexandria, Egypt |  |  | Ahmedsamir19700@gmail.com | Data Collection |
| Laibah Arshad | Khan | University of Mississippi Medical Center | USA | 0000-0002-9361-572X | laibahkhan12@gmail.com | Data Collection |
| Omnia Gamal | Hagag | Faculty of Medicine zagazig University | Egypt | 0009-0001-3341-4192 | omneiagamal131@gmail.com | Data Collection |
| Sadhu Aishwarya Reddy | | Osmania Medical College/osmania general hospital | India | 0009-0000-0918-0376 | aishwaryareddysadhu@gmail.com | Data Collection |
| Bhargavi R. | Budihal | BGS Global Institute of Medical Sciences | India | 0000-0002-2140-8748 | bhargavibudihal2001@gmail.com | Local Team Leader |
| Reem | Sayad | Faculty of Medicine, Assiut University. | Egypt | 0000-0002-4378-2714 | Reem.17289806@med.aun.edu.eg | Data Collection |
| Leena | Saeed | National Ribat University | Khartoum, Sudan | 0000-0001-5332-930X | Leenasaeed95@hotmail.com | Data Collection |
| Zinelabedin | Mohamed | Faculty of Medicine, Tobruk University, Tobruk, Libya | Libya | 0000-0003-0133-809X | Zen_Zen47@yahoo.com | local team Leader |
| Douaa | Albelal | Faculty of Medicine Hama University | Syria | 0000-0001-7899-8438 | douaa.albelal98@gmail.com | Data Collection |
| Abdalla | Hadhoud | Alexandria Faculty of Medicine | Egypt | 0000-0002-1410-7680 | Abdullah.hadhoud73@gmail.com | Data Collection |
| Muhammad Besher | Shabouk | Faculty of Medicine, University of Aleppo, Aleppo, Syria | Syria | 0000-0002-2636-1504 | mu.besher_shabouk@outlook.com | Data Collection |
| Ahmed | Saadoun | Faculty of Medicine, Mansoura University, Egypt | Egypt | 0009-0000-4330-2648 | ahmedattifsadoon1@gmail.com | Data Collection |
| Eman | E. Alshial | Biochemistry Department, Faculty of Science, Damanhour University, Al Buhayrah, Egypt | | | emanalshial12@gmail.com | Data Collection |
| Aleenah | Mohsin | King Edward Medical University, Lahore, Pakistan | Pakistan | 0000-0002-3441-619X | aleenahmohsin10@gmail.com | Data Collection |
| Muhannad | Alnaasan | University of Aleppo Faculty of Medicine |  | 0000-0002-9821-0713 | mohannad02000@gmail.com | Data Collection |
| Rama | Sakkour | Faculty of Pharmacy, Tishreen University, Latakia, Syria |  | 0000-0002-3990-0044 | ramasakour77@gmail.com | Data Collection |
| Umaima | Rafiq | Liaquat National Hospital and Medical College |  |  | rafiqumaima@gmail.com | Data Collection |
| Areeba | Abdullah | Liaquat National Hospital and Medical College | Pakistan | 0000-0002-0937-2076 | areebaabdullah97@gmail.com | Local Team Leader |
| Ethem | Unal | University of Health Sciences Türkiye, Hamidiye Faculty of Medicine, İstanbul Türkiye | Türkiye |  | drethemunal@gmail.com | Local Team Leader |
| Kashifa | Haqqani Zara | Dr. VRK Women's Medical College, Hyderabad | India | 0009-0006-0701-7931 | kashifahaqqani@gmail.com | Local Team Leader |
| Areeba | Abdullah | Liaquat National Hospital and Medical College |  | 0000-0002-0937-2076 | areebaabdullah97@gmail.com | Data Collection |
| Yash | Kumar | Liaquat National Medical College | Pakistan | 0000-0002-8909-0887 | yashrathi2010@yahoo.com | Local Team Leader |
| Kanika | Handu | Esic Medical College & PGIMSR | India | 0000-0003-1637-7673 | handukanika@gmail.com | National/Regional Leader |
| Akshita | Suleria | Government Medical College Patiala, India | India | 0009-0006-9720-8247 | dr.akshitasuleria@gmail.com akshitasuleria@gmail.com | Data Collection |
| Ann Mariam | Stanley | Christian Medical College, Ludhiana | India | 0009-0009-0422-2202 | annmariamstanley09@gmail.com | Data Collection |
| Sai Santhosha Mrudula | Alla | Andhra Medical College | India | 0000-0001-5700-6743 | mrudualla123@gmail.com | Data Collection |
| Deekshitha | Alla | Andhra Medical College | India | 0000-0003-4234-6141 | adeekshitha123@gmail.com | Data Collection |
| Priyanka | Kataria | Government Medical College, Nagpur | India | 0009-0005-9774-8879 | priyankakataria246@gmail.com | Data Collection |
| Gurinder Jeet | Singh | GMC Patiala | India |  | gurinderjeet.singh08@gmail.com | Data Collection |
| Brundha | Arya | BGS Global Institute of Medical Sciences | India |  | brundhaarya@gmail.com | Data Collection |
| Mehreen | Taqi | Dr. VRK Women's Medical College | India | 0009-0007-4401-2328 | batool14032001@gmail.com | Data Collection |
| Brahmjot | Kaur | Government Medical College Patiala, India | India | 0009-0009-7443-7823 | Brahmjot01@gmail.com | Data Collection |
| Asem | Shaikh Alnajjarin | Hama Faculty of Medicine, Hama, Syria | Syria in | 0009-0007-1493-6722 | hp.asem98@gmail.com | Data Collection |
| Aliae | Mohamed Hussein | Assiut University, Assiut Faculty of Medicine | Egypt | 0000-0002-7111-2195 | aliaehussein@aun.edu.eg | Data Collection |
| Rahma | Alkurdi | Hama national hospital, Syria | Syria |  | alkurdirahma@gmail.com | Data Collection |
| Jawaria | Sattar | Rawalpindi Medical University, Rawalpindi | Pakistan | 0009-0006-6920-6893 | jawarias1234@gmail.com | Data Collection |
| Mohammed Amir | Rais | Faculty of Medicine of Algiers |  |  | raismohammedamir@gmail.com | Local Team Leader |
| Armaghan | Ayub | Brigham and Women’s Hospital | USA | 0009-0002-7772-8502 | Armaghanayub786@gmail.com | Data Collection |
| Ahmed K. | Awad | Faculty of Medicine, Ain-Shams University, Cairo, Egypt |  |  | ahmedkawad@gmail.com | National/Regional Leader |
| Sara | Aldali |  |  |  | sara_aldali1998@hotmail.com | Data Collection |
| Hamed | Kozom | University of Aleppo, Faculty of Medicin |  |  | Hamed96kozom@gmail.com | Data Collection |
| Koohyar | Ahmadzadeh | Physiology Research Center, Iran University of Medical Sciences, Tehran, Iran | Iran | 0000-0001-8056-1761 | koohyar.ahmadzadeh@gmail.com | Data Collection |
| Darshini | Shah | GCS Medical College, Hospital & Research Centre | India | 0009-0003-2175-2877 | darshini13shah@gmail.com | Data Collection |
| Amal | Farghaly |  |  |  | aml.abdo118@gmail.com | Data Collection |
| Eman | Akhtar | RMU, Rawalpindi, Pakistan | Pakistan | 0009-0005-9936-4596 | emanakhtar0@gmail.com | Data Collection |
| Eman | A. Soliman |  |  |  | nona.ali171999@gmail.com | Data Collection |
| Eman | A. Soliman |  |  |  | nona.ali171999@gmail.com | Data Collection |
| Aiman | Idrees | King Edward Medical University, Mayo Hospital, Lahore | Pakistan | 0000-0001-5566-2659 | aimanidrees99@gmail.com | Data Collection |
| Joshua | Chacko | Dr |  |  | joshuachacko53@gmail.com | Data Collection |
| Omnia Gamal | Hagag | Faculty of Medicine zagazig University |  |  | omniafares946@gmail.com | Data Collection |
| Sarah | ElReesh | Faculty of Medicine Zagazig | Egypt | 0009-0005-2327-9651 | elreeshsarah@gmail.com | Data Collection |
| Alia | Elgamel | Faculty of Medicine, Mansoura University, Egypt |  | 0000-0001-9930-7501 | aliaelgamel@yahoo.com | Data Collection |
| Amro | Eliwa | Faculty of Medicine, Mansoura University, Egypt |  |  | Amrhatem1998@yahoo.com | Data Collection |
| Syed Atif | Ul Haq | LIAQUAT NATIONAL HOSPITAL AND MEDICAL COLLEGE | Pakistan | 0000-0001-9819-9844 | syedatifulhaq@gmail.com | Local Team Leader |
| Pavani B. | Agarwal | BGS Global Institute of Medical Sciences | India |  | paviagarwal17@gmail.com | Data Collection |
| Aya | Jarrous | Damascus University- Faculty of Medicine | Syria | 0000-0002-5809-1879 | ayajaros@gmail.com | Data Collection |
| Alaa | W. Ibrahim | Faculty of Medicine, Assiut University, Egypt | Egypt | 0000-0003-3342-1330 | dralaawalid310@gmail.com | Data collection |
| Mohammad Mousa | Dello | Aleppo Faculty of Medicine, Aleppo, Syria |  |  | mousadellow@gmail.com | Data Collection |
| Ali Maisam | Eshraqi | Kabul University of Medical Sciences |  | 0000-0003-0709-1200 | alimaisame@gmail.com | Data Collection |
| Amal | Farghaly | Faculty of Medicine Ain Shams University |  |  | aml.abdo118@gmail.com | Data Collection |
| Soode | Tajik Esmaeeli | Assistant professor of psychiatry, Fellowship of Psychosomatic Medicine, Rasool-e Akram Hospital, Mental Health Research Center, Psychosocial Health Research Institute, Department of Psychiatry, School of Medicine, Iran University of Medical Sciences, Tehran, Iran | Iran |  | Soode.t.s@gmail.com | Local Team Leader |
| khatereh | Alipour |  |  |  | Khaterehalipour116650@gmail.com | Data Collection |
| Zahra | Rahimi | School of Medicine, Iran University of Medical Sciences, Tehran, Iran | Iran | 0000-0002-1146-4025 | Zahra.r.98@gmail.com | Data Collection |
| Sara | Hajisadeghi Kahdooyeh | Iran University of Medical Sciences, Tehran, Iran | Iran |  | sara71hk262@gmail.com | Data Collection |
| Mohamed | Gawad Husseiny | a resident of Psychiatry/Tehran University of Medical Science | Iran |  | mohammadjavadhosseini70@gmail.com | Data Collection |
| Soode | Tajik Esmaeeli | Assistant professor of psychiatry, Fellowship of Psychosomatic Medicine, Rasool-e Akram Hospital, Mental Health Research Center, Psychosocial Health Research Institute, Department of Psychiatry, School of Medicine, Iran University of Medical Sciences, Tehran, Iran | Iran |  | Soode.t.s@gmail.com | Local Team Leader |
| mohammad javad | hosseini | Resident of psychiatry/ Iran university of medical science |  |  | mohammadjavadhosseini70@gmail.com | Data Collection |
| Elaheh | Ghalehnovi | Alborz University of Medical Science | Iran | 0009-0009-8212-3576 | elaheghalehnovi@gmail.com | Data Collection |
| khatereh | Alipour | Psychosis Research Center, University of Social Welfare and Rehabilitation Sciences, Tehran, Iran | Iran |  | Kalipour40@gmail.com | Data Collection |
| Seyyedeh Khatereh | Alipour | Psychosis Research Center, University of Social Welfare and Rehabilitation Sciences, Tehran, Iran | Iran |  | Khaterehalipour116650@gmail.com | Data Collection |
| Rawan | Adel Elsayed | Faculty of Medicine, Alexandria University | Egypt | 0009-0000-9011-4236 | Rowanadelelsayed@gmail.com | Data Collection |
| Aiman | Gmzawi | Faculty of Medicine, Hardhrmout University, Mukalla, Yemen | Yemen | 0000-0003-1456-5291 | asgmzawi@gmail.com | Local Team Leader |
| SAEED MUBARAK EISSA | ALHAMEDI | Faculty of Medicine, Hadramout university, mukalla, yemen |  |  | Saeedalhamdee300@gmail.com | Data Collection |
| Islam | A. Mahmoud | Faculty of Medicine, Assiut university, Egypt | Egypt | 0000-0002-2071-2756 | islam.abodeef310@gmail.com | National/Regional Leader |
| Saeed | Bafataim | Faculty of Medicine, Hadramout university, mukalla, yemen |  |  | saeedbaf50@gmail.com | Data Collection |
| Alshaymaa | Ali | Ain shams university | Egypt | 0000-0001-6867-8326 | alshaymaaali62@gmail.com | Data Collection |
| Abdulnaser | Baanas | Faculty of Medicine _Hadhramout university -Mukalla_Yemen |  |  | aoa.baanas@gmail.com | Data Collection |
| Ahmed | Saadoun | Faculty of Medicine, Mansoura University |  |  | ahmedattifsadoon1@std.mans.edu.eg | Data Collection |
| Amro Essam | Amer | Alexandria Faculty of Medicine | Egypt | 0000-0002-7551-2305 | amr.essam.amer.us@gmail.com | Data Collection |
| Madiha Zehra | Panjwani | Liaquat National Medical College and Hospital Karachi Pakistan | Pakistan | 0000-0002-5371-8214 | madihazehra20111@hotmail.com | Data Collection |
| Jamil | AlHayek | Faculty of Medicine, University of Aleppo, Aleppo, Syria | Syria | 0009-0001-5167-2347 | jamil.hayk88@gmail.com | Data Collection |
| Tarek | Ahmad Mahmoud Zaho | Alexandria Faculty of Medicine, Alexandria university | Egypt | 0009-0002-1960-7104 | Tarekzaho99@gmail.com | Data Collection |
|  |  |  |  |  |  | Data Collection |
| Ahmad | Mustafa | University of Aleppo, Faculty of Medicine, Aleppo, Syria |  | 0000-0002-2211-3490 | ahmed17696@gmail.com | Data Collection |
| Mehreen | Taqi | Dr. VRK Women's Medical College |  |  | batool14032001@gmail.com | Data Collection |
| Yousef | Hesham | Mansoura Faculty of Medicine | Egypt | 0000-0002-3303-7737 | Yousefhesham551@yahoo.com | Data Collection |
| Umaima | Rafiq | Liaquat National Hospital and Medical College |  |  | rafiqumaima@gmail.com | Data Collection |
| Sherif | Wael | Mansoura Faculty of Medicine | Egypt | 0009-0009-9900-0820 | sherif.wael2001@gmail.com | Data Collection |
| Hashim | Khan | Rawalpindi Medical University | Pakistan | 0000-0002-0149-1660 | hashimb09@gmail.com | Data Collection |
| Hashim | Khan | Holy Family Hospital, Rawalpindi, Pakistan | Pakistan | 0000-0002-0149-1660 | hashimb09@gmail.com | Data Collection |
| Shankarsai | Kashyap | BGS Global Institute of Medical Sciences | India | 0000-0001-9865-8473 | shankarsaikashyap@gmail.com | Data Collection |
| Donia | Domiaty | Alexandria Faculty of Medicine | Egypt | 0000-0001-5273-6720 | donia.domiaty2000@gmail.com | Data Collection |
| Marwa Ali | Mahmoud |  |  |  | marwaali199615@gmail.com | Data Collection |
| Abdul Majeed | Momeni | cheragh medical institute | Afghanistan |  | mk.majmomeni10@gmail.com | Data Collection |
| Yashas | Maragowdanahalli Somegowda | BGS Global Institute of Medical Sciences | India | 0009-0009-0357-4281 | yashasms1407@gmail.com | Data Collection |
| Harshitha | A | BGS Global Institute of Medical Sciences | India | 0009-0004-1087-7372 | harshitha17a@gmail.com | Data Collection |
| Achuth | Kantharaj | BGS Global Institute of Medical Sciences | India |  | achuthk812@gmail.com | Data collection |
| Brundha | Arya | BGS Global Institute of Medical Sciences | India | 0000-0003-1413-175X | brundhaarya@gmail.com | Data collection |
| Ahmed | Elamir | Mansoura Faculty of Medicine | Egypt | 0009-0002-2077-8883 | Ahmedelamer194@gmail.com | Data collection |
| mohamed | Hesham | Mansoura Faculty of Medicine | Egypt | 0009-0003-0934-7553 | heshamfarag@std.mans.edu.eg | Data collection |
| Ahmed | Osama | Mansoura Faculty of Medicine | Egypt | 0009-0008-1614-1576 | aosama94@std.mans.edu.eg | Data collection |
| Mohamed | El saka | Mansoura Faculty of Medicine | Egypt | 0009-0004-5304-3175 | mg457121@gmail.com | Data collection |
| Hedayatullah | Ehsan | Medical Sciences Research Center, Ghalib University | Afghanistan | 0000-0001-5970-713X | Hedayatullahehsan@gmail.com | Data collection |
| Abubakr | Yosufi | Kabul University of Medical Sciences | Afghanistan | 0000-0003-2672-0228 | abubakr.educational@gmail.com | local team leader |
| Nour | Sh. Abdel-Aleem | Assiut University, Faculty of Medicine | Egypt | 0000-0002-3773-2702 | noursh3ban411@gmail.com | National/Regional Leader |
| Eithar | Alqady | Assiut University, Faculty of Medicine | Egypt | 0000-0003-05676758 | ethar.alkady@gmail.com | Data collection |
| Omar | Abdel Raheem | Assiut University, Faculty of Medicine | Egypt | 0000-0002-2565-8533 | Omarmoawad627@gmail.com | Data collection |
| Esraa | Elsayed | Assiut University, Faculty of Medicine | Egypt | 0000-0002-8510-7688 | essamesraa52@gmail.com | Data collection |
| Montaser | Gamal | Assiut University, Faculty of Medicine | Egypt | 0000-0002-7008-5535 | montaser_zahran@yahoo.com | Data collection |
| Eman | Soliman | Faculty of Medicine, Ain Shams University | Egypt |  | nona.ali171999@gmail.com | Data Collection |
| MARWA | KAREEM | Tobruk University Faculty of Medicine | Libya |  | marwaali199615@gmail.com | Data collection |
| Mohammad | Delsoz | 1. NOOR Eye-Care Training Center  2. Medical Research Center, Kateb University, Kabul Afghanistan | Afghanistan | 0000-0001-5638-2034 | Delsoz_mohammad@yahoo.com | National leader, hospital leader |
| Shekiba | Madadi | Medical Research Center, Kateb University, Kabul, Afghanistan | Afghanistan | 0000-0003-0318-2973 | Shekiba.madadi@kateb.edu.af | Data collection |
| Ghulam Haidar | Alizada | Medical Research Cen  ter, Kateb University, Kabul, Afghanistan | Afghanistan | 0000-0002-4289-2073 | g.haidar.alizada@kateb.edu.af | Data collection |
| Lutfullah | Qurbani | Medical Research Center, Kateb University, Kabul, Afghanistan | Afghanistan | 0000-0003-1867-8146 | Lutfullah.qurbani@kateb.edu.af | Data collection |
| Sherif | Wael | Intern doctor at Mansoura University Hospitals, Mansoura, Egypt | Egypt | 0009-0009-9900-0820 | sherif.wael2001@gmail.com | Data collection |
